# Epigallocatechin-3-gallate attenuates PAR-2–mediated inflammatory signaling and preserves markers of mitochondrial integrity in osteoarthritic chondrocytes

**DOI:** 10.1371/journal.pone.0357766

**Published:** 2026-11-02

**Authors:** Rajashree Patnaik, Yajnavalka Banerjee

**Affiliations:** College of Medicine, Mohammed Bin Rashid University of Medicine and Health Sciences, Dubai Health, Dubai, United Arab Emirates; Michigan Medicine, UNITED STATES OF AMERICA

## Abstract

Epigallocatechin-3-gallate (EGCG), the principal catechin of green tea, exhibits broad anti-inflammatory properties; however, its effect on protease-activated receptor-2 (PAR-2)–mediated signaling in osteoarthritis (OA) has not been investigated. Here, we report that EGCG (5–10 µM) dose-dependently suppresses PAR-2 expression at both mRNA and protein levels in lipopolysaccharide (LPS)-stimulated human chondrocytes derived from bone marrow mesenchymal stem cells. This upstream inhibition attenuated ERK1/2 phosphorylation (pERK/total ERK ratio reduced below basal levels at 10 µM) and produced concordant suppression of three functionally linked effector arms: (i) pro-inflammatory cytokines (TNF-α, IL-1β, MCP-1), with near-maximal inhibition at 10 µM and a saturation plateau at 20 µM; (ii) the SOX4–ADAMTS-5 catabolic axis (81.9% and 66.2% protein reduction, respectively); and (iii) RANK/RANKL surface expression. EGCG also restored mitochondrial membrane potential and biomass to near-homeostatic levels. Crucially, small interfering RNA (siRNA)-mediated PAR-2 silencing reduced LPS-induced TNF-α by ~38% and substantially blunted EGCG’s incremental efficacy, supporting PAR-2 as a predominant, though not exclusive, mediator of EGCG’s anti-inflammatory action. The effective concentrations employed are physiologically attainable through dietary green tea consumption, as documented by a comprehensive survey of EGCG content across 36 commercial varieties. These findings support PAR-2 as a candidate upstream target of EGCG in OA chondrocytes and suggest a mechanistic model involving PAR-2 → ERK1/2 → TNF-α/SOX4/ADAMTS-5, through which a dietary polyphenol exerts coordinated anti-inflammatory and anti-catabolic effects, while preserving structural and thermodynamic markers of mitochondrial integrity.

## Introduction

Osteoarthritis (OA) is a chronic degenerative arthropathy characterized by progressive articular cartilage erosion, subchondral bone remodeling, and persistent low-grade synovial inflammation, collectively culminating in pain, stiffness, and functional decline [1]. Once considered a disease of senescence, OA is now increasingly diagnosed in working-age adults (30–60 years), driven by rising obesity prevalence, sedentary lifestyles, and cumulative metabolic and environmental exposures [2]. At the molecular level, OA pathogenesis is sustained by a dysregulated cytokine milieu, prominently tumour necrosis factor-α (TNF-α), interleukin-1β (IL-1β), and matrix metalloproteinases, that orchestrates cartilage matrix degradation, nociceptor sensitization, and progressive joint remodeling [3].

Current pharmacological strategies remain largely palliative and are limited. NSAIDs, although effective analgesics, are encumbered by gastrointestinal, renal, and cardiovascular toxicities, particularly with chronic use [4]. Intra-articular corticosteroids provide transient relief but may paradoxically accelerate cartilage degeneration upon repeated administration [5], while opioids are increasingly contraindicated owing to their dependence liability and limited disease-modifying efficacy [6]. Symptomatic slow-acting drugs such as glucosamine and chondroitin remain controversial regarding their clinical benefit [7]. In the absence of a bona fide pathway-specific candidate for disease modification, there is a compelling rationale for investigating nutraceutical compounds capable of modulating the inflammatory and catabolic cascades central to OA pathogenesis [8].

Epigallocatechin-3-gallate (EGCG), the principal polyphenolic catechin of green tea (*Camellia sinensis*), constituting up to 63% of total catechin content, has emerged as a particularly promising candidate in this regard. EGCG possesses 25- to 100-fold greater antioxidant potency than vitamins C or E [9], and has demonstrated broad-spectrum anti-inflammatory and cytoprotective properties across diverse chronic inflammatory disorders [10]. Within the context of joint disease, EGCG suppresses pro-inflammatory mediators (nitric oxide, PGE2, COX-2, iNOS) and matrix-degrading enzymes (MMP-1, MMP-13, ADAMTS-4, ADAMTS-5) in activated chondrocytes while preserving glycosaminoglycan integrity [35,37], and modulates COX-2 and PGE2 production through ERK-dependent signaling [42]. ***In vivo***, intra-articular EGCG ameliorates cartilage degradation in spontaneous guinea pig OA, suppresses IL-1β–induced senescence markers, restores anabolic gene expression (aggrecan, COL2A1), and enhances autophagic flux through mTOR inhibition [36,39].

Despite this substantial body of evidence, the precise effect of EGCG on protease-activated receptor-2 (PAR-2)–mediated inflammatory signaling in OA, a pathway of singular mechanistic importance, remains unexplored. PAR-2 is a G-protein-coupled receptor activated by proteolytic cleavage of its extracellular N-terminus, and its expression in chondrocytes and synovial fibroblasts positions it as a critical upstream regulator of the inflammatory response in OA [11]. PAR-2 activation drives the transcriptional upregulation of TNF-α, IL-1β, IL-6, and MCP-1, augments nociceptive signaling via substance P and CGRP [12], and promotes aggrecanase-mediated cartilage catabolism. Notably, Bono *et al.* established that PAR-2 activation directly induces vascular smooth muscle cell proliferation through discrete intracellular signaling cascades [13], while subsequent work demonstrated that PAR-2 deficiency in hematopoietic lineage cells attenuates inflammatory infiltration and fibrosis following myocardial infarction [14], underscoring the receptor’s broad pro-inflammatory significance beyond the joint. Furthermore, articular chondrocytes constitutively express multiple ADAMTS family members [15], and IL-1β-induced COX-2 expression and PGE2 production in human chondrocytes are critically dependent on ERK1/2 isoform-specific activation [16], the very downstream effectors through which PAR-2 transduces its pro-inflammatory signals. In the chondrocyte context specifically, our group previously demonstrated that curcumin downregulates PAR-2 expression and suppresses its downstream inflammatory cascade in LPS-stimulated human chondrocytes [17], and that oleocanthal selectively attenuates PAR-2-mediated cytokine release and catabolic enzyme activity [18]. These findings collectively establish PAR-2 signaling as a tractable and therapeutically significant axis in OA.

However, whether EGCG, notwithstanding its well-characterized capacity to modulate ERK, NF-κB, and mTOR signaling, can directly attenuate PAR-2-driven inflammatory processes in OA chondrocytes has not been investigated. This represents a critical knowledge gap, given that PAR-2 occupies a nodal position upstream of the very cytokine networks and catabolic enzymes that EGCG is known to suppress. In this study, we directly address this lacuna by interrogating the effect of EGCG on PAR-2 expression and its downstream inflammatory mediators, including TNF-α, SOX4, ADAMTS-5, and the ERK1/2 phosphorylation axis in an LPS-stimulated pro-inflammatory chondrocyte model of OA. Through integrated assessment of protein expression, gene regulation, RANK/RANKL surface dynamics, and mitochondrial functional integrity, complemented by siRNA-mediated PAR-2 silencing to assess mechanistic dependence, we provide evidence that EGCG may function as a targeted modulator of the PAR-2 signaling axis in osteoarthritic chondrocytes, thereby supporting its investigation as a pathway-relevant candidate in OA, a proposition now requiring in vivo validation.

## Materials and methods

### Ethics considerations for the present study

All experimental procedures in this study were performed exclusively ***in vitro*** and did not involve animal models, human participants, patient-derived samples, or associated data. Consequently, the research posed minimal risk and falls under the exempt review categories defined by the Institutional Review Board (IRB) at Mohammed Bin Rashid University (MBRU). Additional details or clarification can be obtained from the MBRU IRB (irb@mbru.ac.ae). As the work involved neither human participants nor identifiable human data, informed consent was not applicable and was formally waived by the MBRU IRB.

### Cell culture and chondrogenic differentiation

Human bone marrow-derived mesenchymal stem cells (BM-MSCs; AddexBio Technologies, San Diego, CA, USA) were cultured in mesenchymal stem cell growth medium (MSCM; AddexBio Technologies) supplemented with 10% fetal bovine serum (FBS; Himedia, Mumbai, India) and 1% penicillin-streptomycin (Himedia) at 37 °C in a humidified 5% CO_2_ atmosphere. Medium was replenished every 72 h, and cells were passaged at ~90% confluency using trypsin/EDTA (Himedia). For chondrogenic differentiation, passage 5 BM-MSCs (2 × 10^6^ cells), enumerated by automated cell counter (DeNovix CellDrop, Wilmington, DE, USA), were pelleted by centrifugation (1000 rpm, 10 min) in 15-mL polypropylene tubes to establish scaffold-free three-dimensional micromass cultures that recapitulate the native cartilage microenvironment. Pellets were resuspended in 2 mL of complete chondrogenic differentiation medium (Stem Cell Technologies, Vancouver, Canada), distributed equally into four tubes, and centrifuged again under identical conditions. Cultures were maintained at 37 °C with 5% CO_2_; fresh medium was supplemented on day 3 and subsequently replaced every 72 h for 21 days, with pellets gently dislodged at each exchange to prevent mural adhesion. Following an additional 7-day maturation period, differentiated chondrocytes were cryopreserved in DMEM containing 5% DMSO (Himedia) at −80 °C overnight prior to transfer to liquid nitrogen (Passage 5 after differentiation). The chondrogenic differentiation and the histochemical and immunocytochemical staining procedures used here to validate the chondrocytic phenotype followed our previously established and published protocols [17–19].

### Histological assessment

Cell pellets were fixed in 10% neutral-buffered formalin (24 h), processed through graded ethanol dehydration, xylene clearing, and paraffin embedding. Sections (6 µm) were prepared on a rotary microtome (Leica RM2255, Nussloch, Germany). Chondrogenic phenotype was verified by collagen type II immunostaining, together with Alcian Blue and Toluidine Blue staining (Abcam, Waltham, MA, USA). All sections were imaged using an Olympus BX63 microscope equipped with a DP80 camera and CellSens Dimension 2.3 software (Olympus, Tokyo, Japan) at 20× and 40× magnification.

### Cytotoxicity assessment, inflammatory chondrocyte model, and TNF-α quantification

Cytotoxic profiles of epigallocatechin-3-gallate (EGCG; MedChem Express, Monmouth Junction, NJ, USA) and lipopolysaccharide (LPS; Thermo Fisher Scientific, Waltham, MA, USA) were independently determined by MTT assay (Thermo Fisher Scientific) as previously described [17,19].

LPS (a high-affinity TLR4 ligand) was selected as the pro-inflammatory stimulus because it reproduces the TLR4-driven signalling that endogenous damage-associated molecular patterns activate in osteoarthritic cartilage, including the TLR4-NF-κB inductive pathway for PAR-2 expression; the mechanistic rationale for this choice is developed in the Discussion. The model was established at LPS 10 µg/mL, a concentration validated in prior work from our group to elicit robust pro-inflammatory signalling without cytotoxicity [17–19]. This concentration lies within the 1–10 µg/mL range conventionally employed to elicit reproducible TLR4-dependent inflammatory signalling in chondrocytes in vitro, and was selected to ensure a robust, reproducible stimulus; its relationship to the lower lipopolysaccharide concentrations encountered physiologically, and the translational rationale for this choice, are considered in detail in the Discussion.

An *in vitro* inflammatory chondrocyte model was generated by stimulating differentiated chondrocytes with LPS (10 µg/mL, 24 h, 37 °C, 5% CO_2_), a concentration validated to elicit robust pro-inflammatory signaling without appreciable cytotoxicity [17,19]. To evaluate the anti-inflammatory efficacy of EGCG, LPS-primed chondrocytes cultured in 6-well plates were subsequently treated with EGCG (0, 1, 5, 10, and 20 µM) for an additional 24 h. A broader concentration range than the 5–10 µM working concentrations used in the mechanistic experiments was applied for this cytokine assay in order to resolve the full concentration–response relationship: 1 µM (near-inactive) anchors the lower baseline, 5 and 10 µM span the steep sub-maximal region, and 20 µM defines the upper plateau, confirming a saturable, concentration-dependent effect; adequate sampling of both the lower baseline and the upper plateau in this way is a prerequisite for accurate characterisation of a concentration–response curve [20]. Conditioned supernatants were harvested by centrifugation (5000 rpm, 10 min), and TNF-α concentrations were quantified by enzyme-linked immunosorbent assay (ELISA; Abcam) per manufacturer’s instructions. Because TNF-α, in common with the other cytokines quantified here (IL-1β and MCP-1), is a secreted protein released into the extracellular medium rather than retained intracellularly, it was measured directly in the conditioned supernatant by ELISA, the standard quantitative immunoassay for soluble cytokines [21], rather than by immunoblotting of cell lysates, which is appropriate for cell-associated protein. Optical densities were recorded at 450 nm (reference: 620 nm) on a Hidex microplate reader (Turku, Finland).

### Western blotting

Total protein was extracted using 0.5% SDS lysis buffer (Invitrogen, Thermo Fisher Scientific, Carlsbad, CA, USA) supplemented with protease inhibitor cocktail (Roche Diagnostics), followed by clarification at 14,000 × g for 10 min at 4 °C. Protein concentrations were determined by bicinchoninic acid (BCA) assay (Thermo Fisher Scientific). Based on prior optimisation and pharmacokinetic evidence demonstrating physiologically achievable plasma levels following high-dose oral supplementation (800–1600 mg) with enhanced-bioavailability formulations [22,23], EGCG was administered at 5 µM and 10 µM.

Equal amounts of protein (15 µg) were resolved by 10% SDS–PAGE and electro-transferred onto nitrocellulose membranes (Bio-Rad Laboratories, Mississauga, ON, Canada) via semi-dry transfer. Membranes were blocked with 5% bovine serum albumin (BSA) in Tris-buffered saline (TBS) for 1 h at room temperature, washed thrice with TBS containing 0.1% Tween-20 (TTBS), and incubated overnight at 4 °C with the following primary antibodies (1:1000; Abcam): mouse anti-human PAR-2 (catalogue number, ab184673; mouse monoclonal; clone number, SAM11; Abcam, validated for WB by manufacturer), rabbit anti-human SOX4 (catalogue number, ab316850; rabbit monoclonal; clone number, EPR28132-51; Abcam, validated for WB by manufacturer), rabbit anti-human ADAMTS-5 (catalogue number, ab41037; rabbit polyclonal; Abcam, validated for WB by manufacturer), rabbit anti-human ERK1/2 (catalogue number, ab184699; rabbit monoclonal; clone number, EPR17526; Abcam, validated for WB by manufacturer), and rabbit anti-human phospho-ERK1/2 (Thr202/Tyr204) (catalogue number, 9101S; rabbit polyclonal; Cell Signaling Technology, validated for WB by manufacturer). Following TTBS washes, membranes were probed with HRP-conjugated anti-mouse (catalogue number, ab205719; polyclonal; Abcam, validated for WB by manufacturer) or anti-rabbit (catalogue number, ab6721; polyclonal; Abcam, validated for WB by manufacturer) secondary antibodies (1:2000) for 1 h at room temperature. Chemiluminescent detection was performed using SuperSignal™ West Pico PLUS Chemiluminescent Substrate (Thermo Fisher Scientific), and signals were captured using an Azure Biosystems imaging system (Azure Biosystems, Dublin, CA, USA). Band intensities were quantified by densitometry using ImageJ (NIH, Bethesda, MD, USA) normalized to GAPDH (catalogue number, ab9482; mouse monoclonal; clone number, mAbcam 9484; Abcam, validated for WB by manufacturer). Target protein expression was normalized to the corresponding GAPDH band intensity from the same lane. The normalized values were then expressed relative to the LPS-only group, which was set to 1.0. All immunoblots were performed in triplicate **(three independent biological replicates)**.

### RNA extraction, cDNA synthesis, and quantitative PCR

Total RNA was isolated from control and treated chondrocytes using an RNA isolation kit (NZYTech, Lisbon, Portugal), and concentration and purity were assessed using a NanoDrop spectrophotometer (Thermo Fisher Scientific). First-strand cDNA was synthesised using the First-Strand cDNA Synthesis Kit (OriGene, Heidelberg, Germany) according to the manufacturer’s protocol [24]. Quantitative PCR was performed on a QuantStudio 5 Flex system (Applied Biosystems, Thermo Fisher Scientific) using gene-specific primers (Table 1) designed via OriGene’s proprietary algorithm, with specificity confirmed *in silico* by BLAST analysis [25,26]. All reactions included no-template controls and were run in technical replicates. Relative gene expression was calculated by the 2^−ΔΔCT^ method with GAPDH as the endogenous reference [27].

**Table 1:** Oligonucleotide primers used for real-time PCR.

Gene	Primer Type	Sequence	Accession	E-value	Bit Score
PAR-2	Forward (5’-3’) Reverse (5’-3’)	CTCCTCTCTGTCATCTGGTTCC TGCACACTGAGGCAGGTCATGA	NM_005242	0.0	2861
GAPDH	Forward (5’-3’) Reverse (5’-3’)	GTCTCCTCTGACTTCAACAGCG ACCACCCTGTTGCTGTAGCCAA	NM_002046	0.0	2374
TNF-α	Forward (5’-3’) Reverse (5’-3’)	CTCTTCTGCCTGCTGCACTTTG ATGGGCTACAGGCTTGTCACTC	NM_000594	0.0	2449
DUSP6	Forward (5’-3’) Reverse (5’-3’)	GTCCCAAGGCTTTGGAATCTGTC CCTACCAAGACAGGAGTTCTGG	NM_001946	0.0	3627
ERK 1	Forward (5’-3’) Reverse (5’-3’)	TGGCAAGCACTACCTGGATCAG GCAGAGACTGTAGGTAGTTTCGG	NM_002746	0.0	1787
ERK 2	Forward (5’-3’) Reverse (5’-3’)	ACACCAACCTCTCGTACATCGG TGGCAGTAGGTCTGGTGCTCAA	NM_002745	0.0	5881
SOX 4	Forward (5’-3’) Reverse (5’-3’)	GACATGCACAACGCCGAGATCT GTAGTCAGCCATGTGCTTGAGG	NM_003107	0.0	4869
ADAMTS 5	Forward (5’-3’) Reverse (5’-3’)	CCTGGTCCAAATGCACTTCAGC TCGTAGGTCTGTCCTGGGAGTT	NM_007038	0.0	9621

PAR-2: Protease Activated Receptor-2; GAPDH: Glyceraldehyde-3-phosphate dehydrogenase; DUSP6: Dual specificity phosphatase 6; ERK 1: Extracellular signal-regulated kinase 1; ERK 2: Extracellular signal-regulated kinase 2; SOX 4: SRY-box transcription factor 4; ADAMTS 5: A Disintegrin and Metalloproteinase with Thrombospondin Motifs 5.

### Flow cytometric analysis of RANK and RANKL surface expression

Chondrocytes treated with EGCG (5 µM and 10 µM, 24 h) were detached with 0.25% trypsin–EDTA (Himedia), pelleted at 500 × g for 5 min at 4 °C, and resuspended at 1 × 10^6^ cells/mL in phosphate-buffered saline (PBS). For surface immunolabelling, cells were incubated (30 min, 4 °C, light-protected) with PE-conjugated mouse anti-human RANK (20 µg/mL; Abcam) (catalogue number, ab233817; mouse monoclonal; clone number, 8H8B2; Abcam, validated for Flow cytometry analysis and WB by manufacturer) or unconjugated rabbit anti-human RANKL (15 µg/mL; Abcam) (catalogue number, ab216484; rabbit polyclonal; Abcam, validated for Flow cytometry analysis and WB by manufacturer) followed by FITC-conjugated goat anti-rabbit secondary antibody (7.5 µg/mL; Abcam) (catalogue number, ab97050; polyclonal; Abcam, validated for Flow cytometry analysis and WB by manufacturer). Isotype-matched immunoglobulins served as negative controls. Samples were acquired on a FACSCalibur flow cytometer (BD Biosciences, Mississauga, ON, Canada) and analyzed using FlowJo software (Tree Star Inc., Ashland, OR, USA). The complete gating strategy, sequential exclusion of debris (FSC-A vs SSC-A), single-cell discrimination (FSC-A vs FSC-H), and the RANK- and RANKL-positive thresholds set against isotype/negative controls, is provided in S2 Fig. All experiments were performed in triplicate.

### Mitochondrial membrane potential and activity

Mitochondrial membrane potential (ΔΨm) was assessed by Rhodamine 123 staining. Following EGCG treatment (5 µM and 10 µM), cells were incubated with 2 µM Rhodamine 123 (Thermo Fisher Scientific, Carlsbad, CA, USA) for 15 min at room temperature, washed, and imaged by fluorescence microscopy (Olympus). Mean fluorescence intensity was quantified using ImageJ and expressed as a percentage reduction relative to untreated controls.

Mitochondrial biomass was evaluated using Janus Green B staining (Sigma-Aldrich, St. Louis, MO, USA). Following treatment, chondrocytes were stained with 0.01% (w/v) Janus Green B in PBS for 5 min. Excess dye was removed by gentle washing, and destaining was performed using 0.5% acetic acid in ethanol for 5 min. Representative fluorescence micrographs were acquired using an inverted fluorescence microscope prior to dye elution. For quantitative assessment, the retained dye was extracted with 0.5 N HCl in ethanol, and optical density was measured spectrophotometrically at 595 nm. All conditions were assessed in triplicate, and absorbance values were normalized and expressed as a percentage relative to untreated control cells.

### siRNA-mediated PAR-2 silencing

To delineate PAR-2’s contribution to TNF-α regulation under inflammatory conditions, PAR-2 expression was selectively silenced using small interfering RNA (siRNA). PAR-2-specific siRNA was complexed with Oligofectamine (OriGene, US) at 1:4 and 1:3 ratios, respectively, and incubated at room temperature for 15 min. Chondrocytes in 100-mm dishes were washed thrice with serum-free DMEM and incubated with the siRNA–lipid complexes (final siRNA concentration: 0.1 µM) for 5 h at 37 °C. Transfection medium was then replaced with complete DMEM supplemented with 10% FBS, and cells were allowed to recover for 24 h before stimulation with LPS in the presence or absence of EGCG. Knockdown efficiency and downstream effects on TNF-α expression were verified by Western blotting and qPCR, respectively.

### Statistical analysis

All data are expressed as mean ± standard deviation (SD) of n  =  3 independent biological experiments, each comprising a fully independent BMSC-derived chondrocyte culture established, differentiated, treated, and measured on separate occasions. Group differences were evaluated by one-way analysis of variance (ANOVA) across the four treatment conditions (untreated control, LPS, LPS  +  EGCG 5 µM, and LPS  +  EGCG 10 µM), followed by Dunnett’s multiple-comparison post-hoc test, in which each treatment condition was compared against the common LPS-stimulated reference group: (i) untreated chondrocytes vs. LPS; (ii) LPS vs. LPS  +  EGCG 5 µM; and (iii) LPS vs. LPS  +  EGCG 10 µM. Although it has been argued that pre-planned comparisons defined a priori against a single biological reference do not strictly require family-wise correction [28], we nonetheless adopted Dunnett’s procedure to provide a conservative and widely accepted evaluation of group differences, as it is specifically designed for the comparison of several treatment groups against a single control and controls the family-wise error rate [29], in keeping with established recommendations for handling multiple comparisons in biological research [30].

For each assay, n = 3 denotes three independent biological replicates, defined as fully separate chondrocyte cultures established, differentiated, treated, and assayed on different occasions, rather than technical replicates of a single culture [31–33]. A p-value of <0.05 was considered statistically significant. Analyses were conducted using GraphPad Prism v9.5.1 (GraphPad Software, San Diego, CA, USA).

## Results

### Validation of chondrogenic differentiation

Following 28 days of chondrogenic induction, BM-MSCs transitioned from a spindle-shaped fibroblastic morphology (Fig 1A) to a phenotype consistent with functional chondrogenesis. Phase-contrast microscopy at this 28-day endpoint confirmed this transition at the morphological level: the cells had lost the elongated, spindle-shaped fibroblastic appearance of the undifferentiated population (Fig 1A) and adopted a more compact, rounded, polygonal chondrocyte-like morphology with denser intercellular contacts (Fig 1B), consistent with the progressive acquisition of a chondroblastic phenotype described during BM-MSC chondrogenesis [34]. Alcian Blue staining revealed abundant sulphated glycosaminoglycan (GAG) deposition within the extracellular matrix (Fig 1C), while Toluidine Blue demonstrated robust metachromasia indicative of dense proteoglycan-rich matrices (Fig 1D). Immunocytochemical analysis confirmed intense collagen type II expression (Fig 1E), the definitive molecular marker of chondrogenic commitment. Together, these data verify the acquisition of a mature chondrocytic phenotype under the induction conditions employed.

**Fig 1:**
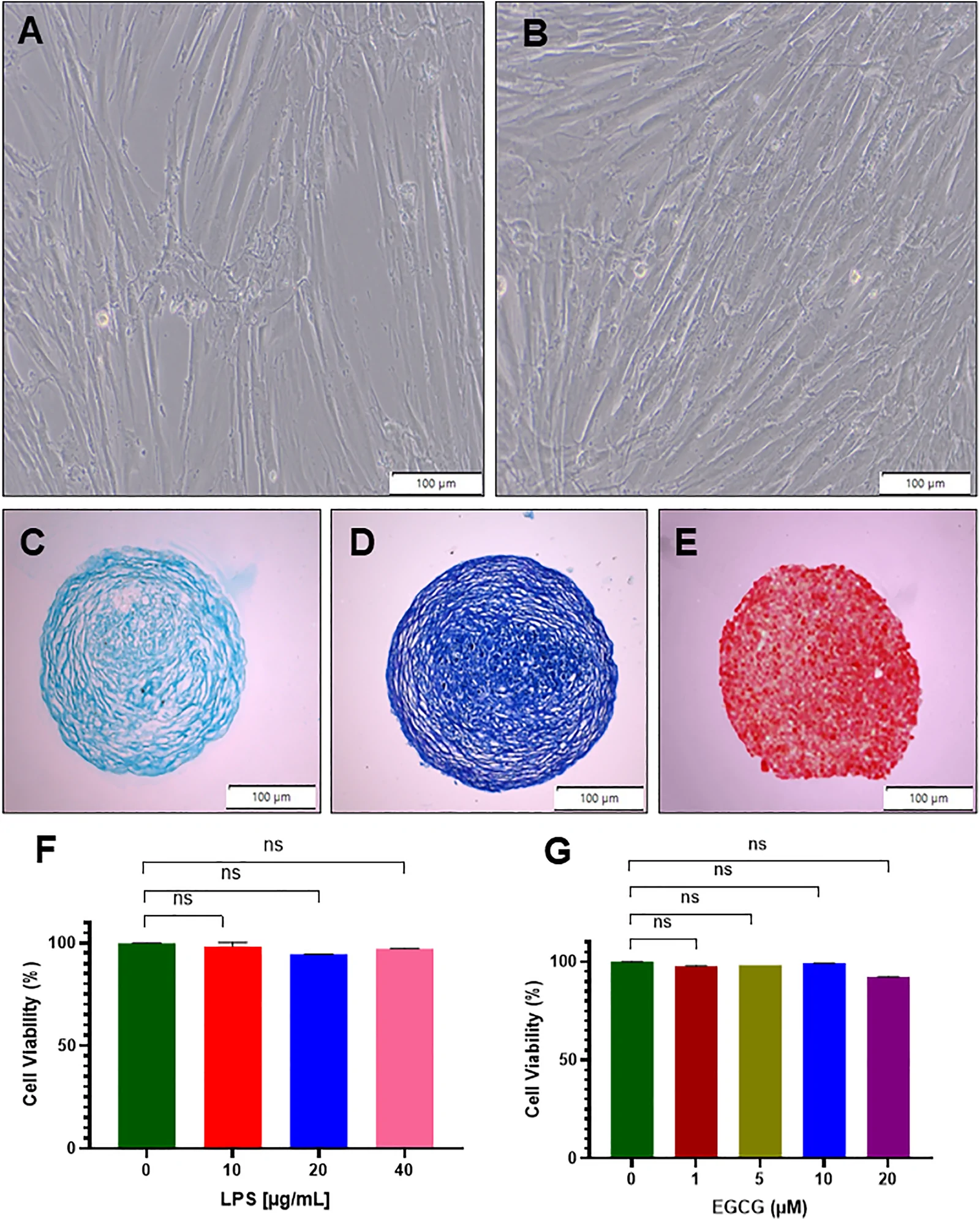
Chondrogenic differentiation and cytotoxicity profiling. (A) Phase-contrast micrograph of undifferentiated BM-MSCs showing spindle-shaped fibroblastic morphology. (B) Phase-contrast micrograph of the same population after 28-day chondrogenic induction, showing the transition to a compact, rounded, polygonal chondrocyte-like morphology. (C) Alcian Blue staining demonstrating sulphated glycosaminoglycan deposition after 28-day chondrogenic induction. (D) Toluidine Blue staining confirming metachromatic proteoglycan-rich matrix. (E) Immunocytochemical detection of collagen type II expression. (F) MTT viability assay: LPS (0–40 µg/mL) demonstrates no significant cytotoxicity across the tested range. (G) MTT viability assay: EGCG (0–20 µM) demonstrates no significant reduction in chondrocyte viability. Data are mean ± SD (n = 3). Scale bars: 100 µm (A–E).

### Cytotoxicity profiling of EGCG and LPS

LPS exhibited no discernible cytotoxicity across the tested concentration range (0–40 µg/mL; Fig 1F), validating its suitability for establishing the pro-inflammatory model. Similarly, EGCG demonstrated no significant reduction in chondrocyte viability at concentrations up to 20 µM (Fig 1G). The working concentrations selected for all subsequent experiments (5 µM and 10 µM EGCG; 10 µg/mL LPS) thus fall well within the non-cytotoxic range, ensuring that all observed downstream effects reflect pharmacological modulation rather than cytotoxic artefacts.

Protein expression levels, phosphorylation status, and downstream signaling changes—including PAR2, pERK1/2, total ERK1/2, SOX4, and ADAMTS5—were evaluated across the experimental treatment groups using full-length uncropped Western blot membranes with GAPDH as an internal loading control (Supplementary S1 Fig).

### EGCG downregulates PAR-2 expression in LPS-stimulated chondrocytes

LPS stimulation markedly upregulated PAR-2 protein expression in chondrocytes relative to untreated controls (Fig 2A). Densitometric analysis normalised to GAPDH (Fig 2B) confirmed this induction, which was significantly attenuated by 5 µM EGCG (p < 0.001) and further suppressed by 10 µM EGCG to sub-basal levels (p < 0.001). RT-PCR analysis (Fig 2C) revealed an approximately 4-fold increase in PAR-2 mRNA following LPS exposure, which EGCG dose-dependently abrogated, achieving significant suppression at 10 µM (p < 0.01). The concordance between protein and transcript data indicates that EGCG attenuates PAR-2 expression through a transcriptionally driven mechanism.

**Fig 2:**
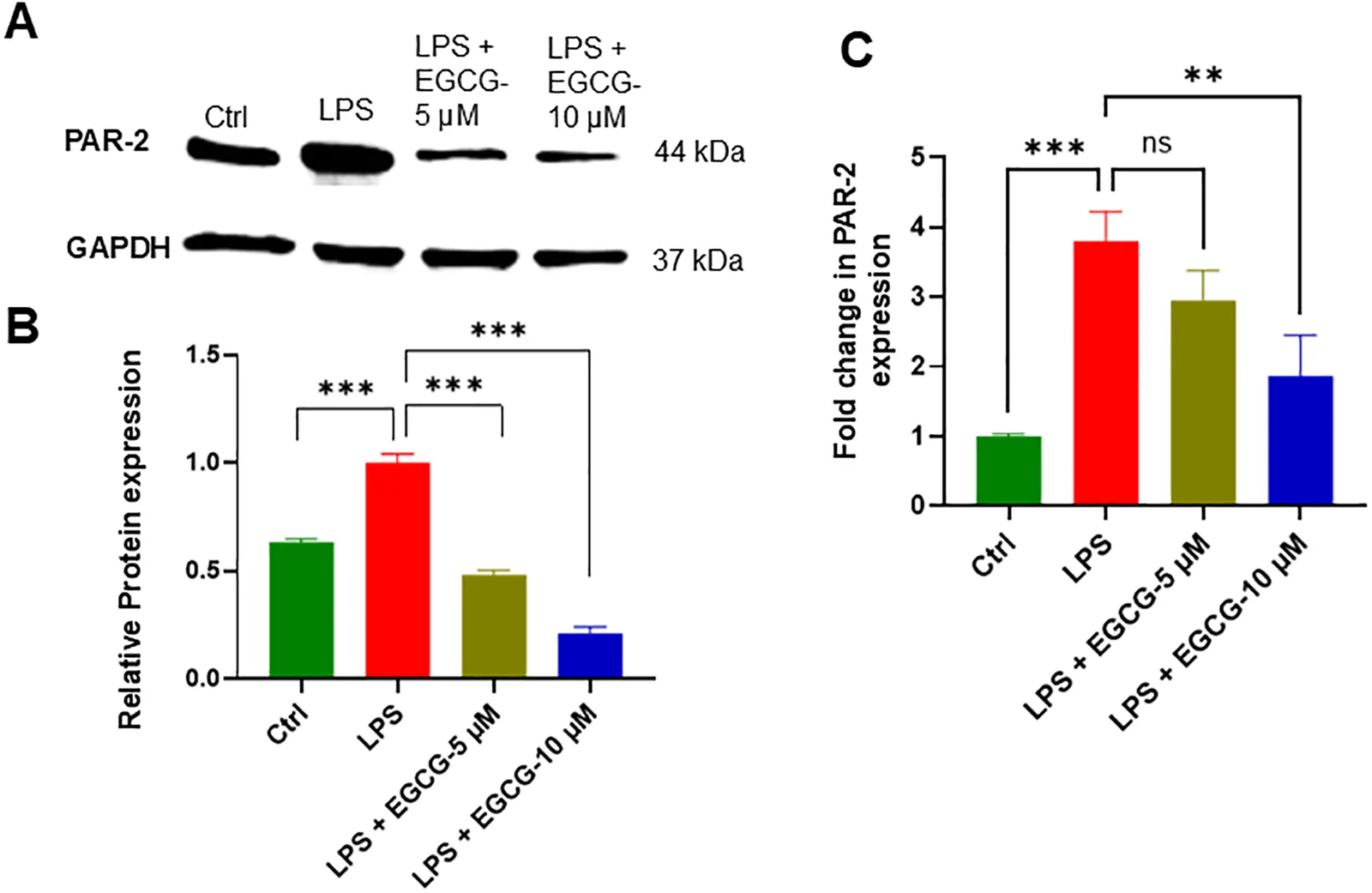
EGCG dose-dependently downregulates PAR-2 expression at both the protein and mRNA level in LPS-stimulated human chondrocytes. BM-MSC-derived chondrocytes were pre-treated with EGCG (5 or 10 µM) for 2 h prior to stimulation with LPS (10 µg/mL) for 24 h. Untreated chondrocytes served as the negative control. (A) Representative western blot images showing PAR-2 protein expression across the four treatment conditions (Control, LPS, LPS + 5 µM EGCG, LPS + 10 µM EGCG). GAPDH was used as the loading control. Full-length, unprocessed blot images are provided in the Supplementary Data. (B) Densitometric quantification of PAR-2 protein band intensity, normalized to the corresponding GAPDH band for each lane and expressed relative to the LPS-only group. (C) RT-PCR analysis of PAR-2 mRNA expression, quantified by the 2^−ΔΔCT^ method and expressed as fold change relative to the untreated control, with GAPDH serving as the reference gene. Data are presented as mean ± SD (n = 3 independent biological experiments). *p<0.05, **p<0.01, ***p<0.001 versus LPS-only group (one-way ANOVA with Dunnett’s post-hoc test).

### EGCG suppresses ERK1/2 phosphorylation and transcript expression downstream of PAR-2

Western blot analysis (Fig 3A) demonstrated robust LPS-induced ERK1/2 phosphorylation. Densitometry (Fig 3B) confirmed significant increases in pERK1/2 levels, which were dose-dependently reduced by EGCG at both 5 µM and 10 µM (p < 0.01 at 5 µM; p < 0.001 at 10 µM). Total ERK1/2 protein was also elevated by LPS and modestly suppressed by EGCG (Fig 3C; p < 0.01 and p < 0.001). The pERK/total ERK ratio (Fig 3D), which isolates activation-state changes from total protein fluctuations, was markedly increased by LPS (p < 0.001 vs. control) and dose-dependently reversed by EGCG, with 10 µM driving the ratio below basal levels (p < 0.01 at 5 µM; p < 0.001 at 10 µM). This sub-basal restoration of the activation ratio indicates preferential inhibition of ERK1/2 phosphorylation exceeding the reduction in total protein abundance, consistent with upstream kinase interference or enhanced phosphatase activity.

**Fig 3:**
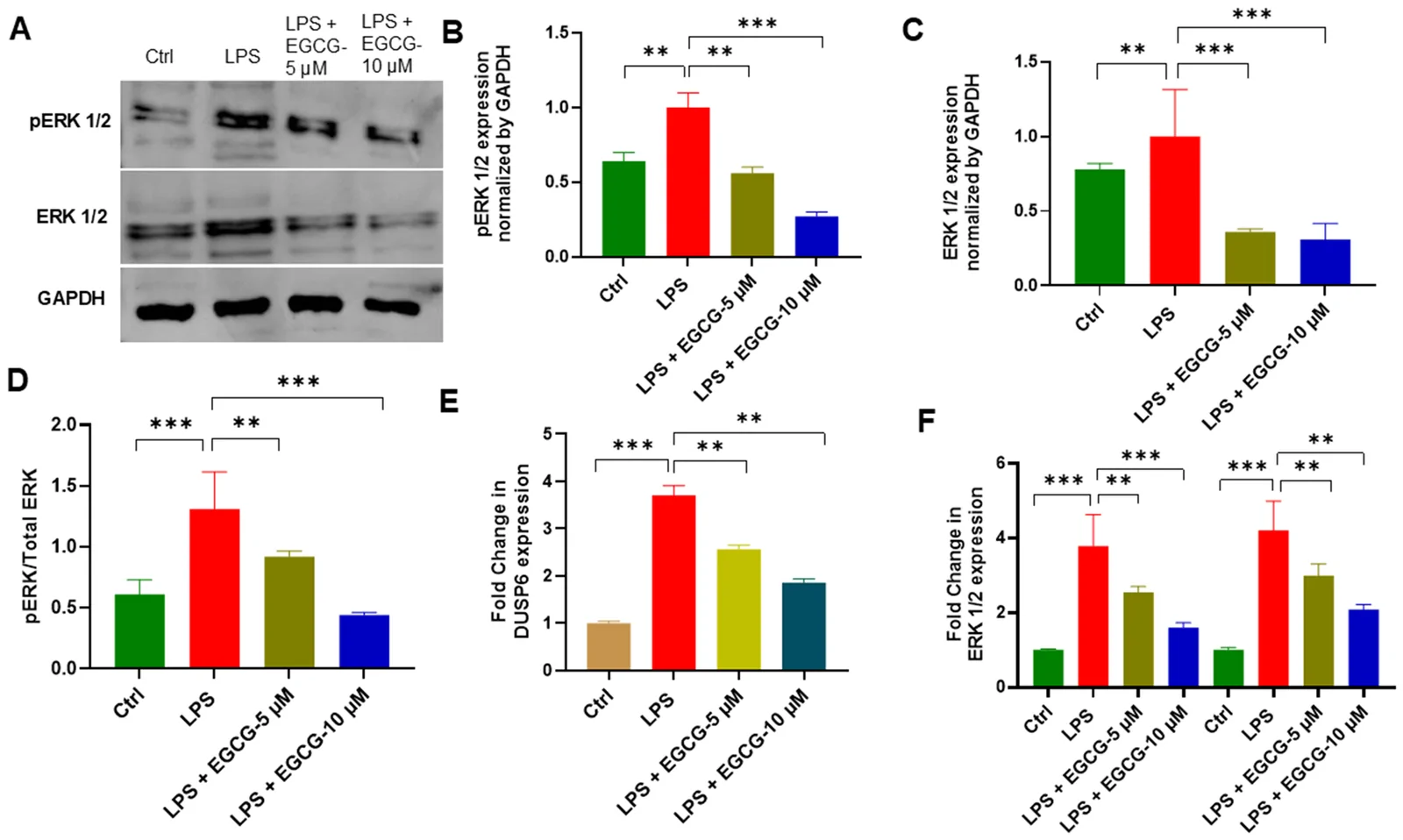
EGCG attenuates ERK1/2 phosphorylation and modulates ERK pathway transcript expression in LPS-stimulated chondrocytes. Cells were treated as described in Fig 2. (A) Representative western blot images for phosphorylated ERK1/2 (pERK1/2; Thr202/Tyr204), total ERK1/2, and GAPDH across all four treatment conditions. Full-length blot images are provided in the Supplementary Data. (B) Densitometric quantification of pERK1/2 band intensity, normalized to GAPDH and expressed relative to the LPS-only group. (C) Densitometric quantification of total ERK1/2 protein, normalized to GAPDH. (D) Ratio of pERK1/2 to total ERK1/2 across treatment groups, reflecting the net phosphorylation status of the ERK1/2 kinase. (E) RT-PCR analysis of DUSP6 (dual-specificity phosphatase 6) mRNA expression, a transcriptional target and negative feedback regulator of ERK1/2 signaling, expressed as fold change relative to the untreated control. (F) Independent RT-PCR quantification of ERK1 and ERK2 transcript levels, expressed as fold change relative to the untreated control. Data are presented as mean ± SD (n = 3 independent biological experiments). *p < 0.05, **p < 0.01, ***p < 0.001 versus LPS-only group (one-way ANOVA with Dunnett’s post-hoc test).

At the transcriptional level, LPS induced a ~ 3.8-fold increase in DUSP6 mRNA (Fig 3E), which was significantly attenuated by EGCG at both concentrations (p < 0.01). ERK1 and ERK2 transcripts, assessed independently (Fig 3F), were both upregulated by LPS (~3.8-fold and ~4.0-fold, respectively) and suppressed by EGCG in an isoform-concordant, dose-dependent manner (p < 0.01 to p < 0.001), excluding compensatory upregulation of one isoform upon suppression of the other. Together with the PAR-2 data (Fig 2), these findings support a coordinated, multi-level inhibitory action of EGCG on the PAR-2–ERK1/2 axis, operating simultaneously at receptor expression, kinase activation, and transcript availability.

### EGCG suppresses TNF-α, IL-1β, and MCP-1 at both protein and transcript levels

ELISA quantification of conditioned supernatants (Fig 4A) demonstrated that LPS stimulation elevated TNF-α, IL-1β, and MCP-1 secretion to maximal levels (normalized to 100%) relative to basal expression in untreated chondrocytes (38.68 ± 2.01%, 44.06 ± 1.55%, and 40.09 ± 0.97%, respectively). EGCG co-treatment produced concentration-dependent suppression across all three cytokines. At 1 µM, reductions were negligible (TNF-α: 98.23%; IL-1β: 97.3%; MCP-1: 96.43%), whereas 5 µM elicited appreciable suppression (TNF-α: 76.42 ± 2.03%; IL-1β: 70.8 ± 1.55%; MCP-1: 66.9 ± 1.15%), with MCP-1 exhibiting the greatest dose-sensitivity at this concentration. At 10 µM, expression was reduced to approximately one-third of LPS-stimulated levels (TNF-α: 34.66 ± 2.00%; IL-1β: 41.4 ± 2.03%; MCP-1: 39.21 ± 2.8%). The marginal incremental benefit observed between 10 µM and 20 µM (TNF-α: 31.08 ± 1.08%; IL-1β: 38.37 ± 1.08%; MCP-1: 35.7 ± 1.76%) indicates near-maximal target engagement at 10 µM, consistent with a saturable inhibitory mechanism.

**Fig 4:**
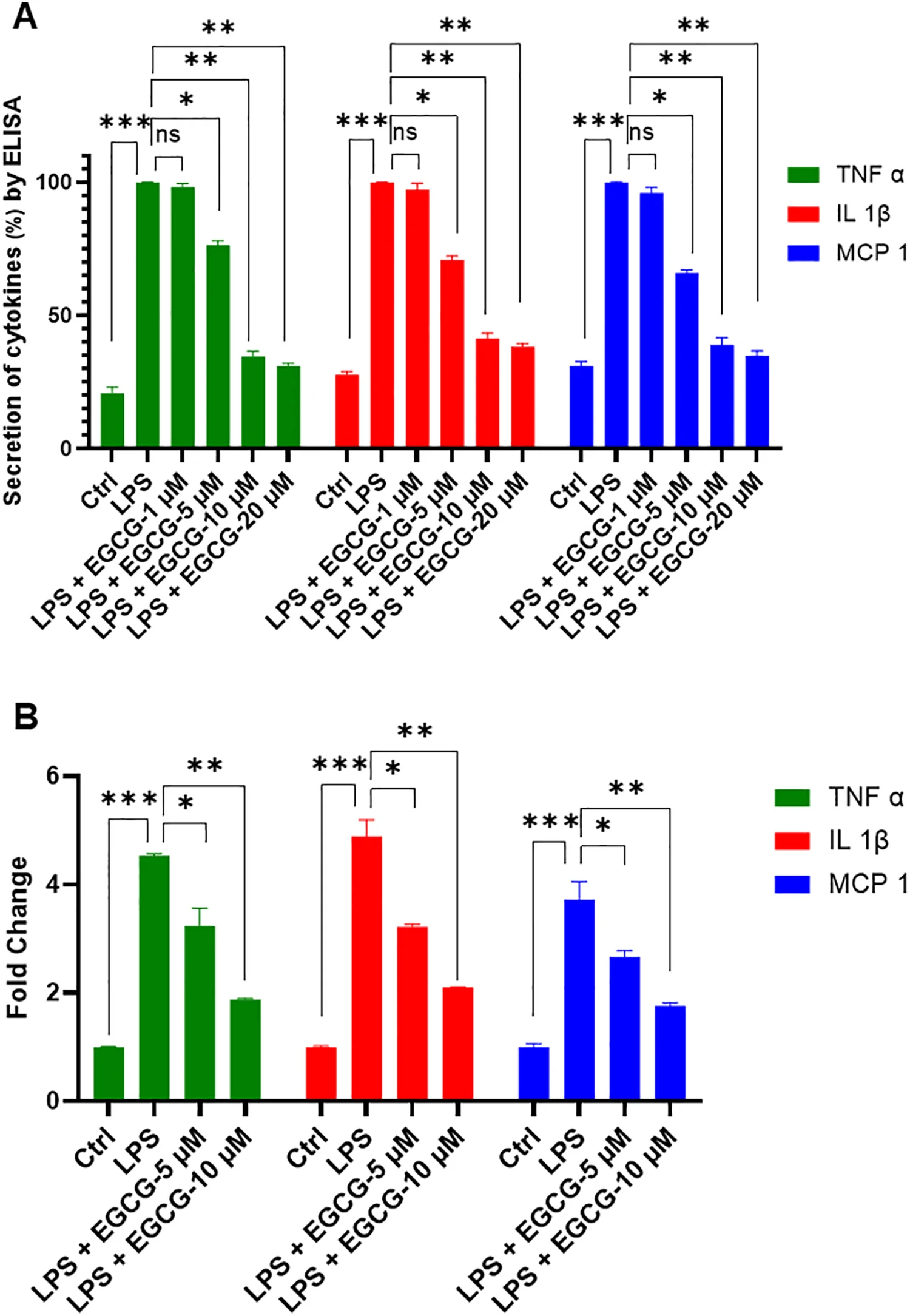
EGCG suppresses TNF-α, IL-1β, and MCP-1 at protein and transcript levels in LPS-stimulated chondrocytes, demonstrating concentration-dependent, transcriptionally driven repression of the three principal pro-inflammatory mediators. (A) ELISA quantification of cytokine secretion in conditioned supernatants across EGCG concentrations (0, 1, 5, 10, 20 µM). Values are expressed as percentage expression (± SD) relative to LPS-stimulated controls (normalized to 100%). (B) RT-PCR analysis of TNF-α, IL-1β, and MCP-1 mRNA expression (fold change). Data are mean ± SD (n = 3). *p < 0.05, **p < 0.01, ***p < 0.001 vs. LPS-only group.

qPCR analysis (Fig 4B) corroborated these findings at the transcript level. LPS induced robust upregulation of TNF-α, IL-1β, and MCP-1 mRNA (~4.5-, ~ 4.5-, and ~3.8-fold, respectively; p < 0.001 vs. control). EGCG produced concordant dose-dependent suppression, with 5 µM significantly attenuating all three transcripts (p < 0.05 for TNF-α, IL-1β and MCP-1) and 10 µM achieving more pronounced abrogation across the panel (p < 0.01). The close quantitative correspondence between protein secretion and mRNA expression data indicates that EGCG exerts its anti-inflammatory effects principally through transcriptional repression, consistent with inhibition of AP-1– and NF-κB–dependent gene programmes operating downstream of the PAR-2–ERK1/2 axis.

### EGCG attenuates the SOX4–ADAMTS-5 catabolic axis

LPS stimulation induced a 2.38-fold increase in SOX4 protein expression (Fig 5A, B), which was reduced by 41.1% and 81.9% upon treatment with 5 µM and 10 µM EGCG, respectively (p < 0.01 at 5 µM and *p* < 0.001 at 10 µM). RT-PCR demonstrated parallel reductions in SOX4 mRNA of 31.0% and 64.0% (Fig 5C), supporting transcriptional repression as a likely regulatory mechanism.

**Fig 5:**
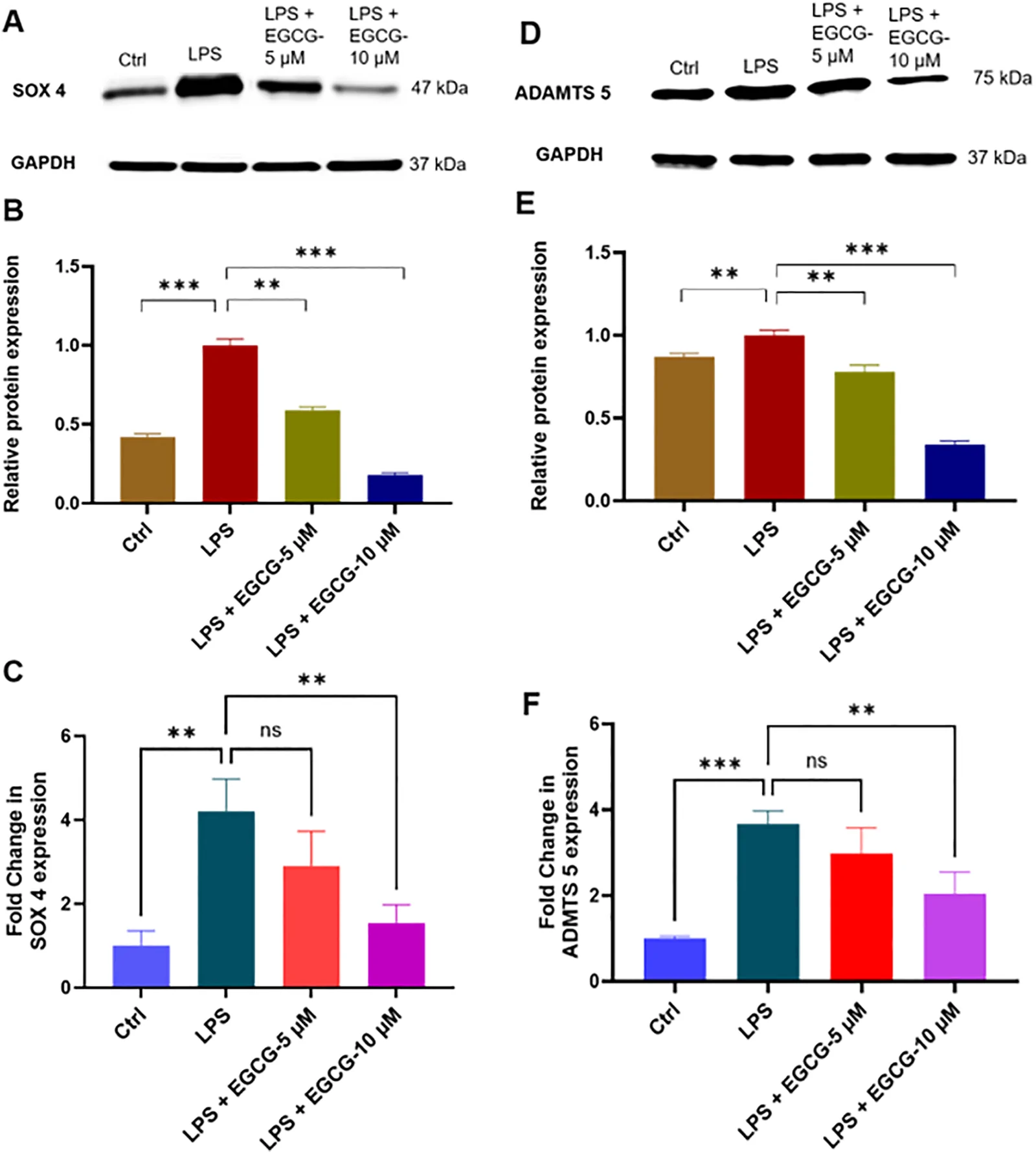
EGCG attenuates the SOX4–ADAMTS-5 catabolic transcription–protease axis at both protein and mRNA levels in LPS-stimulated chondrocytes. Cells were treated as described in Fig 2. SOX4, a transcription factor that drives ADAMTS-5 expression and cartilage catabolism in OA, and its downstream effector ADAMTS-5, the principal aggrecanase implicated in proteoglycan degradation, were assessed in parallel. (A) Representative western blot for SOX4 protein across all four treatment conditions, with GAPDH as loading control. (B) Densitometric quantification of SOX4 protein, normalized to GAPDH and expressed relative to the LPS-only group. (C) RT-PCR analysis of SOX4 mRNA, expressed as fold change relative to the untreated control. (D) Representative western blot for ADAMTS-5 protein, with GAPDH as loading control. Full-length blot images for panels (A) and (D) are provided in the Supplementary Data. (E) Densitometric quantification of ADAMTS-5 protein, normalized to GAPDH and expressed relative to the LPS-only group. (F) RT-PCR analysis of ADAMTS-5 mRNA, expressed as fold change relative to the untreated control. Data are presented as mean ± SD (n = 3 independent biological experiments). *p < 0.05, **p < 0.01, ***p < 0.001 versus LPS-only group (one-way ANOVA with Dunnett’s post-hoc test).

Downstream of SOX4, ADAMTS-5 protein was robustly upregulated by LPS (1.14-fold; Fig 5D, E) and suppressed by EGCG in a dose-dependent manner (22.0% and 66.2% reduction at 5 µM and 10 µM, respectively, with statistical significance of p<0.01 and p<0.001 at the corresponding concentrations). ADAMTS-5 mRNA was reduced by 46.0% at 10 µM EGCG (Fig 5F). The coordinate suppression of SOX4 and its transcriptional target ADAMTS-5 at both mRNA and protein levels supports a coherent mechanistic model in which EGCG may interrupt the coupling of inflammatory signaling to irreversible extracellular matrix degradation.

### EGCG suppresses RANK and RANKL surface expression

Cell surface expression of RANK and RANKL was evaluated using a sequential gating strategy (Supplementary S2 Fig), wherein debris and doublets were excluded prior to fluorescence intensity analysis against isotype controls. Flow cytometric analysis revealed low basal RANK surface expression in untreated chondrocytes (Fig 6A), with LPS inducing a robust rightward shift indicative of substantial upregulation (Fig 6B). EGCG co-treatment dose-dependently attenuated RANK expression, with 5 µM producing partial suppression (Fig 6C) and 10 µM nearly restoring the basal phenotype (Fig 6D). Quantitative analysis confirmed significant dose-dependent reductions in RANK-positive events (Fig 6E).

**Fig 6:**
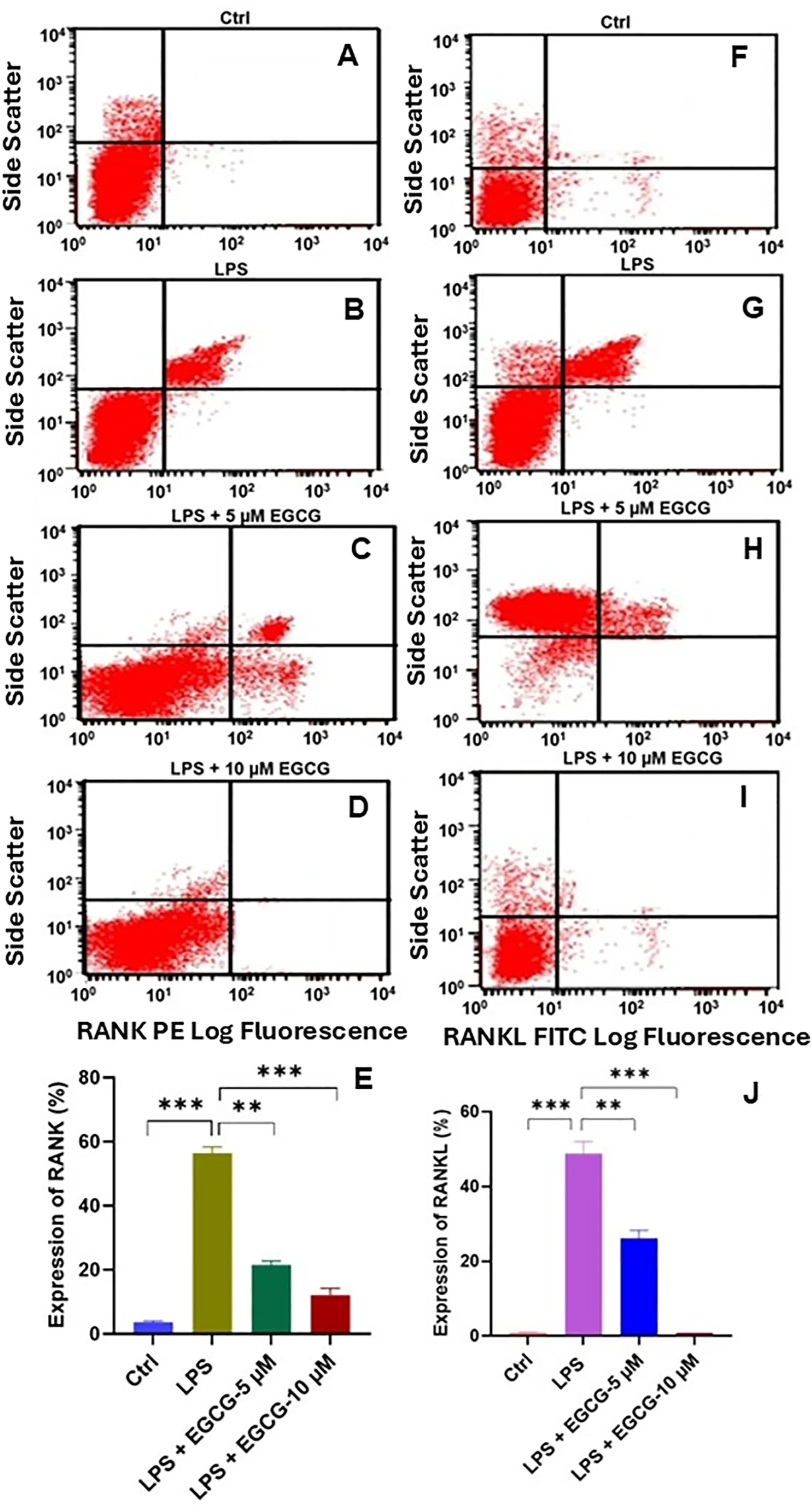
EGCG dose-dependently suppresses RANKL and RANK surface expression in LPS-stimulated chondrocytes, assessed by flow cytometry. Cells were treated as described in Fig 2 and then harvested and labelled with fluorophore-conjugated antibodies against surface RANK and RANKL prior to flow cytometric acquisition. Representative dot plots of side scatter against fluorescence intensity are shown for RANK (PE; panels A–D) and RANKL (FITC; panels F–I) under each treatment condition: (A, F) untreated control; (B, G) LPS (10 µg/mL); (C, H) LPS + 5 µM EGCG; (D, I) LPS + 10 µM EGCG. Quadrant thresholds were set against isotype controls. The representative gating strategy applied to all samples is shown in S2 Fig. Quantitative analysis of the percentage of RANK-positive events (E) and RANKL-positive events (J) across all four treatment conditions, derived from a minimum of 10,000 events acquired per sample. Data are presented as mean ± SD (n = 3 independent biological experiments). *p<0.05, **p<0.01, ***p<0.001 versus LPS-only group (one-way ANOVA with Dunnett’s post-hoc test).

An analogous pattern was observed for RANKL: minimal basal expression (Fig 6F) was markedly elevated by LPS (Fig 6G) and dose-dependently reversed by EGCG at 5 µM (Fig 6H) and 10 µM (Fig 6I), with the higher concentration recapitulating basal levels. Quantitative assessment confirmed statistically significant reductions across EGCG-treated groups (Fig 6J). The bilateral suppression of both RANK and RANKL suggests that EGCG may exert coordinated regulatory effects over this osteoimmunological signalling dyad, potentially disrupting the osteoclastogenic crosstalk that bridges cartilage degeneration and subchondral bone erosion in OA.

### EGCG restores mitochondrial membrane potential and biomass

Rhodamine 123 staining revealed strong, homogeneous fluorescence in untreated chondrocytes (Fig 7A), reflecting intact mitochondrial membrane potential (ΔΨm). LPS exposure markedly attenuated fluorescence intensity, consistent with mitochondrial depolarization (Fig 7B). EGCG treatment partially restored ΔΨm at 5 µM (Fig 7C) and achieved near-complete recovery at 10 µM (Fig 7D). Quantitative analysis of integrated fluorescence density confirmed statistically significant, dose-dependent restoration of ΔΨm (Fig 7E).

**Fig 7:**
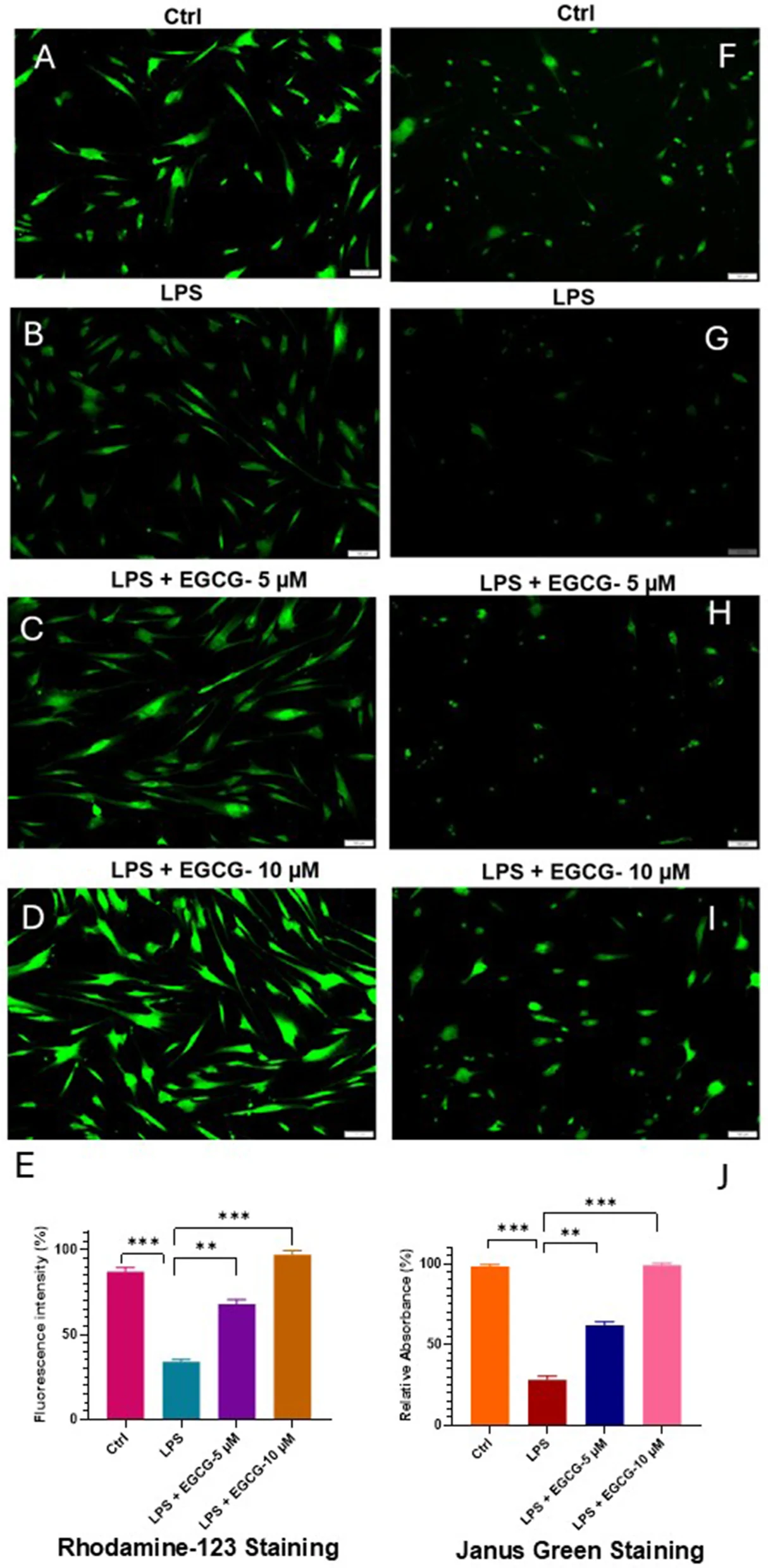
EGCG restores mitochondrial membrane potential and biomass. Two complementary stains are used: Rhodamine 123, whose fluorescence reports the mitochondrial membrane potential (ΔΨm), and Janus Green B, which reports mitochondrial mass; LPS reduces both, and EGCG restores them dose-dependently towards control levels. Rhodamine 123 fluorescence micrographs: (A) untreated control; (B) LPS; (C) LPS + 5 µM EGCG; (D) LPS + 10 µM EGCG. (E) Quantification of integrated fluorescence density reflecting mitochondrial membrane potential (ΔΨm). Janus Green B staining: (F) untreated control; (G) LPS; (H) LPS + 5 μM EGCG; (I) LPS + 10 μM EGCG. (J) Absorbance quantification (595 nm) of Janus Green B staining across treatment groups. Data are mean ± SD (n = 3). *p < 0.05, **p < 0.01, ***p < 0.001 vs. LPS-only group.

Janus Green staining independently corroborated these findings: LPS-treated chondrocytes exhibited markedly diminished mitochondrial staining relative to untreated controls (Figs 7F, G), consistent with inflammation-induced loss of mitochondrial content. EGCG treatment dose-dependently restored staining intensity, with 10 µM producing near-complete rescue (Figs 7H–J; *p* < 0.001 vs. LPS). The concordant preservation of both membrane potential and mitochondrial biomass indicates that EGCG preserves the structural (mitochondrial mass) and thermodynamic (membrane potential) markers of mitochondrial integrity under inflammatory stress, an effect mechanistically consonant with the observed suppression of the PAR-2–ERK1/2 axis, which has been implicated in mitochondrial destabilization through intracellular Ca^2+^ perturbations and aberrant mitophagy. Because functional bioenergetic assays were not performed, we do not infer a direct effect on respiratory capacity or ATP output.

### siRNA-mediated PAR-2 silencing supports PAR-2 involvement in EGCG’s anti-inflammatory action

To assess the mechanistic contribution of PAR-2 in EGCG-mediated anti-inflammatory signaling, we compared TNF-α responses in non-silenced and PAR-2–silenced chondrocytes across matched experimental conditions (conditions 1–4 vs. 5–8). Western blot analysis confirmed efficient and selective PAR-2 ablation in siRNA-transfected cells (lanes 5–8), with GAPDH expression remaining uniform across all conditions (Fig 8A). The efficiency of PAR-2 gene silencing and the relative expression levels across the experimental treatment groups were confirmed using full-length uncropped Western blot membranes (Supplementary S3 Fig).

**Fig 8:**
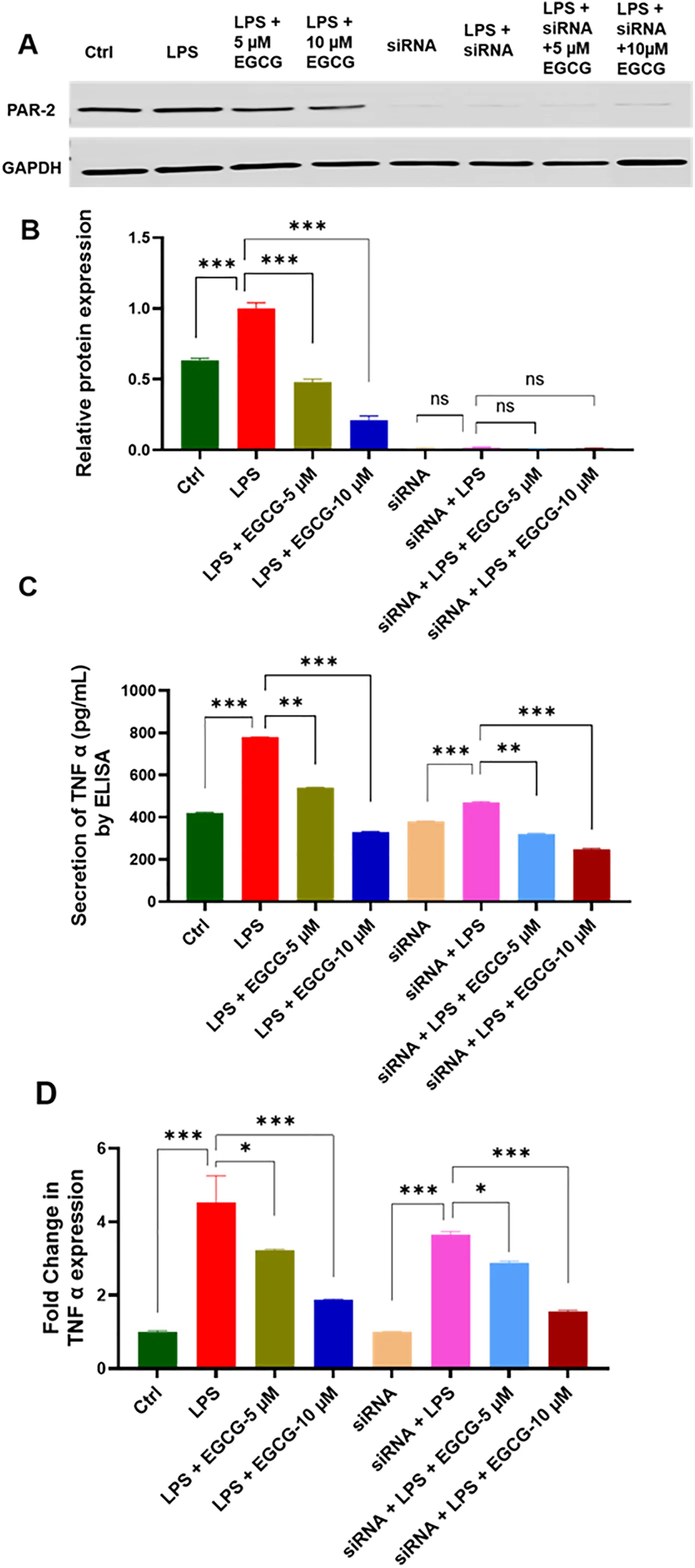
siRNA-mediated PAR-2 silencing supports PAR-2 involvement in EGCG’s anti-inflammatory action. Comparison of non-silenced with PAR-2–silenced chondrocytes shows that PAR-2 knockdown alone lowers LPS-induced TNF-α and markedly reduces the further suppression achieved by EGCG, supporting PAR-2 as a predominant but not exclusive mediator of EGCG. (A) Western blot confirming selective PAR-2 ablation in siRNA-transfected chondrocytes (conditions 5–8) with uniform GAPDH loading. PAR-2 was detected at approximately 44 kDa and GAPDH at approximately 37 kDa; the full-length, uncropped blot is provided in the Supplementary Data. (B) Densitometric quantification of relative PAR-2 protein expression normalized to GAPDH, demonstrating a dose-dependent reduction by EGCG under lipopolysaccharide (LPS) stimulation, and negligible expression in siRNA-transfected groups (ns = non-significant). (C) ELISA quantification of TNF-α secretion (pg/mL) in non-silenced and PAR-2–silenced chondrocytes. (D) RT-PCR analysis of TNF-α mRNA expression across matched conditions. Data are mean ± SD (n = 3). *p < 0.05, **p < 0.01, ***p < 0.001.

In non-silenced chondrocytes, LPS induced robust TNF-α secretion (~780 pg/mL; condition 2), which EGCG dose-dependently suppressed to ~500 pg/mL at 5 µM and ~350 pg/mL at 10 µM (Fig 8C). In PAR-2–silenced cells, LPS-induced TNF-α was substantially diminished (~480 pg/mL; condition 6), a ~ 38% reduction relative to the non-silenced LPS group, supporting PAR-2 as an important upstream contributor to cytokine production under inflammatory conditions. Critically, EGCG co-treatment in PAR-2–silenced cells yielded only modest additional suppression (~320 and ~240 pg/mL at 5 µM and 10 µM, respectively), with the incremental reduction markedly attenuated compared to the non-silenced arm. qPCR analysis (Fig 8D) corroborated these findings at the transcript level: LPS induced a ~ 4.5-fold increase in TNF-α mRNA in non-silenced cells versus ~3.6-fold in PAR-2–silenced cells, with EGCG-mediated suppression substantially blunted upon PAR-2 knockdown.

### An integrated signalling model summarises EGCG-associated modulation of the PAR-2 cascade across five effector modules

The experimental findings presented above are synthesised into a unified mechanistic model in Fig 9, which positions PAR-2 as the apical node through which EGCG coordinates its chondroprotective action and traces the consequences of receptor engagement through five functionally linked downstream effector modules. In the inflamed osteoarthritic joint, a TNF-α–rich proteolytic milieu drives activation of protease-activated receptor-2 (PAR-2, encoded by *F2RL1*) at the chondrocyte plasma membrane, whereas EGCG acts at the very summit of this cascade by downregulating PAR-2 expression and activation at both the protein and transcript levels (Fig 2). Because every downstream module depicted in the model is contingent on signal flux through this single receptor, suppression of PAR-2 by EGCG is propagated co-ordinately to all five effector arms, each of which was interrogated independently in the present study using a methodologically distinct readout. The model thus renders explicit how a single upstream intervention produces the concordant, pathway-wide attenuation documented across Figs 2–8 and frames the divergent cellular outcomes that follow from either unchecked or EGCG-restrained receptor signalling.

**Fig 9:**
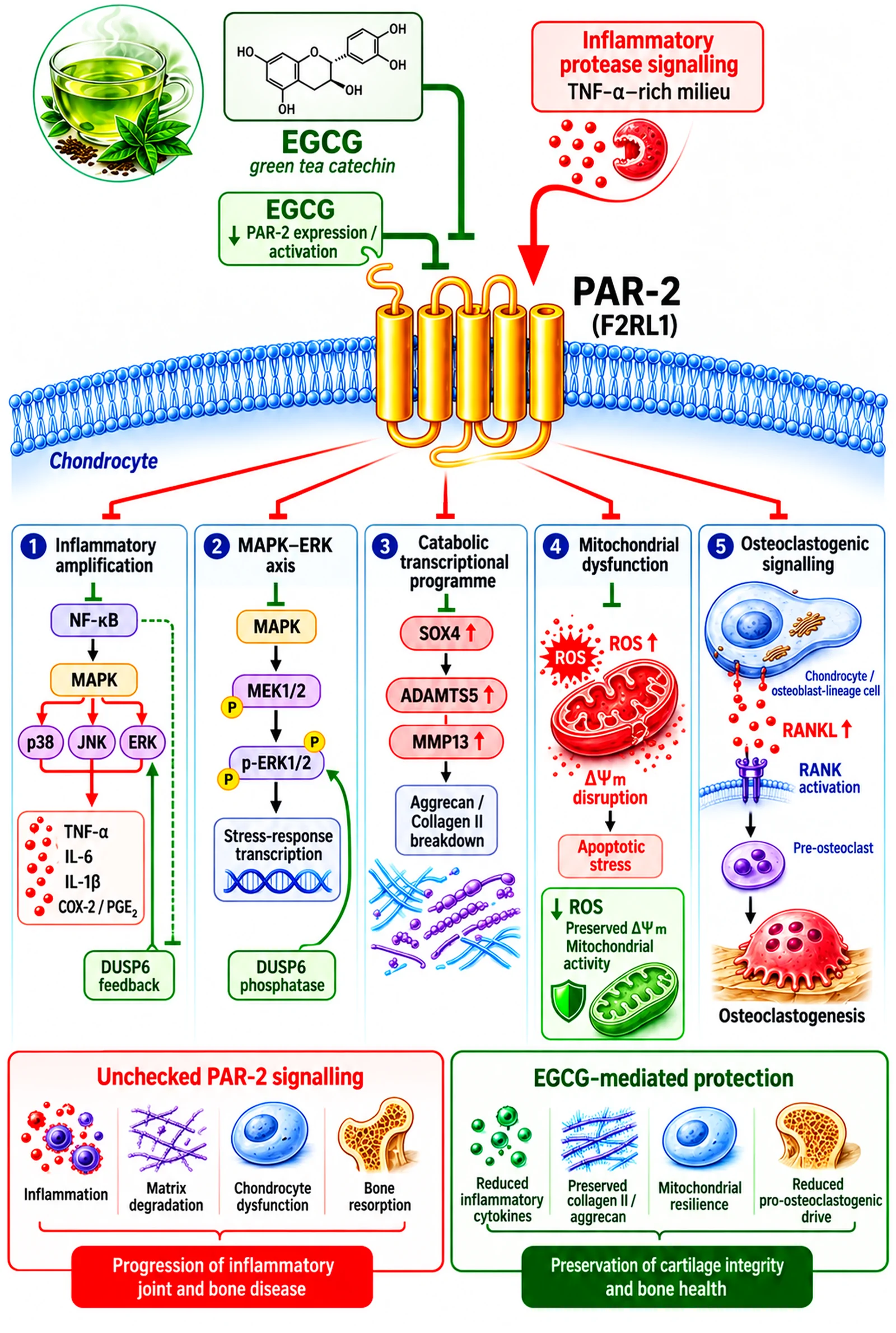
Integrated signalling model of EGCG’s chondroprotective mechanism in osteoarthritic chondrocytes. Schematic synthesis integrating the experimental findings of this study into a unified, PAR-2–centred framework. In the inflamed joint, a TNF-α–rich proteolytic milieu activates protease-activated receptor-2 (PAR-2; gene F2RL1) at the chondrocyte surface, while EGCG acts at the apex of the cascade by downregulating PAR-2 expression and activation. Five functionally linked downstream modules are depicted: (1) inflammatory amplification, in which NF-κB and the p38/JNK/ERK MAPK arms drive induction of TNF-α, IL-6, IL-1β, and COX-2/PGE2 under DUSP6-mediated negative feedback; (2) the MAPK–ERK axis, in which MEK1/2-dependent ERK1/2 phosphorylation licenses stress-response transcription and is counter-regulated by the DUSP6 phosphatase; (3) the catabolic transcriptional programme, in which SOX4 drives ADAMTS-5 and MMP-13 expression and consequent aggrecan/collagen II breakdown; (4) mitochondrial dysfunction, comprising ROS accumulation, ΔΨm disruption, and apoptotic stress; and (5) osteoclastogenic signalling, in which RANKL/RANK upregulation promotes pre-osteoclast commitment and osteoclastogenesis. The lower panels contrast the two divergent trajectories: unchecked PAR-2 signalling (inflammation, matrix degradation, chondrocyte dysfunction, and bone resorption) drives progression of inflammatory joint and bone disease, whereas EGCG-mediated suppression (reduced inflammatory cytokines, preserved collagen II/aggrecan, mitochondrial resilience, and reduced pro-osteoclastogenic drive) favours preservation of cartilage integrity and bone health. Green elements denote EGCG-mediated downregulation and protection; red elements denote PAR-2–driven inflammatory, catabolic, mitochondrial, and osteoclastogenic signalling. Created in BioRender. Banerjee, Y. (2026), Publication license available at https://BioRender.com/o26w5ik.

The first module (Fig 9, module 1) depicts inflammatory amplification, the transcriptional limb through which receptor engagement is converted into a self-reinforcing cytokine output. PAR-2 activation converges on the NF-κB and mitogen-activated protein kinase (MAPK) signalling nodes, the latter resolving into the canonical triad of p38, JNK, and ERK that together drive the pro-inflammatory and eicosanoid programme represented in the model by TNF-α, IL-6, IL-1β, and COX-2/PGE2. Of these effectors, three mediators were quantified directly in this study, namely TNF-α, IL-1β, and MCP-1, each of which was suppressed by EGCG in a concentration-dependent manner at both protein and transcript level (Fig 4), supporting an association between PAR-2-axis modulation and the secreted inflammatory output. The module additionally incorporates *DUSP6* as the inducible dual-specificity phosphatase that constitutes the principal negative-feedback brake on this circuit; its LPS-driven induction and EGCG-sensitive attenuation were measured directly (Fig 3E) and are positioned in the model as the endogenous counter-regulatory element of the amplification loop. The p38, JNK, and NF-κB nodes, together with COX-2/PGE2, are rendered as the established architecture of the module rather than as endpoints assayed here; the ERK arm, by contrast, was singled out for direct mechanistic dissection, as detailed in the second module.

The second module (Fig 9, module 2) isolates the MAPK-ERK axis, the specific kinase limb through which the present study traced signal transmission from receptor to nucleus. PAR-2–driven MAPK activity is relayed through MEK1/2 to the phosphorylation of ERK1/2, which in turn licenses stress-response transcription, while the same *DUSP6* phosphatase that brakes the amplification loop is depicted here as the proximal negative regulator of ERK activation. This axis was characterised at the highest mechanistic resolution of any module: EGCG suppressed ERK1/2 phosphorylation and reduced total ERK1/2 abundance, with the activation-state-specific pERK1/2:total ERK1/2 ratio driven below basal levels at 10 µM (Fig 3A–D), while the ERK1 and ERK2 transcripts were concordantly downregulated (Fig 3F) and DUSP6 was attenuated (Fig 3E). The sub-basal restoration of the phosphorylation ratio, depicted in the model as the pivotal control point downstream of PAR-2, indicates preferential inhibition of kinase activation beyond the reduction in kinase abundance, consistent with combined upstream interference and preserved phosphatase tone. MEK1/2 is represented as the canonical immediate-upstream activator of ERK1/2 rather than as a node assayed directly in this work.

The third module (Fig 9, module 3) represents the catabolic transcriptional programme that couples inflammatory signalling to irreversible matrix destruction. The transcription factor SOX4 is positioned upstream of the aggrecanase and collagenase effectors ADAMTS-5 and MMP-13, whose combined activity culminates in the depicted breakdown of aggrecan and type II collagen, the two principal load-bearing constituents of the cartilage extracellular matrix. The SOX4–ADAMTS-5 limb of this programme was quantified directly; EGCG produced coordinate, concentration-dependent suppression of both SOX4 and ADAMTS-5 at protein and transcript level (Fig 5), interrupting the transcription-factor-to-protease coupling at its origin. MMP-13 is incorporated in the model as the canonical co-effector collagenase that acts in concert with ADAMTS-5 to complete matrix catabolism, and the aggrecan/collagen II breakdown shown as the terminus of the module represents the functional consequence inferred from this protease output rather than an endpoint measured here. The paired matrix insets contrast the degraded fibrillar architecture that accompanies unchecked catabolism with the preserved matrix retained under EGCG, encapsulating the structural stakes of this arm of the cascade.

The fourth module (Fig 9, module 4) depicts the mitochondrial dimension of PAR-2–driven injury, in which receptor signalling promotes the accumulation of reactive oxygen species (ROS), disruption of the mitochondrial membrane potential (ΔΨm), and consequent apoptotic stress. The two readouts that anchor this module empirically are the thermodynamic and structural markers of mitochondrial integrity, ΔΨm, reported by Rhodamine 123 fluorescence, and mitochondrial mass, reported by Janus Green B staining, both of which were diminished by LPS and restored towards homeostatic levels by EGCG in a concentration-dependent manner (Fig 7). Consistent with the deliberately circumscribed scope of these assays, ROS accumulation and apoptotic stress are rendered in the model as the established upstream cause and downstream consequence of membrane-potential collapse rather than as parameters measured here, and the EGCG-protection inset depicts the restitution of mitochondrial integrity that accompanies receptor suppression. Positioning this module alongside the inflammatory and catabolic arms makes explicit that EGCG-mediated PAR-2 interception spares the mitochondrial compartment in parallel with its suppression of nuclear inflammatory programmes.

The fifth module (Fig 9, module 5) traces the osteoclastogenic limb that links inflamed cartilage to the subchondral bone compartment. PAR-2 activation upregulates RANKL on the chondrocyte/osteoblast-lineage cell, engaging RANK to drive pre-osteoclast commitment and, ultimately, osteoclastogenesis. The proximal, chondrocyte-resident events of this axis were measured directly by flow cytometry: EGCG bilaterally suppressed both RANKL and RANK surface expression in a concentration-dependent fashion (Fig 6), curtailing the osteoimmunological signal at its source. The pre-osteoclast and osteoclastogenesis stages shown downstream represent the established cellular consequence of sustained RANKL-RANK engagement and are depicted to situate the measured surface-receptor changes within their pathophysiological trajectory rather than as endpoints assayed in chondrocyte monoculture. This module thereby connects the receptor-level intervention characterised here to the cartilage–bone crosstalk that underlies structural progression in osteoarthritis.

The lower panels of Fig 9 integrate the five modules into the two divergent cellular trajectories that the model is constructed to contrast. Under unchecked PAR-2 signalling, the combined output of the modules, inflammation, matrix degradation, chondrocyte dysfunction, and bone resorption, converges on the progression of inflammatory joint and bone disease. Under EGCG-mediated suppression, the mirror-image outcome obtains, reduced inflammatory cytokines, preserved type II collagen and aggrecan, mitochondrial resilience, and a reduced pro-osteoclastogenic drive together favour the preservation of cartilage integrity and bone health. The convergence of five independently measured effector arms onto a single upstream receptor, all concordantly reversed by EGCG, is the central relationship that the model formalises; involvement of that receptor is supported quantitatively by the siRNA silencing data (Fig 8), which identify PAR-2 as the predominant, though not exclusive, node through which EGCG exerts its effect. Throughout the schematic, elements rendered in green denote EGCG-mediated downregulation and protection, whereas elements in red denote the inflammatory, catabolic, mitochondrial, and osteoclastogenic signalling that proceeds when the receptor is left unrestrained.

## Discussion

### Evidence supports PAR-2 as a principal upstream mediator of EGCG action in osteoarthritic chondrocytes

The present study supports EGCG as a potential modulator of PAR-2–mediated inflammatory signaling in osteoarthritic chondrocytes, extending prior observations of its generic anti-inflammatory activity [35,36], to a defined receptor–kinase axis of established pathogenic significance. The principal finding, that EGCG dose-dependently suppresses PAR-2 at both mRNA and protein levels, with consequent attenuation of ERK1/2 phosphorylation, pro-inflammatory cytokine secretion, catabolic enzyme expression, osteoclastogenic signaling, and mitochondrial depolarization (summarized in Fig 9), supports a coherent upstream-to-downstream inhibitory model that has not, to our knowledge, been previously reported for any dietary polyphenol in OA chondrocytes.

PAR-2 is a G-protein-coupled receptor that senses extracellular proteases and amplifies inflammation through MAPK and NF-κB effectors [11,12]. Its expression is elevated in OA cartilage and correlates with disease severity [11,17]. The mechanistic involvement of PAR-2 in our model is supported by the siRNA silencing experiments (Fig 8): PAR-2 knockdown alone reduced LPS-induced TNF-α secretion by ~38% and substantially blunted the incremental efficacy of EGCG co-treatment. That EGCG retained residual suppressive activity in PAR-2–silenced cells indicates that PAR-2 constitutes the predominant, though not exclusive, locus of EGCG action, consistent with parallel modulation of NF-κB or mTOR signalling as reported elsewhere [10,35].

Together, these loss-of-function and concentration–response data establish PAR-2 as a critical mediator of LPS-induced TNF-α output and show that EGCG acts predominantly, though not exclusively, through it; the PAR-2–independent axes that account for its residual activity are catalogued in Table 2.

**Table 2:** PAR-2-independent signalling pathways modulated by EGCG with OA pathobiological relevance.

Signalling Pathway	Primary Molecular Target(s) of EGCG	OA-Relevant Downstream Effects	Basis for Independence from PAR-2	Key References
NF-κB / IKKβ Axis	Direct allosteric inhibition of IKKβ (IκB kinase β), the catalytic subunit responsible for IκBα Ser32/Ser36 phosphorylation and proteasomal targeting. EGCG prevents IκBα degradation and consequent NF-κB p65 nuclear translocation.	Suppresses NF-κB-driven transcription of TNF-α, IL-1β, IL-6, MCP-1, COX-2, iNOS, MMP-1, MMP-3, MMP-13, ADAMTS-4, and ADAMTS-5, the complete OA catabolic and inflammatory effector panel documented in human OA chondrocytes.	IKKβ inhibition by EGCG is mechanistically upstream of, and independent of, PAR-2’s capacity to activate NF-κB; EGCG intercepts NF-κB at the kinase level regardless of which upstream receptor initiated the cascade.	[35,37]
AP-1 (JNK–c-Jun / c-Fos) Axis	Selective inhibition of JNK1/2 phosphorylation (Thr183/Tyr185) and downstream c-Jun phosphorylation at Ser63/Ser73, preventing assembly of the AP-1 transcriptional complex at MMP and cytokine gene promoters.	Reduces AP-1-driven transcription of MMP-1, MMP-3, MMP-13, COX-2, IL-6, and MCP-1. Inhibition of JNK-MAPK by EGCG in human OA chondrocytes has been directly demonstrated, with consequent suppression of aggrecanase and collagenase-mediated cartilage catabolism.	AP-1 is a convergence point for multiple upstream kinase cascades (JNK, p38, ERK). EGCG’s suppression of AP-1 via JNK inhibition is demonstrable in PAR-2-naïve cellular systems and does not require prior PAR-2 GPCR activation.	[35,37]
Nrf2–Keap1–ARE Antioxidant / Mitoprotective Axis	Promotion of Nrf2 nuclear translocation via Keap1 dissociation (at Cys151/Cys273), enabling ARE-driven transcription of HO-1, NQO1, glutamate-cysteine ligase, thioredoxin reductase, and ferritin.	Reduces mitochondrial ROS, preserves mitochondrial membrane potential (ΔΨm), and prevents mPTP opening. Directly parallels the mitoprotective findings of Fig 7 through a PAR-2-independent route.	Nrf2 activation by EGCG is a direct chemical interaction at the Keap1 sensor domain, entirely upstream of and independent of GPCR-mediated signalling. Operative in cells lacking PAR-2 expression; provides the most parsimonious explanation for residual ΔΨm recovery in PAR-2-silenced chondrocytes.	[35,37]
PI3K–Akt–mTORC1 Axis	Inhibition of class I PI3K catalytic subunits (p110α and p110δ), reducing PtdIns(3,4,5)P3 generation and attenuating PDK1-mediated Akt(Thr308) and mTORC2-mediated Akt(Ser473) phosphorylation, suppressing downstream mTORC1 (S6K1, 4E-BP1) activity.	Reduces Akt-driven IKKβ co-activation and mTORC1-mediated translational upregulation of pro-inflammatory proteins. Enhances ULK1-mediated autophagic mitochondrial quality control (mitophagy), preserving chondrocyte energetic homeostasis.	PI3K catalytic inhibition by EGCG targets receptor-tyrosine-kinase-coupled Class Ia PI3K isoforms directly, without requiring GPCR input, constituting a PAR-2-independent route to NF-κB and mTOR suppression.	[35,37]
JAK1/2–STAT3 Axis	Suppression of constitutive and IL-6-induced STAT3 phosphorylation at Tyr705 (JAK1/2-mediated) and Ser727 (ERK/p38-mediated), preventing STAT3 homodimerisation and nuclear translocation to TTCN2–4GAA response elements.	Reduces STAT3-driven transcription of VEGF, survivin, Bcl-xL, MMP-1, and IL-6 (STAT3 auto-inductive target). In OA chondrocytes, STAT3 activation drives catabolic gene expression and cartilage degradation; its suppression by EGCG provides a parallel anti-catabolic contribution.	JAK–STAT3 signalling is activated principally by IL-6R/gp130, IFN receptors, and growth factor receptors — a receptor family entirely distinct from GPCRs including PAR-2. EGCG’s JAK/STAT3-suppressive activity is demonstrable in cell systems without functional PAR-2.	[35,37]
AMPK (AMP-activated Protein Kinase) / Metabolic-Sensing Axis	AMPK activation (via transient Complex I perturbation and elevated AMP:ATP ratio), phosphorylating TSC2(Ser1387) and Raptor(Ser792) to suppress mTORC1, and attenuating IKKβ-NF-κB through energy-sensing mechanisms.	Reduces mTOR-driven pro-inflammatory protein synthesis; suppresses NF-κB through an energy-sensing-independent parallel route. In OA chondrocytes, AMPK deficiency accelerates cartilage degradation; pharmacological AMPK activation reduces MMP and ADAMTS expression. AMPK activates ULK1, promoting mitophagic clearance of depolarised mitochondria — consistent with the ΔΨm recovery in Fig 7.	AMPK activation by EGCG is a metabolic-sensing, receptor-independent mechanism operating entirely outside the GPCR–PAR-2 axis. Provides the most parsimonious explanation for near-complete ΔΨm rescue at 10 µM EGCG in PAR-2-silenced chondrocytes.	[35,37]

(Note: AMPK, AMP-activated protein kinase; AP-1, activator protein-1; ARE, antioxidant response element; GPCR, G-protein-coupled receptor; HO-1, haem oxygenase-1; IKKβ, IκB kinase β; JNK, c-Jun N-terminal kinase; mPTP, mitochondrial permeability transition pore; mTORC1, mechanistic target of rapamycin complex 1; NF-κB, nuclear factor kappa-B; NQO1, NAD(P)H quinone oxidoreductase 1; Nrf2, nuclear factor erythroid 2-related factor 2; OA, osteoarthritis; PAR-2, protease-activated receptor-2; PI3K, phosphoinositide 3-kinase; ROS, reactive oxygen species; STAT3, signal transducer and activator of transcription 3; ΔΨm, mitochondrial membrane potential.)

This residual activity is mechanistically informative rather than paradoxical, and is consistent with the polypharmacological profile of EGCG. Singh et al. documented EGCG’s inhibitory effects on NF-κB, JNK-MAPK, and AP-1 in arthritis-relevant systems [35]; and Akhtar and Haqqi showed that EGCG suppresses TNF-α, IL-6, MCP-1, and the MMP/ADAMTS panel through NF-κB and JNK-MAPK inhibition in primary human OA chondrocytes [37]. The Nrf2–Keap1–ARE and AMPK axes, together with the remaining PAR-2–independent pathways, are catalogued in Table 2. The PAR-2 axis remains the quantitatively predominant pathway, accounting for ~38% of LPS-induced TNF-α production as supported by the siRNA silencing data (Fig 8).

Three OA-relevant, PAR-2–independent axes are the most directly evidenced. The first is the NF-κB–JNK/AP-1 inflammatory module [35,37]. The second is the cytoprotective Nrf2–Keap1–ARE antioxidant response: in interleukin-1β-challenged human chondrocytes EGCG upregulates Nrf2, haem oxygenase-1, and NQO1, reduces reactive oxygen species, and limits chondrocyte apoptosis and senescence, effects abolished by the Nrf2 inhibitor ML385 [38]. The third is restoration of autophagic flux, where intra-articular EGCG attenuates cartilage degeneration in a post-traumatic OA model by enhancing chondrocyte autophagy through suppression of mTOR and induction of LC3, beclin-1, and reduced p62 [39]. Together, these three axes plausibly account for the ~ 62% of LPS-induced TNF-α output that persists after PAR-2 knockdown. Beyond these axes, EGCG also modulates canonical Wnt/β-catenin signalling, promoting β-catenin degradation independently of GSK-3β and PP2A [40], a credible but as-yet-unexamined node of EGCG action in cartilage. We emphasize that several of these effects derive from single-pathway studies and that the redox-active structure of EGCG can confound in vitro readouts [41]; this survey provides a plausible, multi-pathway framework rather than a definitive accounting.

### Coordinated suppression of downstream inflammatory, catabolic, and osteoclastogenic effectors

Downstream of PAR-2–ERK1/2 inhibition, EGCG produced coordinated suppression of three functionally linked effector arms. First, ELISA and qPCR data (Fig 4) revealed concentration-dependent repression of TNF-α, IL-1β, and MCP-1 at both protein and transcript levels, with near-maximal target engagement at 10 µM, and a clear saturation plateau between 10 and 20 µM. The close correspondence between protein secretion and mRNA expression implicates transcriptional repression, likely via AP-1 and NF-κB–dependent promoters, as the primary regulatory mechanism [42]. Second, EGCG attenuated the SOX4–ADAMTS-5 catabolic axis (Fig 5), reducing SOX4 protein by 81.9% and ADAMTS-5 by 66.2% at 10 µM. Given that SOX4 directly transactivates aggrecanase genes [43], this coordinate suppression indicates that EGCG interrupts the coupling of inflammatory signalling to irreversible extracellular matrix degradation. Third, flow cytometric analysis demonstrated bilateral suppression of both RANK and RANKL surface expression (Fig 6), suggesting that EGCG may attenuate the osteoclastogenic crosstalk between inflamed cartilage and subchondral bone that drives structural progression in OA [44].

### Preservation of mitochondrial integrity

An often-underappreciated dimension of OA pathobiology is mitochondrial dysfunction, which both results from and potentiates inflammatory cartilage damage [45]. EGCG strikingly restored mitochondrial membrane potential (ΔΨm) and biomass in LPS-challenged chondrocytes (Fig 7). The tight coupling between these improvements and PAR-2–ERK suppression is mechanistically coherent; PAR-2 activation precipitates intracellular calcium perturbations and ERK-mediated oxidative stress that compromise mitochondrial integrity [18]. By intercepting this signalling at the receptor level, EGCG simultaneously curtails nuclear inflammatory gene programmes and spares the mitochondrial compartment, a dual action of relevance in avascular cartilage, where chondrocytes depend critically on efficient oxidative phosphorylation for matrix synthesis and survival.

Additionally, the ΔΨm recovery and mitochondrial mass preservation observed in the present study through Rhodamine 123 and Janus Green B staining represent the primary thermodynamic (membrane potential) and structural (mitochondrial mass) markers of mitochondrial integrity, rather than direct measures of bioenergetic function and are consistent with a growing body of published evidence characterising EGCG as a multimechanistic mitoprotective agent in other cell systems. At the level of mitochondrial reactive oxygen species (ROS), EGCG suppresses oxidative stress through two convergent mechanisms: (i) activation of the Nrf2/ARE pathway, with induction of haem oxygenase-1 (HO-1), NAD(P)H quinone oxidoreductase-1 (NQO1), and glutamate-cysteine ligase, thereby reinforcing glutathione-dependent antioxidant buffering [46]; and (ii) suppression of ERK1/2-associated NADPH oxidase signalling, a major upstream source of cellular superoxide in LPS-stimulated inflammatory cells [47]. Because ΔΨm is the principal driving force for F_1_F_0_-ATP synthase, the near-homeostatic ΔΨm recovery we document (Fig 7) would be expected to favour, but does not by itself demonstrate, oxidative-phosphorylation–coupled ATP synthesis, consistent with evidence that EGCG preserves ATP production via AMPK activation and mTORC1 suppression [10,48]. In OA chondrocytes specifically, where avascular tissue architecture renders cells critically dependent on efficient oxidative phosphorylation for extracellular matrix synthesis and survival, the functional significance of ΔΨm restoration extends beyond a bioenergetic readout, depolarisation of the inner membrane below a threshold ΔΨm drives mPTP opening and cytochrome c release, initiating the intrinsic apoptotic cascade that contributes to chondrocyte loss in OA [45,49]. The near-complete ΔΨm recovery at 10 µM EGCG (Fig 7D–E) is therefore consistent with, though it does not directly establish, simultaneous protection against chondrocyte apoptosis, ATP depletion, and oxidative stress, a trifecta of mitochondrial consequences that drive OA cartilage degeneration.

### Physiological and pharmacokinetic considerations

A recurrent criticism of polyphenol studies is the use of supraphysiological concentrations. We specifically addressed this concern through Table 3, which catalogues EGCG content across 36 commercially consumed green tea varieties. As catalogued there, across every variety the EGCG dose delivered to the gastrointestinal lumen exceeds the 5–10 µM concentrations used here by between one-and-a-half and two-and-a-half orders of magnitude. We emphasise that these values represent the concentration presented to the intestinal lumen and mucosa rather than the systemic plasma concentration; because only a small fraction of luminal catechin is absorbed intact, the limiting step in translation is intestinal absorption and first-pass metabolism, not dietary intake [50]. Even accounting for the well-documented attenuation of oral bioavailability (rapid glucuronidation, short plasma half-life [22]), the margin between dietary intake and effective concentration appears substantial, supporting the translational plausibility of our findings, while systemic delivery remains the key constraint. Moreover, EGCG’s efficacy aligns with our group’s prior demonstrations that curcumin [17,51], oleocanthal [18] and the endogenous lipid palmitoylcarnitine [52] similarly suppress the PAR-2 axis in chondrocytes, reinforcing PAR-2 as a tractable nutraceutical target and a common pharmacological node for structurally unrelated polyphenols and an endogenous lipid.

**Table 3:** Variation in EGCG content and bio-accessible dose among commonly consumed green teas.

Green Tea Type	Geographical Area of Origin	Processing Method / Cultivar	EGCG Content in Dry Leaf (mg/g)	EGCG per Serving (mg) (based on 2g dry tea or powder)	Serving Volume (mL)	EGCG Concentration per Serving (µM)	Ref	Remarks on EGCG Measurement & Brewing
**General Green Tea (Infusion)**	Global	Various	N/A	165	240	1499.0	[53]	General average for brewed green tea.
**General Green Tea (Infusion)**	Global	Various	N/A	90	200	981.7	[54]	Based on 2.5g tea leaves per 200mL water.
**General Green Tea (Dry Leaves)**	Global	Various	73.8	147.6	N/A	N/A	[53]	EGCG content in dried leaves, not brewed.
**Matcha**	Japan	Powdered, Shade-grown	50.5–56.6 (avg. ceremonial: 56.6; culinary: 50.5)	101–113	100	~2204–2465	[55]	Based on 2g powder per cup. Consuming whole leaf.
**Gyokuro**	Japan	Shade-grown	53.31	106.62	240	969.4	[56]	EGCG in dry leaf.
**Gyokuro (Infusion)**	Japan	Shade-grown	N/A	268.09	240	2437.0	[56]	Infusion from 10g leaves in 60mL water at 60°C for 2 min. Highly concentrated.
**Sencha (Superior)**	Japan	Sun-grown	67.45	134.90	240	1226.2	[56]	EGCG in dry leaf.
**Sencha (Superior Infusion)**	Japan	Sun-grown	N/A	74.41	240	676.0	[56]	Infusion from 6g leaves in 170mL water at 70°C for 1 min.
**Sencha (Standard)**	Japan	Sun-grown	62.16	124.32	240	1131.0	[56]	EGCG in dry leaf.
**Sencha (Standard Infusion)**	Japan	Sun-grown	N/A	91.30	240	830.0	[56]	Infusion from 6g leaves in 260mL water at 90°C for 1 min.
**Sencha (Deep-Steamed)**	Japan	Sun-grown, Deep-steamed	63.51	127.02	240	1155.0	[56]	EGCG in dry leaf.
**Sencha (Deep-Steamed Infusion)**	Japan	Sun-grown, Deep-steamed	N/A	114.20	240	1038.0	[56]	Infusion from 6g leaves in 260mL water at 90°C for 1 min.
**Sencha (Infusion)**	Japan	Sun-grown	N/A	124 mg/100mL	100	2705.4	[57]	EGCG content per 100mL of infusion.
**Tamaryokucha (Pan-Fired)**	Japan	Pan-fired	64.29	128.58	240	1168.0	[56]	EGCG in dry leaf.
**Tamaryokucha (Pan-Fired Infusion)**	Japan	Pan-fired	N/A	65.72	240	597.1	[56]	Infusion from 6g leaves in 260mL water at 90°C for 1 min.
**Tamaryokucha (Steamed)**	Japan	Steamed	61.61	123.22	240	1120.0	[56]	EGCG in dry leaf.
**Tamaryokucha (Steamed Infusion)**	Japan	Steamed	N/A	90.00	240	818.0	[56]	Infusion from 6g leaves in 260mL water at 90°C for 1 min.
**Lu’an Guapian (HSGP)**	Anhui, China (Huoshan County)	Traditional processing	110.69	221.38	240	2013.7	[58]	EGCG in dry leaf (WT%).
**Lu’an Guapian (JZGP)**	Anhui, China (Jinzhai County)	Traditional processing	87.18	174.36	240	1585.5	[58]	EGCG in dry leaf (WT%).
**Lu’an Guapian (YAGP)**	Anhui, China (Yu’an District)	Traditional processing	80.23	160.46	240	1459.7	[58]	EGCG in dry leaf (WT%).
**Lu’an Guapian (IMGP)**	Anhui, China (Inner Mountain)	Traditional processing	92.75	185.50	240	1687.2	[58]	EGCG in dry leaf (WT%).
**Lu’an Guapian (OMGP)**	Anhui, China (Outer Mountain)	Traditional processing	79.33	158.66	240	1443.5	[58]	EGCG in dry leaf (WT%).
**Korean Green Tea (Woojeon)**	Korea	Early plucking period	105.37	210.74	240	1916.6	[59]	EGCG in dry leaf.
**Korean Green Tea (Sejak)**	Korea	Mid plucking period	103.95	207.90	240	1890.3	[59]	EGCG in dry leaf.
**Korean Green Tea (Joongjak)**	Korea	Late plucking period	111.59	223.18	240	2030.0	[59]	EGCG in dry leaf.
**Korean Green Tea (Daejak)**	Korea	Latest plucking period	112.86	225.72	240	2053.3	[59]	EGCG in dry leaf.
**Jeju Green Tea (Steamed)**	Jeju, Korea	Steaming	24.0 (extract)	48.0 (extracted)	240	436.0	[60]	EGCG extracted from dry tea into infusion (mg/g dry tea). Assumed 2g dry tea serving.
**Jeju Green Tea (Pan-fired)**	Jeju, Korea	Pan-firing	31.8 (extract)	63.6 (extracted)	240	578.0	[60]	EGCG extracted from dry tea into infusion (mg/g dry tea). Assumed 2g dry tea serving.
**Jeju Green Tea (Steamed & Pan-fired, Light)**	Jeju, Korea	Steaming & Pan-firing	20.2 (extract)	40.4 (extracted)	240	367.0	[60]	EGCG extracted from dry tea into infusion (mg/g dry tea). Assumed 2g dry tea serving.
**Jeju Green Tea (Steamed & Pan-fired, Heavy Roast)**	Jeju, Korea	Steaming & Pan-firing, Heavy roasting	42.3 (extract)	84.6 (extracted)	240	769.0	[60]	EGCG extracted from dry tea into infusion (mg/g dry tea). Assumed 2g dry tea serving.
**Boseong Green Tea (Pan-fired)**	Boseong, Korea	Pan-firing	39.9 (extract)	79.8 (extracted)	240	725.0	[60]	EGCG extracted from dry tea into infusion (mg/g dry tea). Assumed 2g dry tea serving.
**Hangzhou Green Tea (Pan-fired)**	Hangzhou, China	Pan-firing	36.9 (extract)	73.8 (extracted)	240	671.0	[60]	EGCG extracted from dry tea into infusion (mg/g dry tea). Assumed 2g dry tea serving.
**Shizuoka Green Tea (Steamed)**	Shizuoka, Japan	Steaming	24.5 (extract)	49.0 (extracted)	240	445.0	[60]	EGCG extracted from dry tea into infusion (mg/g dry tea). Assumed 2g dry tea serving.
**Longjing (Dragon Well)**	Hangzhou, Zhejiang, China	Pan-fired	Variable	Variable	240	Variable	[61]	Known for high thiamine; EGCG present but specific values per serving not consistently provided.
**Biluochun (Green Snail Spring)**	Jiangsu, China	Traditional processing	Variable	Variable	240	Variable	[61]	Rich in polyphenols, high antioxidant level. Specific EGCG per serving not provided.
**Huangshan Maofeng**	Anhui, China	Traditional processing	Variable	Variable	240	Variable	[61]	Rich in antioxidants, including EGCG, but specific values per serving not provided.

Additionally, a rigorous contextualisation of the in vitro concentrations employed in this study within the pharmacokinetic reality of oral EGCG administration is warranted, and the translational implications of this gap require transparent discussion. Following oral consumption, EGCG is absorbed across the small intestinal epithelium with inherently limited efficiency; the landmark human pharmacokinetic study by Lee et al. [62] established that a single oral dose of green tea (~2 mg EGCG/kg) yields a peak plasma concentration (Cmax) of only 77.9 ± 22.2 ng/mL in healthy subjects, corresponding to approximately 0.17 µM total (free plus conjugated) EGCG. Even at the high supplementary doses examined in the multiple-dose study of Chow et al. [22], plasma concentrations remain in the low micromolar range, and published pharmacokinetic modelling consistently indicates that free, unconjugated EGCG exceeds 1 µM systemically only at intake doses of ≥1 g, substantially above what is achievable from a single cup of green tea, irrespective of variety. The principal barriers to systemic bioavailability are threefold: (i) physicochemical instability of the catechol and pyrogallol moieties under alkaline intestinal conditions and active apical efflux at the enterocyte brush border, both of which reduce net absorption [63]; (ii) rapid and extensive phase II biotransformation in the intestinal epithelium and hepatocytes, principally glucuronidation (EGCG-4″-O-glucuronide by UGT1A1, UGT1A8, and UGT1A9), sulfation (EGCG-4″-sulfate by SULT1A1 in the liver and SULT1A3 in the intestine, recently identified as the quantitatively dominant conjugation pathway in humans [64]), and catechol-O-methyltransferase (COMT)-mediated methylation generating 4″-O-methyl-EGCG and 4′,4″-di-O-methyl-EGCG [65], all conjugates exhibiting substantially reduced cellular bioactivity compared to the parent compound; and (iii) a short plasma half-life of 1.9–4.6 h, compounded by late-appearing ring-fission metabolites (M4 and M6 valerolactones) generated by colonic microbiota [62] that serve as secondary systemic catechin species of uncertain bioactivity. It must therefore be explicitly acknowledged that the 5–10 µM EGCG concentrations employed in this study are not directly achievable at systemic plasma levels through conventional single-serving green tea consumption, and that dietary intake concentrations do not translate directly to the intracellular concentrations used in in vitro experiments.

Joint tissue pharmacokinetics introduce an additional, compounding layer of complexity that is poorly characterised in the published literature. Articular cartilage is avascular and aneural; bioactive compounds must, after achieving systemic distribution, traverse the synovial microvasculature, equilibrate into the synovial fluid compartment, diffuse through the dense negatively charged extracellular matrix of glycosaminoglycans and type II collagen, and ultimately be transported across the chondrocyte plasma membrane. Each barrier concentrically attenuates the effective intracellular concentration relative to the plasma level. To our knowledge, no published human pharmacokinetic study has directly quantified EGCG concentrations in synovial fluid following oral consumption, a gap that underscores the importance of direct intra-articular delivery strategies. Intra-articular administration of EGCG bypasses systemic bioavailability limitations entirely: Huang et al. [36] demonstrated that intra-articular EGCG injection in spontaneous guinea pig OA effectively attenuates cartilage degeneration, suppresses ADAMTS-5 and MMP-13, and restores anabolic gene expression at locally maintained concentrations. Furthermore, nanoparticle encapsulation strategies, including cationic PLGA/Eudragit RL, are now established to enhance both joint retention and cartilage penetration of molecules such as hyaluronic acid [66], with EGCG-loaded nanoparticle formulations demonstrating enhanced synovial fluid retention and anti-inflammatory activity in in vivo models. These intra-articular and nanoparticle delivery strategies, together with phospholipid-complexed phytosome formulations that achieve 2- to 4-fold higher plasma Cmax relative to unformulated EGCG [22], constitute the pharmacological bridge between the mechanistic proof-of-concept supported here and achievable therapeutic concentrations in the joint microenvironment.

### Selection of the LPS and EGCG concentrations: Physiological versus supraphysiological context

A reasonable question is whether the concentrations of lipopolysaccharide (LPS) and EGCG employed here are physiologically realistic or supraphysiological, and why they were chosen. For LPS, the 10 µg/mL concentration used to establish the inflammatory model lies within the 1–10 µg/mL range that is conventionally applied to elicit reproducible TLR4-dependent inflammatory responses in cultured chondrocytes, and which we and others have previously validated as producing robust pro-inflammatory signalling without cytotoxicity [17–19]. Importantly, this is not an idiosyncratic choice: several independent groups have used closely comparable LPS concentrations to generate inflammatory chondrocyte models of OA. Xu et al. employed precisely 10 µg/mL LPS to induce inflammatory and catabolic mediator expression (IL-6, IL-8, TNF-α, and the MMP panel) in chondrocytes as the in vitro arm of a combined in vitro and in vivo study of osteoarthritis [67], and Ma et al. similarly used LPS to stimulate chondrocytes as a model of the inflammatory and apoptotic processes that underlie OA [68]; comparable LPS-driven chondrocyte models recur throughout the OA literature. The concentration we adopted is therefore consistent with established practice in the field rather than an outlier. We nonetheless acknowledge explicitly that this in vitro concentration is supraphysiological relative to the LPS burden encountered in vivo. Circulating LPS in healthy individuals is in the low picogram-per-millilitre range, and even the chronic, low-grade elevation that characterises metabolic endotoxaemia represents only a two- to three-fold increase over baseline, remaining 10- to 50-fold below the concentrations seen in overt sepsis [69,70]; indeed, the minimum concentration capable of initiating a measurable TLR4-dependent response in susceptible cells can be as low as the picogram-per-millilitre range under sustained exposure [70], far below the stimulus used here. This apparent disparity reflects a general and well-recognised feature of in vitro inflammatory modelling rather than a peculiarity of the present work. Monolayer and short-term cultures lack the amplifying cellular crosstalk, paracrine feedback, and prolonged exposure of the intact joint, and consequently require stimulus concentrations substantially higher than those measured in tissue or synovial fluid in order to evoke a synchronous, robust, and quantifiable response within a tractable experimental window. This principle is well documented for chondrocyte models specifically, where the interleukin-1β concentrations conventionally used for in vitro inflammatory priming (1–10 ng/mL) lie three to four orders of magnitude above the levels actually present in osteoarthritic synovial fluid (typically 1–20 pg/mL), yet are required to produce a reproducible OA-like transcriptional phenotype [71]. The deliberately higher in vitro LPS dose used here is therefore best understood not as a literal reproduction of joint LPS levels but as a means of producing reproducible activation of the TLR4–NF-κB–PAR-2 axis, in exactly the manner that the field already accepts for IL-1β. Crucially, the choice of LPS as the stimulus also carries a coherent translational rationale. In obesity, gut-barrier dysfunction permits translocation of microbiome-derived LPS into the systemic circulation, sustaining a chronic metabolic endotoxaemia that drives TLR4-mediated low-grade inflammation in target tissues including the joint, and both systemic and intra-articular LPS burden have been shown to correlate with radiographic knee OA severity and synovial inflammation [69,70,72]. Obesity is itself among the strongest modifiable risk factors for OA, and current EULAR recommendations for the non-pharmacological core management of hip and knee OA place the maintenance of a healthy weight and weight loss among their central pillars [73]. The LPS stimulus used here thus models, in a controlled and amplified form, a mechanistically authentic and clinically relevant inflammatory input that links the obesity–microbiome axis to the PAR-2-driven catabolic signalling that is the focus of this study.

For EGCG, the 5–10 µM concentrations were selected to sit at the lower end of the range over which EGCG exhibits reproducible anti-inflammatory activity in chondrocytes while remaining non-cytotoxic (Fig 1G), and their relationship to dietary intake is addressed in detail above and in Table 3. As catalogued there across 36 commercially consumed green teas, a single serving delivers EGCG to the intestinal lumen at concentrations (several hundred to several thousand micromolar) that exceed the 5–10 µM used here by one-and-a-half to two-and-a-half orders of magnitude; the working concentrations are therefore modest relative to the dose presented to the gut. The critical caveat, which we emphasise rather than minimise, is that luminal concentration does not equate to systemic or intra-articular exposure, only a small fraction of ingested EGCG is absorbed intact, peak plasma concentrations after a single serving are submicromolar (approximately 0.17 µM [62]), and, to our knowledge, no human pharmacokinetic study has directly quantified EGCG in synovial fluid following oral consumption [62]. The 5–10 µM concentrations employed in vitro are thus not directly attainable in the joint through ordinary green tea consumption, and we explicitly frame the present work as a mechanistic proof-of-concept investigation. The enhanced-bioavailability formulations, intra-articular delivery, and nanoparticulate strategies discussed above represent the routes by which therapeutically effective chondrocyte concentrations could plausibly be achieved. Read together with the EULAR emphasis on weight management and lifestyle modification in OA [73], these considerations position dietary EGCG not as a stand-alone pharmacological therapy at the doses studied here, but as the mechanistic basis for a rationally formulated, joint-targeted intervention warranting dedicated in vivo and pharmacokinetic evaluation.

In reconciling these constraints with our findings, we reiterate that the study is a proof-of-concept mechanistic investigation whose validity is independent of the eventual delivery route, and that chronic habitual consumption and the increased vascular permeability of the inflamed joint may raise local exposure above single-dose predictions. None of these considerations closes the quantitative gap, which we do not claim; rather, joint-targeted delivery, nanoparticulate encapsulation, intra-articular administration, and enhanced-bioavailability phytosome formulations remain the necessary next steps to reach therapeutically relevant chondrocyte concentrations in vivo.

### Translational context across animal, human-tissue, and clinical evidence

We acknowledge that the mechanistic cascade characterised in the present work was, by design, dissected in an in vitro chondrocyte system; however, these findings should not be regarded as an isolated cell-culture phenomenon. The translational context for the mechanistic cascade examined in the present study is supported by a converging body of evidence spanning three hierarchically ordered levels of biological fidelity, which we summarise in Table 4. At the animal in vivo level, intra-articular EGCG administration in the Dunkin-Hartley guinea pig spontaneous osteoarthritis model, the most clinically representative preclinical model in terms of aetiology and histopathological progression, reduced OARSI scores, suppressed the cartilage senescence marker p16INK4a, attenuated MMP-13, and restored anabolic gene expression including COL2A1 and aggrecan, outcomes mechanistically concordant with the cytokine and catabolic suppression documented herein [36]. In the surgically induced destabilisation of the medial meniscus mouse model, systemic EGCG (25 mg/kg i.p.) attenuated OARSI progression at both four- and eight-week time points, suppressed ADAMTS5, the aggrecanase that constitutes the terminal effector of the SOX4-driven catabolic cascade we characterise, and produced concomitant improvements in pain behaviour and dorsal root ganglion nociceptive gene expression, extending the chondroprotective mechanism to include neurogenic sensitisation [74,75] (see Table 4, which records a research integrity caveat for this study). Dai et al. further identified Pla2g2a as a novel in vivo EGCG-responsive target in the monosodium iodoacetate rat model through RNA-seq-guided transcriptomic profiling, supporting the existence of additional downstream effectors beyond the PAR-2 axis and consistent with the residual EGCG efficacy we observe in PAR-2-silenced chondrocytes [41].

**Table 4:** Evidence for EGCG in attenuating inflammation and cartilage degeneration in osteoarthritis: animal in vivo, human ex vivo, and human patient studies.

Reference	Study Design / Model / Population	EGCG Dose & Route	Duration	Markers Assessed	Key Findings	Critical Assessment
**SECTION 1 – Animal In Vivo Studies**						
[36]	**Species: Dunkin-Hartley guinea pig** *Induction*: Spontaneous age-related primary OA (no surgical/chemical induction – highest clinical fidelity) *Delivery:* Intra-articular (IA) injection into the knee joint	*Route:* IA injection of EGCG solution (dose concentration in full text) *In vitro arm:* 5–20 µM EGCG on human articular chondrocytes (IL-1β-stimulated)	Longitudinal OA progression timeline (weeks to months); exact sacrifice endpoint in full text	**Histology:** • Safranin O; OARSI score **IHC:** • Collagen II • MMP-13 • p16INK4a (senescence) • Apoptosis (TUNEL) **In vitro:** • COL2A1, aggrecan mRNA • Autophagy: beclin-1, mTOR	• Reduced proteoglycan loss; lower OARSI scores • Preserved Col II; decreased MMP-13 • Reduced p16INK4a and chondrocyte apoptosis • Restored anabolic gene expression (COL2A1, aggrecan) in vitro • Enhanced autophagic flux (↓mTOR, ↑ beclin-1) *Conclusion: IA EGCG chondroprotective in spontaneous OA*	***Strengths:*** • Spontaneous model best recapitulates idiopathic human OA • Multi-endpoint design (histology + IHC + molecular) • Senescence suppression (p16INK4a) — clinically relevant OA mechanism ***Limitations:*** • IA delivery bypasses oral bioavailability; limits translation to dietary EGCG • PAR-2 / ERK1/2 axis not interrogated in vivo • Group sizes and exact IA dose not in abstract • Guinea pig genetically intractable
[74,75] ⚠ ORI-CONFIRMED MISCONDUCT: Image reuse by co-author D. Leong across 16 NIH grant applications. Paper not retracted as of March 2026; independent replication warranted.	**Species: C57BL/6 mouse (male)** *Induction*: Surgical DMM; gold-standard post-traumatic OA model *Group size*: n = 6/group *Time points*: 4 weeks and 8 weeks post-surgery	**EGCG: 25 mg/kg body weight** *Route:* Intraperitoneal (i.p.), daily *Start:* Day of DMM surgery	4 wks and 8 wks post-DMM (dual time points for early and advanced OA)	**Cartilage:** • Safranin O; OARSI **IHC:** • Aggrecan neoepitope • Col II cleavage • MMP-13 • ADAMTS5 **mRNA:** • MMP-1/-3/-8/-13; ADAMTS5; IL-1β; TNF-α; CITED2 **Pain:** • Locomotor activity; rearing; mechanical allodynia **Neurogenic:** • DRG CCR2, IL-1β, TNF-α mRNA	• OARSI reduced: 4 wk (2.0 → 1.2); 8 wk (6.5 → 2.1) • Decreased aggrecan/Col II cleavage; ↓ MMP-13; ↓ ADAMTS5 • CITED2 upregulated (chondroprotective factor) • Suppressed IL-1β and TNF-α mRNA • Improved locomotor activity, rearing, allodynia • Reduced DRG nociceptive markers (CCR2, IL-1β, TNF-α) **Conclusion: Broadest in vivo OA dataset for EGCG – structural, molecular, and pain endpoints**	**Strengths:** • DMM is the most widely reproduced and validated surgical OA model • Dual time points capture early and advanced disease • Pain behaviour endpoints extend clinical relevance • ADAMTS5 suppression directly corroborates the SOX4–ADAMTS-5 axis in the present study **Limitations:** • i.p. delivery; does not model oral bioavailability • Post-traumatic model only; n = 6/group is marginal • PAR-2 / ERK1/2 not interrogated ⚠ **Integrity:** ORI-confirmed image misconduct. Interpret with caution; await independent replication.
[41]	**Species: Sprague-Dawley rat (male)** *Induction:* Intra-articular MIA (2 mg/50 µL) — chemical chondrolytic model *Group size:* n = 10/group *Parallel in vitro arm:* Primary rat chondrocytes + IL-1β	**Single IA injection: 100 µL EGCG at 10 µg/mL (= 1 µg total)** Administered 2 weeks post-MIA; sacrifice 1-week post-treatment	Single IA treatment at 2 wks post-MIA; sacrifice at 3 wks post-MIA (1-week observation only)	**In vitro:** • ROS; viability • IL-1β, MMP-13, TNF-α • RNA-seq (KEGG/GO/PPI network analysis) **In vivo:** • Pla2g2a (sPLA2-IIA) expression • Micro-CT: BV/TV, trabecular thickness • Histology: H&E and Safranin O **Computational:** • Molecular docking EGCG–Pla2g2a (−6.97 kcal/mol)	• In vitro: ↓ ROS, ↓ cytokines; ↑ Pla2g2a • Pla2g2a identified as principal EGCG-responsive gene by PPI network • In vivo: improved cartilage histology; partial subchondral bone preservation • Stable EGCG–Pla2g2a docking interaction **Conclusion: Identifies Pla2g2a as a novel EGCG target with transcriptomic depth**	**Strengths:** • Multi-platform (in vitro + in vivo + transcriptomics + docking) • Largest group size (n = 10) in this review • Micro-CT extends analysis beyond cartilage to subchondral bone **Limitations:** • MIA: rapid chemical necrosis ≠ gradual enzymatic/inflammatory OA • 1 µg total IA dose has no pharmacokinetic relevance to oral dosing • 1-week observation precludes disease-modifying assessment • Pla2g2a’s causal role in OA not fully established • No pain behaviour or synovial analysis • PAINS characteristics of EGCG acknowledged by authors
**SECTION 2 – Human Ex Vivo Tissue Studies (Primary OA Chondrocytes from Patients)**						
[37]	*Study type*: Human ex vivo primary OA chondrocytes *Source*: Primary chondrocytes isolated from cartilage of OA patients (surgical specimens) *Stimulation*: IL-1β (5 ng/mL, 24 h) ± EGCG pre-treatment Cell source confirmed OA: Institutional Review Board approval stated	**EGCG: 10–100 µM** *Route*: In vitro (direct medium supplementation); 2-hour pre-treatment before IL-1β stimulation	24-hour cytokine stimulation after 2-hour EGCG pre-treatment; single time point	**Cytokine antibody array:** • 80-protein panel on conditioned medium (chemiluminescent detection) **mRNA (RT-PCR, 18 genes):** • MMP-1, −3, −8, −13; ADAMTS4/5; COX-2; iNOS; IL-6; IL-8; NF-κB targets **ELISA:** • IL-6, IL-8, TNF-α protein (secreted)	• EGCG suppressed >50% of the 80 assayed proteins including IL-6, IL-8, MCP-1, GM-CSF, GRO-α, IP-10, ENA-78, and multiple chemokines • Dose-dependent inhibition of IL-6, IL-8, and TNF-α (ELISA confirmation) • Suppressed MMP-1, −3, −8, −13 and ADAMTS4/5 mRNA • Inhibited NF-κB activation and nuclear translocation • Reduced COX-2 and iNOS expression **Conclusion: Comprehensive suppression of the OA inflammatory network in primary human chondrocytes from OA patients, directly supporting PAR-2 → NF-κB → TNF-α/MCP-1 axis documented in present study**	**Strengths:** • Primary human OA cartilage specimens — highest human tissue fidelity • Broadest inflammatory panel (80 proteins) assayed in any EGCG-OA study • Multi-method validation: array → RT-PCR → ELISA • Directly relevant: MCP-1 and TNF-α suppression overlap with present manuscript’s key endpoints **Limitations:** • In vitro only; no structural or in vivo readouts • Supra-physiological EGCG concentrations (100 µM) may not be achievable in joint fluid after oral intake • Patient number and clinical characteristics (KL grade, joint site) not reported in detail • PAR-2 signalling not interrogated **Relevance to present study:** TNF-α, MCP-1, and MMP suppression in primary human OA cells directly corroborate the mechanistic cascade (PAR-2→ERK1/2→TNF-α/MCP-1) described herein.
[76]	*Study type*: Human ex vivo primary OA chondrocytes (miRNA microarray) *Source:* Chondrocytes derived from OA cartilage specimens of patients undergoing knee arthroplasty Stimulation: IL-1β (5 ng/mL) ± EGCG *Platform:* Human miRNA microarray (1,347 miRNAs assayed); validated by TaqMan assay and miRNA inhibitor transfection	**EGCG: Concentration consistent with Haqqi laboratory (10–100 µM range)** *Route:* Direct supplementation of culture medium	miRNA expression profiling post-IL-1β stimulation with and without EGCG pre-treatment; standard 24-hour OA inflammatory challenge protocol	**miRNA microarray:** • 1,347 human miRNAs screened • EGCG: 19 upregulated, 17 downregulated vs. IL-1β alone **Target pathway analysis:** • miR-140-3p / ADAMTS5 axis • miR-140-3p is a negative regulator of ADAMTS5 mRNA **Functional validation:** • TaqMan assay confirmation of key miRNAs • miRNA inhibitor transfection to confirm miR-140-3p / ADAMTS5 regulatory relationship	• EGCG upregulated miR-140-3p (most significantly altered miRNA) • miR-140-3p negatively co-regulates ADAMTS5 mRNA in OA chondrocytes • EGCG therefore suppresses ADAMTS5 through epigenetic miRNA-mediated mechanism, independent of NF-κB/ERK1/2 transcriptional suppression • 36/1347 miRNAs differentially expressed indicates selective rather than global miRNA disruption **Conclusion: Identifies a miR-140-3p → ADAMTS5 epigenetic pathway as a second mechanism by which EGCG suppresses catabolic activity in human OA chondrocytes**	**Strengths:** • Primary human OA cartilage specimens – genuine patient tissue • Genome-wide miRNA screen (1,347 miRNAs) – discovery-level evidence • Functional validation by TaqMan and inhibitor transfection strengthens regulatory claim • miR-140-3p / ADAMTS5 connection is of direct and critical relevance: the present manuscript demonstrates EGCG suppression of the SOX4-ADAMTS-5 axis, and this study provides the upstream epigenetic basis for ADAMTS5 suppression in human OA tissue **Limitations:** • In vitro only; cannot confirm whether these miRNA changes occur in OA joints in vivo • Patient numbers and clinical OA severity not detailed in abstract • EGCG concentration may be supra-physiological **Relevance to present study**: The miR-140-3p→ADAMTS5 mechanism described here is an epigenetic complement to the SOX4→ADAMTS-5 transcriptional suppression documented in the present manuscript.
**SECTION 3 — Human Patient Studies (Epidemiological Cohort and Randomised Controlled Trial)**						
[77]	**Study type: Prospective population-based cohort study** *Population*: 11,091 Japanese community-dwellers (Murakami City, Niigata, Japan) *Age*: Mean 58.1 years (SD 9.3) *Exposure*: Self-reported green tea consumption frequency (dietary questionnaire) OA definition: Symptomatic knee OA of grade ≥2 (Kellgren-Lawrence radiographic scoring)	**Dietary exposure – habitual green tea consumption** *Assessment*: Categorised green tea intake frequency (cups/day) via validated dietary questionnaire No supplemental purified EGCG used; exposure reflects natural green tea catechins	5-year follow-up (to 2016); incident knee OA cases ascertained at hospitals and orthopaedic clinics.	**Primary outcome:** • Incident symptomatic knee OA (K-L grade ≥2 + knee symptoms) • 429 incident OA cases identified over 5 years **Secondary/stratified analyses:** • Sex-stratified associations (men vs. women) • Adjustment for: age, BMI, physical activity (METs), smoking, alcohol **Statistical analysis:** • Multivariate logistic regression; P for trend across consumption categories	• In men: higher green tea consumption was significantly associated with lower incidence of knee OA (P for trend = 0.0437) • In women: no significant association between green tea intake and knee OA incidence • Sex-dependent differences observed for multiple lifestyle factors (age, BMI strongest risk factors in both sexes) • Alcohol consumption associated with higher knee OA risk in women, less smoking and less physical activity in men **Conclusion: First prospective cohort evidence of an inverse association between habitual green tea consumption and incident symptomatic knee OA in men, consistent with EGCG’s anti-inflammatory mechanisms**	**Strengths:** • Large prospective cohort (n = 11,091) with radiographic OA endpoint confirmation • 5-year follow-up captures incident disease over meaningful time horizon • Validated dietary questionnaire, adjustment for major confounders • East Asian population with high habitual green tea consumption ecologically appropriate for dose-response analysis **Limitations:** • Green tea intake assessed by dietary questionnaire does not measure EGCG plasma levels or bioavailability • Green tea contains multiple bioactive compounds; cannot attribute effects to EGCG alone • Sex-specificity of the association (men only) limits generalisability; plausible biological mechanism for sex dimorphism not established • Residual confounding cannot be excluded in observational design • Kellgren-Lawrence grading has limited sensitivity for early structural changes **Relevance to present study:** Provides the only population-level evidence that green tea intake reduces OA incidence in humans, lending clinical translational context to the mechanistic in vitro and in vivo findings.
[78]	**Study type: Randomized open-label active-controlled clinical trial** *Centre:* Shiraz University of Medical Sciences, Iran Participants: 50 adults with radiographically confirmed knee OA *Arms:* Green tea extract tablet + diclofenac (n = 25) vs. diclofenac alone (n = 25) Randomisation: Computer-generated allocation; open-label design	**Green tea extract (GTE) tablet: standardised extract containing catechins including EGCG** *Comparator:* Diclofenac 100 mg/day (NSAID — standard-of-care) Both arms received diclofenac; GTE was additive supplementation	4 weeks (28 days) of treatment; assessed at baseline and week 4; adverse effects monitored throughout	**Pain:** • Visual Analogue Scale (VAS) — 0–100 mm **Patient-reported function:** • WOMAC total score • WOMAC pain subscale • WOMAC stiffness subscale • WOMAC physical function subscale **Safety:** • Adverse effects questionnaire throughout trial	• VAS pain: GTE + diclofenac significantly superior to diclofenac alone (P = 0.038) • WOMAC total score: significantly reduced in GTE group (P = 0.006) • WOMAC physical function: significantly improved in GTE group (P = 0.004) • WOMAC pain subscale: no significant between-group difference • WOMAC stiffness subscale: no significant between-group difference • No adverse effects reported in GTE-treated group **Conclusion: Adjunctive green tea extract produced significant additive benefit on global pain (VAS) and functional capacity (WOMAC) over diclofenac monotherapy, supporting safety and clinical utility of EGCG-containing preparations in knee OA management**	**Strengths:** • Only randomised controlled clinical trial of green tea/EGCG specifically in OA patients • Active comparator (diclofenac) rather than placebo — real-world adjunctive clinical question • Validated, disease-specific instruments (VAS, WOMAC) • No adverse effects observed — supports safety profile **Limitations:** • Small sample (n = 50; 25/group) – underpowered for detecting differential subscale effects • Open-label design introduces performance and detection bias; placebo arm absent • Very short duration (4 weeks) precludes assessment of structural (disease-modifying) effects • GTE tablet not standardised for specific EGCG content – exact active dose unknown • No biomarker assay (CRP, IL-6, cartilage turnover markers); mechanism of improvement unstudied • No radiographic follow-up; structural changes not assessed • Population predominantly male (unequal sex distribution reported) **Relevance to present study**: Provides the only direct clinical validation that EGCG-containing preparations improve pain and function outcomes in knee OA patients – the translational endpoint of the mechanistic cascade characterised herein.

***Abbreviations***: ADAMTS, a disintegrin and metalloproteinase with thrombospondin motifs; BV/TV, bone volume fraction; CITED2, CBP/p300-interacting transactivator 2; Col II, collagen type II; COX-2, cyclooxygenase-2; DRG, dorsal root ganglion; DMM, destabilisation of the medial meniscus; EGCG, epigallocatechin-3-gallate; GTE, green tea extract; IA, intra-articular; i.p., intraperitoneal; IHC, immunohistochemistry; IL-1β, interleukin-1β; K-L, Kellgren-Lawrence; MCP-1, monocyte chemotactic protein-1; miRNA, microRNA; MMP, matrix metalloproteinase; mTOR, mechanistic target of rapamycin; NF-κB, nuclear factor kappa-B; OA, osteoarthritis; OARSI, Osteoarthritis Research Society International; PAR-2, protease-activated receptor-2; Pla2g2a, phospholipase A2 group IIA; ROS, reactive oxygen species; RT-PCR, real-time polymerase chain reaction; TNF-α, tumour necrosis factor-α; VAS, visual analogue scale; WOMAC, Western Ontario and McMaster Universities Osteoarthritis Index.

Critically, two independent investigations employing primary chondrocytes derived directly from osteoarthritic cartilage of patients undergoing knee arthroplasty provide human-tissue-level corroboration of our principal endpoints. Akhtar and Haqqi demonstrated that EGCG globally suppressed an 80-protein inflammatory network in IL-1β-stimulated primary human OA chondrocytes, with the most pronounced effects on TNF-α, IL-6, MCP-1, and the full MMP and ADAMTS catabolic panel [37], an independent, human-tissue validation of the precise effectors quantified in our ELISA and RT-PCR datasets. Rasheed et al. extended this to the epigenetic dimension, demonstrating that EGCG upregulates miR-140-3p, a well-characterised negative regulator of ADAMTS5 mRNA, in genome-wide miRNA profiling of human OA chondrocytes [76]. The miR-140-3p→ADAMTS5 post-transcriptional regulatory axis identified in primary human OA tissue constitutes an epigenetic complement to the SOX4→ADAMTS-5 transcriptional cascade downstream of PAR-2–ERK1/2 that we characterise in the present study, together delineating a dual-layered regulatory architecture, one transcriptional, one post-transcriptional, through which EGCG coordinates suppression of aggrecanase-mediated matrix catabolism in OA chondrocytes. That both mechanisms have now been independently documented in primary human OA cartilage specimens substantially strengthens the case for human relevance.

At the human patient level, the Murakami prospective cohort study (n = 11,091; five-year follow-up) reported that higher habitual green tea consumption was significantly associated with a lower incidence of symptomatic knee OA in men after multivariate adjustment for the major confounders including age, body mass index, and physical activity (P for trend = 0.0437) [77]. The sex-specificity of this association, restricted to men, is biologically plausible given documented dimorphisms in chondrocyte inflammatory responsiveness and EGCG pharmacokinetics, and warrants prospective investigation in adequately powered cohorts with biomarker endpoints. In the sole randomised clinical trial of green tea extract in knee OA patients to date (n = 50; green tea extract plus diclofenac versus diclofenac alone, four weeks), Hashempur et al. reported significant adjunctive improvements in visual analogue scale pain score (P = 0.038) and WOMAC total (P = 0.006) and physical function subscale scores (P = 0.004) with no adverse events, demonstrating a clinically meaningful analgesic benefit over standard-of-care NSAID therapy at intake concentrations that are physiologically attainable, as documented in Table 3 of the present manuscript [78]. Taken together, the hierarchical convergence of in vitro mechanistic data, multi-model animal in vivo corroboration, primary human OA tissue validation, prospective epidemiological evidence, and randomised clinical trial data provides supportive translational context for the PAR-2→ERK1/2→TNF-α/SOX4/ADAMTS-5 cascade characterised here within a broader and clinically meaningful context, while underscoring the priority of purpose-designed in vivo PAR-2 studies as the immediate next step in the translational pathway.

***Research integrity note***: The row for references [74,75] in Section 1 of Table 4 is flagged because the US Office of Research Integrity (ORI) confirmed that a co-author committed research misconduct through image reuse across 16 NIH grant applications. The published paper has not been formally retracted as of March 2026; findings should be interpreted with appropriate caution pending independent replication.

Note on translational hierarchy: Evidence in this table is ordered from the lowest to the highest level of translational fidelity (animal model→human tissue→human patient). The human ex vivo data (Section 2) use primary chondrocytes derived from OA patient surgical specimens and represent the closest available cellular model of human OA. The human patient data (Section 3) provide direct clinical relevance: the Murakami cohort [77] establishes a prospective epidemiological association between green tea intake and reduced OA incidence, while the randomised trial [78] demonstrates significant adjunctive clinical benefit over standard-of-care diclofenac, supporting translational potential of the PAR-2–chondroprotective mechanism documented in the present manuscript.

### Rationale for the LPS–TLR4 model as a surrogate for DAMP-driven inflammation in osteoarthritis

A consideration that informs the interpretation of the present findings is the choice of LPS as the pro-inflammatory stimulus, which was predicated on the innate immunobiology of OA rather than on its generic utility as an in vitro inflammatory agent. In osteoarthritic cartilage, progressive enzymatic and mechanical disintegration of the extracellular matrix generates a diverse repertoire of damage-associated molecular patterns (DAMPs), including fibronectin fragments, high-mobility group box 1 (HMGB1), tenascin-C, hyaluronan oligosaccharides, and S100A8/A9, that collectively drive innate immune activation and persistent low-grade synovial inflammation [79,80]. Toll-like receptor 4 (TLR4) is the canonical pattern-recognition receptor through which the preponderance of these endogenous DAMPs transduce their signals in chondrocytes and synoviocytes, and TLR4 surface expression is elevated in osteoarthritic cartilage in proportion to histological disease severity [81]. As the high-affinity ligand of the TLR4–MD-2–CD14 co-receptor complex, LPS activates the identical intracellular cascades, MyD88 → IRAK1/4 → TRAF6 → TAK1 → IKKβ → NF-κB and TAK1 → MKK1/2 → ERK1/2, as those initiated by endogenous TLR4-activating DAMPs, because downstream signalling is determined by the intracellular adaptor architecture rather than by ligand identity [82]. LPS therefore serves as a mechanistically faithful surrogate for DAMP-mediated TLR4 activation in the OA joint, a rationale reinforced by evidence that both systemic and intra-articular LPS burden in humans are independently associated with radiographic knee OA severity and synovial inflammatory biomarkers [72]. This systemic source of TLR4 ligand is itself mechanistically grounded: metabolic endotoxaemia, the chronic low-grade elevation of circulating LPS arising from gut-barrier dysfunction in obesity, sustains systemic and synovial TLR4-driven inflammation and provides a biologically authentic basis for LPS as a model stimulus of the metabolically driven OA phenotype that constitutes a substantial share of the contemporary OA population [69].

The suitability of LPS is further reinforced by the mechanistic focus of this study on PAR-2. PAR-2 transcription in chondrocytes and innate immune cells is governed by NF-κB response elements in the F2RL1 promoter [83], and TLR4 → MyD88 → TRAF6 → IKKβ → NF-κB signalling constitutes the primary upstream inductive pathway for PAR-2 expression in this context. LPS stimulation thus reproduces the TLR4 → NF-κB → PAR-2 transcriptional axis operant in DAMP-stimulated osteoarthritic cartilage, as corroborated by the approximately four-fold increase in PAR-2 mRNA we observed in LPS-stimulated chondrocytes (Fig 2C). The commonly used alternative stimulus IL-1β signals through a structurally distinct receptor (IL-1R1) and, being itself a downstream effector of PAR-2 signalling, would position a product of the receptor as the upstream stimulus, introducing a logical circularity that would complicate interpretation of PAR-2-centred experiments. By activating PAR-2 through its authentic transcriptional inductive pathway and positioning PAR-2 as a primary downstream effector rather than as a precondition of the model, LPS–TLR4 stimulation is therefore the specifically appropriate stimulus for the experimental question addressed here.

### Limitations

Several limitations warrant consideration. Foremost, the present investigation is, by design, an *in vitro* mechanistic study, and as such it does not fully recapitulate the systemic and joint-level pharmacokinetic, biomechanical, and multicellular complexity of the osteoarthritic joint in vivo, nor the physiological conditions that govern EGCG bioavailability and tissue distribution. To situate the present findings within the wider translational landscape rather than to overstate their immediate clinical reach, we have collated the available animal in vivo, human ex vivo, and human patient evidence into a structured hierarchical framework (Table 4); this framework suggests that the PAR-2→ERK1/2→TNF-α/SOX4/ADAMTS-5 cascade dissected here is concordant with, and corroborated by, evidence at progressively higher levels of biological fidelity. We nonetheless emphasise that this contextual evidence is not a substitute for direct in vivo interrogation, and that purpose-designed in vivo studies targeting the PAR-2–ERK1/2 axis remain the essential next step before clinical inferences can be drawn. Our model employs LPS (a TLR4 agonist) as the inflammatory stimulus; although widely validated, this does not fully recapitulate the OA milieu in which endogenous PAR-2 agonists (trypsin, tryptase, matriptase) and mechanical load co-drive pathology [84,85]. As detailed above, LPS nonetheless reproduces the TLR4-driven cascade through which endogenous DAMPs signal in the OA joint, and is the appropriate stimulus for the PAR-2-centred question addressed here. We further acknowledge that the LPS–TLR4 stimulus does not capture the full breadth of innate immune activation operative in the osteoarthritic joint. First, LPS engages the extracellular TLR4–MD-2–CD14 complex only, whereas endogenous DAMPs additionally signal through intracellular pattern-recognition receptors, including the NLRP3 inflammasome, and through integrins such as α5β1 and αvβ3, pathways that LPS stimulation does not reproduce [86,87]. Second, mechanical-loading–induced inflammation, which is transduced by distinct mechanosensing machinery including PIEZO1/2 channels and integrin-mediated signalling, is not modelled by a soluble chemical stimulus [88,89]. Third, the present monoculture does not recapitulate the cellular ecology of the OA joint, in particular the reciprocal crosstalk among chondrocytes, synoviocytes, and macrophages that shapes the inflammatory response in vivo [90]. These constraints temper the interpretation of the present findings and define clear priorities for multi-stimulus, co-culture, and in vivo models in future work.

A further consideration concerns the cellular model itself. The chondrocytes used here were differentiated from bone marrow-derived mesenchymal stem cells (BM-MSCs) and, although phenotypically validated against established chondrogenic criteria, they represent a developmentally “naive” chondrocyte population that may not fully reproduce the molecular state of chondrocytes resident in chronically diseased human OA cartilage. Osteoarthritic chondrocytes acquire a distinctive and heritable epigenetic landscape, encompassing aberrant DNA methylation and histone modifications that, together with cellular senescence, drive the sustained imbalance between catabolic and anabolic gene programmes that characterises the disease; this progressive, stably propagated remodelling is often termed epigenetic drift [91,92]. Such disease-associated marks, for example hypomethylation of the promoters of matrix-degrading proteases including MMP-13 and the ADAMTS aggrecanases, are established ***in situ*** over years of mechanical and inflammatory stress and are unlikely to be recapitulated by a freshly differentiated BM-MSC-derived population. In addition, monolayer expansion of chondrocytes is itself accompanied by dedifferentiation and culture-induced shifts in DNA methylation that alter expression of phenotypic and signalling genes, including components of the ERK pathway interrogated here [93], underscoring that the transcriptional baseline of any in vitro chondrocyte model is sensitive to its derivation and culture history. Consequently, the magnitude and even the hierarchy of EGCG’s effects on the PAR-2–ERK1/2 axis could differ in primary OA chondrocytes carrying the full epigenetic burden of established disease, and the present findings should be regarded as defining the mechanistic potential of EGCG in a well-controlled chondrocyte system rather than as a quantitative prediction of its activity in diseased tissue. We therefore regard validation in primary OA patient-derived chondrocytes, ideally in three-dimensional or co-culture systems that preserve the diseased phenotype and incorporate synoviocytes, as an important next step. Continuous *in vitro* exposure to a fixed EGCG concentration does not reflect the pharmacokinetic reality of intermittent oral dosing [22], and the 24-h treatment window precludes assessment of chronic effects on matrix synthesis or phenotypic stability. A further limitation concerns the scope of the mitochondrial assessment. The two readouts employed here, Rhodamine 123 fluorescence and Janus Green B staining, report, respectively, the thermodynamic state of the inner membrane (mitochondrial membrane potential, ΔΨm) and the structural parameter of mitochondrial mass. They are validated and informative markers of mitochondrial integrity, but they are not functional bioenergetic assays, and they do not directly measure respiratory chain activity or ATP production [94]. Accordingly, our claims are confined to the preservation of these structural and thermodynamic markers, and we explicitly refrain from inferring that EGCG restores oxidative-phosphorylation–coupled ATP synthesis or respiratory capacity; such functional conclusions would require direct measurement. Definitive characterisation of the bioenergetic consequences of EGCG-mediated PAR-2 suppression will therefore require dedicated functional assays, including Seahorse XF-based profiling of the oxygen consumption rate (OCR) and extracellular acidification rate (ECAR), luminometric ATP quantification, and mitochondria-targeted ROS measurement (MitoSOX Red); these are planned for the in vivo follow-up investigation.

Finally, although the siRNA data support PAR-2 involvement, the residual EGCG efficacy in silenced cells indicates additional targets that remain to be fully characterised. Future *in vivo* studies employing destabilization of the medial meniscus or similar models [95], coupled with intra-articular delivery strategies (e.g., nanoparticle encapsulation [96]), will be essential to establish whether the mechanistic cascade delineated here translates into disease modification at the joint level.

## Conclusions

This study supports EGCG as a potential inhibitor of the PAR-2–ERK1/2–TNF-α signaling axis in OA chondrocytes, with downstream suppression of the SOX4–ADAMTS-5 catabolic cascade, RANK/RANKL osteoclastogenic signaling, and inflammation-induced mitochondrial dysfunction. The siRNA validation (Fig 8) provides mechanistic evidence that PAR-2 is the predominant, though not sole mediator of EGCG’s anti-inflammatory action. The high EGCG dose delivered by dietary green tea relative to the effective in vitro range (Table 3), together with the breadth of downstream effects captured in the integrated signalling model (Fig 9), supports the candidacy of EGCG as a multifunctional agent for dietary-based adjunctive management of OA, a proposition that now requires *in vivo* validation.

## Supporting information

S1 File Graphical abstract. (PNG)

S1 Fig Full-length uncropped Western blot membranes corresponding to the main manuscript figures. Raw chemiluminescent and colorimetric blot images showing molecular weight markers and target bands for proteins of interest and their corresponding GAPDH loading controls across experimental lanes.
(PDF)

S2 Fig Flow cytometry gating strategy and fluorescence intensity histograms for RANK and RANKL expression. (A) Initial forward scatter (FSC-A) versus side scatter (SSC-A) dot plot used to exclude cellular debris and gate viable cells (P1). (B) Forward scatter area (FSC-A) versus height (FSC-H) plot used to select single cells (Singlets, P2) and eliminate cell doublets. (C) Representative histogram showing fluorescence intensity distribution for RANK+ PE staining (red shaded curve) compared to the negative/isotype control (gray shaded curve), with a dashed threshold line indicating positive gating. (D) Representative histogram showing fluorescence intensity distribution for RANKL+ FITC staining (red shaded curve) compared to the negative/isotype control (gray shaded curve).
(PDF)

S3 Fig Full-length, uncropped western blot for Figure 8A (PAR-2). Full-length, uncropped chemiluminescent western blot from which the cropped panel shown in Figure 8A was prepared. PAR-2 migrates at approximately 44 kDa and the GAPDH loading control at approximately 37 kDa; the positions of the 44 kDa and 37 kDa molecular-weight markers are indicated at the left of the membrane. PAR-2 is strongly expressed in the non-silenced lanes (1–4) and is efficiently ablated in the PAR-2 siRNA–transfected lanes (5–8), while GAPDH remains uniform across all lanes. The lane numbering (1–8) corresponds to that used in Fig 8A.
(PDF)
