# Supplementary material for: Epigallocatechin-3-gallate attenuates PAR-2–mediated inflammatory signaling and preserves markers of mitochondrial integrity in osteoarthritic chondrocytes
Source: PLoS One. 2026 Nov 2;21(11):e0357766. doi: 10.1371/journal.pone.0357766 (PMC13726950; doi:10.1371/journal.pone.0357766)

Supplementary Information

Epigallocatechin-3-gallate Attenuates PAR-2–mediated Inflammatory Signaling and Preserves Markers of Mitochondrial Integrity in Osteoarthritic Chondrocytes

Rajashree Patnaik[1] and Yajnavalka Banerjee[1] *

[1]College of Medicine,
Mohammed Bin Rashid University of Medicine and Health Sciences (MBRU),
Dubai Health, Dubai, United Arab Emirates.

PAR 2 for Figure 2A

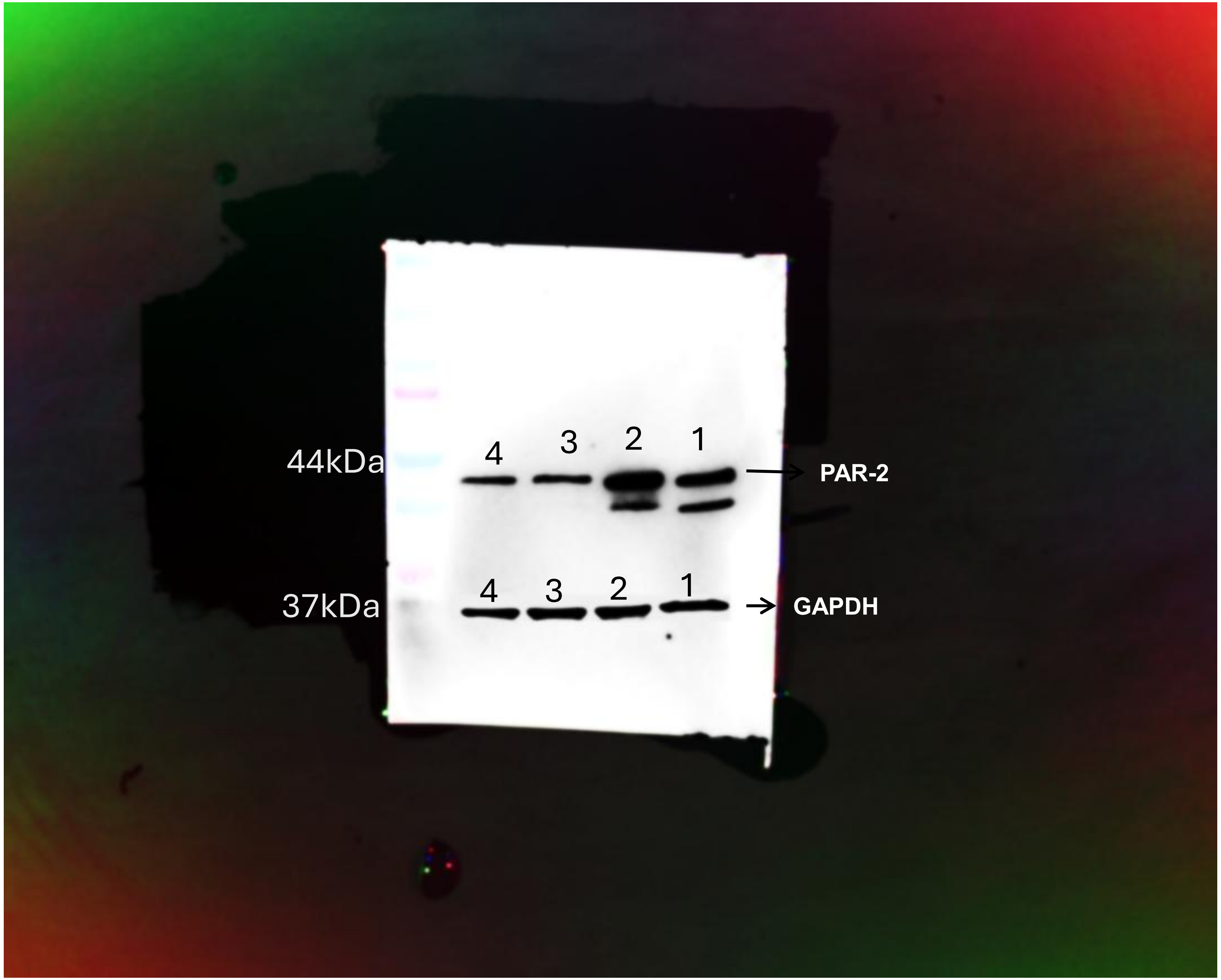

Submitted Blot for PAR 2

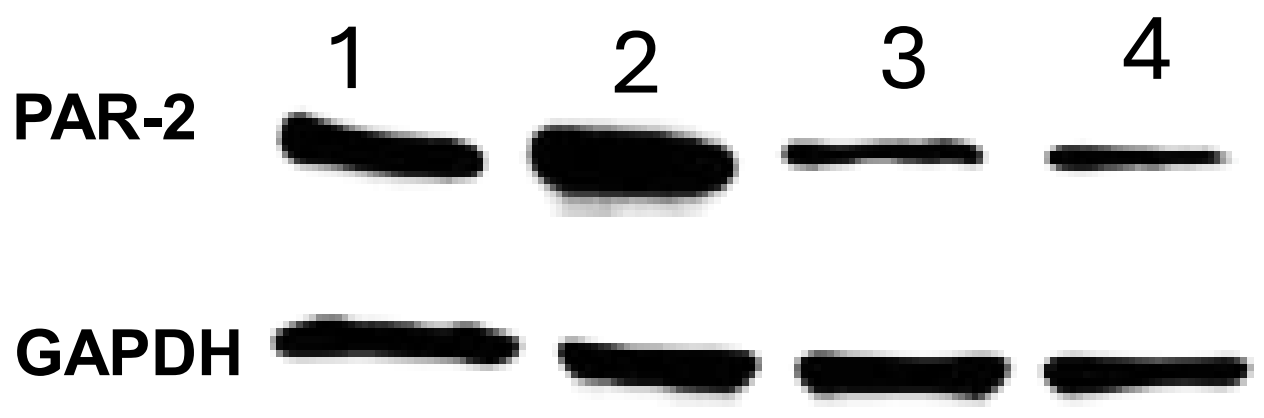

pERK ½ for Figure 3A

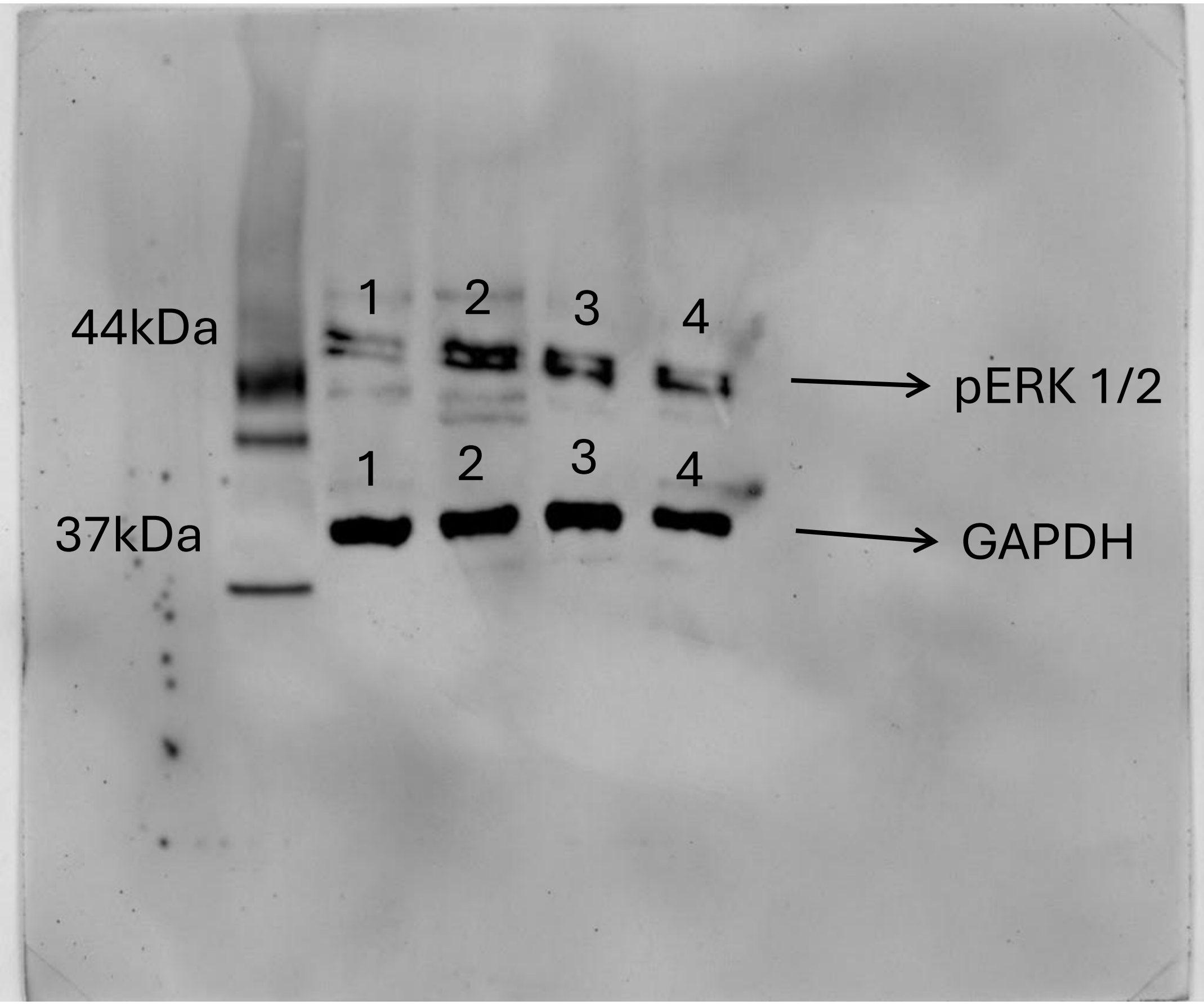

Submitted Blot for pERK 1/2

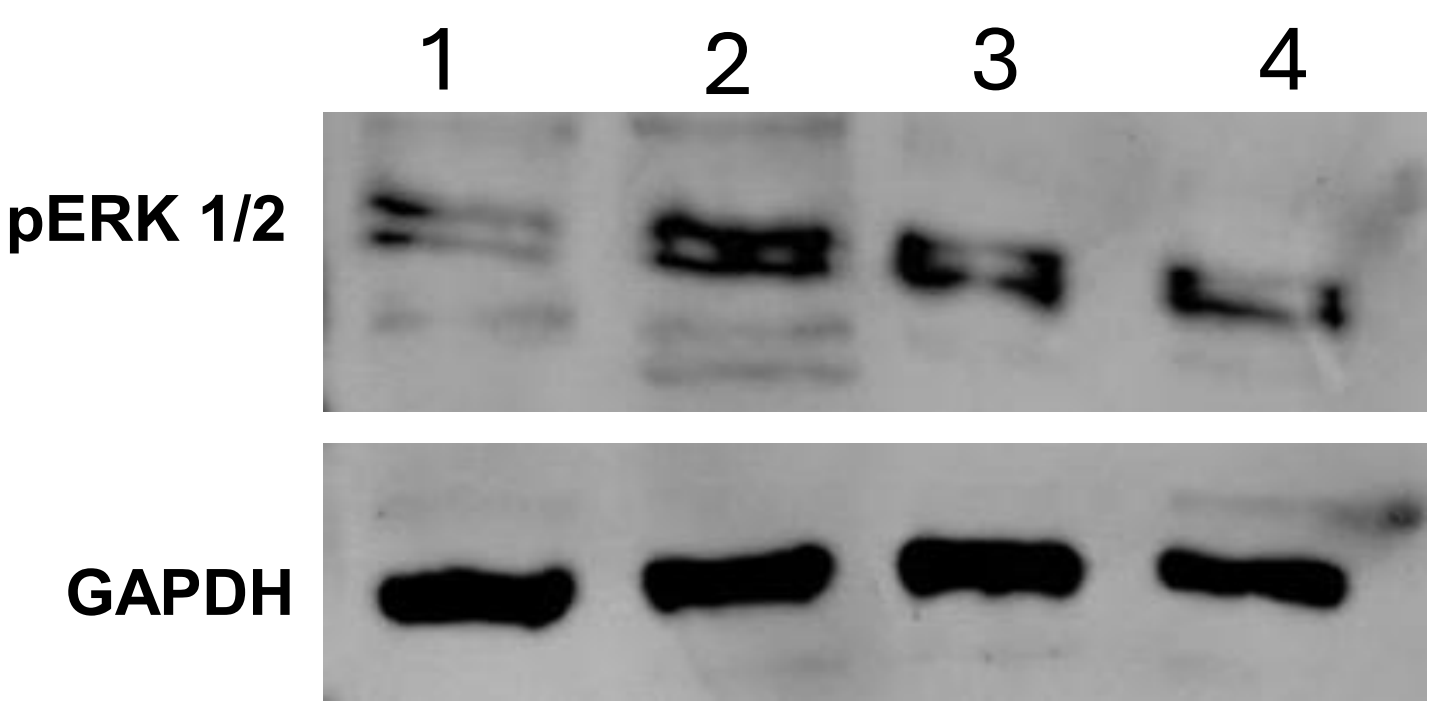

ERK ½ for Figure 3A

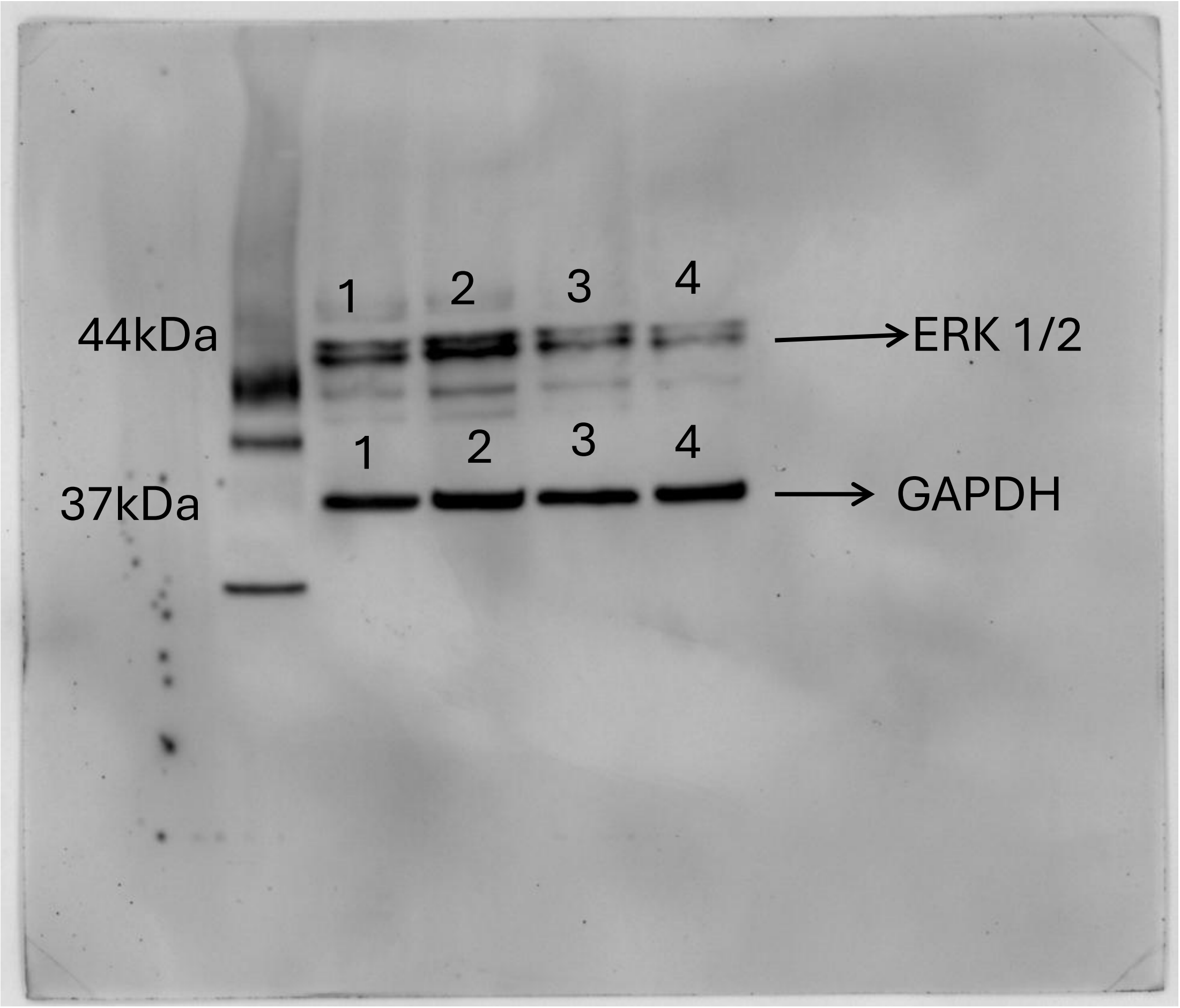

Submitted Blot for ERK 1/2

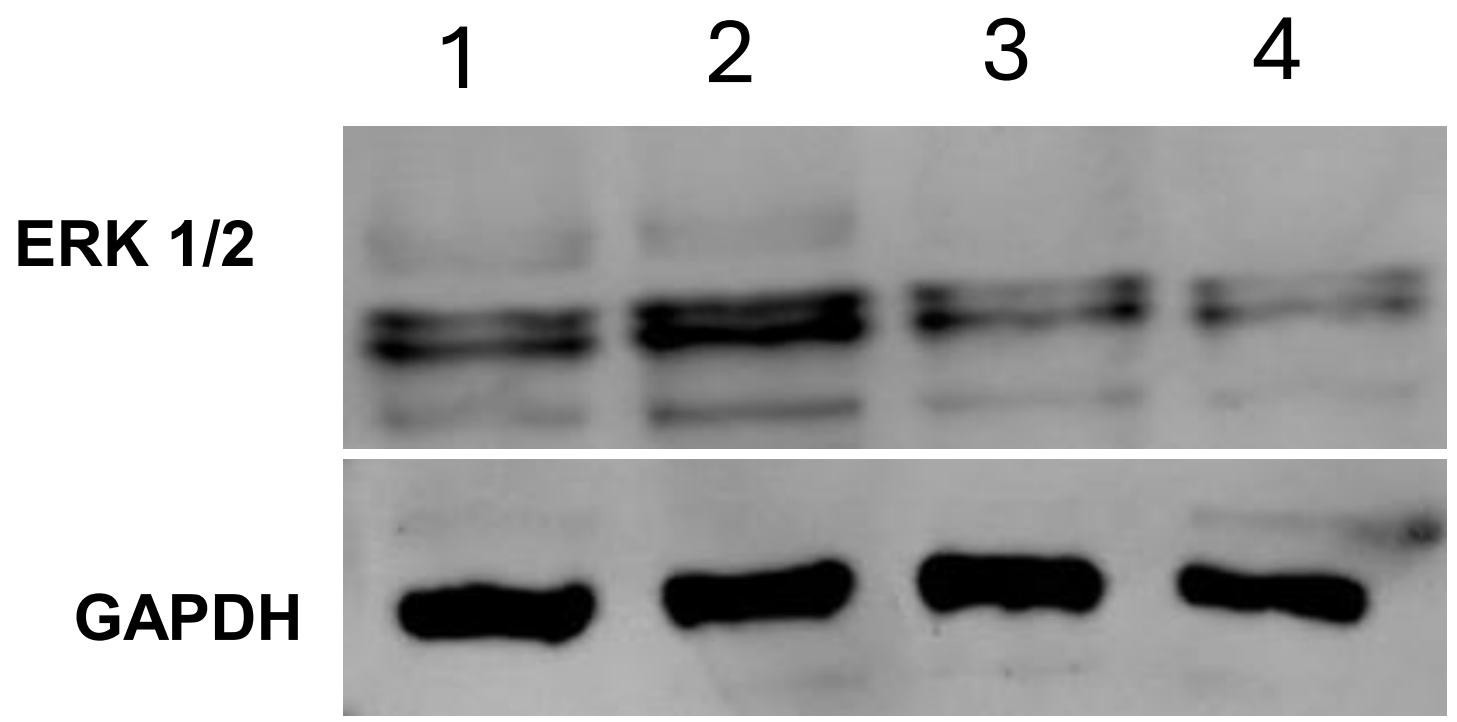

Note on Sequential Probing and Densitometric Analysis (For Figure 3A):

Phospho-ERK1/2 (p-ERK1/2) and total ERK1/2 were detected on the same nitrocellulose membrane via sequential stripping and reprobing. The membrane was first incubated with anti-p-ERK1/2 antibody and imaged. Following mild stripping, the membrane was reprobed for GAPDH to assess sample loading. Subsequently, the membrane underwent an additional stripping cycle and was reprobed for total ERK1/2, followed by a final reprobing for GAPDH to monitor signal retention. To ensure consistency and avoid potential artifacts from cumulative protein loss across multiple stripping cycles, the GAPDH signal obtained directly after the initial probing round was utilized for all quantitative densitometric calculations (both p-ERK1/2/GAPDH and ERK1/2/GAPDH ratios).

SOX4 for Figure 5A

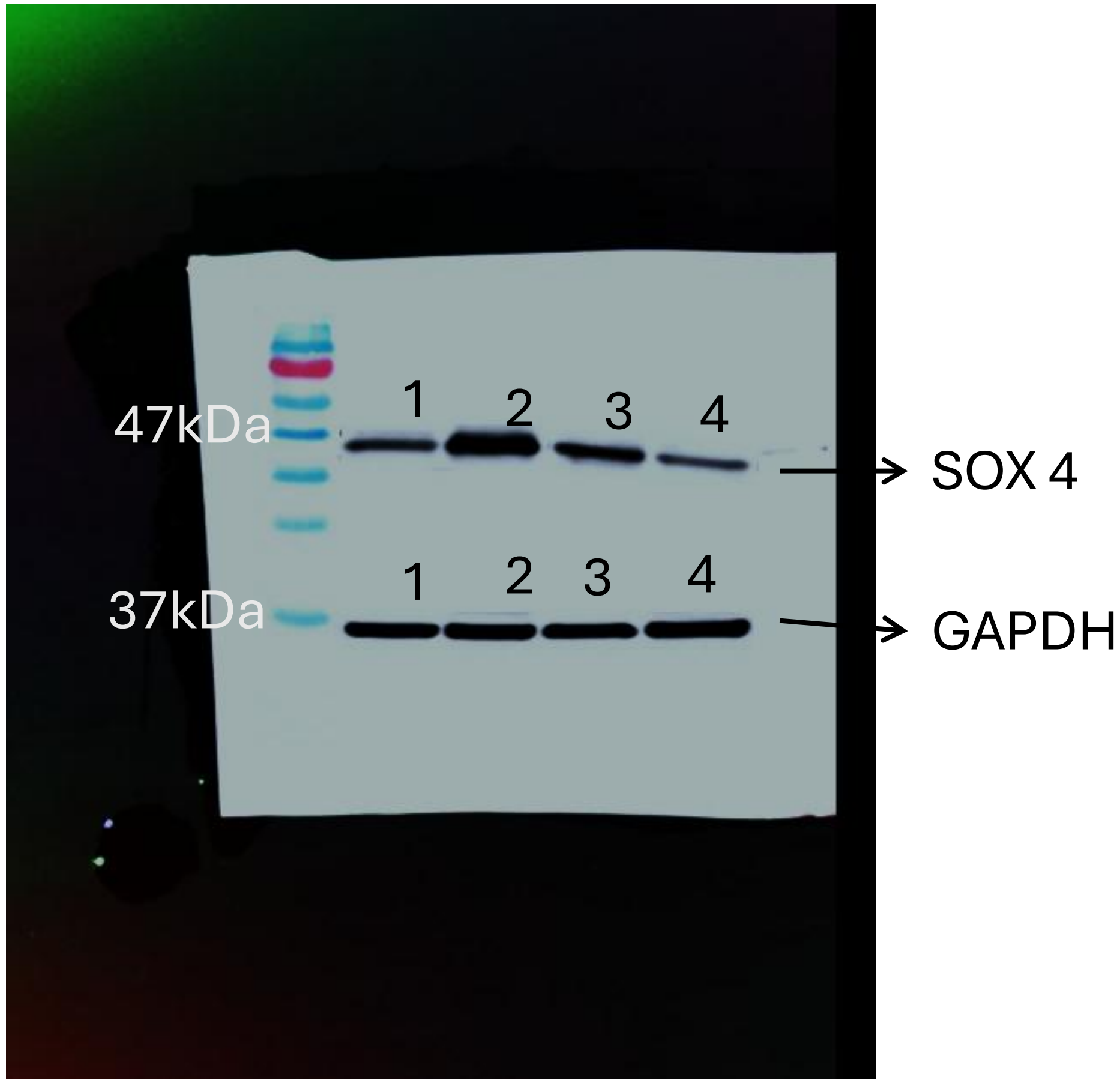

Submitted Blot for SOX 4

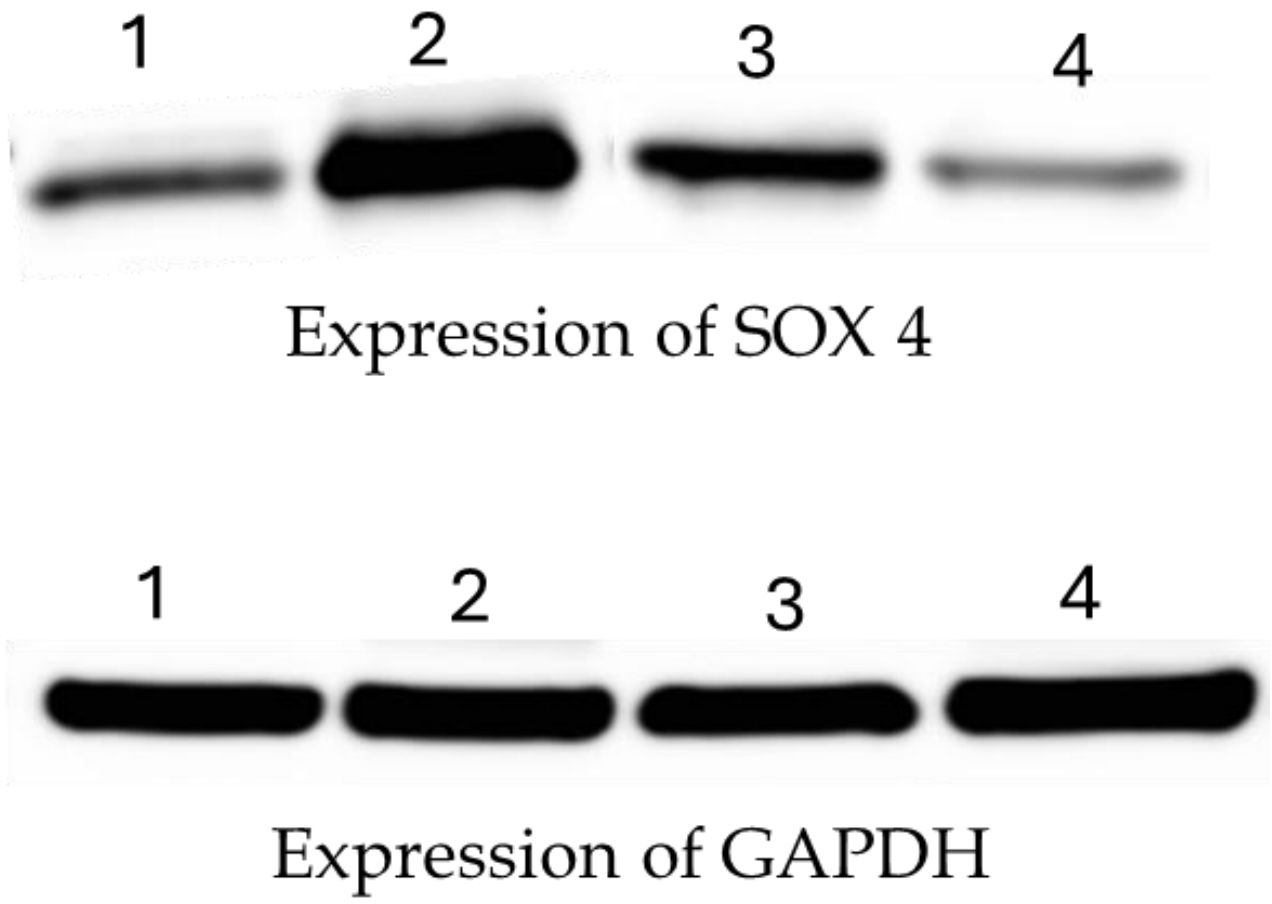

ADAMTS 5 for Figure 5D

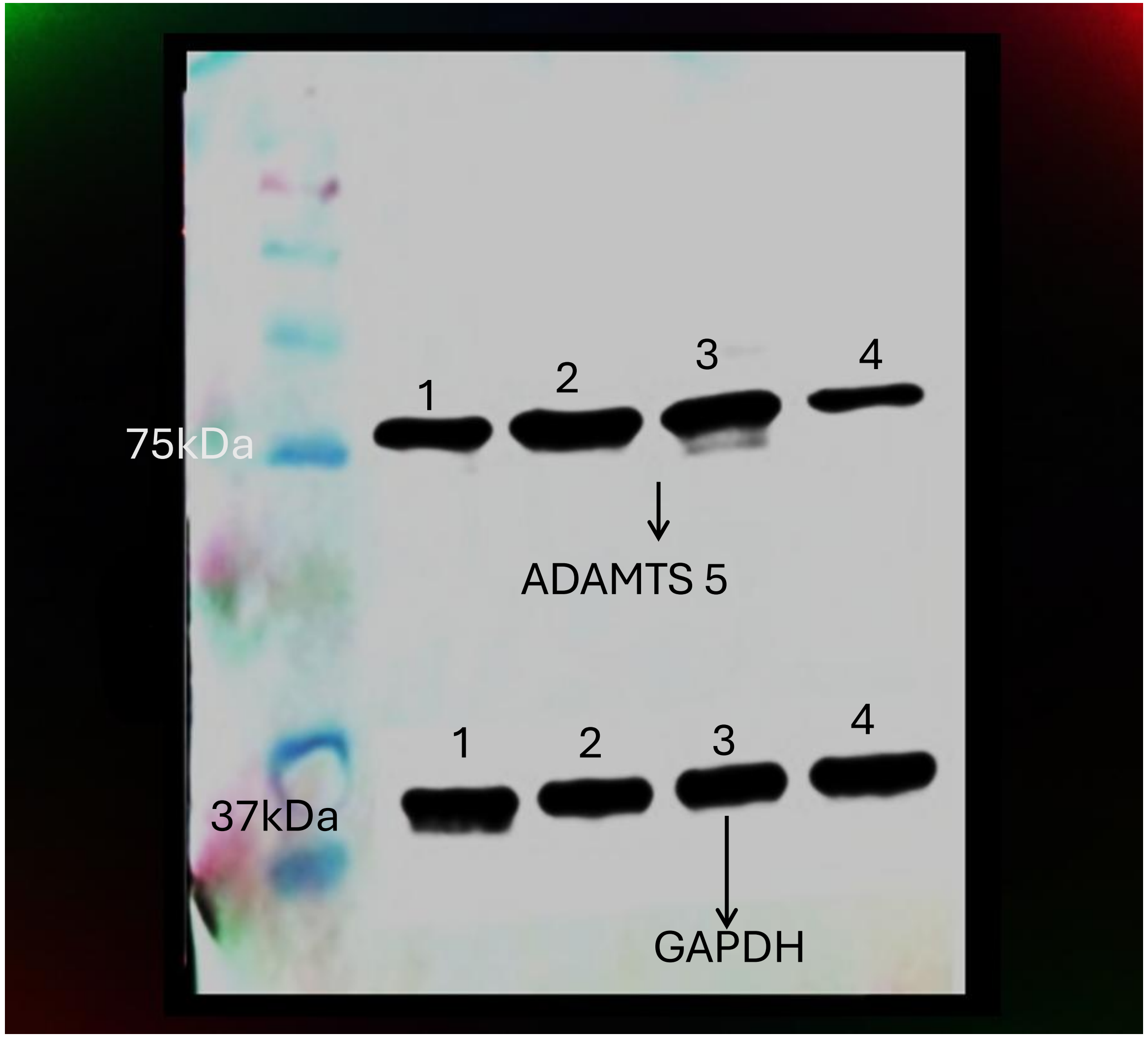

Submitted Blot for ADAMTS 5

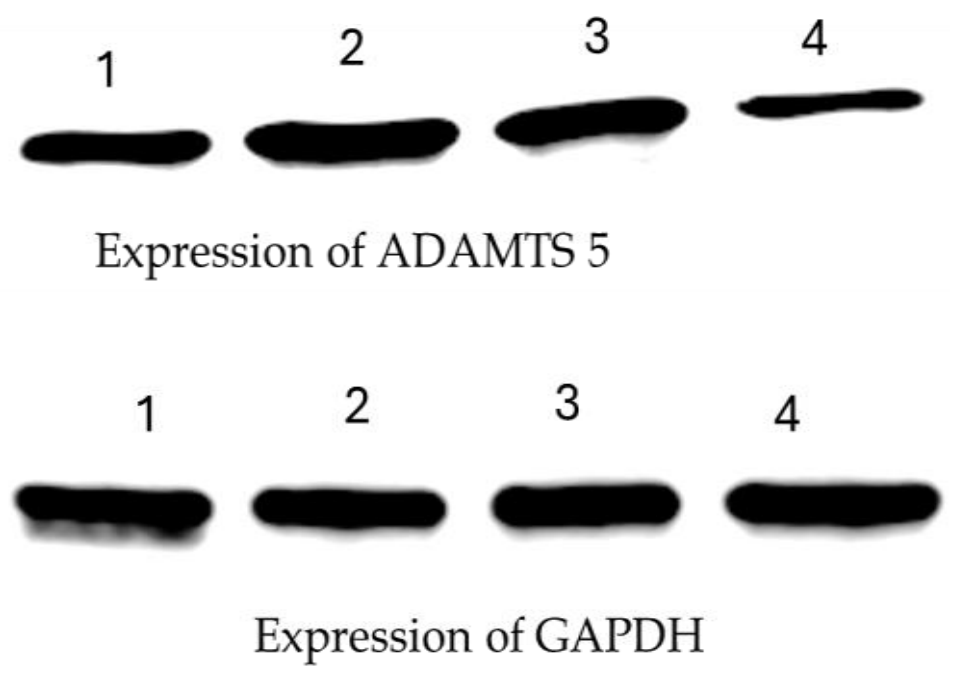

Supplement: Supplementary file 1 [file pone.0357766.s002.pdf]
