# Supplementary material for: Epigallocatechin-3-gallate attenuates PAR-2–mediated inflammatory signaling and preserves markers of mitochondrial integrity in osteoarthritic chondrocytes
Source: PLoS One. 2026 Nov 2;21(11):e0357766. doi: 10.1371/journal.pone.0357766 (PMC13726950; doi:10.1371/journal.pone.0357766)
Supplement: Supplementary file 2 [file pone.0357766.s003.pdf]

Supplementary Information file

Supplementary Information for

Epigallocatechin-3-gallate Attenuates PAR-2–mediated Inflammatory Signaling and Preserves Markers of Mitochondrial Integrity in Osteoarthritic Chondrocytes

*****Rajashree Patnaik and Yajnavalka Banerjee

Contents

Supplementary Figure S1. Flow cytometry gating strategy for RANK and RANKL surface staining.

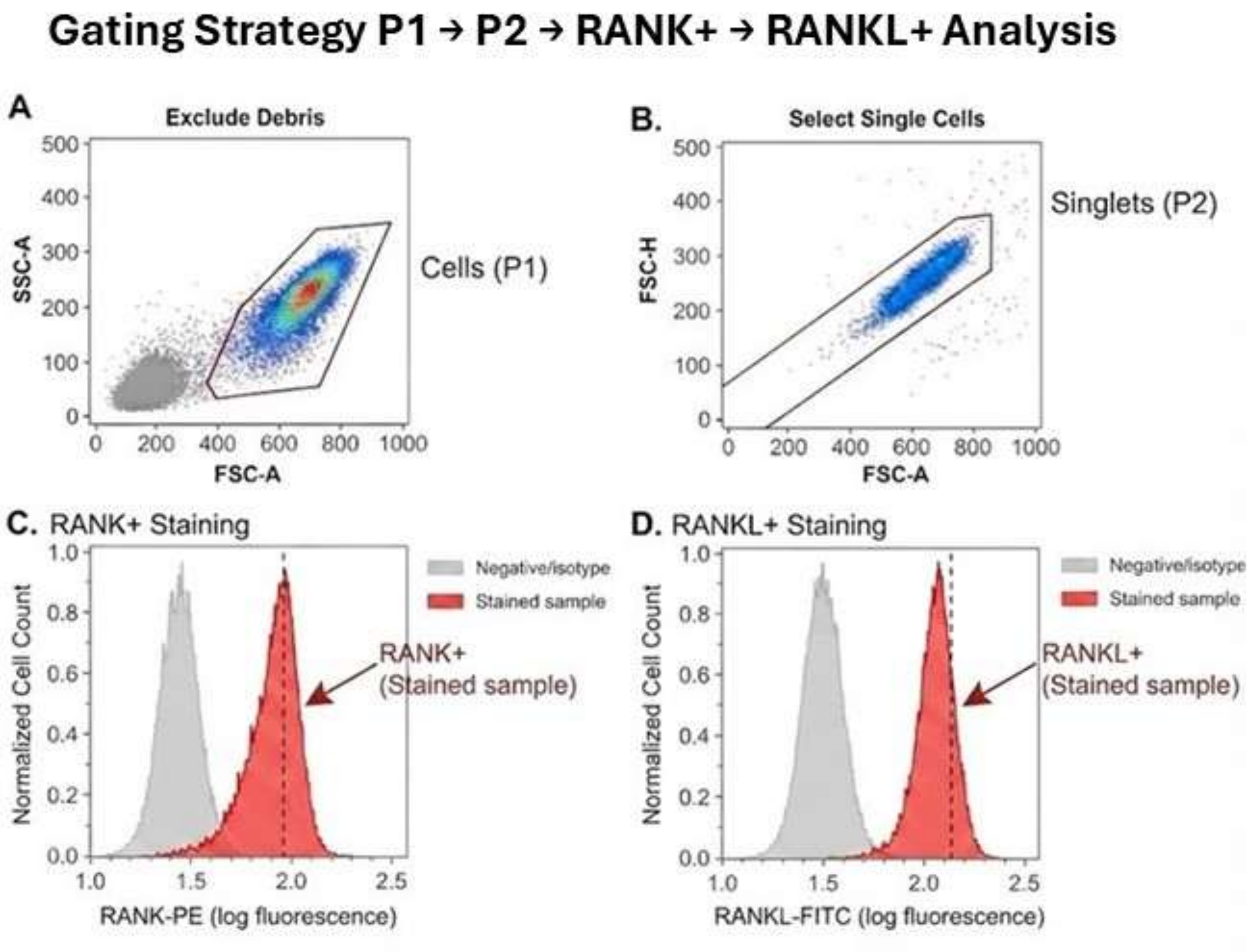

Fig. S1. Flow cytometry gating strategy and fluorescence intensity histograms for RANK and RANKL expression. (A) Initial forward scatter (FSC-A) versus side scatter (SSC-A) dot plot used to exclude cellular debris and gate viable cells (P1). **(B)** Forward scatter area (FSC-A) versus height (FSC-H) plot used to select single cells (Singlets, P2) and eliminate cell doublets. **(C)** Representative histogram showing fluorescence intensity distribution for RANK+ PE staining (red shaded curve) compared to the negative/isotype control (gray shaded curve), with a dashed threshold line indicating positive gating. **(D)** Representative histogram showing fluorescence intensity distribution for RANKL+ FITC staining (red shaded curve) compared to the negative/isotype control (gray shaded curve).
