# Supplementary material for: Epigallocatechin-3-gallate attenuates PAR-2–mediated inflammatory signaling and preserves markers of mitochondrial integrity in osteoarthritic chondrocytes
Source: PLoS One. 2026 Nov 2;21(11):e0357766. doi: 10.1371/journal.pone.0357766 (PMC13726950; doi:10.1371/journal.pone.0357766)
Supplement: Supplementary file 3 [file pone.0357766.s004.pdf]

Supporting Information

Epigallocatechin-3-gallate Attenuates PAR-2–mediated Inflammatory Signalling and Preserves Markers of Mitochondrial Integrity in Osteoarthritic Chondrocytes — Patnaik & Banerjee

PAR 2 for Figure 8A

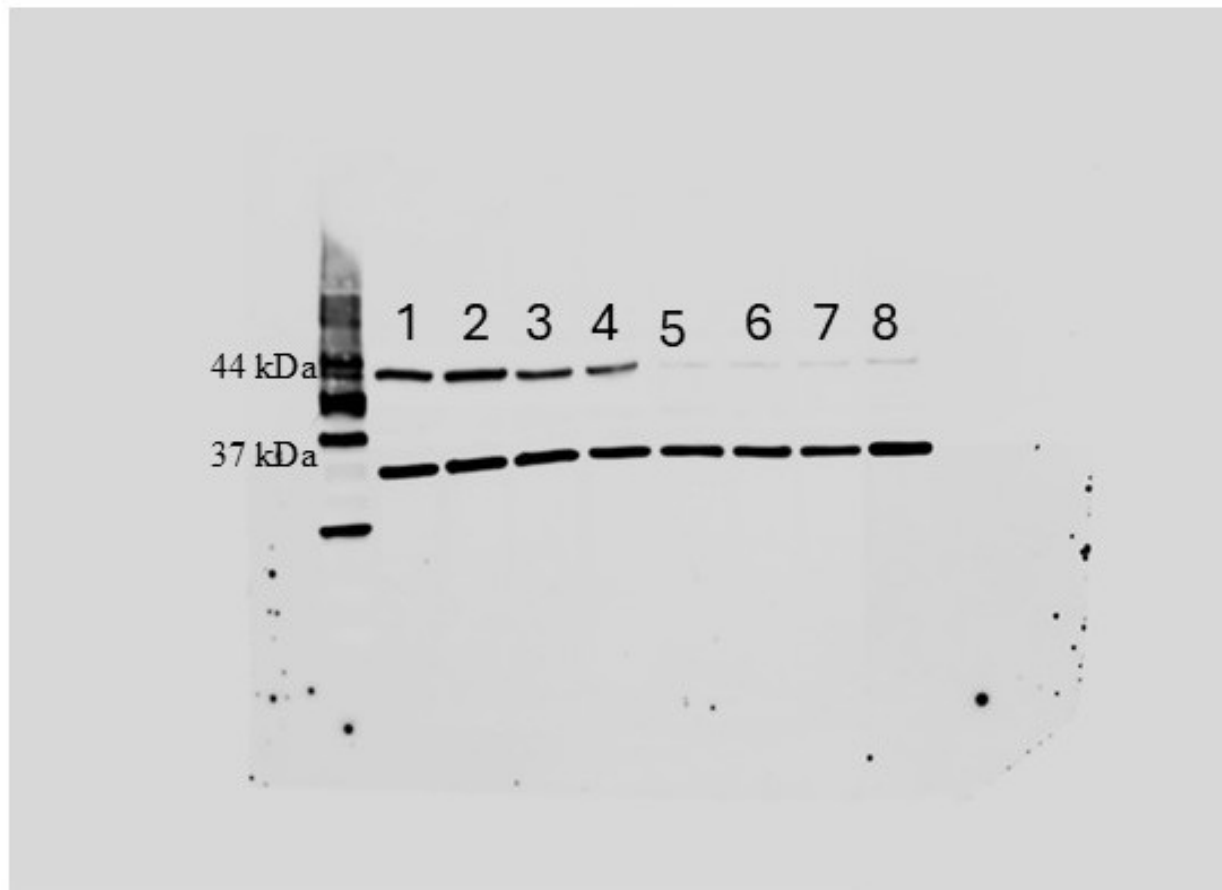

S1 Fig. Full-length, uncropped western blot for Figure 8A (PAR-2).

Full-length, uncropped chemiluminescent western blot from which the cropped panel shown in Figure 8A was prepared. PAR-2 migrates at approximately 44 kDa and the GAPDH loading control at approximately 37 kDa; the positions of the 44 kDa and 37 kDa molecular-weight markers are indicated at the left of the membrane. PAR-2 is strongly expressed in the non-silenced lanes (1–4) and is efficiently ablated in the PAR-2 siRNA–transfected lanes (5–8), while GAPDH remains uniform across all lanes. The lane numbering (1–8) corresponds exactly to that used in the initial submission, as tabulated below.

Lane	Treatment condition
**1**	Chondrocytes (untreated control)
**2**	Chondrocytes + LPS
**3**	Chondrocytes + LPS + 5 µM EGCG
**4**	Chondrocytes + LPS + 10 µM EGCG
**5**	Chondrocytes + PAR-2 siRNA
**6**	Chondrocytes + LPS + PAR-2 siRNA
**7**	Chondrocytes + LPS + PAR-2 siRNA + 5 µM EGCG
**8**	Chondrocytes + LPS + PAR-2 siRNA + 10 µM EGCG

Abbreviations: EGCG, epigallocatechin-3-gallate; LPS, lipopolysaccharide; PAR-2, protease-activated receptor-2; siRNA, small interfering RNA; GAPDH, glyceraldehyde-3-phosphate dehydrogenase.
