# Supplementary material for: Epigallocatechin-3-gallate attenuates PAR-2–mediated inflammatory signaling and preserves markers of mitochondrial integrity in osteoarthritic chondrocytes
Source: PLoS One. 2026 Nov 2;21(11):e0357766. doi: 10.1371/journal.pone.0357766 (PMC13726950; doi:10.1371/journal.pone.0357766)
Supplement: Supplementary file 4 [file pone.0357766.s005.pdf]

Cover letter with Detailed Response to Reviewers

Dear Dr Bhattacharya,

We thank you, and the three reviewers, most sincerely for the time and effort invested in evaluating our manuscript. The comments have been uniformly constructive, and the revised manuscript is, we believe, substantially clearer and more rigorous as a result.

We have considered every point raised, and the great majority of the reviewers' recommendations have been accepted and implemented in full, as documented point by point in the responses that follow and as shown in the tracked-changes manuscript. In a small number of instances we have, after careful deliberation, retained our original approach and provided a detailed scientific justification in place of the requested change. *For the Editor's convenience, these are the only substantive points on which the requested change has not been made, and both arise from Reviewer #3*:

- **Panel order (mRNA versus protein).** Reviewer #3 proposed that the figure panels be reordered so that mRNA precedes protein (Comment 7, with the same point recurring in Comments 12, 13, 14 and 19). We have adopted every other presentational change requested for these figures, but have retained the protein-first ordering, which follows the inferential logic of a results figure, the functional primacy of the cell-surface PAR-2 receptor protein, the well-documented discordance between transcript and protein abundance, and the uniform protein-first convention maintained throughout the manuscript and the Methods. The full rationale is set out in our response under Comment 7 (Section C).

- **Retention of the Discussion summary tables.** Reviewer #3 proposed that the Discussion summary tables be removed (Comments 22 and 23). We have retained Tables 2, 3 and 4 because each is the evidentiary basis of an already-accepted response to a concern raised by another reviewer: Table 4 answers Reviewer #1's principal Major Concern regarding the entirely in vitro design (Comment 1); Table 3 answers Reviewer #1's Major Concern on the physiological achievability of the working concentrations (Comment 5); and Table 2 answers Reviewer #2's request that EGCG be framed as a multifunctional agent in which PAR-2 is predominant but not exclusive (Comment 3). Removing these tables would therefore re-open points already resolved to the satisfaction of Reviewers #1 and #2. Our full reasoning is given in our responses to Comments 22 and 23.

We respond to each reviewer in turn below.

Response to Reviewers

Reviewer #1 — Major Concern

Reviewer Comment 1

The study is entirely based on an in vitro model, which limits its biological and clinical relevance.

Authors' Response

We thank the Reviewer for this important and entirely fair observation, and we agree that the in vitro nature of the model is a genuine limitation that must be stated plainly. We address the concern on two complementary fronts: first, by clarifying the deliberate scope of the study as a proof-of-concept mechanistic investigation; and second, by strengthening the manuscript text so that the in vitro findings are explicitly framed within a structured, multi-level translational evidence hierarchy rather than presented in isolation. We have not introduced any new data or any new table; instead, we have revised the surrounding Discussion text to make the existing translational framework (**Table 4**) carry the argumentative weight that the Reviewer's comment rightly demands.

On the question of scope, the primary and explicitly stated objective of this study was to identify PAR-2 as a novel upstream target of EGCG in osteoarthritic chondrocytes and to delineate the consequent PAR-2→ERK1/2→TNF-α/SOX4/ADAMTS-5 signalling cascade. The dissection of a receptor–kinase signalling axis at this level of mechanistic resolution, incorporating qPCR, Western blot, ELISA, flow cytometry, and, critically, siRNA-mediated PAR-2 silencing as a causal manipulation, is appropriately and necessarily performed in a controlled in vitro system, in which the contribution of the receptor can be isolated from the systemic, biomechanical, and multicellular confounders of the intact joint. The mechanistic validity of this objective is independent of the route through which therapeutically effective chondrocyte concentrations are ultimately achieved in vivo.

On the question of biological and clinical relevance, we fully accept that a mechanistic in vitro result, taken alone, cannot establish translational significance. For this reason the manuscript already contained Table 4, which collates the supporting evidence across three hierarchically ordered levels of biological fidelity: animal in vivo studies (*the Dunkin-Hartley spontaneous-OA guinea pig, the DMM mouse, and the MIA rat models*), human ex vivo studies in primary chondrocytes isolated from the cartilage of OA patients, and human patient data (*a prospective cohort of 11,091 individuals and the only randomised controlled trial of green tea extract in knee OA to date*). Each level corroborates specific effectors of the cascade characterised in our in vitro work, for example, in vivo ADAMTS5 suppression corroborates our SOX4–ADAMTS-5 finding, and the suppression of TNF-α and MCP-1 in primary human OA chondrocytes corroborates our ELISA and RT-PCR endpoints.

We recognise, however, that in the previous version the role of **Table 4** as the direct answer to the in vitro limitation was not made sufficiently explicit at the points in the Discussion where the Reviewer would expect to find it. We have therefore made two targeted textual revisions, shown as tracked changes in the revised manuscript, that foreground Table 4 as the structured bridge between our mechanistic findings and their broader biological and clinical relevance. We have deliberately kept Table 4 in its existing position and have not altered its content.

Specific Revisions Made (tracked changes in the revised manuscript)

The two revisions below are the only changes introduced in response to this comment. Both are visible as insertions attributed in the tracked-changes layer of the revised manuscript.

1. **Discussion — lead-in to Table 4 (translational evidence paragraph).** A framing sentence has been inserted at the start of the paragraph that introduces the multi-level evidence hierarchy, so that

the paragraph now opens by conceding the in vitro design and immediately positioning Table 4 as the corroborating framework. The inserted text reads:

INSERTED TEXT (DISCUSSION)
"We acknowledge that the mechanistic cascade characterised in the present work was, by design, dissected in an in vitro chondrocyte system; however, these findings should not be regarded as an isolated cell-culture phenomenon."
This sentence is placed immediately before the existing sentence beginning "The translational significance of the mechanistic cascade delineated in the present study is substantiated by a converging body of evidence … which we summarise in Table 4."

2. **Limitations — explicit in vitro statement with cross-reference to Table 4.** An explicit limitation statement has been inserted at the head of the Limitations paragraph, immediately after "Several limitations warrant consideration." This owns the in vitro limitation directly, cross-references Table 4 as the translational framework, and reaffirms that purpose-designed in vivo PAR-2 studies are the required next step. The inserted text reads:

INSERTED TEXT (LIMITATIONS)
"Foremost, the present investigation is, by design, an in vitro mechanistic study, and as such it does not fully recapitulate the systemic and joint-level pharmacokinetic, biomechanical, and multicellular complexity of the osteoarthritic joint in vivo, nor the physiological conditions that govern EGCG bioavailability and tissue distribution. To situate the present findings within the wider translational landscape rather than to overstate their immediate clinical reach, we have collated the available animal in vivo, human ex vivo, and human patient evidence into a structured hierarchical framework (Table 4); this framework demonstrates that the PAR-2–ERK1/2–TNF-α/SOX4/ADAMTS-5 cascade dissected here is concordant with, and corroborated by, evidence at progressively higher levels of biological fidelity. We nonetheless emphasise that this contextual evidence is not a substitute for direct in vivo interrogation, and that purpose-designed in vivo studies targeting the PAR-2–ERK1/2 axis remain the essential next step before clinical inferences can be drawn."

Summary

Taken together, these two revisions ensure that the in vitro limitation is acknowledged transparently at the outset, that **Table 4** is explicitly invoked as the structured evidence hierarchy bridging the in vitro mechanism to animal in vivo, human ex vivo, and human patient data, and that the necessity of purpose-designed in vivo PAR-2 studies is restated as the immediate translational priority. We believe these changes directly and substantively address the Reviewer's concern regarding the biological and clinical relevance of the work, and we are grateful for the comment, which has improved the clarity and balance of the Discussion.

Reviewer Comment 2

The sample size (n = 3 biological replicates) is insufficient to ensure robustness and reproducibility.

Authors' Response

We thank the Reviewer for raising the question of sample size, which gives us a welcome opportunity to clarify the structure and rigour of our experimental design. We respectfully submit that the use of three independent biological replicates is not a limitation of robustness in this study, but rather the established and statistically appropriate standard for hypothesis-driven in vitro mechanistic work of this kind. We address the concern below and have added a clarifying statement, with supporting references, to the Statistical analysis section of the revised manuscript (shown as tracked changes).

First, and most importantly, we wish to clarify what n = 3 denotes in our study. The three replicates are genuine biological replicates, not technical replicates. Each replicate corresponds to a fully independent bone-marrow-derived mesenchymal stem cell culture that was separately established, chondrogenically differentiated, treated, and assayed on a different occasion. This distinction is decisive, because it is biological replication, defined as independent measurements of the population of interest from separate experiments, that constitutes the legitimate unit of statistical inference in cell-culture research. This principle is set out explicitly in the methodological literature on experimental design, including the widely cited guidance of Naegle, Gough and Yaffe in Science Signaling, Lazic, Clarke-Williams and Munafo in PLOS Biology, and Lord and colleagues in the Journal of Cell Biology. A study reporting three independent biological replicates is therefore reporting a true n of 3, in contrast to studies that inflate n by counting wells, cells, or technical repeats of a single culture, a practice these authors identify as pseudoreplication.

Second, three independent biological replicates represent the conventional minimum for this category of in vitro mechanistic study and are consistent with the prevailing standard in the chondrocyte and osteoarthritis signalling literature, including several of the human chondrocyte studies cited in the present manuscript. We respectfully note that our analytical approach was, in fact, designed conservatively: comparisons were restricted to three pre-specified, hypothesis-driven contrasts anchored to a single LPS-stimulated reference group, and the statistical framework is equivalent to one-way ANOVA with Dunnett correction against a control. The design was therefore not an unstructured search for significance across many comparisons, but a focused test of a small number of a priori hypotheses, for which n = 3 biological replicates is well suited.

Third, we would emphasise that robustness in a mechanistic study of this nature derives not from replicate count alone, but from the convergence of independent lines of evidence. The central conclusion, that EGCG suppresses the PAR-2–ERK1/2–TNF-α/SOX4/ADAMTS-5 axis, is supported concordantly by five methodologically orthogonal techniques: quantitative PCR, Western blotting, ELISA, flow cytometry, and, critically, siRNA-mediated loss-of-function. The effects are reproducible and dose-dependent across the 5 and 10 μM conditions, the effect sizes are large (for example, 81.9% suppression of SOX4 and 66.2% of ADAMTS-5 protein at 10 μM), and the directionality is internally consistent at both transcript and protein levels. This multi-modal convergence provides a substantially stronger basis for inference than would any single assay performed at higher replicate number, and it directly addresses the Reviewer's underlying concern about reproducibility.

Taken together, we are confident that the design is statistically sound and that the conclusions are well supported. To make this reasoning explicit for the reader, we have revised the Statistical analysis section as described below and have cited the relevant methodological literature.

Specific Revisions Made (tracked changes in the revised manuscript)

The following change is the only addition made in response to this comment and is shown as a tracked insertion in the revised manuscript.

1. **Statistical analysis section — clarification of biological replication with supporting references.** A sentence has been inserted that defines n = 3 as independent biological replicates (the unit of statistical inference), affirms that this is the conventional minimum for hypothesis-driven in vitro mechanistic studies, and notes that robustness is reinforced by orthogonal multi-method convergence, dose-dependence, and large effect sizes. The inserted text reads:

INSERTED TEXT (STATISTICAL ANALYSIS)

"For each assay, n = 3 denotes three independent biological replicates, defined as fully separate chondrocyte cultures established, differentiated, treated, and assayed on different occasions, rather than technical replicates of a single culture; this constitutes the unit of statistical inference in accordance with established guidance on what validly constitutes 'n' in cell-culture experiments [1, 2]. Three independent biological replicates represent the conventional minimum for hypothesis-driven in vitro mechanistic studies, and the robustness of the present conclusions is further reinforced by the convergence of orthogonal, methodologically independent readouts (qPCR, immunoblotting, ELISA,

flow cytometry, and siRNA-mediated loss of function), by the reproducible, dose-dependent nature of the responses, and by the large effect sizes obtained, which collectively provide a stronger basis for inference than replicate count considered in isolation [3].”

References Added

Three methodological references have been added to the reference list).

1. Naegle K, Gough NR, Yaffe MB. Criteria for biological reproducibility: what does “n” mean? Sci Signal. 2015;8(371):fs7. doi: 10.1126/scisignal.aab1125. PMID: 25852186.
2. Lazic SE, Clarke-Williams CJ, Munafo MR. What exactly is ‘N’ in cell culture and animal experiments? PLoS Biol. 2018;16(4):e2005282. doi: 10.1371/journal.pbio.2005282. PMID: 29617358; PMCID: PMC5902037.
3. Lord SJ, Velle KB, Mullins RD, Fritz-Laylin LK. SuperPlots: Communicating reproducibility and variability in cell biology. J Cell Biol. 2020;219(6):e202001064. doi: 10.1083/jcb.202001064. PMID: 32346721; PMCID: PMC7265319.

Reviewer Comment 3

The statistical analysis is not appropriate, as multiple t-tests were used instead of one-way ANOVA with proper post hoc correction.

Authors’ Response

We thank the Reviewer for this constructive observation. We accept the recommendation in full and have revised the statistical analysis accordingly. Group differences are now evaluated by one-way analysis of variance (ANOVA) followed by Dunnett’s multiple-comparison post-hoc test, rather than by independent t-tests. We have updated the Statistical analysis section of the Methods and the affected figure legends to reflect this change, and we have added a supporting methodological reference. The relevant edits are shown as tracked changes in the revised manuscript.

We selected Dunnett’s post-hoc test specifically because the experimental design compares several treatment conditions (untreated control, LPS, LPS + EGCG 5 μM, and LPS + EGCG 10 μM) against a single common reference, the LPS-stimulated group. Dunnett’s procedure is the post-hoc test designed precisely for this many-treatments-versus-one-control structure, and it controls the family-wise error rate across the set of comparisons. Where an investigator wishes instead to compare every group with every other group, Tukey’s honestly significant difference test would be the appropriate alternative; because our hypotheses are anchored to the LPS reference rather than to all possible pairwise contrasts, Dunnett’s test is both the most appropriate and the most statistically powerful choice for this design. The significance thresholds ($p < 0.05$ and $p < 0.01$) and the GraphPad Prism analysis environment are unchanged.

We note for completeness that the magnitude and direction of the reported effects are robust to this change in analytical approach: the dose-dependent suppression of the PAR-2–ERK1/2–TNF-α/SOX4/ADAMTS-5 axis remains statistically significant under ANOVA with Dunnett’s correction. The revised analysis therefore strengthens the rigour of the evaluation without altering the study’s conclusions.

Specific Revisions Made (tracked changes in the revised manuscript)

The following edits were made in response to this comment and are shown as tracked changes. The previously added statement on biological replication (Statistical analysis section) was left unchanged.

1. **Statistical analysis section — method replaced.** The description of Student’s two-tailed unpaired t-tests was deleted and replaced with one-way ANOVA followed by Dunnett’s multiple-comparison post-hoc test. The revised passage now reads:

INSERTED TEXT (STATISTICAL ANALYSIS)
"Group differences were evaluated by one-way analysis of variance (ANOVA) across the four treatment conditions (untreated control, LPS, LPS + EGCG 5 µM, and LPS + EGCG 10 µM), followed by Dunnett's multiple-comparison post-hoc test, in which each treatment condition was compared against the common LPS-stimulated reference group: (i) untreated chondrocytes vs. LPS; (ii) LPS vs. LPS + EGCG 5 µM; and (iii) LPS vs. LPS + EGCG 10 µM. Although it has been argued that pre-planned comparisons defined a priori against a single biological reference do not strictly require family-wise correction [Rothman], we nonetheless adopted Dunnett's procedure to provide a conservative and widely accepted evaluation of group differences, as it is specifically designed for the comparison of several treatment groups against a single control and controls the family-wise error rate [Dunnett], in keeping with established recommendations for handling multiple comparisons in biological research [Curran-Everett]."
Citation numbers in the manuscript correspond to Rothman = [32], Dunnett = [33], and the newly added Curran-Everett reference (see Reference Added below).

2. **Figure legends — Figure legends — statistical test updated.** In the legends to Figures 2, 3, 5, and 6, the parenthetical "(Student's two-tailed unpaired t-test)" was replaced with "(one-way ANOVA with Dunnett's post-hoc test)." The legends to Figures 4, 7, and 8, which report significance thresholds without naming the test, remain consistent with the revised analysis and were left unchanged.

Figure legends — p-value updates

Following re-analysis under the one-way ANOVA with Dunnett's post-hoc framework, the reported significance values in the figure legends were updated. The change is twofold: (i) the significance key in every quantified figure (Figs 2–8) was extended from a two-tier scheme to a three-tier scheme by adding the $p < 0.001$ (***) level; and (ii) three specific dose-level statistics were strengthened to the values below. No effect sizes, means, or standard deviations were changed; only the p-value annotations were updated to match the re-analysis.

Significance key (all quantified figures, Figs 2–8):

SIGNIFICANCE KEY — FIGS 2–8
Was: ~~*p < 0.05, **p < 0.01 versus LPS-only group~~
Now: **p < 0.05, **p < 0.01, ***p < 0.001 versus LPS-only group*

Specific dose-level statistics updated:

FIG 2 — PERK1/2 : TOTAL ERK1/2 RATIO
Was: ~~…driving the ratio below basal levels (p < 0.05 at 5 µM; p < 0.01 at 10 µM).~~
Now: *…driving the ratio below basal levels (p < 0.01 at 5 µM; p < 0.001 at 10 µM).*

FIG 5 — SOX4 PROTEIN EXPRESSION
Was: ~~…reduced by 41.1% and 81.9% upon treatment with 5 µM and 10 µM EGCG, respectively (p < 0.01 at 10 µM).~~

Now: *...reduced by 41.1% and 81.9% upon treatment with 5 μM and 10 μM EGCG, respectively (p < 0.01 at 5 μM and p < 0.001 at 10 μM).*

FIG 5 — ADAMTS-5 PROTEIN EXPRESSION

Was: ~~…a 22.0% and 66.2% reduction at 5 μM and 10 μM, respectively; p < 0.01).~~

Now: *...a 22.0% and 66.2% reduction at 5 μM and 10 μM, respectively, with statistical significance of P < 0.01 and P < 0.001 at the corresponding concentrations).*

*Note: in the current draft two of the inserted significance keys contain minor typographical slips, "***< 0.001" (missing the italic p) in the Fig 2 key and "***p <0.001" (missing a space) in the Fig 6 key. These should be corrected to "***p < 0.001" for consistency before submission.*

Reference Added

One methodological reference has been added (numbered accordingly, continuing the existing sequence in the manuscript).

1. Curran-Everett D. Multiple comparisons: philosophies and illustrations. Am J Physiol Regul Integr Comp Physiol. 2000;279(1):R1-8. doi: 10.1152/ajpregu.2000.279.1.R1. PMID: 10896857.

Summary

In direct response to the Reviewer, the statistical analysis has been changed from multiple t-tests to one-way ANOVA with Dunnett's post-hoc correction throughout the Methods and figure legends, with an accompanying supporting reference. This provides a more rigorous and conventionally accepted evaluation of group differences while leaving the study's conclusions intact. We are grateful for the Reviewer's input, which has improved the statistical rigour of the manuscript.

Reviewer Comment 4

The use of LPS as a model for osteoarthritis inflammation remains artificial and should be more critically discussed.

Authors' Response

We thank the Reviewer for this thoughtful comment, and we agree that the choice of inflammatory stimulus warrants careful and balanced discussion. We respond in two parts. First, we summarise the mechanistic rationale for LPS as an osteoarthritis-relevant stimulus, which is already set out in the Methods and which we have reinforced with one additional element. Second, and in direct response to the Reviewer's call for more critical discussion, we have substantially expanded the Limitations to acknowledge, candidly and specifically, what the LPS model does not capture.

1. The mechanistic rationale for LPS is OA-specific, not generic (stated in the Methods and developed in the Discussion)

We respectfully note that the manuscript does not defend LPS on grounds of common usage. The Methods already articulate an OA-specific, receptor-level rationale, which we briefly restate here for the Reviewer's convenience. The endogenous damage-associated molecular patterns (DAMPs) that accumulate in the osteoarthritic joint, including fibronectin fragments, HMGB1, tenascin-C, hyaluronan oligosaccharides, and S100A8/A9, signal predominantly through Toll-like receptor 4 (TLR4), whose expression is elevated in OA cartilage in proportion to histological severity. Because the intracellular cascade downstream of TLR4 (MyD88 → IRAK1/4 → TRAF6 → TAK1 → IKKβ → NF-κB and the parallel MAPK arms) is determined by the adaptor architecture rather than by the identity of the extracellular ligand, LPS, as the

canonical high-affinity TLR4 ligand, reproduces DAMP-driven TLR4 signalling with high receptor fidelity. LPS therefore models the molecular receptor that the OA joint itself exploits, rather than bacterial infection per se.

This rationale is particularly apposite to the present study because our central question concerns PAR-2 induction. PAR-2 transcription is governed by NF-κB response elements in the F2RL1 promoter, and the TLR4 → NF-κB axis is the principal upstream inductive pathway for PAR-2 expression in chondrocytes. Consistent with this, LPS produced an approximately four-fold increase in PAR-2 mRNA in our model (Fig. 2C). LPS is thus the stimulus that engages the authentic transcriptional pathway by which PAR-2 is induced in DAMP-stimulated OA cartilage.

To strengthen the in-vivo relevance of this paradigm, we have added a sentence and supporting reference on metabolic endotoxaemia, the chronic low-grade elevation of circulating LPS arising from gut-barrier dysfunction in obesity, which sustains systemic and synovial TLR4-driven inflammation. This establishes LPS not as an artificial trigger but as a biologically authentic stimulus for the metabolically driven OA phenotype that represents a large share of the contemporary OA population.

2. We have substantially expanded the critical discussion of the model's limitations

In direct response to the Reviewer's request, we have added an explicit and specific acknowledgement of what the LPS model does not capture. The expanded Limitations now state that (i) LPS engages only the extracellular TLR4–MD-2–CD14 complex, whereas endogenous DAMPs additionally signal through intracellular pattern-recognition receptors, including the NLRP3 inflammasome, and through integrins such as α5β1 and αvβ3; (ii) mechanical-loading–induced inflammation, transduced by distinct mechanosensors including PIEZO1/2 channels, is not modelled by a soluble chemical stimulus; and (iii) the present monoculture does not recapitulate the chondrocyte–synoviocyte–macrophage crosstalk that shapes the inflammatory response in vivo. We have framed these as priorities for multi-stimulus, co-culture, and in vivo models in future work.

Taken together, we believe the revised manuscript now presents a balanced position: a substantive, OA-specific justification for the stimulus chosen, paired with a candid and specific account of its limitations. We are grateful to the Reviewer, whose comment has improved the critical balance of both the Methods and the Discussion.

Specific Revisions Made (tracked changes in the revised manuscript)

Two additions were made in response to this comment, shown as tracked changes. No existing text was deleted.

1. **Discussion (LPS–TLR4 rationale subsection) — metabolic-endotoxaemia sentence added.** The following sentence was inserted within the dedicated Discussion subsection "Rationale for the LPS–TLR4 model as a surrogate for DAMP-driven inflammation in osteoarthritis":

INSERTED TEXT (DISCUSSION)
"This systemic source of TLR4 ligand is itself mechanistically grounded: metabolic endotoxaemia, the chronic low-grade elevation of circulating LPS arising from gut-barrier dysfunction in obesity, sustains systemic and synovial TLR4-driven inflammation and provides a biologically authentic, rather than artificial, basis for LPS as a model stimulus of the metabolically driven OA phenotype that constitutes a substantial share of the contemporary OA population [Cani]."

2. **Discussion (Limitations) — LPS-model concessions added.** The following passage was inserted immediately after the existing LPS limitation sentence:

INSERTED TEXT (LIMITATIONS)
"We further acknowledge that the LPS–TLR4 stimulus does not capture the full breadth of innate immune activation operative in the osteoarthritic joint. First, LPS engages the extracellular TLR4–MD-2–CD14 complex only, whereas endogenous DAMPs additionally signal through intracellular pattern-recognition receptors, including the NLRP3 inflammasome, and through integrins such as α5β1 and

αvβ3, pathways that LPS stimulation does not reproduce. Second, mechanical-loading–induced inflammation, which is transduced by distinct mechanosensing machinery including PIEZO1/2 channels and integrin-mediated signalling, is not modelled by a soluble chemical stimulus. Third, the present monoculture does not recapitulate the cellular ecology of the OA joint, in particular the reciprocal crosstalk among chondrocytes, synoviocytes, and macrophages that shapes the inflammatory response in vivo. These constraints temper the interpretation of the present findings and define clear priorities for multi-stimulus, co-culture, and in vivo models in future work."

Reference Added

One reference was added (continuing the existing sequence in the manuscript).

1. Cani PD, Amar J, Iglesias MA, Poggi M, Knauf C, Bastelica D, et al. Metabolic endotoxemia initiates obesity and insulin resistance. Diabetes. 2007;56(7):1761-72. doi: 10.2337/db06-1491. PMID: 17456850.

Summary

In response to this comment we have (i) reinforced the OA-specific mechanistic rationale for LPS, now developed in a dedicated Discussion subsection, with a metabolic-endotoxaemia argument and supporting reference, and (ii) substantially expanded the Limitations to provide the candid, specific critical discussion of the model that the Reviewer requested. The revised manuscript now balances a defensible justification for the stimulus with a transparent account of what it does not model

---

Reviewer Comment 5

The claim that the tested EGCG concentrations are physiologically achievable requires stronger supporting evidence.

Authors' Response

We thank the Reviewer for this fair challenge, which has prompted us to make our claim more precise and better supported. On reflection, we recognise that the phrase "physiologically achievable" conflates two distinct quantities, the concentration of EGCG delivered to the gastrointestinal lumen, and the concentration ultimately attained at the chondrocyte. We have therefore revised the Discussion to (i) present the per-variety evidence in Table 3 in full, framed explicitly as the delivered (luminal) dose, and (ii) reconcile this with the systemic pharmacokinetic reality and the delivery strategies that bridge the two. We believe the revised text supports a defensible, internally consistent position rather than an overstated one.

1. Table 3 establishes that the delivered dose vastly exceeds the experimental concentrations

Table 3 catalogues EGCG content across 36 commercially consumed green teas, and the revised Discussion now draws on the full breadth of that table rather than two exemplars. The upper tier (Gyokuro and superior Sencha infusions) delivers approximately 676 to 2,705 µM EGCG per serving; traditionally processed Chinese Lu'an Guapian (1,444 to 2,014 µM) and the Korean Woojeon, Sejak, Joongjak, and Daejak grades (1,890 to 2,053 µM) occupy a similar range; and even the more modestly concentrated steamed and pan-fired Jeju, Boseong, and Shizuoka teas yield 367 to 769 µM per serving. Across every entry in Table 3, the delivered dose therefore exceeds the 5 to 10 µM concentrations used in our experiments by between one-and-a-half and two-and-a-half orders of magnitude. The limiting factor in translation is thus not dietary intake but intestinal absorption.

2. We have made the luminal-versus-systemic distinction explicit, and reconciled it with the pharmacokinetics

We are careful not to claim that a single cup of tea produces 5 to 10 µM EGCG in plasma; indeed, the manuscript's own pharmacokinetic analysis states that systemic plasma concentrations after conventional

single-serving consumption are submicromolar. The revised text now states explicitly that the Table 3 values represent the concentration presented to the intestinal lumen and mucosa, that only a small fraction of luminal catechin is absorbed intact, and that the gap between luminal exposure and effective tissue concentration is therefore an absorption-and-delivery problem. This honest framing strengthens the claim by aligning it precisely with the pharmacokinetic data already presented, rather than contradicting them.

3. Pharmacokinetic and retention strategies bridge the luminal-to-tissue gap

The revised Discussion connects this argument to the delivery strategies already described in the manuscript: enhanced-bioavailability formulations (phospholipid-complexed phytosomes achieving 2- to 4-fold higher plasma Cmax) and joint-targeted approaches (intra-articular administration and nanoparticle encapsulation with demonstrated synovial retention and cartilage penetration). Together, these establish a credible pharmacological route by which therapeutically effective EGCG concentrations can be attained at the chondrocyte, notwithstanding the modest systemic bioavailability of unformulated EGCG. The newly added review by Cai et al. supports both halves of this argument: that only a small fraction of luminal catechin is absorbed, and that nanostructure-based and related delivery systems materially improve bioavailability.

Taken together, the revised manuscript replaces a single, potentially over-broad claim with a graded, evidence-anchored argument: the delivered dose is unambiguously sufficient (Table 3); the bottleneck is absorption (now stated explicitly and referenced); and that bottleneck is addressable through established delivery and retention strategies. We are grateful to the Reviewer, whose comment has materially improved both the rigour and the internal consistency of this section.

Specific Revisions Made (tracked changes in the revised manuscript)

Table 3 was retained without modification. The following change was made; no existing text was deleted.

1. **Discussion (Table 3 paragraph) — per-variety evidence and luminal/absorption framing added.** The following passage was inserted immediately after the existing sentence ending "…the 5–10 µM range used in our experiments.":

INSERTED TEXT (DISCUSSION)

"Indeed, the magnitude of this margin is consistent across the full range of varieties catalogued in Table 3: at the upper end, Gyokuro and superior Sencha infusions deliver approximately 676 to 2,705 µM EGCG per serving, while traditionally processed Chinese Lu'an Guapian (1,444 to 2,014 µM) and the Korean Woojeon, Sejak, Joongjak, and Daejak grades (1,890 to 2,053 µM) occupy a similar tier; even the more modestly concentrated steamed and pan-fired Jeju, Boseong, and Shizuoka teas yield 367 to 769 µM per serving. Across every entry in Table 3, therefore, the EGCG dose delivered to the gastrointestinal lumen exceeds the 5 to 10 µM concentrations used here by between one-and-a-half and two-and-a-half orders of magnitude. We emphasise that these values represent the concentration presented to the intestinal lumen and mucosa rather than the systemic plasma concentration; because only a small fraction of luminal catechin is absorbed intact, the limiting step in translation is intestinal absorption and first-pass metabolism, not dietary intake [Cai]. This distinction is mechanistically important and is reconciled, below, with the systemic pharmacokinetic data and with the enhanced-bioavailability and joint-targeted delivery strategies that bridge the gap between luminal exposure and effective chondrocyte concentrations."

Reference Added

One review was added to support the luminal-exposure and bioavailability-improvement argument (continuing the existing sequence in the manuscript).

1. Cai ZY, Li XM, Liang JP, Xiang LP, Wang KR, Shi YL, et al. Bioavailability of Tea Catechins and Its Improvement. Molecules. 2018;23(9):2346. doi: 10.3390/molecules23092346. PMID: 30217074; PMCID: PMC6225109.

Summary

In response to this comment, we have, while retaining Table 3 unchanged, expanded the Discussion to present the per-variety delivered-dose evidence in full and to frame it honestly as luminal rather than plasma exposure, with the absorption bottleneck explicitly stated and addressed through established delivery and retention strategies. The revised claim is graded and internally consistent with the manuscript's own pharmacokinetic analysis.

Reviewer #1 — Minor Concerns

We thank the Reviewer for these constructive minor comments, which we address individually below. All changes are shown as tracked changes in the revised manuscript, and no references were removed.

Minor Concern 1

The manuscript is overly verbose in several sections and would benefit from more concise writing.

Authors' Response

We agree, and we have addressed this on two fronts. First, recognising that the Discussion was the densest section and that a closely related concern was raised by Reviewer #3, we have introduced descriptive subheadings that partition the Discussion into clearly signposted thematic units. This imposes navigable structure without removing any content or references. The six thematic subheadings added, alongside the existing Limitations subsection, are:

1. *PAR-2 is the principal upstream target of EGCG in osteoarthritic chondrocytes*
2. *Coordinated suppression of downstream inflammatory, catabolic, and osteoclastogenic effectors*
3. *Preservation of mitochondrial integrity*
4. *Physiological and pharmacokinetic considerations*
5. *Selection of the LPS and EGCG concentrations: physiological versus supraphysiological context*
6. *Translational context across animal, human-tissue, and clinical evidence*
7. *Limitations*

Second, we have tightened the two densest passages by replacing verbose, partially redundant sentences with more economical technical phrasing, again without loss of substance or citations. Specifically, the sentence describing the relationship between mitochondrial membrane potential and ATP synthesis was condensed, and the opening of the paragraph on PAR-2-independent activity was shortened to remove a verbatim restatement of cytokine values already given in the preceding paragraph.

Minor Concern 2

Some figure descriptions require clearer explanation.

Authors' Response

We have clarified the three figure legends whose descriptions were most terse, adding a brief interpretive statement to each so that the reader can grasp the purpose and result of the experiment without cross-referencing the main text:

1. Figure 4 (cytokines): the title now states that the figure demonstrates concentration-dependent, transcriptionally driven repression of the three principal pro-inflammatory mediators in LPS-stimulated chondrocytes.

2. Figure 7 (mitochondria): a sentence was added explaining that Rhodamine 123 reports mitochondrial membrane potential ($\Delta\Psi m$) and Janus Green B reports mitochondrial mass, and that LPS reduces both while EGCG restores them dose-dependently.
3. Figure 8 (siRNA): a sentence was added explaining that comparison of non-silenced (conditions 1–4) with PAR-2–silenced (conditions 5–8) chondrocytes shows PAR-2 knockdown lowers LPS-induced TNF-α and markedly reduces EGCG's further effect, identifying PAR-2 as the predominant but not exclusive target.

Minor Concern 3

The discussion overstates the translational significance of the findings.

Authors' Response
We have moderated the language at the points where the translational claim was strongest, so that the text now reflects mechanistic plausibility and supportive context rather than demonstrated clinical effect. The substantive edits were:

4. In the Conclusions, "the physiological attainability of effective concentrations (Table 3) … positions EGCG as a multifunctional candidate" was revised to refer to the high delivered (dietary) dose relative to the effective in vitro range, and to state that this "supports the candidacy" of EGCG rather than asserting attainable plasma concentrations.
5. In the Discussion, "lending translational credibility to our findings" was softened to "supporting the translational plausibility of our findings, while systemic delivery remains the key constraint."
6. "Provides a substantive translational framework situating the PAR-2 … cascade" was changed to "provides supportive translational context for the PAR-2 … cascade."

These changes preserve the evidentiary content of the Discussion (including Table 4) while ensuring the framing is proportionate to in vitro mechanistic findings that still require in vivo validation.

Summary
In response to the minor concerns we have (1) restructured the Discussion with six thematic subheadings and condensed its two densest passages, addressing the verbosity noted by Reviewers #1 and #3; (2) clarified the legends of Figures 4, 7, and 8; and (3) moderated the translational language at its strongest points. No references were removed. We are grateful for these suggestions, which have improved the readability and calibration of the manuscript.

Reviewer #2 —Concerns

We thank Reviewer #2 for the encouraging assessment of the work, and in particular for recognising the siRNA validation as elevating the study from correlative to causative. We address Comment 1 below.

Reviewer Comment 1

Sensitivity Analysis: While your pairwise t-test approach is justified, please verify if results maintain significance under a standard one-way ANOVA with Dunnett's post-hoc test to ensure the robustness of the findings across common statistical frameworks.

Authors' Response

We thank the Reviewer for this suggestion, which aligns closely with a point also raised by Reviewer #1 (Comment 3). We are pleased to confirm that this analysis has already been performed and incorporated into the revised manuscript. The statistical approach has been changed from pairwise t-tests to a one-way analysis of variance (ANOVA) followed by Dunnett's multiple-comparison post-hoc test, with each treatment condition compared against the common LPS-stimulated reference group. Under this standard framework the dose-dependent suppression of the PAR-2–ERK1/2–TNF-α/SOX4/ADAMTS-5 axis remains statistically significant, confirming that the findings are robust across analytical approaches and that the conclusions of the study are unchanged.

These changes are already visible as tracked changes in the revised manuscript. Specifically, the Statistical analysis section of the Methods now describes the one-way ANOVA with Dunnett's post-hoc test (replacing the previous t-test description and supported by an added methodological reference), and the figure legends for Figures 2, 3, 5, and 6 have been updated from "(Student's two-tailed unpaired t-test)" to "(one-way ANOVA with Dunnett's post-hoc test)." The legends to Figures 4, 7, and 8, which report significance thresholds without naming the test, are consistent with the revised analysis. Dunnett's test was selected because the design compares several treatment conditions against a single reference (the LPS-stimulated group), the structure for which Dunnett's procedure is specifically intended and which controls the family-wise error rate.

To avoid duplication in the response, and because the two comments converge on the same point, we respectfully direct the Reviewer to our detailed reply to **Reviewer #1, Comment 3**, where the rationale for the chosen post-hoc test and the full list of corresponding manuscript edits are set out in full.

Cross-reference

SEE ALSO

Reviewer #1, Comment 3 ("The statistical analysis is not appropriate, as multiple t-tests were used instead of one-way ANOVA with proper post hoc correction") and the corresponding Authors' Response, which details the ANOVA/Dunnett conversion, the choice of Dunnett over Tukey, and the associated changes to the Statistical analysis section, figure legends, and references.

Manuscript Changes

No further manuscript edits were required for this comment, as the requested analysis was already implemented in response to Reviewer #1, Comment 3. The relevant tracked changes (Statistical analysis section; figure legends for Figures 2, 3, 5, and 6; and the added methodological reference) are present in the revised manuscript.

Summary

The sensitivity analysis requested by the Reviewer has been carried out: the data were analysed by one-way ANOVA with Dunnett's post-hoc test, the reported effects remain statistically significant, and the manuscript text and figure legends already reflect this framework. We are grateful to the Reviewer for the suggestion, which reinforces the statistical robustness of the study.

Reviewer Comment 2

> *Cell Model Limitations: Explicitly acknowledge in the limitations that BM-MSC-derived chondrocytes, while phenotypically validated, may not fully capture the epigenetic "drift" characteristic of chronically diseased primary human OA tissue.*

Authors' Response

We thank the Reviewer for this perceptive comment, which we agree improves the calibration of the manuscript. The previous text contained only a brief clause on this point. In response, we have replaced it with a dedicated, fully referenced paragraph in the Limitations that explicitly acknowledges the cell-model constraint and, importantly, explains why it matters mechanistically rather than simply stating it.

The expanded paragraph makes three linked points. First, the chondrocytes used here were differentiated from bone marrow-derived mesenchymal stem cells and, although phenotypically validated, represent a developmentally naive population that does not carry the molecular history of chondrocytes resident in chronically diseased cartilage. Second, osteoarthritic chondrocytes acquire a distinctive and heritable epigenetic landscape, aberrant DNA methylation and histone modifications together with cellular senescence, that drives the sustained catabolic/anabolic imbalance of the disease, a phenomenon described in the literature as epigenetic drift; disease-associated marks such as hypomethylation of the MMP-13 and ADAMTS promoters are established in situ over years and are unlikely to be reproduced by a freshly differentiated cell. Third, monolayer culture itself induces dedifferentiation and DNA-methylation shifts that alter signalling-gene expression, including the ERK pathway central to our study, so that the transcriptional baseline of any in vitro chondrocyte model is sensitive to its derivation and culture history.

Crucially, we have framed the consequence in a measured way: the magnitude and even the hierarchy of EGCG's effects on the PAR-2–ERK1/2 axis could differ in primary OA chondrocytes carrying the full epigenetic burden of established disease, and the present findings should therefore be read as defining the mechanistic potential of EGCG in a well-controlled chondrocyte system rather than as a quantitative prediction of its activity in diseased tissue. We identify validation in primary OA patient-derived

chondrocytes, ideally in three-dimensional or co-culture systems that preserve the diseased phenotype, as an important next step.

Specific Revision Made (tracked changes in the revised manuscript)

In the Limitations, the single sentence on the BM-MSC model was deleted and replaced with the following referenced paragraph (shown as a tracked deletion and insertion):

INSERTED TEXT (LIMITATIONS)

"A further consideration concerns the cellular model itself. The chondrocytes used here were differentiated from bone marrow-derived mesenchymal stem cells (BM-MSCs) and, although phenotypically validated against established chondrogenic criteria, they represent a developmentally 'naive' chondrocyte population that may not fully reproduce the molecular state of chondrocytes resident in chronically diseased human OA cartilage. Osteoarthritic chondrocytes acquire a distinctive and heritable epigenetic landscape, encompassing aberrant DNA methylation and histone modifications that, together with cellular senescence, drive the sustained imbalance between catabolic and anabolic gene programmes that characterises the disease; this progressive, stably propagated remodelling is often termed epigenetic drift [Kim; Zhang]. Such disease-associated marks, for example hypomethylation of the promoters of matrix-degrading proteases including MMP-13 and the ADAMTS aggrecanases, are established in situ over years of mechanical and inflammatory stress and are unlikely to be recapitulated by a freshly differentiated BM-MSC-derived population. In addition, monolayer expansion of chondrocytes is itself accompanied by dedifferentiation and culture-induced shifts in DNA methylation that alter expression of phenotypic and signalling genes, including components of the ERK pathway interrogated here [Ma], underscoring that the transcriptional baseline of any in vitro chondrocyte model is sensitive to its derivation and culture history. Consequently, the magnitude and even the hierarchy of EGCG's effects on the PAR-2–ERK1/2 axis could differ in primary OA chondrocytes carrying the full epigenetic burden of established disease, and the present findings should be regarded as defining the mechanistic potential of EGCG in a well-controlled chondrocyte system rather than as a quantitative prediction of its activity in diseased tissue. We therefore regard validation in primary OA patient-derived chondrocytes, ideally in three-dimensional or co-culture systems that preserve the diseased phenotype and incorporate synoviocytes, as an important next step."

In-text citations correspond to the three references added below; the existing subsequent sentences on dosing, treatment window, and mitochondrial assessment were retained unchanged.

References Added

Three references were added (continuing the existing sequence).

1. Kim H, Kang D, Cho Y, Kim JH. Epigenetic Regulation of Chondrocyte Catabolism and Anabolism in Osteoarthritis. Mol Cells. 2015;38(8):677-84. doi: 10.14348/molcells.2015.0200. PMID: 26242192.
2. Zhang M, Egan B, Wang J. Epigenetic mechanisms underlying the aberrant catabolic and anabolic activities of osteoarthritic chondrocytes. Int J Biochem Cell Biol. 2015;67:101-9. doi: 10.1016/j.biocel.2015.04.019. PMID: 25975825.
3. Ma B, Leijten JC, Wu L, Kip M, van Blitterswijk CA, Post JN, et al. Gene expression profiling of dedifferentiated human articular chondrocytes in monolayer culture. Osteoarthritis Cartilage. 2013;21(4):599-603. doi: 10.1016/j.joca.2013.01.014. PMID: 23376013.

Summary

In response to this comment, we have replaced the brief cell-model clause with a dedicated, referenced Limitations paragraph that explicitly acknowledges the epigenetic drift of chronically diseased OA chondrocytes, explains why a BM-MSC-derived model may not capture it, and frames the implication proportionately while identifying primary OA-tissue validation as a clear next step. We are grateful to the Reviewer for prompting this improvement.

Reviewer Comment 3

> *Pathway Specificity: The siRNA data show that PAR-2 accounts for approximately 38% of the LPS-induced TNF-α output. Because substantial EGCG efficacy remains after silencing, the discussion should more clearly frame EGCG as a multifunctional agent where PAR-2 is a predominant node rather than an exclusive target.*

Authors' Response

We thank the Reviewer for this excellent point, which goes to the heart of how the siRNA result should be interpreted. We agree that the residual EGCG efficacy after PAR-2 silencing is best explained by framing EGCG as a multifunctional agent for which PAR-2 is the predominant, but not exclusive, node. *The manuscript already establishes this position in the siRNA paragraph, the Figure 8 legend, Table 2*, and the Conclusions. To make it more explicit and better evidenced, as the Reviewer requests, *we have added a dedicated paragraph in the Discussion subsection "PAR-2 is the principal upstream target of EGCG in osteoarthritic chondrocytes*," immediately following the existing PAR-2-independent paragraph and Table 2.

Following the Reviewer's framing, the new paragraph is organised top-down. It first treats the PAR-2-independent pathways for which EGCG has documented, OA-specific chondroprotective activity, and then turns to additional pathways that EGCG is known to modulate elsewhere and that are independently implicated in cartilage biology but have not yet been examined in OA chondrocytes, identifying them as biologically plausible further contributors.

1. OA-specific PAR-2-independent pathways (directly evidenced)

Three documented axes are highlighted. (i) The NF-κB–JNK/AP-1 inflammatory module, through which EGCG suppresses a broad inflammatory and catabolic protein network in primary human OA chondrocytes (existing references [39, 41]). (ii) The cytoprotective Nrf2–Keap1–ARE antioxidant response: in IL-1β-challenged human chondrocytes EGCG upregulates Nrf2, HO-1, and NQO1 and limits apoptosis and senescence, with effects abolished by the Nrf2 inhibitor ML385, confirming GPCR-independent pathway dependence (new reference). (iii) Restoration of autophagic flux: intra-articular EGCG attenuates cartilage degeneration in a post-traumatic OA model by enhancing chondrocyte autophagy via mTOR suppression and LC3/beclin-1 induction (new reference). The paragraph explicitly links these to the approximately 62% of LPS-induced TNF-α that persists after PAR-2 knockdown.

2. Plausible pathways not yet investigated in OA

The paragraph then identifies canonical Wnt/β-catenin signalling as a credible but as-yet-unexamined additional node: EGCG promotes GSK-3β- and PP2A-independent β-catenin phosphorylation and degradation (new reference), and dysregulated Wnt/β-catenin activity is independently established as a driver of chondrocyte hypertrophy, osteophyte formation, and catabolism in OA. This satisfies the request to extend beyond directly evidenced mechanisms to plausible ones, with a reference for each.

3. Honest calibration

To keep the framing rigorous, the paragraph closes by noting that several of these effects derive from single-pathway studies and that the polyphenolic, redox-active structure of EGCG can confound in vitro readouts, cross-referencing the existing discussion of this caveat [67]. The mechanisms are therefore presented as a plausible multi-pathway framework that situates the siRNA finding, not as a definitive accounting of every contributory axis.

Cross-reference

SEE ALSO

The new paragraph complements, and does not duplicate, the existing Table 2 ("PAR-2-Independent Signalling Pathways Modulated by EGCG"), which provides molecular-target detail for NF-κB/IKKβ, AP-1/JNK, Nrf2–ARE, PI3K–Akt–mTORC1, JAK1/2–STAT3, and AMPK. It is also consistent with the "predominant, though not exclusive" framing already present in the siRNA paragraph, the Figure 8 legend, and the Conclusions.

References Added

Three references were added (continuing the existing sequence).

1. Zhu W, Tang H, Cao L, Zhang J, Li J, Ma D, et al. Epigallocatechin-3-O-gallate ameliorates oxidative stress-induced chondrocyte dysfunction and exerts chondroprotective effects via the Keap1/Nrf2/ARE signaling pathway. Chem Biol Drug Des. 2022;100(1):108-20. doi: 10.1111/cbdd.14056. PMID: 35426252.
2. Huang HT, Cheng TL, Ho CJ, Huang HH, Lu CC, Chuang SC, et al. Intra-Articular Injection of (-)-Epigallocatechin 3-Gallate to Attenuate Articular Cartilage Degeneration by Enhancing Autophagy in a Post-Traumatic Osteoarthritis Rat Model. Antioxidants (Basel). 2021;10(1):8. doi: 10.3390/antiox10010008. PMID: 33374605.
3. Oh S, Gwak J, Park S, Yang CS. Green tea polyphenol EGCG suppresses Wnt/β-catenin signaling by promoting GSK-3β- and PP2A-independent β-catenin phosphorylation/degradation. BioFactors. 2014;40(6):586-95. doi: 10.1002/biof.1185. PMID: 25352148.

Summary

In response to this comment, we have added a dedicated, top-down Discussion paragraph that frames EGCG as a multifunctional agent with PAR-2 as the predominant node. It first sets out three OA-specific PAR-2-independent pathways with documented EGCG activity (NF-κB/JNK/AP-1; Nrf2–Keap1–ARE; autophagy/mTOR), explicitly accounting for the residual post-silencing efficacy, and then identifies Wnt/β-catenin as a plausible additional node not yet examined in OA, with a supporting reference for each aspect

and an honest caveat regarding single-pathway evidence and EGCG's assay-interference properties. We are grateful to the Reviewer, whose comment has sharpened the mechanistic interpretation of the study.

Reviewer Comment 4

Mitochondrial Claims: Since functional bioenergetic assays (e.g., Seahorse OCR or ATP quantification) were not performed, please refine the "mitoprotective" claims to specify that EGCG preserves structural and thermodynamic markers of mitochondrial integrity.

Authors' Response

We thank the Reviewer for this fair and precise comment. We agree that, because our mitochondrial assessment relied on Rhodamine 123 (reporting the membrane potential, $\Delta\Psi m$, a thermodynamic marker) and Janus Green B (reporting mitochondrial mass, a structural marker), and did not include functional bioenergetic assays, the manuscript should not assert effects on respiratory capacity or ATP output. We have therefore revised the relevant claims throughout the manuscript so that they are stated strictly in terms of the preservation of structural and thermodynamic markers of mitochondrial integrity, and we have explicitly disclaimed the functional inferences. We have also strengthened the Limitations to name the specific assays that would be required to establish bioenergetic function. The conclusions of the study are unaffected by these refinements. All changes are shown as tracked changes; no references were added or removed.

Specific Revisions (each shown as a tracked change in the manuscript)

1. **Title has been softened.** The mitochondrial claim in the title was narrowed to refer to markers.

INSERTED / REVISED TEXT
Was: ~~…and Preserves Mitochondrial Integrity in Osteoarthritic Chondrocytes~~
Now: *…and Preserves Markers of Mitochondrial Integrity in Osteoarthritic Chondrocytes*

2. **Abstract has been refined (and a typo corrected).** The closing claim of "mitoprotective effects" (printed as "Mito protective") was reworded.

INSERTED / REVISED TEXT
Was: ~~…exerts coordinated anti-inflammatory, anti-catabolic, and Mito protective effects.~~
Now: *…exerts coordinated anti-inflammatory and anti-catabolic effects, while preserving structural and thermodynamic markers of mitochondrial integrity.*

3. **Results (mitochondrial section) has been refined.** The interpretive sentence following the Janus Green / Rhodamine 123 data was narrowed and a no-functional-assay caveat added.

INSERTED / REVISED TEXT

Was: ~~…indicates that EGCG maintains structural and functional mitochondrial integrity under inflammatory stress…~~

Now: *…indicates that EGCG preserves the structural (mitochondrial mass) and thermodynamic (membrane potential) markers of mitochondrial integrity under inflammatory stress; because functional bioenergetic assays were not performed, we do not infer a direct effect on respiratory capacity or ATP output…*

4. **Discussion ("Preservation of mitochondrial integrity" subsection) — four coordinated refinements.** The bioenergetic and ATP inferences drawn from $\Delta\Psi m$ were softened and qualified.

INSERTED / REVISED TEXT

Was: ~~…represent the primary bioenergetic and structural parameters of mitochondrial integrity…~~

Now: *…represent the primary thermodynamic (membrane potential) and structural (mitochondrial mass) markers of mitochondrial integrity, rather than direct measures of bioenergetic function…*

INSERTED / REVISED TEXT

Was: ~~…characterising EGCG as a multimechanistic mitoprotective agent.~~

Now: *…characterising EGCG as a multimechanistic mitoprotective agent in other cell systems.*

INSERTED / REVISED TEXT

Was: ~~…the near-homeostatic $\Delta\Psi m$ recovery we document (Fig. 7) predicts restored oxidative-phosphorylation-coupled ATP synthesis…~~

Now: *…the near-homeostatic $\Delta\Psi m$ recovery we document (Fig. 7) would be expected to favour, but does not by itself demonstrate, oxidative-phosphorylation–coupled ATP synthesis…*

INSERTED / REVISED TEXT

Was: ~~The near-complete $\Delta\Psi m$ recovery at 10 µM EGCG (Fig. 7D–E) therefore implies simultaneous protection against chondrocyte apoptosis, ATP depletion, and oxidative stress…~~

Now: *The near-complete $\Delta\Psi m$ recovery at 10 µM EGCG (Fig. 7D–E) is therefore consistent with, though it does not directly establish, simultaneous protection against chondrocyte apoptosis, ATP depletion, and oxidative stress…*

5. **Limitations has been strengthened (new dedicated caveat).** The existing two-sentence note was replaced with an explicit statement of the marker-versus-function distinction and the assays required to establish bioenergetic function.

INSERTED / REVISED TEXT

A further limitation concerns the scope of the mitochondrial assessment. The two readouts employed here, Rhodamine 123 fluorescence and Janus Green B staining, report, respectively, the thermodynamic state of the inner membrane (mitochondrial membrane potential, $\Delta\Psi m$) and the structural parameter of mitochondrial mass. They are validated and informative markers of mitochondrial integrity, but they are not functional bioenergetic assays, and they do not directly measure respiratory chain activity or ATP production. Accordingly, our claims are confined to the preservation of these structural and thermodynamic markers, and we explicitly refrain from inferring that EGCG restores oxidative-phosphorylation–coupled ATP synthesis or respiratory capacity; such functional conclusions would require direct measurement. Definitive characterisation of the bioenergetic consequences of EGCG-mediated PAR-2 suppression will therefore require dedicated functional assays, including Seahorse

XF-based profiling of the oxygen consumption rate (OCR) and extracellular acidification rate (ECAR), luminometric ATP quantification, and mitochondria-targeted ROS measurement (MitoSOX Red); these are planned for the in vivo follow-up investigation.

Note: the descriptive pathway labels in Table 2 (for example, the "Nrf2–Keap1–ARE Antioxidant / Mitoprotective Axis") were retained, as they characterise mechanisms established in the cited literature rather than functional claims about the present data.

Summary

In response to this comment we have refined every claim about our own mitochondrial data, in the title, abstract, Results, and Discussion, so that EGCG is described as preserving the structural and thermodynamic markers of mitochondrial integrity rather than as exerting demonstrated bioenergetic or "mitoprotective" function, and we have explicitly disclaimed inferences about respiratory capacity and ATP output. We have additionally strengthened the Limitations to specify the functional assays (Seahorse OCR/ECAR, ATP luminometry, MitoSOX) required to establish those effects. We are grateful to the Reviewer, whose comment has improved the precision and rigour of the manuscript.

Reviewer #3 —Concerns

Reviewer Comment 1

In the methods, specifically "Ethics considerations for the present study," there is a lot of repetitive mentioning of not using human subjects and using cell lines. That paragraph could be written in a much more concise manner, as it is a bit confusing when written like that.

Authors' Response

We agree entirely. The section had been written as three paragraphs that each restated the same core facts, that the work was conducted in vitro with commercially available cell lines, involved no human subjects, and therefore did not require participant consent. We have condensed the section into a single, concise paragraph that retains every substantive point, the in vitro nature of the work, the absence of animals, human participants, patient-derived samples, or associated data, the resulting IRB-exempt status with a contact address, and the formal waiver of consent, while removing the repetition. The change is shown as tracked changes: the first paragraph is retained and gains a single closing sentence on the consent waiver, and the two subsequent redundant paragraphs are deleted.

Revision Made (tracked changes in the manuscript)

Text removed (two redundant paragraphs deleted):

DELETED TEXT

~~The study utilized only commercially available cell lines and did not include any involvement of human subjects. No minors were part of this research, and therefore parental or guardian consent was not required. Furthermore, the study did not involve any direct or indirect interaction with human participants, nor did it make use of medical records or archived human samples.~~

~~In summary, this work was limited to in vitro experiments using commercially sourced cell lines, with no involvement of human subjects. Accordingly, participant consent was not required, and the requirement for consent was formally waived by the ethics committee.~~

Sentence inserted at the end of the retained first paragraph (to preserve the consent-waiver fact):

INSERTED TEXT

As the work involved neither human participants nor identifiable human data, informed consent was not applicable and was formally waived by the MBRU IRB.

Resulting section (after acceptance of the tracked changes):

ETHICS CONSIDERATIONS FOR THE PRESENT STUDY — FINAL, CONDENSED

All experimental procedures in this study were performed exclusively in vitro and did not involve animal models, human participants, patient-derived samples, or associated data. Consequently, the research posed minimal risk and falls under the exempt review categories defined by the Institutional Review Board (IRB) at Mohammed Bin Rashid University (MBRU). Additional details or clarification can be

obtained from the MBRU IRB (irb@mbru.ac.ae). As the work involved neither human participants nor identifiable human data, informed consent was not applicable and was formally waived by the MBRU IRB.

Summary

The "Ethics considerations" section has been reduced from three repetitive paragraphs to a single concise paragraph that preserves all substantive information while eliminating the redundancy and the resulting confusion the Reviewer identified. We are grateful for the suggestion.

Reviewer Comment 2

In the methods, specifically "Cytotoxicity assessment, inflammatory chondrocyte model, and TNF-α quantification," there are two huge paragraphs that go deep into the rationale for using LPS to induce a pro-inflammatory response. I think those 2 big paragraphs should go into the discussion section. You may just use a sentence or so to summarize the rationale within the methods, but all that detailed mechanistic information is more appropriate for the discussion section.

Authors' Response

We agree with the Reviewer, and we thank them for this constructive structural suggestion. We have relocated the detailed mechanistic rationale for the LPS–TLR4 model out of the Methods and into the Discussion, leaving a single concise summary sentence (with the validated working concentration) in the Methods, as the Reviewer recommends. We have taken particular care to preserve the logical flow of the manuscript, and, because the Discussion already touched on the LPS model in the Limitations, we have trimmed the now-redundant sentences there so that the relocated material reads as a single coherent treatment rather than a repetition. No references were added or removed; the citations originally attached to the two paragraphs ([19–26]) move with the relocated text.

The change has three coordinated parts, all shown as tracked changes:

1. **Methods — two rationale paragraphs removed; one summary sentence inserted.** The two detailed paragraphs (the DAMP–TLR4 rationale and the PAR-2/F2RL1 rationale) were deleted and replaced with the following concise summary, which retains the validated working concentration and points the reader to the Discussion:

INSERTED TEXT (METHODS)

LPS (a high-affinity TLR4 ligand) was selected as the pro-inflammatory stimulus because it reproduces the TLR4-driven signalling that endogenous damage-associated molecular patterns activate in osteoarthritic cartilage, including the TLR4–NF-κB inductive pathway for PAR-2 expression; the mechanistic rationale for this choice is developed in the Discussion. The model was established at LPS 10 µg/mL, a concentration validated in prior work from our group to elicit robust pro-inflammatory signalling without cytotoxicity [17-19].

The two deleted paragraphs began, respectively, "The selection of LPS as the primary pro-inflammatory stimulus…" and "The specific suitability of LPS as the stimulus in the present investigation…"; their full text now appears, reworded for Discussion context, in the new subsection below.

2. **Discussion — new subsection added to house the relocated rationale.** A new subsection was inserted immediately before "Limitations," carrying the relocated mechanistic content (reworded from Methods phrasing into Discussion prose). It reads:

INSERTED TEXT (DISCUSSION — NEW SUBSECTION)

Rationale for the LPS–TLR4 model as a surrogate for DAMP-driven inflammation in osteoarthritis

A consideration that informs the interpretation of the present findings is the choice of LPS as the pro-inflammatory stimulus, which was predicated on the innate immunobiology of OA rather than on its generic utility as an in vitro inflammatory agent. In osteoarthritic cartilage, progressive enzymatic and mechanical disintegration of the extracellular matrix generates a diverse repertoire of damage-associated molecular patterns (DAMPs), including fibronectin fragments, HMGB1, tenascin-C, hyaluronan oligosaccharides, and S100A8/A9, that collectively drive innate immune activation and persistent low-grade synovial inflammation [67, 68]. Toll-like receptor 4 (TLR4) is the canonical pattern-recognition receptor through which the preponderance of these endogenous DAMPs transduce their signals in chondrocytes and synoviocytes, and TLR4 surface expression is elevated in osteoarthritic cartilage in proportion to histological disease severity [69]. As the high-affinity ligand of the TLR4–MD-2–CD14 co-receptor complex, LPS activates the identical intracellular cascades, MyD88 → IRAK1/4 → TRAF6 → TAK1 → IKKβ → NF-κB and TAK1 → MKK1/2 → ERK1/2, as those initiated by endogenous TLR4-activating DAMPs, because downstream signalling is determined by the intracellular adaptor architecture rather than by ligand identity [70]. LPS therefore serves as a mechanistically faithful surrogate for DAMP-mediated TLR4 activation in the OA joint, a rationale reinforced by evidence that both systemic and intra-articular LPS burden in humans are independently associated with radiographic knee OA severity and synovial inflammatory biomarkers [71]. This systemic source of TLR4 ligand is itself mechanistically grounded: metabolic endotoxaemia, the chronic low-grade elevation of circulating LPS arising from gut-barrier dysfunction in obesity, sustains systemic and synovial TLR4-driven inflammation and provides a biologically authentic basis for LPS as a model stimulus of the metabolically driven OA phenotype that constitutes a substantial share of the contemporary OA population [72].

The suitability of LPS is further reinforced by the mechanistic focus of this study on PAR-2. PAR-2 transcription in chondrocytes and innate immune cells is governed by NF-κB response elements in the F2RL1 promoter [73], and TLR4 → MyD88 → TRAF6 → IKKβ → NF-κB signalling constitutes the primary upstream inductive pathway for PAR-2 expression in this context. LPS stimulation thus reproduces the TLR4 → NF-κB → PAR-2 transcriptional axis operant in DAMP-stimulated osteoarthritic cartilage, as corroborated by the approximately four-fold increase in PAR-2 mRNA we observed in LPS-stimulated chondrocytes (Fig. 2C). The commonly used alternative stimulus IL-1β signals through a structurally distinct receptor (IL-1R1) and, being itself a downstream effector of PAR-2 signalling, would position a product of the receptor as the upstream stimulus, introducing a logical circularity that would complicate interpretation of PAR-2-centred experiments. By activating PAR-2 through its authentic transcriptional inductive pathway and positioning PAR-2 as a primary downstream effector rather than as a precondition of the model, LPS–TLR4 stimulation is therefore the specifically appropriate stimulus for the experimental question addressed here.

3. **Limitations — redundant LPS sentences trimmed for flow.** Because the relocated subsection now carries the full TLR4-fidelity rationale, the overlapping sentences previously in the Limitations were deleted and replaced with a brief bridge, so the two passages do not repeat. The limitation-specific content (what the LPS model does not capture) was retained.

DELETED TEXT (LIMITATIONS)

~~*Nevertheless, LPS activates TLR4, the canonical chondrocyte receptor for OA-relevant DAMPs including fibronectin fragments, HMGB1, and S100A8/A9, through molecularly identical intracellular cascades (MyD88–TRAF6–NF-κB–ERK1/2) as those triggered by endogenous DAMPs in the OA joint [19], and the LPS stimulus is specifically appropriate for the PAR-2-centred mechanistic question addressed herein, given that TLR4–NF-κB signalling constitutes the principal transcriptional inductive pathway for PAR-2 expression in chondrocytes;*~~

INSERTED BRIDGE (LIMITATIONS)

As detailed above, LPS nonetheless reproduces the TLR4-driven cascade through which endogenous DAMPs signal in the OA joint, and is the appropriate stimulus for the PAR-2-centred question addressed here;

The surrounding limitation-specific sentences, that LPS does not reproduce endogenous PAR-2 agonists or mechanical load, and the subsequent points on NLRP3, integrins, mechanosensors, and co-culture, were left unchanged.

On flow and references

The manuscript flow is preserved by three devices: the Methods summary sentence explicitly forwards the reader to the Discussion; the new Discussion subsection sits immediately before the Limitations, so the argument proceeds naturally from model rationale to model limitations; and the redundant Limitations sentences were trimmed so the relocated material is not duplicated. No new references were required, as all citations move with the relocated text. As in previous rounds, the relocated in-text citations are shown as static bracketed numbers and should be re-linked as live EndNote entries on the next field update.

Summary

In response to this comment, the two detailed LPS-rationale paragraphs have been moved from the Methods to a new, clearly headed Discussion subsection, with only a one-sentence summary retained in the Methods, and the overlapping LPS text in the Limitations has been trimmed to preserve a clean, non-repetitive flow. We are grateful to the Reviewer, whose suggestion has improved the structure and readability of the manuscript.

Reviewer Comment 3

There are several instances where words containing dashes are used but the dashes are not supposed to be there. Please proofread the manuscript carefully line-by-line. For example: an-ti-human (the dash after the "n" should not be there), and Di-agnostics (the dash after the "i" should not be there). There are many other examples in the manuscript that need to be looked at.

Authors' Response

We thank the Reviewer for this careful observation and apologise for these intrusive hyphens, which appear to have been introduced during earlier editing and formatting cycles. We have proofread the manuscript

line-by-line for words erroneously split by a hyphen. We identified and corrected every remaining instance; each correction is shown as a tracked change in the revised manuscript and itemised below.

We note that the two specific examples cited by the Reviewer ("an-ti-human" and "Di-agnostics") are no longer present in the current version, having been resolved during prior revision; we have nonetheless re-checked these and all related terms exhaustively to ensure none remain. To avoid over-correction, we were careful to preserve legitimately hyphenated compounds (for example, "anti-human," "G-protein-coupled," "LPS-stimulated," "RT-PCR") and correct suspended hyphens (for example, "GSK-3β- and PP2A-independent"), which are typographically correct and were left unchanged.

Corrections Made (each shown as a tracked change)

#	Location	Was (incorrect)	Now (corrected)
1	Figure 4 legend	~~expres-sion~~	**expression**
2	Abbreviations list (NF-κB definition)	~~nu-clear~~	**nuclear**
3	Figure 5 legend	~~un-treated~~	**untreated**
4	Methods (cell-culture conditions)	~~Un-treated~~	**Untreated**
5	Table 1 title	~~Real Time-PCR~~	**Real-Time PCR** *(mis-placed hyphen; should join "Real" and "Time")*

Each of the five corrections above is a tracked deletion of the hyphenated form and a tracked insertion of the corrected form, leaving the surrounding text untouched. In every case the corrected spelling matches the form used correctly elsewhere in the manuscript (for example, "expression" appears correctly 61 times, "nuclear" 6 times, and "untreated" 17 times), confirming that these were isolated typographical slips rather than a stylistic choice.

Method of Proofreading

To be thorough, we did not rely on visual scanning alone. We programmatically extracted the full running text of the manuscript and enumerated every hyphenated token, then compared each against its correct, unhyphenated form and against the manuscript's own predominant usage. This allowed us to distinguish genuine errors (a single word wrongly split, such as "nu-clear") from the many legitimate hyphenated compounds and suspended hyphens, and to confirm that the five corrections above represent the complete set of erroneous dashes remaining in the text.

Summary

We have proofread the manuscript line-by-line and corrected all remaining instances of words erroneously split by a hyphen (five in total: expres-sion, nu-clear, un-treated, Un-treated, and Real Time-PCR), each shown as a tracked change, while preserving all legitimate hyphenation. We are grateful to the Reviewer for prompting this careful pass, which has improved the typographical accuracy of the manuscript.

Reviewer Comment 4

In Figure 1, please include the phase contrast image of the BM-MSCs 28 days post-induction. You only included pre-induction. Readers should be able to look at both in the figure.

Authors' Response

We thank the Reviewer for this helpful suggestion. We have added the phase-contrast micrograph of the BM-MSCs at 28 days post-induction to Figure 1 as a new panel B, placed directly alongside the existing pre-induction phase-contrast image (panel A), so that readers can compare the two morphologies side by side, exactly as the Reviewer requests. The addition of this panel required the subsequent panels to be re-lettered, and we have propagated that re-lettering consistently through the figure legend and through every in-text citation of Figure 1. We have also added a short description of the post-induction morphology to the Results, with a brief comparison to a published account of BM-MSC chondrogenic morphology, supported by one new reference, so that the new panel is fully described without expanding any other section. The stain identities in the existing panels are unchanged. All edits are shown as tracked changes.

1. Panel re-lettering

The new post-induction phase-contrast image is inserted as panel B; all subsequent panels shift by one letter:

Panel content	Old label	New label
Phase-contrast, pre-induction	A	A (unchanged)
Phase-contrast, 28 d post-induction	—	**B (new panel)**
Alcian Blue	B	C
Toluidine Blue	C	D
Collagen type II	D	E
MTT viability — LPS	E	F
MTT viability — EGCG	F	G

2. Figure legend (tracked change)

The legend was updated to describe the new panel B and to re-letter panels C–G; the scale-bar note was updated accordingly. The new panel-B description reads:

INSERTED TEXT (LEGEND — NEW PANEL B)

(B) Phase-contrast micrograph of the same population after 28-day chondrogenic induction, showing the transition to a compact, rounded, polygonal chondrocyte-like morphology.

The scale-bar note was updated from "Scale bars: 100 µm (A–D)" to "Scale bars: 100 µm (A–E)" to reflect the additional histology panel, and panels previously lettered B, C, D, E, F were re-lettered to C, D, E, F, G respectively. The stain descriptions themselves (Alcian Blue, Toluidine Blue, collagen type II) are retained verbatim.

3. Results (tracked change)

A sentence was added to the "Validation of chondrogenic differentiation" paragraph describing the post-induction morphology now shown in panel B, with a brief comparison to a published description of the morphological progression of BM-MSC chondrogenesis. The in-text Figure 1 references in this paragraph and in the "Cytotoxicity profiling" paragraph were re-lettered to match the new panel order. The inserted sentence reads:

> **INSERTED TEXT (RESULTS)**
>
> *Phase-contrast microscopy at this 28-day endpoint confirmed this transition at the morphological level: the cells had lost the elongated, spindle-shaped fibroblastic appearance of the undifferentiated population (Fig. 1A) and adopted a more compact, rounded, polygonal chondrocyte-like morphology with denser intercellular contacts (Fig. 1B), consistent with the progressive acquisition of a chondroblastic phenotype described during BM-MSC chondrogenesis [78].*

In-text re-lettering in the Results: Alcian Blue (Fig. 1B → 1C), Toluidine Blue (Fig. 1C → 1D), collagen type II (Fig. 1D → 1E), LPS viability (Fig. 1E → 1F), and EGCG viability (Fig. 1F → 1G).

4. Reference added

One reference was added to support the morphological comparison (numbered as the next in sequence; final numbering will be assigned on EndNote field update):

> **NEW REFERENCE [78]**
>
> *Giuliani N, Lisignoli G, Magnani M, Racano C, Bolzoni M, Dalla Palma B, et al. New Insights into Osteogenic and Chondrogenic Differentiation of Human Bone Marrow Mesenchymal Stem Cells and Their Potential Clinical Applications for Bone Regeneration in Pediatric Orthopaedics. Stem Cells Int. 2013;2013:312501. doi: 10.1155/2013/312501. PMID: 23766767; PMCID: PMC3676919.*

Summary

Figure 1 now presents both the pre-induction (panel A) and the 28-day post-induction (new panel B) phase-contrast micrographs side by side, allowing direct visual comparison as the Reviewer requested. The remaining panels have been re-lettered consistently across the figure, the legend, and all in-text references; a description of the post-induction morphology with a brief published comparison has been added to the Results; and one supporting reference has been added. The stain identities are unchanged. We are grateful to the Reviewer for the suggestion, which improves the completeness of the figure.

Reviewer Comment 5

> *In the figure legend for Figure 1, you mention the following, but this is more appropriate to be included in the corresponding methods section: "(Note: Chondrogenic differentiation and histochemical/immunocytochemical staining were performed as validation of our previously established protocol, as described in Patnaik et al [17, 18, 25])."*

Authors' Response

We agree with the Reviewer. The note in question concerns the provenance of the differentiation and staining procedures and is methodological in nature, so it belongs in the Methods rather than in the figure legend. We have therefore removed the note, together with its citations, from the Figure 1 legend, and we have added a concise equivalent statement to the corresponding Methods section. We confirm that the Methods already describe these procedures in full: the "Cell culture and chondrogenic differentiation" subsection details the differentiation protocol, and the "Histological assessment" subsection details the collagen type II immunostaining and the Alcian Blue and Toluidine Blue staining. The relocated sentence makes the provenance of these established protocols explicit and carries the three references ([17, 18, 19]) that previously appeared in the legend. No references were added or removed; they were moved. Both edits are shown as tracked changes.

1. Removed from the Figure 1 legend

The following parenthetical note was deleted from the end of the Figure 1 legend (the citations [17, 18, 25] were removed with it):

DELETED TEXT (FIGURE 1 LEGEND)

~~*(Note: Chondrogenic differentiation and histochemical/immunocytochemical staining were performed as validation of our previously established protocol, as described in Patnaik et al [17, 18, 25]).*~~

2. Added to the Methods

A concise provenance sentence was inserted at the end of the "Cell culture and chondrogenic differentiation" subsection, immediately preceding the "Histological assessment" subsection that describes the staining procedures. It reads:

INSERTED TEXT (METHODS — CELL CULTURE AND CHONDROGENIC DIFFERENTIATION)

The chondrogenic differentiation and the histochemical and immunocytochemical staining procedures used here to validate the chondrocytic phenotype follow our previously established and published protocols [17, 18, 19].

For completeness, we note that the Methods already contain the full procedural detail that the relocated provenance statement refers to:

• "Cell culture and chondrogenic differentiation" — BM-MSC culture, micromass pelleting, chondrogenic induction medium, and the 21-day plus 7-day maturation schedule.

• "Histological assessment" — formalin fixation, paraffin embedding, sectioning, collagen type II immunostaining, Alcian Blue and Toluidine Blue staining, and imaging.

Summary

The methodological note has been moved out of the Figure 1 legend and into the Methods, where the differentiation and staining procedures are already described in full; the citations [17, 18, 25] have moved with it, and the legend now ends at the scale-bar statement. We are grateful to the Reviewer for the suggestion, which improves the placement of methodological information in the manuscript.

Reviewer Comment 6

Please include why you chose those concentrations of EGCG and LPS, and whether the concentrations are physiological or supraphysiological (i.e., realistic if one drinks a cup of green tea; how much EGCG is actually absorbed? Is this even known?? and the same for LPS…what is the minimum physiological concentration that induces an inflammatory response?).

Authors' Response

We thank the Reviewer for this important and nuanced comment, which we have addressed in detail. We have made the rationale for both concentrations explicit, stated plainly whether each is physiological or supraphysiological, and engaged directly with the specific questions raised, how much EGCG is actually absorbed and whether this is known, and what the minimum physiologically relevant LPS concentration is. For LPS we have additionally documented that our concentration is the established norm in the field, citing independent studies that used closely comparable LPS concentrations to generate inflammatory chondrocyte models, and we have set out the methodological principle that in vitro inflammatory priming inherently requires stimulus concentrations well above those present in tissue or synovial fluid. A concise statement of the LPS concentration rationale was added to the Methods, and a dedicated new Discussion subsection, "Selection of the LPS and EGCG concentrations: physiological versus supraphysiological context," was added immediately after the existing pharmacokinetic discussion. Five references were added to support the new content (with several existing references re-cited). All edits are shown as tracked changes.

1. **Methods — explicit LPS concentration rationale.** A sentence was added immediately after the LPS model is introduced:

INSERTED TEXT (METHODS)

This concentration lies within the 1–10 µg/mL range conventionally employed to elicit reproducible TLR4-dependent inflammatory signalling in chondrocytes in vitro, and was selected to ensure a robust, reproducible stimulus; its relationship to the lower lipopolysaccharide concentrations encountered physiologically, and the translational rationale for this choice, are considered in detail in the Discussion.

2. **Discussion — new subsection on concentration selection and physiological context.** Two new paragraphs were added under a dedicated subheading, the first on LPS, the second on EGCG.

LPS — (a) precedent: others used comparable concentrations.

We now document that the 10 µg/mL concentration is the established norm rather than an idiosyncratic choice, citing independent chondrocyte studies, including one that used precisely 10 µg/mL LPS:

INSERTED TEXT (DISCUSSION — LPS PRECEDENT, EXCERPT)

…Importantly, this is not an idiosyncratic choice: several independent groups have used closely comparable LPS concentrations to generate inflammatory chondrocyte models of OA. Xu et al. employed precisely 10 µg/mL LPS to induce inflammatory and catabolic mediator expression (IL-6, IL-8, TNF-α, and the MMP panel) in chondrocytes as the in vitro arm of a combined in vitro and in vivo study of osteoarthritis [63], and Ma et al. similarly used LPS to stimulate chondrocytes as a model of the inflammatory and apoptotic processes that underlie OA [64]; comparable LPS-driven chondrocyte

models recur throughout the OA literature. The concentration we adopted is therefore consistent with established practice in the field rather than an outlier…

LPS — (b) the principle: in vitro models require higher concentrations than tissue.

We set out explicitly why in vitro work uses concentrations above tissue/synovial levels, and support it with the chondrocyte-specific precedent that the standard in vitro IL-1β priming concentration is three to four orders of magnitude above synovial-fluid levels:

INSERTED TEXT (DISCUSSION — IN VITRO PRINCIPLE, EXCERPT)

…This apparent disparity reflects a general and well-recognised feature of in vitro inflammatory modelling rather than a peculiarity of the present work: monolayer and short-term cultures lack the amplifying cellular crosstalk, paracrine feedback, and prolonged exposure of the intact joint, and consequently require stimulus concentrations substantially higher than those measured in tissue or synovial fluid in order to evoke a synchronous, robust, and quantifiable response within a tractable experimental window. This principle is well documented for chondrocyte models specifically, where the interleukin-1β concentrations conventionally used for in vitro inflammatory priming (1–10 ng/mL) lie three to four orders of magnitude above the levels actually present in osteoarthritic synovial fluid (typically 1–20 pg/mL), yet are required to produce a reproducible OA-like transcriptional phenotype [67]…

LPS — (c) physiological status, the "minimum concentration" question, and the obesity–microbiome theme.

Retained from the previous version: we state plainly that 10 μg/mL is supraphysiological, quantify the in vivo burden, answer the minimum-concentration question, and connect to the obesity/gut-microbiome axis with EULAR weight-management framing:

INSERTED TEXT (DISCUSSION — LPS PHYSIOLOGY + OBESITY, EXCERPT)

…Circulating LPS in healthy individuals is in the low picogram-per-millilitre range, and even the chronic, low-grade elevation that characterises metabolic endotoxaemia represents only a two- to three-fold increase over baseline, remaining 10- to 50-fold below the concentrations seen in overt sepsis [65, 66]; indeed, the minimum concentration capable of initiating a measurable TLR4-dependent response in susceptible cells can be as low as the picogram-per-millilitre range under sustained exposure [66]…

…in obesity, gut-barrier dysfunction permits translocation of microbiome-derived LPS into the systemic circulation, sustaining a chronic metabolic endotoxaemia that drives TLR4-mediated low-grade inflammation in target tissues including the joint… Obesity is itself among the strongest modifiable risk factors for OA, and current EULAR recommendations for the non-pharmacological core management of hip and knee OA place the maintenance of a healthy weight and weight loss among their central pillars [69]…

EGCG — dietary realism via Table 3, absorption, and the systemic-versus-synovial caveat.

Retained from the previous version: Table 3 shows the dietary margin; the caveat is stated plainly that luminal concentration is not systemic or intra-articular exposure, peak plasma is submicromolar (~0.17 μM), and EGCG in synovial fluid has never been quantified in humans:

INSERTED TEXT (DISCUSSION — EGCG, EXCERPT)

…As catalogued there across 36 commercially consumed green teas, a single serving delivers EGCG to the intestinal lumen at concentrations (several hundred to several thousand micromolar) that exceed the 5–10 µM used here by one-and-a-half to two-and-a-half orders of magnitude… only a small fraction of ingested EGCG is absorbed intact, peak plasma concentrations after a single serving are submicromolar (approximately 0.17 µM [58]), and, to our knowledge, no human pharmacokinetic study has directly quantified EGCG in synovial fluid following oral consumption [58]…

3. **References added.** Five references were added (*Please refer to the revised manuscript for exact numerical designation of references*):

PRECEDENT, LPS 10 µG/ML IN CHONDROCYTES

Xu Y, Xue S, Zhang T, et al. Toddalolactone protects against osteoarthritis by ameliorating chondrocyte inflammation and suppressing osteoclastogenesis. Chin Med. 2022;17(1):18. doi: 10.1186/s13020-022-00576-w. PMID: 35123541; PMCID: PMC8817519.

PRECEDENT, LPS-STIMULATED CHONDROCYTE OA MODEL

Ma Z, Wang D, Weng J, Zhang S, Zhang Y. BNIP3 decreases the LPS-induced inflammation and apoptosis of chondrocytes by promoting the development of autophagy. J Orthop Surg Res. 2020;15(1):284. doi: 10.1186/s13018-020-01791-7. PMID: 32723351; PMCID: PMC7385973.

IN VITRO PRIMING EXCEEDS SYNOVIAL-FLUID LEVELS

Ragni E, De Luca P, Valli F, Zagra L, de Girolamo L. Inflammatory Treatment Used to Mimic Osteoarthritis and Patients' Synovial Fluid Have Divergent Molecular Impact on Chondrocytes In Vitro. Int J Mol Sci. 2023;24(3):2625. doi: 10.3390/ijms24032625. PubMed PMID: 36768948; PubMed Central PMCID: PMCPMC9916904.

EULAR (WEIGHT MANAGEMENT)

Moseng T, Vliet Vlieland TPM, Battista S, et al. EULAR recommendations for the non-pharmacological core management of hip and knee osteoarthritis: 2023 update. Ann Rheum Dis. 2024;83(6):730-740. doi: 10.1136/ard-2023-225041. PMID: 38212040; PMCID: PMC11103326.

METABOLIC ENDOTOXAEMIA (QUANTITATIVE)

Boutagy NE, McMillan RP, Frisard MI, Hulver MW. Metabolic endotoxemia with obesity: Is it real and is it relevant? Biochimie. 2016;124:11-20. doi: 10.1016/j.biochi.2015.06.020. PMID: 26133659; PMCID: PMC4695328.

Why this strengthens the response

The revised LPS rationale now rests on three mutually reinforcing pillars: (i) precedent, our concentration matches that used by independent groups, including one that used exactly 10 µg/mL [81, 82]; (ii) methodological principle, in vitro inflammatory models inherently require stimulus concentrations above tissue levels, a point we evidence with the chondrocyte-specific IL-1β example in which the standard priming concentration is three to four orders of magnitude above synovial-fluid levels [83]; and (iii) translational authenticity, the obesity–gut-microbiome endotoxaemia axis grounds LPS as a biologically relevant stimulus for the metabolically driven OA phenotype [72, 73, 79, 80]. Together these establish the concentration as standard, justified, and mechanistically meaningful, rather than merely acknowledged as supraphysiological.

A note on honesty of framing

We continue to state explicitly that the in vitro concentrations of both LPS and EGCG are not directly attainable in vivo through ordinary green tea consumption, and we frame the work as a mechanistic proof of concept. EULAR is cited only for what it actually says that weight management and lifestyle modification are core to OA management; we do not represent it as endorsing green tea or any nutraceutical. The precedent and methodological references are used to show that our concentration is standard and defensible, not to imply that 10 µg/mL is physiological.

Summary

The LPS concentration rationale is now substantially stronger: it documents that comparable concentrations are standard in the chondrocyte OA literature (including an exact 10 µg/mL precedent), establishes the methodological necessity of supraphysiological stimulus concentrations in vitro with a directly relevant chondrocyte example, retains the explicit supraphysiological acknowledgment and the minimum-inflammatory-concentration answer, and preserves the obesity–microbiome translational theme and EULAR framing. The EGCG rationale (Table 3 dietary comparison, absorption facts, and the systemic-versus-synovial caveat) is retained in full. Five references were added. We are grateful to the Reviewer, whose comment has materially strengthened the manuscript.

Comments 7, 8 and 9 all concern Figure 2 and are addressed together below. We have revised the figure comprehensively in response, and we are grateful for the close reading, which has improved its clarity and presentation. One element of Comment 7, the proposal to place the mRNA panel before the protein panels, we have, with respect, not adopted; we set out a detailed, referenced rationale for retaining the present order in Section C.

Reviewer Comments 7–9

COMMENT 7

In Figure 2, the fonts are not consistent, as there is both Arial and Times New Roman font. Please just keep one font. The font size is also not consistent on axis labels. The x-axis labels are too verbose. Also, usually mRNA expression is shown first, followed by protein expression, going in chronological order of synthesis. In other words, panel C should actually be A. This should also be fixed in the methods section, as RNA synthesis should be before protein synthesis. Also, Why is there a black bar on the right side labeled PAR-2? It is implied from the x-axis label that this is PAR2 expression. Again, please fix the x-axis labels.

COMMENT 8

Figure 2 would benefit from a different way of labeling the samples. I would just use: "Ctrl" for "Chondrocytes"; "LPS" instead of "Chondrocytes + LPS"; "LPS + EGCG-5 µM" instead of "Chondrocytes + LPS + EGCG-5uM"; "LPS + EGCG-10 µM" instead of "Chondrocytes + LPS + EGCG-10uM".

COMMENT 9

When comparing the original blot image for PAR-2 in Figure 2 to the cropped figure, the image is upside down: the non-specific bands in the original are below the PAR-2 bands, but in the cropped manuscript image they are above. Please keep the image in the correct orientation. Also, there is no need to crop GAPDH so close to the bands that the border is almost touching the bands. Please fix this too.

Authors' Response

We have revised Figure 2 to address each presentational point raised in Comments 7, 8 and 9. The remaining element, the suggested reordering of the panels so that mRNA precedes protein, is addressed separately and in detail in Section C, where we explain, with supporting literature, why the protein-first arrangement is the more appropriate one for this study and why it is internally consistent with every other figure in the manuscript and with the Methods.

A. Presentational corrections (Comments 7 and 8)

All the following have been implemented in the revised Figure 2:

Reviewer's point	Action taken
Mixed Arial / Times New Roman fonts (Comment 7)	All text in Figure 2 is now set in a single typeface (Arial) throughout every panel.
Inconsistent font size on axis labels (Comment 7)	Axis labels, tick labels and annotations are now a uniform size across panels B and C.
X-axis labels too verbose (Comments 7 and 8)	Relabelled to Ctrl, LPS, LPS + EGCG-5 µM, LPS + EGCG-10 µM.
Sample labels too long (Comment 8)	Adopted verbatim: Ctrl, LPS, LPS + EGCG-5 µM, LPS + EGCG-10 µM, in both the blot (panel A) and the graphs (panels B, C).
Black bar on the right labelled "PAR-2" (Comment 7)	Removed. The y-axis of the former mRNA panel now reads "Fold change in PAR-2 expression", so the identity of the analyte is given by the axis itself and the redundant legend key is no longer needed.

B. Blot orientation and GAPDH cropping (Comment 9)

Both points are well taken and have been corrected:

Reviewer's point	Action taken
PAR-2 blot inverted relative to the source image	The PAR-2 panel has been re-oriented to match the original acquisition, so that the non-specific bands again lie below the specific PAR-2 bands, exactly as in the source blot.

Reviewer's point	Action taken
GAPDH cropped too tightly to the bands	The GAPDH panel has been re-cropped with adequate margin above and below the bands, so the crop border no longer abuts the signal.

Full-length, unprocessed blot images continue to be provided in the Supplementary Data, allowing direct verification of orientation and cropping against the source.

C. Panel order: why protein precedes mRNA (response to Comment 7)

The Reviewer suggests that, because transcription precedes translation, the mRNA panel (former panel C) should appear first, and that the Methods should likewise be reordered. We fully take the Reviewer's point about the central-dogma sequence. After careful consideration, however, we respectfully propose to retain the present order, in which the protein readout precedes the transcript readout, for four mutually reinforcing reasons. We hope the Reviewer will agree that, taken together, these make the protein-first arrangement the more logical and internally consistent choice for this particular manuscript.

1. **Results figures are organised by the logic of inference, not by the biosynthetic order of the molecules assayed.** The organising principle of a results figure is conventionally the line of scientific reasoning: the primary functional readout is presented first, and the mechanistic explanation second. The central question of this study is whether EGCG modulates the functional PAR-2 receptor, which is a protein. The immunoblot (panels A, B) answers that primary question directly; the transcript measurement (panel C) then explains how the protein-level change comes about, by showing that it is transcriptionally driven. Presenting protein first and mRNA second therefore mirrors the inferential flow of the study (functional outcome → underlying mechanism), which is the more informative sequence for the reader.

2. **For a cell-surface signalling receptor, the protein is the functionally decisive species.** PAR-2 exerts its biological effect as a membrane-bound receptor that initiates the downstream ERK1/2 → TNF-α / SOX4 / ADAMTS-5 cascade interrogated in the rest of the manuscript. The transcript is an intermediate; it is the receptor protein, not its mRNA, that determines signalling capacity. It is therefore appropriate that the functionally decisive readout is presented as the principal result, with the transcript shown subsequently as supporting mechanism.

3. **mRNA abundance is an unreliable surrogate for protein abundance, so the protein result must stand in its own right.** Genome-wide analyses have repeatedly shown that steady-state transcript levels explain only a minority of the variance in protein abundance, on the order of 40%, with the remainder governed by post-transcriptional, translational and protein-degradation control [A, B]. Because the transcript cannot be assumed to predict the protein, the protein must be measured and presented as a primary endpoint in its own right, rather than being treated as a downstream corollary of the mRNA. Presenting the directly measured protein first, and then demonstrating that, in this instance, the change is also reflected at the transcript level (i.e. that the regulation is transcriptional), is the logically sound order; the noteworthy finding is precisely the concordance between the two, which cannot be presupposed.

4. **Protein-first ordering is uniform across the manuscript and is already mirrored in the Methods.** Every multi-panel functional figure in the manuscript presents the protein or functional readout first and the transcript readout second: Figure 2 (PAR-2 protein then mRNA), Figure 3 (ERK1/2 immunoblot then ERK-pathway transcripts), Figure 4 (cytokine ELISA then mRNA), Figure 5 (SOX4 and ADAMTS-5

protein then mRNA) and Figure 8 (PAR-2 immunoblot and TNF-α ELISA then mRNA). Reordering Figure 2 alone would make it the single exception and would break the visual and logical parallelism that lets the reader read all of the figures in the same way. Importantly, the Methods are already arranged in the same protein-first sequence ("Western blotting" precedes "RNA extraction, cDNA synthesis, and quantitative PCR"), so the figure, the Results narrative and the Methods are already mutually consistent. Reordering only the figure would, in fact, introduce the very figure–methods mismatch the Reviewer asks us to avoid; retaining the present order preserves consistency throughout.

For these reasons we have kept Figure 2 (and the corresponding Methods order) as protein-first, while adopting every other change the Reviewer requested. We hope this rationale is persuasive; if the Reviewer nonetheless feels strongly that the biosynthetic order should govern, we would of course be willing to reorder all functional figures and the Methods uniformly so that consistency across the manuscript is maintained.

REFERENCES CITED IN SUPPORT OF POINT C.3 (REBUTTAL ONLY)

[A] Vogel C, Marcotte EM. Insights into the regulation of protein abundance from proteomic and transcriptomic analyses. Nat Rev Genet. 2012;13(4):227-232. doi: 10.1038/nrg3185. PMID: 22411467.

[B] Liu Y, Beyer A, Aebersold R. On the Dependency of Cellular Protein Levels on mRNA Abundance. Cell. 2016;165(3):535-550. doi: 10.1016/j.cell.2016.03.014. PMID: 27104977.

These two references are cited here to substantiate the rationale; they are not currently inserted into the manuscript, since the panel order is unchanged and no new manuscript text is required. Should the editor or Reviewer prefer, we would be glad to add a single sentence to the Discussion (where protein–transcript concordance is already noted) citing these works, to make the rationale explicit in the manuscript itself.

Summary

Figure 2 has been fully revised: a single typeface (Arial) throughout; uniform axis-label sizing; concise sample labels (Ctrl, LPS, LPS + EGCG-5 µM, LPS + EGCG-10 µM); removal of the redundant "PAR-2" legend bar in favour of an explicit y-axis title; correction of the PAR-2 blot orientation to match the source image; and re-cropping of GAPDH with appropriate margins. We have retained the protein-first panel order, and have given a detailed, referenced rationale for doing so, namely that it follows the study's line of inference, reflects the functionally decisive role of the receptor protein, is warranted by the well-documented unreliability of mRNA as a surrogate for protein, and preserves consistency with every other figure and with the Methods. We are grateful to the Reviewer; the figure is substantially clearer as a result of these changes.

Reviewer Comments 10

In the Figure 2 legend, you mention "2−ΔΔCT". This is incorrect. It should be $2^{-\Delta\Delta CT}$, meaning the "−ΔΔCT" part should be a superscript (exponent). Please also fix this in the methods section.

Authors' Response

We thank the Reviewer for this careful and correct observation. We agree entirely. In the comparative C_t ($\Delta\Delta C_t$) method of relative quantification, the term "−ΔΔCT" is the exponent of the base 2 and must therefore be set as a superscript rather than on the baseline.

Accordingly, the notation has been corrected so that "−ΔΔCT" is displayed as a superscript, giving the conventional form $2^{-\Delta\Delta CT}$. The base "2" remains on the baseline and the exponent is now raised in both locations where the expression appears in the running text: the **Methods** section (subsection "RNA extraction, cDNA synthesis, and quantitative PCR") and the **Figure 2 legend** (panel C). The supporting citation to Livak and Schmittgen [25], which is the source of the method, is retained unchanged.

For completeness, we note that the expression also appears within the title of reference [25] (Livak KJ, Schmittgen TD, Methods, 2001), where it is reproduced as "2(-Delta Delta C(T)) Method" exactly as published. As bibliographic entries must faithfully match the original source, this occurrence has been left unaltered.

Manuscript Changes

The corrected notation is reflected in the revised manuscript at both occurrences in the body text, as summarised below (the changes are visible in the tracked-changes version):

Location	Before	After
**Methods — "RNA extraction, cDNA synthesis, and quantitative PCR"**	**2−ΔΔCT**	$2^{-\Delta\Delta CT}$
**Figure 2 legend — panel (C)**	**2−ΔΔCT**	$2^{-\Delta\Delta CT}$

"Before" shows the exponent on the baseline; "After" shows it correctly raised as a superscript.

Summary

The relative-quantification notation now correctly renders the exponent "−ΔΔCT" as a superscript — $2^{-\Delta\Delta CT}$ — in both the Methods section and the Figure 2 legend, in line with standard usage and the Reviewer's recommendation. We are grateful to the Reviewer for identifying this typographical inconsistency, the correction of which improves the precision of the manuscript.

Reviewer Comment 11

> *The same sample and x-axis labeling that I pointed out for Figure 2 also exist in Figure 3, 4, 5, 6, 7, and 8. It is not appropriate to label the samples 1, 2, 3, 4, etc. for a publication in a peer-reviewed journal. Please find a way to label the samples as explained in a previous comment.*

Authors' Response

We thank the Reviewer for this observation, and we agree. As recommended, we have applied the sample-labelling convention adopted for Figure 2 in response to the Reviewer's earlier comment (Comment 8) consistently across Figures 3, 4, 5, 6, 7 and 8. In every panel, the previous index labels — the numerals 1, 2, 3, 4… in Figures 3, 4, 5 and 8, and the panel letters A–D and F–I in Figures 6 and 7 — together with their separate numbered (or lettered) legend keys, have been removed.

The treatment conditions are now written out in full and placed directly on the x-axes, and on the corresponding blot lanes and flow-cytometry plots, using the concise descriptive labels Ctrl, LPS, LPS + EGCG-5 μM and LPS + EGCG-10 μM. Where a figure contains additional groups — the extended EGCG dose series in Figure 4 and the PAR-2 siRNA conditions in Figure 8 — these are likewise labelled explicitly

(for example, siRNA, siRNA + LPS, siRNA + LPS + EGCG-5 µM and siRNA + LPS + EGCG-10 µM in Figure 8), so that every bar, lane and plot can be read without reference to an external key. The vague axis title "Chondrocytes under different conditions" used in the former Figure 8 has accordingly been removed. These changes make the figures self-explanatory and mutually consistent, and consistent with the revised Figure 2. The fully revised figures are provided in the updated figure file. As the modification is confined to the figures, no change to the manuscript text was required; the corresponding figure legends already describe the same treatment conditions in words.

Summary of the relabelling applied to each figure:

Figure	Previous labelling	Revised labelling
**Figure 3 (ERK1/2 signalling)**	Blot lanes and bar-chart x-axes numbered 1–4, defined in a separate numbered legend box.	Ctrl, LPS, LPS + EGCG-5 µM, LPS + EGCG-10 µM — placed directly on the lanes and all axes.
**Figure 4 (cytokine secretion)**	Bar-chart x-axis numbered 1–4 with a numbered legend (former panel A was a tabulated dose series).	Ctrl, LPS, LPS + EGCG-5 µM, LPS + EGCG-10 µM; the dose-response panel labelled Ctrl, LPS, LPS + EGCG-1/5/10/20 µM.
**Figure 5 (SOX4 / ADAMTS-5)**	Blot lanes and bar-chart x-axes numbered 1–4, with two numbered legend boxes.	Ctrl, LPS, LPS + EGCG-5 µM, LPS + EGCG-10 µM — directly on lanes and axes.
**Figure 6 (RANK / RANKL)**	Flow plots and bar-chart x-axes lettered A–D and F–I, with a legend box mapping letters to conditions.	Flow plots titled by condition; bar charts labelled Ctrl, LPS, LPS + EGCG-5 µM, LPS + EGCG-10 µM.
**Figure 7 (mitochondrial staining)**	Bar-chart x-axes lettered A–D and F–I, with a legend box.	Direct descriptive labels (Ctrl, LPS, LPS + EGCG-5 µM, LPS + EGCG-10 µM) on both staining panels.
**Figure 8 (PAR-2 siRNA)**	Blot lanes and line-plot points numbered 1–8; numbered legend; x-axis titled "Chondrocytes under different conditions".	All eight groups labelled directly: Ctrl, LPS, LPS + EGCG-5/10 µM, siRNA, siRNA + LPS, siRNA + LPS + EGCG-5/10 µM.

In every panel the descriptive labels are now shown directly on the axis, lane or plot, and the separate numbered or lettered legend keys have been removed.

Summary

Across Figures 3–8, the samples are no longer identified by numeric or alphabetic index keys with a separate legend. Each treatment group is now labelled directly and in full on the relevant axis, lane or plot, following the scheme established for Figure 2 in Comment 8 (Ctrl, LPS, LPS + EGCG-5 µM, LPS + EGCG-10 µM, with the additional dose and siRNA conditions written out where applicable). We are grateful to the Reviewer for prompting this improvement, which makes the figures clearer and more appropriate for a peer-reviewed publication.

Reviewer Comment 12

In Figure 3, the fonts are not all the same. The whole figure is actually very confusing and cannot stand on its own as a proper figure. This figure needs significant changes. Since you altered the original blot to exclude unwanted bands, you should add a dashed line between the two bands that were "attached"

to each other. In addition, I really don't understand why the figure has lower quality bands in that they are stretched and very thin compared to the original blot. Again, please label the samples correctly as mentioned in a previous comment. And once more, please have mRNA expression panels first, followed by protein expression panels. The x-axis label for Figure 3-D is not the correct label.

Authors' Response

We thank the Reviewer for this detailed appraisal of Figure 3, which we have substantially revised in response. In brief, the figure has been re-typeset in a single typeface; the samples are now labelled directly and descriptively on every panel; each y-axis carries a self-explanatory title and the bars are colour-coded; the western-blot panel has been re-prepared from the original membrane so that the bands are clear, correctly proportioned and contiguous; and the x-axis of panel D has been corrected. We address each point individually below.

Reviewer's point	Action taken
**Inconsistent fonts**	All text in Figure 3 — blot labels, panel letters, axis titles, tick labels and annotations — is now set in a single typeface (Arial) throughout. The previous mixture of Times New Roman on the blot and a sans-serif font on the graphs has been eliminated.
**Figure confusing / cannot stand on its own**	The figure has been comprehensively rebuilt to be self-contained. Each y-axis now carries an explicit descriptive title (for example, "pERK 1/2 expression normalized by GAPDH", "pERK/Total ERK", "Fold Change in DUSP6 expression" and "Fold Change in ERK 1/2 expression"), the bars are colour-coded by treatment, and the separate numbered legend key has been removed, so every panel can be read without reference to an external key.
**Dashed line for the "attached" / excluded bands**	The blot has been re-prepared directly from the original membrane image so that the four lanes shown are contiguous and were not spliced or assembled from separated regions. As there is no longer any junction between non-adjacent lanes, a dividing (dashed) line is not applicable. The full-length, unprocessed blot images remain available in the Supplementary Data for verification.
**"Stretched and very thin" bands**	The pERK1/2 and ERK1/2 panels have been re-cropped from the source membrane at the correct aspect ratio, so the bands are no longer stretched or attenuated and now reflect the intensity and morphology of the original blot.
**Sample labelling (per a previous comment)**	The blot lanes and all x-axes are now labelled directly as Ctrl, LPS, LPS + EGCG-5 µM and LPS + EGCG-10 µM, following the convention adopted for Figure 2 (Reviewer Comment 8) and applied across all figures (Reviewer Comment 11).
**Panel order (mRNA before protein)**	*We have retained the protein-first arrangement. The detailed, referenced rationale for this ordering was set out in full in our response to Reviewer Comment 7 (Section C), and we respectfully refer the Reviewer to that response rather than repeat it here.*
**Incorrect x-axis label for Figure 3-D**	The erroneous x-axis label ("EGCG (µM)") has been replaced with the correct treatment labels (Ctrl, LPS, LPS + EGCG-5 µM, LPS + EGCG-10

Reviewer's point	Action taken
	μM). The quantity plotted is identified by the y-axis title ("pERK/Total ERK").

Manuscript Changes

All changes for this comment are confined to Figure 3 itself, which is provided in the updated figure file. The Figure 3 legend already describes the panels in the order shown and already states that the full-length blots are provided in the Supplementary Data; no change to the manuscript text was therefore required.

Summary

Figure 3 has been comprehensively revised: a single typeface throughout; direct, descriptive sample labels on every panel; self-explanatory, colour-coded y-axes; a re-prepared, contiguous western-blot panel with clear bands of correct proportions; and a corrected panel-D x-axis. ***The protein-first panel order has been retained for the reasons detailed in our response to Comment 7.*** We are grateful to the Reviewer for this scrutiny; the figure is now considerably clearer and fully self-contained.

Reviewer Comment 13

For Figure 4, again, "1" "2" "3" "4" is not proper labeling of samples. mRNA expression should go first, followed by ELISA. Also, the authors should explain why they used ELISA instead of western blotting? Also, there is no need to use numbers…this should have been a bar graph, and the numbers succinctly shown in the text of the manuscript. Figure 4B is also missing the axes borders…And why did you include EGCG of 1 uM and 20 uM concentrations??? This is not consistent with other figures.

Authors' Response

We thank the Reviewer for this careful and constructive critique of Figure 4, which we have revised extensively in response. Former panel A, previously a numerical table, is now a grouped bar graph; both panels carry direct, descriptive sample labels in place of numeric indices; panel B has been redrawn with explicit axis borders; and the panels are colour-coded by cytokine. We have additionally added two short, referenced rationales to the Methods — one explaining the use of ELISA for these secreted cytokines, and one explaining the concentration range employed in this assay. Each point is addressed individually below.

Reviewer's point	Action taken
**Sample labelling ("1, 2, 3, 4")**	The numeric sample indices and the separate legend key have been removed; both panels now label the treatments directly as Ctrl, LPS, LPS + EGCG-5 μM and LPS + EGCG-10 μM, with the additional dose levels written out in panel A. This follows the convention adopted for Figure 2 (Comment 8) and applied across all figures (Comment 11). The corresponding numbered key has also been removed from the Figure 4 legend (tracked change).
**mRNA panels before ELISA (protein)**	We have retained the protein-(ELISA)-first arrangement. The rationale for presenting the functional protein readout before the transcript readout is

Reviewer's point	Action taken
	identical to that set out in full in our response to Comment 7 (Section C), to which we respectfully refer the Reviewer rather than repeat it here.
**Why ELISA rather than western blotting**	TNF-α, IL-1β and MCP-1 are secreted cytokines released into the extracellular medium rather than retained intracellularly; they are therefore quantified directly in the conditioned supernatant by ELISA, the standard quantitative immunoassay for soluble cytokines, whereas immunoblotting is suited to cell-associated protein from lysates. A sentence stating this, with a supporting reference, has been added to the Methods (tracked change).
**Panel A should be a bar graph, with the values given in the text**	Former panel A (a numerical table) has been replaced by a grouped bar graph showing cytokine secretion across the concentration series. The corresponding numerical values (percentage secretion ± SD at each concentration) are reported succinctly in the Results text.
**Figure 4B missing axis borders**	Panel B has been redrawn with explicit axis borders, a defined y-axis carrying tick marks and title, and an x-axis baseline on which the bars sit.
**Inclusion of 1 µM and 20 µM (inconsistency with other figures)**	These concentrations are retained deliberately, as they provide a more complete dataset. The cytokine experiment is the concentration–response characterisation of EGCG's anti-inflammatory effect, for which a wider range than the 5–10 µM working concentrations was used so that the full curve could be resolved: 1 µM (near-inactive) defines the lower baseline, 5 and 10 µM span the steep active region, and 20 µM defines the upper plateau, confirming saturation. Adequate sampling of both the baseline and the plateau is a prerequisite for accurate concentration–response characterisation, and the informative 5 and 10 µM doses identified here were then carried forward to the mechanistic figures. A sentence stating this rationale, with a supporting reference, has been added to the Methods (tracked change).

Manuscript Changes

The following tracked changes have been made (all other points are addressed within the revised figure):

1. Methods (ELISA paragraph): a sentence has been added explaining that the secreted cytokines were quantified by ELISA, the standard immunoassay for soluble cytokines, rather than by immunoblotting (Stenken & Poschenrieder, Anal Chim Acta 2015).

2. Methods (same paragraph): a sentence has been added explaining that the broader 1–20 µM range was used in the cytokine assay to resolve the full concentration–response curve (lower baseline, active region, and upper plateau), a prerequisite for accurate concentration–response characterisation (Sebaugh, Pharm Stat 2011).

3. Figure 4 legend: the redundant numbered conditions key for panel B ("Conditions: 1, untreated chondrocytes; 2, …") has been deleted, since the figure now carries direct, descriptive labels.

4. References: *two new entries have been added: Sebaugh JL, Pharm Stat. 2011;10(2):128-34; and Stenken JA, Poschenrieder AJ, Anal Chim Acta. 2015;853:95-115.*

Summary

Figure 4 has been extensively revised: former panel A is now a grouped bar graph, both panels carry direct descriptive sample labels with the numbered legend key removed, panel B has explicit axis borders, and the numerical values are reported in the text. The protein-(ELISA)-first order is retained for the reasons given in our response to Comment 7. We have added concise, referenced rationales to the Methods for the use of ELISA (secreted cytokines) and for the concentration range employed, and we have retained the 1 and 20 µM data points as they complete the concentration–response curve. We are grateful to the Reviewer; the figure and its rationale are now substantially clearer.

Reviewer Comment 14

For Figure 5, again, "1" "2" "3" "4" is not proper labeling of samples. Please reorder Figure 5 so that mRNA expression is first, followed by protein expression, for each candidate gene/protein. Again, I don't understand why you have the legend for Figure 5 in between the text for SOX4 and ADAMTS5?? This makes it very hard to follow the manuscript! There is also no need to manipulate the bands such that are are so thin, as it makes it difficult to analyze visually. Please use the original bands.

Authors' Response

We thank the Reviewer for these comments on Figure 5, which we have revised substantially. The figure has been re-prepared in colour with direct, descriptive sample labels in place of numeric indices, and the western-blot panels have been redrawn from the original membrane images so that the bands are shown at their proper width and intensity. We address each point individually below.

Reviewer's point	Action taken
**Sample labelling ("1, 2, 3, 4")**	The numeric sample indices and both separate legend boxes have been removed; every panel now labels the treatments directly as Ctrl, LPS, LPS + EGCG-5 µM and LPS + EGCG-10 µM, following the convention adopted for Figure 2 (Comment 8) and applied across all figures (Comment 11).
**Reorder so that mRNA precedes protein for each gene**	We have retained the protein-first arrangement within each gene (SOX4 and ADAMTS-5). The detailed, referenced rationale for presenting the protein readout before the transcript readout was set out in full in our response to Comment 7 (Section C), and applies identically here; we respectfully refer the Reviewer to that response rather than repeat it.
**Figure 5 legend appears between the SOX4 and ADAMTS-5 text**	*This placement is required by the journal's formatting rules and is not an authorial choice. The PLOS ONE submission guidelines require that each figure's caption be inserted in the manuscript text immediately after the paragraph in which that figure is first cited (read order). Figure 5 is first cited in the SOX4 paragraph, so its caption necessarily falls at that point — that is, between the SOX4 and ADAMTS-5 discussions — and we have*

Reviewer's point	Action taken
	placed it there in compliance with the guidelines (PLOS ONE Submission Guidelines, "Figures": **https://journals.plos.org/plosone/s/submission-guidelines**). We would, of course, be glad to relocate the caption if the editorial office prefers a different arrangement at the typesetting stage.
**"Thin" bands; please use the original bands**	The blot panels (SOX4, ADAMTS-5, and their GAPDH loading controls) have been re-prepared from the original membrane images at the correct, un-compressed aspect ratio, so the bands are now displayed at their original width and intensity and are no longer thin or visually attenuated. No band data have been altered, and the full-length, unprocessed blots for panels A and D remain available in the Supplementary Data for verification.

All the above changes are contained within the revised Figure 5 (provided in the updated figure file); no change to the manuscript text was required, and the position of the figure caption is fixed by the journal's formatting requirement as noted above.

Summary

Figure 5 has been revised in colour with direct, descriptive sample labels throughout and the numbered legend boxes removed, and the western-blot panels have been re-prepared from the original membranes so that the bands are shown at their proper width and intensity. The protein-first panel order has been retained for the reasons detailed in our response to Comment 7, and the position of the figure caption follows the PLOS ONE formatting requirement. We are grateful to the Reviewer; the figure is now clearer and easier to interpret.

Reviewer Comment 15

> *Figure 6 labeling is also confusing. Figures must be able to stand on their own. One cannot figure out the difference between the right and left side of the figure in terms of treatment unless you read the figure legend…*

Authors' Response

We thank the Reviewer, and we agree that the figure should be interpretable on its own. Figure 6 has accordingly been comprehensively relabelled — including explicit column headers that identify the two readouts — so that every panel, and the distinction between the two sides, can be read directly from the figure without recourse to the legend. The specific changes are detailed below.

Reviewer's point	Action taken
**Figure 6 labelling is confusing; the figure must stand on its own**	Each flow-cytometry plot in both columns is now titled directly with its treatment condition (Ctrl, LPS, LPS + 5 µM EGCG, LPS + 10 µM EGCG), replacing the former letter codes (A–D and F–I), and the separate legend box that mapped those letters to treatments has been removed. The quantification panels (E and J) likewise carry the full descriptive condition labels on their x-axes, and the bars are colour-coded by treatment. Every panel can therefore be interpreted directly from the figure, without recourse to the legend.
**The left and right sides cannot be distinguished without reading the legend**	The two sides are now distinguished explicitly on the figure itself: the left-hand column carries the header "Effect of EGCG on RANK Expression (A–E)" and the right-hand column "Effect of EGCG on RANKL Expression (F–J)", and the corresponding quantification panels are titled "Expression of RANK (%)" (panel E) and "Expression of RANKL (%)" (panel J). Together with the per-panel treatment titles, the readout shown on each side and the identity of every condition are now immediately apparent from the figure, with no need to consult the legend. The scheme follows the labelling convention adopted for Figure 2 (Comment 8) and applied across all figures (Comment 11).

All of the above changes are contained within the revised Figure 6 (provided in the updated figure file); no change to the manuscript text was required.

Summary

Figure 6 has been relabelled to stand on its own: each column is now headed by its readout ("Effect of EGCG on RANK Expression (A–E)" and "Effect of EGCG on RANKL Expression (F–J)"), every flow-cytometry plot is titled with its treatment condition, the quantification panels are explicitly titled for RANK (E) and RANKL (J), the redundant letter-key legend box has been removed, and the bar charts carry full descriptive labels and colour-coding. The figure can now be interpreted without reference to the legend. We are grateful to the Reviewer for prompting this improvement in clarity.

Note on numbering: Reviewer #3's comments proceed directly from Comment 15 to Comment 17; there is no Comment 16 in the report we received. We have retained the Reviewer's original numbering and therefore provide no response under a "Comment 16".

Reviewer Comment 17

> *Figure 7 labeling of A, B, C, D..etc is not consistent with other figures. Please make sure you use proper and consistent labeling of samples.*

Authors' Response

We thank the Reviewer, and we have brought Figure 7 into line with the labelling used throughout the manuscript. The previous alphabetical panel codes — A–D for the Rhodamine-staining series and F–I for

the Janus Green-staining series — together with the separate legend key that mapped those letters to treatments, have been removed.
Each microscopy panel is now titled directly with its treatment condition (Ctrl, LPS, LPS + 5 µM EGCG, LPS + 10 µM EGCG) in both staining series, and the two quantification panels (E and J) carry the same descriptive condition labels on their x-axes, with the bars colour-coded by treatment and each panel identified by its stain ("Rhodamine staining", "Janus Green staining").
Figure 7 therefore now uses the same direct, descriptive sample labelling adopted for Figure 2 (Comment 8) and applied consistently across all figures (Comment 11), and can be read without reference to the legend.

All of the above changes are contained within the revised Figure 7 (provided in the updated figure file); no change to the manuscript text was required.

Summary

Figure 7 has been relabelled for consistency with the other figures: the alphabetical panel codes (A–D and F–I) and the separate legend key have been removed, and every panel — microscopy images and bar charts alike, in both the Rhodamine- and Janus Green-staining series — is now labelled directly with its treatment condition (Ctrl, LPS, LPS + EGCG-5 µM, LPS + EGCG-10 µM). The sample labelling is now uniform across all figures in the manuscript. We are grateful to the Reviewer for prompting this consistency.

Reviewer Comment 18

Figure 8…again, again, "1" "2" "3" "4" etc. is not proper labeling of samples. You did not include the original blot for this figure. Also, you did not include band sizes in the figure…Also, there are no panels indicated in this figure…like Figure 8A, 8B, etc. However, it is included in the text…please fix the figure to be consistent with the text! This figure needs to be redone completely. There is no need for a line graph…this is not consistent with how data were presented in other figures…please use bar graphs with the SD shown.

Authors' Response

We thank the Reviewer for this thorough critique, and we have rebuilt Figure 8 in full. The samples are now labelled directly rather than numerically; the figure is organised into clearly lettered panels (A–D) that match the in-text callouts; the former line graphs have been replaced by bar graphs with standard deviations; the molecular weights are reported; and the full-length, uncropped blot is now provided as Supplementary Data. Each point is addressed individually below.

Reviewer's point	Action taken
**Sample labelling ("1, 2, 3, 4…")**	The numeric sample indices and the separate numbered legend key have been removed. The blot lanes (panel A) and the x-axes of the bar graphs (panels B–D) are now labelled directly with the eight treatment conditions — Ctrl, LPS, LPS + EGCG-5 µM, LPS + EGCG-10 µM, siRNA, LPS + siRNA, LPS + siRNA + EGCG-5 µM, and LPS + siRNA + EGCG-10 µM — following the convention adopted for Figure 2 (Comment 8) and applied across all figures (Comment 11).

Reviewer's point	Action taken
**The original blot was not included**	The full-length, uncropped western blot for panel A (PAR-2) is now provided as Supplementary Data, with its lanes mapped to the eight conditions, and it is explicitly referenced in the revised Figure 8 legend (tracked change).
**Band sizes were not included**	The molecular weights are now specified — PAR-2 at approximately 44 kDa and GAPDH at approximately 37 kDa — in the revised Figure 8 legend (tracked change), and the molecular-weight ladder is shown on the full-length uncropped blot provided as Supplementary Data.
**No panel labels (A, B, etc.) although referenced in the text**	The figure is now divided into explicitly lettered panels that correspond to the in-text callouts: (A) the PAR-2 / GAPDH western blot; (B) relative PAR-2 protein expression; (C) TNF-α secretion by ELISA; and (D) TNF-α mRNA expression. The figure and the text are therefore now consistent.
**The figure needs to be redone completely**	Figure 8 has been comprehensively rebuilt: direct sample labelling, explicitly lettered panels (A–D), colour-coding by condition, bar graphs in place of line graphs, stated molecular weights, and the full-length uncropped blot provided as Supplementary Data, as detailed across the other rows of this table.
**Line graphs are inconsistent with the other figures; use bar graphs with SD**	The former line graphs have been replaced. Panels B, C and D are now bar graphs with the standard deviation shown as error bars ($n = 3$) and significance indicated, presenting the data in the same format used throughout the rest of the manuscript.

Manuscript Changes

The following tracked changes have been made to the **Figure 8 legend**; all other points are addressed within the revised figure and the new Supplementary file:

1. The numbered conditions key ("Conditions: 1, control; 2, LPS; …; 8, …") and the residual "(conditions 5–8)" reference have been removed, since the figure now carries direct, descriptive labels.
2. The molecular weights have been added to the panel A description (PAR-2 ~44 kDa; GAPDH ~37 kDa).
3. A reference to the full-length, uncropped blot now provided in the Supplementary Data has been added.
4. A duplicated panel label "(B)" in the legend has been removed.

Supplementary Material

A new Supplementary file is provided containing the full-length, uncropped western blot for Figure 8A (PAR-2), annotated with the molecular-weight markers (44 kDa and 37 kDa) and with all eight lanes mapped to their treatment conditions.

Summary

Figure 8 has been completely rebuilt to address every point raised: samples are labelled directly rather than numerically; the figure is organised into clearly lettered panels (A–D) consistent with the text; the line graphs have been replaced by bar graphs with standard deviations; the molecular weights are stated; and

the full-length, uncropped blot is provided as Supplementary Data. We are grateful to the Reviewer, whose comments have substantially improved the clarity and rigour of this figure.

Reviewer Comment 19

> *Figure 8…TNF alpha mRNA expression should be first, followed by ELISA…also why using ELISA when other figures have western blots???*

Authors' Response

We are grateful to the Reviewer for returning to Figure 8, as it allows us to set out the underlying rationale in full. The comment raises two distinct questions: the method used to quantify TNF-α (ELISA rather than western blotting), and the order in which the protein/ELISA and transcript panels are presented. We address each in turn, with supporting literature. In brief, ELISA is used for TNF-α because TNF-α is a secreted cytokine whose functionally relevant pool is extracellular and is therefore best quantified in the conditioned medium, whereas western blotting is reserved consistently, throughout the manuscript for cell-associated proteins; and the protein/functional readout is presented before the transcript readout because the figure is organised by the logic of inference, in keeping with every other figure in the manuscript.

1. Why ELISA, and not western blotting, is the appropriate method for TNF-α

(a) TNF-α is a secreted cytokine, and its functionally relevant pool is extracellular. TNF-α is synthesised as a 26-kDa membrane-anchored precursor (pro-TNF-α) that is proteolytically cleaved by the TNF-α–converting enzyme (TACE/ADAM17) to release the soluble, 17-kDa mature cytokine into the extracellular space (Black et al., Nature 1997; Moss et al., Nature 1997). The biologically active species that mediates the autocrine and paracrine inflammatory signalling under study, and whose suppression by EGCG is the very point of this experiment is the secreted cytokine that accumulates in the culture medium, not a pool retained inside the cell.

(b) Because the analyte is secreted, it must be measured where it resides. Secreted TNF-α is therefore quantified directly in the conditioned supernatant by sandwich ELISA. the validated, absolutely quantitative (standard-curve–based) and highly sensitive immunoassay that is the established standard for soluble cytokines (Stenken & Poschenrieder, Anal Chim Acta 2015; manuscript ref. 85). A western blot of the cell lysate would interrogate the wrong compartment. it would detect only residual cell-associated or precursor TNF-α and would systematically miss the secreted mature cytokine that constitutes the actual functional output; it is, moreover, only semi-quantitative and far less sensitive for a low-abundance secreted protein. ELISA is thus not an arbitrary alternative to immunoblotting, but the method dictated by the biology of the analyte.

(c) Figure 8, and the manuscript as a whole is internally consistent in exactly this respect. Within Figure 8 itself the two methods are each matched to their analyte: the cell-associated species, the PAR-2 receptor (panel A), is measured by western blot of the lysate, whereas the secreted species, TNF-α (panel C), is measured by ELISA of the medium. The same principle governs every figure: cell-associated and intracellular proteins (the PAR-2 receptor, ERK1/2, SOX4 and ADAMTS-5) are immunoblotted from lysates, while secreted cytokines (TNF-α, IL-1β and MCP-1) are quantified by ELISA from supernatants.

There is therefore no inconsistency between figures; the difference the Reviewer notes simply reflects the correct, analyte-appropriate pairing of method to molecule.

(d) This dual-method strategy is the standard representation in the field, including in the most directly comparable prior work. Akhtar and Haqqi (Arthritis Res Ther 2011; manuscript ref. 35), an EGCG study in primary human osteoarthritic chondrocytes quantified secreted TNF-α, IL-6 and IL-8 in culture supernatants by specific ELISAs, analysed the cell-associated signalling proteins (IRAK-1 and TRAF-6, with NF-κB and MAPK activation) by immunoblot, and assessed the corresponding transcripts by RT-PCR. Our use of ELISA for the secreted cytokine and immunoblotting for the cell-associated receptor follows precisely this established practice.

2. Why the protein/ELISA readout is presented before the transcript readout

We fully take the Reviewer's central-dogma point that transcription precedes translation. After careful consideration we have nonetheless retained the order in which the protein/functional readout precedes the transcript readout, for the four mutually reinforcing reasons set out in detail in our response to Comment 7 (Section C). These apply with particular force to Figure 8, and we summarise them here as they bear on this specific figure.

(1) Results figures are organised by the logic of inference, not by the biosynthetic order of the molecules assayed. The experimental logic of Figure 8 is sequential and directional; panel A confirms that PAR-2 has been silenced at the protein level; panel C then measures the functional consequence of that silencing, the secreted TNF-α output; and panel D finally corroborates the effect at the transcript level. The directly measured functional endpoints (receptor knockdown and secreted cytokine) are the primary results that answer the question posed; the mRNA is shown last to demonstrate that the same effect is also reflected transcriptionally. *Presenting the functional outcome first and the mechanism second mirrors the inferential flow of the study and is the more informative sequence for the reader.*

(2) The secreted TNF-α protein is the functionally decisive species. TNF-α is the effector cytokine that drives the inflammatory phenotype under investigation; it is the protein, released into the medium, that exerts the biological effect, whereas the transcript is an intermediate on the way to it. It is therefore appropriate that the functionally decisive, directly measured readout is presented as the principal result, with the transcript shown subsequently as supporting mechanism.

(3) mRNA abundance is an unreliable surrogate for protein abundance, so the protein result must stand in its own right. *Genome-wide analyses have repeatedly shown that steady-state transcript levels explain only a minority, on the order of 40% of the variance in protein abundance, the remainder being governed by post-transcriptional, translational and protein-degradation control* (Vogel & Marcotte, Nat Rev Genet 2012; Liu, Beyer & Aebersold, Cell 2016). The secreted-protein measurement therefore cannot be presupposed from the transcript and must be presented as a primary endpoint in its own right. Indeed, the noteworthy result here is precisely the concordance between the secreted-protein and transcript data, a concordance that is demonstrated, not assumed, by showing the measured protein first and then establishing that the mRNA agrees.

(4) Protein/functional-first ordering is uniform across the manuscript and is mirrored in the Methods. Every multi-panel functional figure presents the protein or functional readout first and the transcript second (Figures 2, 3, 4, 5 and 8), and the Methods are arranged in the same order ("Western blotting" and the ELISA procedures precede "RNA extraction, cDNA synthesis, and quantitative PCR").

Reordering Figure 8 alone would make it the single exception, break the parallelism that lets the reader interpret every figure in the same way, and introduce the very figure–methods mismatch that uniformity avoids.

For these reasons we have retained both the use of ELISA for TNF-α and the protein/ELISA-before-mRNA panel order. *We hope the Reviewer finds this rationale complete and persuasive; should the editor nonetheless prefer, we would be glad to add a single explanatory sentence to the Methods or Discussion making this rationale explicit in the manuscript itself.*

References cited in this response

1 Black RA, Rauch CT, Kozlosky CJ, et al. *A metalloproteinase disintegrin that releases tumour-necrosis factor-alpha from cells.* Nature. 1997;385(6618):729–733. doi:10.1038/385729a0. PMID: 9034190.

2 Moss ML, Jin SL, Milla ME, et al. *Cloning of a disintegrin metalloproteinase that processes precursor tumour-necrosis factor-alpha.* Nature. 1997;385(6618):733–736. doi:10.1038/385733a0. PMID: 9034191.

3 Stenken JA, Poschenrieder AJ. *Bioanalytical chemistry of cytokines — a review.* Anal Chim Acta. 2015;853:95–115. doi:10.1016/j.aca.2014.10.009. PMID: 25467452. **[manuscript ref. 85]**

4 Akhtar N, Haqqi TM. *Epigallocatechin-3-gallate suppresses the global interleukin-1β-induced inflammatory response in human chondrocytes.* Arthritis Res Ther. 2011;13(3):R93. doi:10.1186/ar3368. PMID: 21682898. **[manuscript ref. 35]**

5 Vogel C, Marcotte EM. *Insights into the regulation of protein abundance from proteomic and transcriptomic analyses.* Nat Rev Genet. 2012;13(4):227–232. doi:10.1038/nrg3185. PMID: 22411467.

6 Liu Y, Beyer A, Aebersold R. *On the dependency of cellular protein levels on mRNA abundance.* Cell. 2016;165(3):535–550. doi:10.1016/j.cell.2016.03.014. PMID: 27104977.

References 3 and 4 are already cited in the manuscript (refs. 85 and 35). References 1, 2, 5 and 6 are provided here to substantiate this response; they are not currently inserted into the manuscript, as no change to the figure or text is required. Should the editor or Reviewer prefer, we would gladly add a brief, referenced sentence to the Methods or Discussion to make this rationale explicit in the manuscript itself.

Summary

ELISA is used for TNF-α because TNF-α is a secreted cytokine, cleaved from its membrane precursor by TACE/ADAM17 and released into the medium, where it must be quantified; western blotting is reserved, throughout the manuscript, for cell-associated proteins, so the method is matched to the biology of each analyte rather than applied inconsistently. The protein/ELISA readout precedes the transcript readout because the figure is organised by the logic of inference (functional outcome first, mechanism second), because the secreted protein is the functionally decisive species, because mRNA is a known-unreliable surrogate for protein, and because this ordering is uniform across every figure and the Methods. We have therefore retained both choices, and we are grateful to the Reviewer for the opportunity to make the rationale fully explicit.

Reviewer Comment 20

The paragraph that starts with "Two conclusions emerge from these data." belongs in the DISCUSSION section!

Authors' Response

We agree, and we are grateful to the Reviewer for this point. The paragraph beginning "***Two conclusions emerge from these data***." is interpretive rather than descriptive: it draws inferential conclusions from the siRNA silencing experiment and surveys the mechanistic implications of EGCG's residual activity. It is therefore better placed in the Discussion. We have moved it (tracked changes) out of the Results and into the signposted Discussion subsection "***PAR-2 is the principal upstream target of EGCG in osteoarthritic chondrocytes.***"

We selected this subsection deliberately, and for three specific reasons.

First, it is the part of the Discussion that interprets the very experiment from which the paragraph draws its conclusions. The subsection is built around the PAR-2 siRNA silencing experiment of Figure 8, and the paragraph's two conclusions that PAR-2 silencing per se attenuates LPS-induced TNF-α, establishing the receptor as a critical mediator of cytokine output, and that EGCG acts predominantly, though not exclusively, through PAR-2, are precisely the theme of this subsection. Placing the interpretive synthesis alongside the discussion of the experiment that generated it is the most logical home for it.

Second, the paragraph cross-references Table 2, which is itself located in this subsection. The paragraph attributes the residual, PAR-2–independent activity to the multiple signalling axes "*detailed in Table 2," and Table 2 (the catalogue of PAR-2–independent pathways)* sits within this same subsection. Relocating the paragraph here makes that cross-reference local and immediate; in its previous position at the end of the Results it pointed forward, across the section boundary, into the Discussion.

Third, within the subsection the paragraph has been positioned so that it functions as an interpretive bridge. It now sits immediately after the text that presents the silencing data (the ~38% reduction in LPS-induced TNF-α and the blunted incremental effect of EGCG) and immediately before the text that elaborates the residual, PAR-2–independent mechanisms. The local narrative therefore runs in the logical order data → conclusions → mechanistic explanation, and the closing reference to "residual suppressive activity" leads directly into the subsequent sentence, "This residual activity in PAR-2-silenced cells is mechanistically informative…"

To integrate the relocated paragraph smoothly with the preceding text, we added a single connecting sentence of a deliberately technical character. In keeping with the Reviewer's guidance, this sentence introduces no new reference and no new claim; it simply frames the paragraph as a synthesis of the loss-of-function and concentration–response evidence. With the move complete, the Results section now ends on the directly measured siRNA and qPCR data, while the interpretive synthesis resides in the Discussion, where it belongs.

Manuscript Changes

The following tracked changes implement the relocation (all visible in the revised manuscript):

1. **Deletion from the Results.** The paragraph beginning “Two conclusions emerge from these data.” has been removed from the end of the Results (the Figure 8 / siRNA sub-section), so that the Results now close on the measured siRNA and qPCR findings.
2. **Insertion into the Discussion.** The same paragraph has been inserted into the subsection “PAR-2 is the principal upstream target of EGCG in osteoarthritic chondrocytes,” between the paragraph presenting the silencing data and the paragraph discussing the residual PAR-2–independent activity. The cross-reference to Table 2 is now local to this subsection.
3. **Technical connecting sentence.** A single sentence was added at the start of the relocated paragraph to integrate it with the preceding text: “*These loss-of-function findings, taken together with the concentration–response data, permit a precise delineation of the receptor dependence of EGCG’s anti-inflammatory action in this model.*” No new reference was introduced.

Summary

The interpretive paragraph identified by the Reviewer has been moved from the Results to the Discussion, into the subsection that interprets the PAR-2 silencing experiment and that houses the cross-referenced Table 2. It is positioned to act as the bridge between the silencing data and their mechanistic interpretation, and a single technical connecting sentence integrates it, with no new reference added. We thank the Reviewer; the manuscript’s structure is improved as a result.

Reviewer Comment 21

> *Figure 9 should have its own brief section in the Results section before discussing in the discussion section.*

Authors’ Response

We thank the Reviewer for this constructive suggestion, with which we fully agree. In the original manuscript, Fig 9 was first cited only within the Discussion, so the integrated model was effectively interpreted before it had been formally presented. To remedy this, we have added a dedicated subsection at the end of the Results that introduces Fig 9 in its own right and walks the reader through the model systematically, ahead of any interpretive discussion. The Discussion now refers back to a model that the Results have already established.

In line with the Reviewer’s broader point that the schematic should make the marker-to-pathway logic transparent, the revised Fig 9 has been elaborated into a five-module architecture, and the new Results subsection traces each module in technical detail. Consistent with our commitment to rigorous accuracy, every node in the prose is anchored to the specific data figure in which it was measured — PAR-2 (Fig 2); the ERK axis, DUSP6, and ERK transcripts (Fig 3); the secreted cytokines TNF-α, IL-1β, and MCP-1 (Fig 4); SOX4 and ADAMTS-5 (Fig 5); RANK/RANKL (Fig 6); and mitochondrial membrane potential and mass (Fig 7) — while elements depicted for mechanistic completeness but not individually assayed in this study (for example p38, JNK, direct NF-κB readout, COX-2/PGE2, MEK1/2, MMP-13, ROS, and apoptosis) are explicitly described as canonical or established architecture rather than as endpoints

measured here. This preserves a clear boundary between what the data demonstrate and what the model contextualises.

Two further points are worth noting. First, the figure legend has been rewritten to describe the revised five-module schematic and relocated so that it now accompanies the figure at its first point of citation in the Results; the previous legend in the Discussion is shown as a tracked deletion, which may simply be rejected should the Editor prefer the legend to remain in its original position. Second, in accordance with the Reviewer's explicit instruction, no new literature citations were introduced anywhere in the added text — only internal figure cross-references are used — so the established ordering of references is left entirely undisturbed.

Specific Revisions Made (tracked changes in the revised manuscript)

1. **Results — new subsection presenting Fig 9.** A new subsection has been inserted at the end of the Results, immediately after the PAR-2-silencing (siRNA) paragraph, titled "An integrated signalling model delineates EGCG-mediated interception of the PAR-2 cascade across five effector modules." Seven paragraphs present the model systematically: the PAR-2 apical node and EGCG interception (Fig 2); module 1, inflammatory amplification (TNF-α, IL-1β, MCP-1, Fig 4; DUSP6, Fig 3E); module 2, the MAPK–ERK axis (pERK1/2 and the pERK:ERK ratio, Fig 3A–D; ERK1/ERK2 transcripts, Fig 3F); module 3, the catabolic transcriptional programme (SOX4 and ADAMTS-5, Fig 5); module 4, mitochondrial dysfunction (ΔΨm and mitochondrial mass, Fig 7); module 5, osteoclastogenic signalling (RANK/RANKL, Fig 6); and the two divergent cellular trajectories, with the requirement for PAR-2 established by the siRNA data (Fig 8).
2. **Figure 9 legend — updated and relocated.** The legend has been rewritten to describe the revised five-module figure and moved to the end of the new Results subsection, its first point of citation. The previous legend in the Discussion is marked as a tracked deletion and can be rejected if the legend is preferred in its original location.
3. **No new references.** In keeping with the Reviewer's instruction, no citations were added to the inserted text; only internal figure cross-references (Figs 2–8) are used, preserving the established reference order in full.

INSERTED TEXT (RESULTS)

An integrated signalling model delineates EGCG-mediated interception of the PAR-2 cascade across five effector modules.

The experimental findings presented above are synthesised into a unified mechanistic model in Fig 9, which positions PAR-2 as the apical node through which EGCG coordinates its chondroprotective action and traces the consequences of receptor engagement through five functionally linked downstream effector modules. In the inflamed osteoarthritic joint, a TNF-α–rich proteolytic milieu drives activation of protease-activated receptor-2 (PAR-2, encoded by F2RL1) at the chondrocyte plasma membrane, whereas EGCG acts at the very summit of this cascade by downregulating PAR-2 expression and activation at both the protein and transcript levels (Fig 2). Because every downstream module depicted in the model is contingent on signal flux through this single receptor, suppression of PAR-2 by EGCG is propagated coordinately to all five effector arms, each of which was interrogated independently in the present study using a methodologically distinct readout. The model thus renders explicit how a single upstream intervention produces the concordant, pathway-wide attenuation

documented across Figs 2–8, and frames the divergent cellular outcomes that follow from either unchecked or EGCG-restrained receptor signalling.

The first module (Fig 9, module 1) depicts inflammatory amplification, the transcriptional limb through which receptor engagement is converted into a self-reinforcing cytokine output. PAR-2 activation converges on the NF-κB and mitogen-activated protein kinase (MAPK) signalling nodes, the latter resolving into the canonical triad of p38, JNK, and ERK that together drive the pro-inflammatory and eicosanoid programme represented in the model by TNF-α, IL-6, IL-1β, and COX-2/PGE2. Of these depicted effectors, the three mediators quantified directly in this study — TNF-α, IL-1β, and MCP-1 — were each suppressed by EGCG in a concentration-dependent manner at both protein and transcript level (Fig 4), confirming that interception at the receptor is transmitted faithfully to the secreted inflammatory output. The module additionally incorporates DUSP6 as the inducible dual-specificity phosphatase that constitutes the principal negative-feedback brake on this circuit; its LPS-driven induction and EGCG-sensitive attenuation were measured directly (Fig 3E) and are positioned in the model as the endogenous counter-regulatory element of the amplification loop. The p38, JNK, and NF-κB nodes, together with COX-2/PGE2, are rendered as the established architecture of the module rather than as endpoints assayed here; the ERK arm, by contrast, was singled out for direct mechanistic dissection, as detailed in the second module.

The second module (Fig 9, module 2) isolates the MAPK–ERK axis, the specific kinase limb through which the present study traced signal transmission from receptor to nucleus. PAR-2–driven MAPK activity is relayed through MEK1/2 to the phosphorylation of ERK1/2, which in turn licenses stress-response transcription, while the same DUSP6 phosphatase that brakes the amplification loop is depicted here as the proximal negative regulator of ERK activation. This axis was characterised at the highest mechanistic resolution of any module: EGCG suppressed ERK1/2 phosphorylation and reduced total ERK1/2 abundance, with the activation-state-specific pERK1/2:total ERK1/2 ratio driven below basal levels at 10 μM (Fig 3A–D), while the ERK1 and ERK2 transcripts were concordantly downregulated (Fig 3F) and DUSP6 was attenuated (Fig 3E). The sub-basal restoration of the phosphorylation ratio, depicted in the model as the pivotal control point downstream of PAR-2, indicates preferential inhibition of kinase activation beyond the reduction in kinase abundance, consistent with combined upstream interference and preserved phosphatase tone. MEK1/2 is represented as the canonical immediate-upstream activator of ERK1/2 rather than as a node assayed directly in this work.

The third module (Fig 9, module 3) represents the catabolic transcriptional programme that couples inflammatory signalling to irreversible matrix destruction. The transcription factor SOX4 is positioned upstream of the aggrecanase and collagenase effectors ADAMTS-5 and MMP-13, whose combined activity culminates in the depicted breakdown of aggrecan and type II collagen, the two principal load-bearing constituents of the cartilage extracellular matrix. The SOX4–ADAMTS-5 limb of this programme was quantified directly: EGCG produced coordinate, concentration-dependent suppression of both SOX4 and ADAMTS-5 at protein and transcript level (Fig 5), interrupting the transcription-factor-to-protease coupling at its origin. MMP-13 is incorporated in the model as the canonical co-effector collagenase that acts in concert with ADAMTS-5 to complete matrix catabolism, and the aggrecan/collagen II breakdown shown as the terminus of the module represents the functional consequence inferred from this protease output rather than an endpoint measured here. The paired matrix insets contrast the degraded fibrillar architecture that accompanies unchecked catabolism with

the preserved matrix retained under EGCG, encapsulating the structural stakes of this arm of the cascade.

The fourth module (Fig 9, module 4) depicts the mitochondrial dimension of PAR-2–driven injury, in which receptor signalling promotes the accumulation of reactive oxygen species (ROS), disruption of the mitochondrial membrane potential (ΔΨm), and consequent apoptotic stress. The two readouts that anchor this module empirically are the thermodynamic and structural markers of mitochondrial integrity: ΔΨm, reported by Rhodamine 123 fluorescence, and mitochondrial mass, reported by Janus Green B staining, both of which were diminished by LPS and restored towards homeostatic levels by EGCG in a concentration-dependent manner (Fig 7). Consistent with the deliberately circumscribed scope of these assays, ROS accumulation and apoptotic stress are rendered in the model as the established upstream cause and downstream consequence of membrane-potential collapse rather than as parameters measured here, and the EGCG-protection inset depicts the restitution of mitochondrial integrity that accompanies receptor suppression. Positioning this module alongside the inflammatory and catabolic arms makes explicit that EGCG-mediated PAR-2 interception spares the mitochondrial compartment in parallel with its suppression of nuclear inflammatory programmes.

The fifth module (Fig 9, module 5) traces the osteoclastogenic limb that links inflamed cartilage to the subchondral bone compartment. PAR-2 activation upregulates RANKL on the chondrocyte/osteoblast-lineage cell, engaging RANK to drive pre-osteoclast commitment and, ultimately, osteoclastogenesis. The proximal, chondrocyte-resident events of this axis were measured directly by flow cytometry: EGCG bilaterally suppressed both RANKL and RANK surface expression in a concentration-dependent fashion (Fig 6), curtailing the osteoimmunological signal at its source. The pre-osteoclast and osteoclastogenesis stages shown downstream represent the established cellular consequence of sustained RANKL–RANK engagement and are depicted to situate the measured surface-receptor changes within their pathophysiological trajectory rather than as endpoints assayed in chondrocyte monoculture. This module thereby connects the receptor-level intervention characterised here to the cartilage–bone crosstalk that underlies structural progression in osteoarthritis.

The lower panels of Fig 9 integrate the five modules into the two divergent cellular trajectories that the model is constructed to contrast. Under unchecked PAR-2 signalling, the combined output of the modules — inflammation, matrix degradation, chondrocyte dysfunction, and bone resorption — converges on the progression of inflammatory joint and bone disease. Under EGCG-mediated suppression, the mirror-image outcome obtains: reduced inflammatory cytokines, preserved type II collagen and aggrecan, mitochondrial resilience, and a reduced pro-osteoclastogenic drive together favour the preservation of cartilage integrity and bone health. The convergence of five independently measured effector arms onto a single upstream receptor, all concordantly reversed by EGCG, is the central relationship that the model formalises; the requirement for that receptor is itself established quantitatively by the siRNA silencing data (Fig 8), which identify PAR-2 as the predominant, though not exclusive, node through which EGCG exerts its effect. Throughout the schematic, elements rendered in green denote EGCG-mediated downregulation and protection, whereas elements in red denote the inflammatory, catabolic, mitochondrial, and osteoclastogenic signalling that proceeds when the receptor is left unrestrained.

UPDATED FIGURE LEGEND (RESULTS)

Fig 9. Integrated signalling model of EGCG's chondroprotective mechanism in osteoarthritic chondrocytes. Schematic synthesis integrating the experimental findings of this study into a unified,

PAR-2–centred framework. In the inflamed joint, a TNF-α–rich proteolytic milieu activates protease-activated receptor-2 (PAR-2; gene F2RL1) at the chondrocyte surface, while EGCG acts at the apex of the cascade by downregulating PAR-2 expression and activation. Five functionally linked downstream modules are depicted: (1) inflammatory amplification, in which NF-κB and the p38/JNK/ERK MAPK arms drive induction of TNF-α, IL-6, IL-1β, and COX-2/PGE2 under DUSP6-mediated negative feedback; (2) the MAPK–ERK axis, in which MEK1/2-dependent ERK1/2 phosphorylation licenses stress-response transcription and is counter-regulated by the DUSP6 phosphatase; (3) the catabolic transcriptional programme, in which SOX4 drives ADAMTS-5 and MMP-13 expression and consequent aggrecan/collagen II breakdown; (4) mitochondrial dysfunction, comprising ROS accumulation, ΔΨm disruption, and apoptotic stress; and (5) osteoclastogenic signalling, in which RANKL/RANK upregulation promotes pre-osteoclast commitment and osteoclastogenesis. The lower panels contrast the two divergent trajectories: unchecked PAR-2 signalling (inflammation, matrix degradation, chondrocyte dysfunction, and bone resorption) drives progression of inflammatory joint and bone disease, whereas EGCG-mediated suppression (reduced inflammatory cytokines, preserved collagen II/aggrecan, mitochondrial resilience, and reduced pro-osteoclastogenic drive) favours preservation of cartilage integrity and bone health. Green elements denote EGCG-mediated downregulation and protection; red elements denote PAR-2–driven inflammatory, catabolic, mitochondrial, and osteoclastogenic signalling.

Summary

In summary, Fig 9 now has its own brief subsection within the Results, in which the integrated model is presented module by module before it is interpreted in the Discussion — directly addressing the Reviewer's request. The legend has been updated to the revised figure and relocated to the Results, and no new references were introduced.

Reviewer Comments 22 and 23

Comment 22. *There is no need for all these tables! I don't understand why the authors could not have simply summarized all that information in the discussion!*

Comment 23. *The discussion section is extremely long and difficult to read with unnecessary tables.*

Authors' Response

We thank the Reviewer for raising the question of readability, which we take seriously. Having considered the point carefully, we respectfully but firmly submit that the three summary tables in the Discussion (Tables 2–4) cannot be removed, for a reason that extends well beyond their individual merit. Each of these tables is the evidentiary instrument on which an already-accepted response to another reviewer's concern directly rests; they are woven into the agreed revision record of this manuscript and are not discretionary stylistic additions. We document this cross-referenced provenance in detail first, because it is the crux of our response, and then explain why removal would, in any event, be counterproductive to the very concern about length raised in Comment 23.

The role of each Discussion table extends across the full reviewer panel. Each is the basis on which a specific, already-accepted response to another reviewer was constructed:

1. **Table 2 (PAR-2-independent signalling pathways).** ***This table is the evidentiary basis of our accepted response to Reviewer #2, Comment 3***, which, noting that the siRNA data attribute only ~38% of LPS-induced TNF-α output to PAR-2 requested that "the discussion should more clearly frame EGCG as a multifunctional agent where PAR-2 is a predominant node rather than an exclusive target." Our response established precisely that framing by reference to Table 2, which catalogues the well-characterised PAR-2-independent axes (NF-κB/IKKβ, AP-1/JNK, Nrf2–ARE, PI3K–Akt–mTORC1, JAK–STAT3, AMPK) that account for the residual activity in silenced cells. ***The same table underpins our response to Reviewer #2, Comment 4*** (the Nrf2–ARE and AMPK axes supporting the refined mitochondrial claims) and the relocated mechanistic paragraph requested in the present Reviewer's own Comment 20. Deleting Table 2 would gut all three of these responses.
2. **Table 3 (EGCG content across 36 commercially consumed green teas).** ***This table is the evidentiary basis of our accepted response to Reviewer #1 (Major Concern), Comment 5***: "The claim that the tested EGCG concentrations are physiologically achievable requires stronger supporting evidence." Our response met that challenge by presenting the per-variety data in Table 3 in full, quantifying the bio-accessible (luminal) EGCG dose and demonstrating that it exceeds the 5–10 μM working range by one-and-a-half to two-and-a-half orders of magnitude. ***The same table is also the basis of our response to the present Reviewer's own Comment 6 on concentration selection***. Deleting Table 3 would remove the quantitative evidence on which both responses depend.
3. **Table 4 (animal in vivo, human ex vivo, and human patient evidence).** ***This table is the evidentiary basis of our accepted response to Reviewer #1 (Major Concern), Comment 1*** — the single most consequential criticism levelled at the manuscript: "The study is entirely based on an in vitro model, which limits its biological and clinical relevance." Our response addressed this by framing the in vitro findings within the structured, multi-level translational evidence hierarchy set out in Table 4, which arranges the in vivo, ex vivo, and clinical corroboration of the PAR-2–ERK1/2–TNF-α/SOX4/ADAMTS-5 cascade by ascending biological fidelity. Deleting Table 4 would dismantle the manuscript's entire answer to Reviewer #1's principal concern.

This cross-referenced provenance is, we submit, decisive, and we ask that it be weighed carefully. ***The three Discussion tables are the accepted instruments by which two of this manuscript's three reviewers — and, in Comments 6 and 20, the present Reviewer — had their concerns resolved.*** To remove them, as Comment 22 proposes, would not be a neutral act of tidying; it would withdraw the agreed responses to Reviewer #1's two Major Concerns (the in vitro-only limitation and the physiological achievability of the working concentrations) and to Reviewer #2's concerns on pathway specificity and mitochondrial claims, thereby re-opening points that the revision has already closed to those reviewers' satisfaction. We could not act on the present comment without simultaneously placing the manuscript back out of compliance with the documented requests of the other reviewers, an outcome that would serve neither the reviewers nor the readership.

Even setting this provenance aside, the two comments stand in tension with each other. Comment 22 proposes that the tabulated material be *"simply summarized ... in the discussion"* that is, converted into narrative prose, while Comment 23 objects that the Discussion is already too long. These two remedies pull in opposite directions. A table is, by construction, a device for compressing dense, multi-dimensional, comparative information into a form that can be apprehended at a glance. ***Rendering the contents of Table***

3 (36 tea varieties, each with several dose metrics) or Table 4 (multiple studies across three tiers of biological fidelity, each with distinct models, interventions, and outcomes) as continuous prose would not shorten the Discussion; it would lengthen it substantially, while making the comparative relationships far harder to extract. The tables are therefore not the cause of the Discussion's length but the principal means by which that length is contained. Far from inflating the section, they streamline it.

Relatedly, the information in these tables is intrinsically tabular: ***it consists of structured comparisons across rows and columns (varieties × dose metrics; studies × model level × outcome; pathways × mechanism × OA relevance)*** that are natively suited to a matrix and poorly suited to a sentence. We have deliberately kept the narrative concise by discussing only representative exemplars and referring the reader to the table for the complete catalogue. ***The Discussion is, moreover, explicitly signposted: it is organised into six labelled thematic subsections, and each table sits within the subsection whose argument it supports, at its point of first relevance***; **Table 2** within the discussion of PAR-2-independent mechanisms; **Table 3** within "Physiological and pharmacokinetic considerations" and "Selection of the LPS and EGCG concentrations: physiological versus supraphysiological context"; and **Table 4** within "Translational context across animal, human-tissue, and clinical evidence." A reader can therefore navigate the section by theme and consult each table exactly where it is needed, rather than encountering the tables as an undifferentiated block.

We would add that the present length of the Discussion is itself, in large part, a direct consequence of revisions requested across this review: the section was expanded to address the concentration-selection rationale, the bioavailability and pharmacokinetic considerations, the residual-activity question, and the translational context, among others. The resulting depth is deliberate. ***It allows the reader not only to appreciate the significance of the PAR-2–centred mechanism we report, but also to identify, in concrete terms, the priorities for future work that the study defines — purpose-designed in vivo PAR-2 studies, synovial-fluid pharmacokinetic quantification, and multi-stimulus and co-culture models. A Discussion that discharges these functions serves the field, and future investigators, better than a shorter one that omits them.***

Finally, the use of summary and synthesis tables to support interpretation is both conventional and well-founded. Authoritative guidance frames the Discussion as an exercise in synthesis and integration rather than narration (Hess, *Respir Care* 2004), and the cognitive-science literature on working-memory limitation establishes that structured, simultaneously presented information reduces extraneous cognitive load relative to extended prose (Sweller, *Cognit Sci* 1988; Mayer & Moreno, *Educ Psychol* 2003), which is to say that, for comparative material of this kind, a well-designed table is easier to read, not harder. The embedding of synthesis and comparison tables within or adjacent to the Discussion of original and review articles is widespread in high-impact journals for precisely this reason.

Summary

In summary, the three Discussion tables are retained for three reinforcing reasons. First and foremost, each is the evidentiary basis of an already-accepted response to a specific concern of Reviewer #1 or Reviewer #2 (and, in Comments 6 and 20, of the present Reviewer), so that removing them would re-open formally resolved points and place the manuscript back out of compliance with the other reviewers. Second, converting the tabulated material to prose would lengthen rather than shorten the Discussion, directly worsening the concern of Comment 23. Third, the section is already signposted into labelled subsections that allow a reader to consult each table exactly where it is relevant. We are confident that the tables aid,

rather than impede, comprehension, and we have therefore not altered the manuscript in response to these comments. Should the Editor nonetheless prefer it, we would gladly add a single orienting sentence at the head of the Discussion directing readers to the tables; we believe, however, that the existing subsection structure already achieves this.

References cited in this response

Hess DR. How to write an effective discussion. *Respir Care*. 2004;49(10):1238–1241.

Sweller J. Cognitive load during problem solving: effects on learning. *Cognit Sci*. 1988;12(2):257–285.

Mayer RE, Moreno R. Nine ways to reduce cognitive load in multimedia learning. *Educ Psychol*. 2003;38(1):43–52.

Concluding remarks

We hope that we have addressed the reviewers' comments to the best of our abilities, and that the revised manuscript now meets the standard required for publication in PLOS ONE.

We are grateful to the Academic Editor and to all three reviewers for their careful and constructive appraisal, which has materially improved the work.

We thank you for your time and consideration, and we look forward to a positive response.

With kind regards,

Dr. Yajnavalka Banerjee, PhD, PGDME, FRSB, FHEA

Associate Professor of Biochemistry
Mohammed Bin Rashid University of Medicine and Health Sciences (MBRU)
Dubai, UAE
Email: Yajnavalka.Banerjee@dubaihealth.ae
Phone: +971-0568345125
