# Supplementary material for: Epigallocatechin-3-gallate attenuates PAR-2–mediated inflammatory signaling and preserves markers of mitochondrial integrity in osteoarthritic chondrocytes
Source: PLoS One. 2026 Nov 2;21(11):e0357766. doi: 10.1371/journal.pone.0357766 (PMC13726950; doi:10.1371/journal.pone.0357766)
Supplement: Supplementary file 5 [file pone.0357766.s006.docx]

**Response to Reviewers - Second Round of Revision**

*Manuscript PONE-D-26-17270R1 - Epigallocatechin-3-gallate Attenuates PAR-2–mediated Inflammatory Signaling and Preserves Markers of Mitochondrial Integrity in Osteoarthritic Chondrocytes*

**Reviewer - 1**

**Reviewer’s General Assessment (Prelude)**

*I appreciate the considerable effort the authors have invested in revising the manuscript. The major concerns raised during the previous round of review have been addressed satisfactorily. The statistical analysis has been strengthened through the implementation of one-way ANOVA with appropriate post hoc testing, the rationale for the inflammatory model has been expanded, the discussion has been substantially improved, and the claims regarding mitochondrial protection have been appropriately moderated.

The manuscript is technically sound, the experiments are well organized, and the conclusions are generally supported by the presented data. The incorporation of PAR-2 silencing experiments further strengthens the mechanistic interpretation of the findings.*

*I have only a few minor suggestions before publication:*

**Authors’ Response**

We sincerely thank Reviewer #1 for this encouraging assessment and for recognising the extensive revisions made in the previous round. We are grateful that the statistical analysis, rationale for the inflammatory model, expanded Discussion, moderated mitochondrial claims, and PAR-2 silencing experiments have addressed the Reviewer’s major concerns. We have considered the remaining minor suggestion carefully and have implemented a comprehensive, manuscript-wide calibration of the relevant interpretive language, as detailed below.

**Reviewer 1, Comment 1**

*Consider replacing a few definitive expressions such as “establishes” or “confirms” with more cautious wording such as “supports” or “suggests”, given that the conclusions are derived from an in vitro model.*

**Authors’ Response**

We thank the Reviewer for this precise and constructive suggestion, which we fully agree improves the calibration of the manuscript. We undertook a systematic audit of the Abstract, final Introduction paragraph, Results text and figure legend, integrated mechanistic model, Discussion, translational context, Limitations, and Conclusions. We revised 33 definitive formulations across 24 manuscript paragraphs, consolidated into 22 location-based entries below. The revisions replace categorical claims of proof, causality, target exclusivity, or translational significance with language that more accurately reflects evidence derived from an in vitro model, using formulations such as “reports,” “supports,” “suggests,” “may,” “candidate,” “potential,” “contributor,” “involvement,” and “association.”

The audit was deliberately selective. We retained definitive terminology when it describes a directly observed assay result, a routine methodological validation, or a finding attributed to cited external literature, because those usages do not overstate the causal or translational reach of the present in vitro study. All newly revised manuscript wording is displayed in red. No references were deleted, added, renumbered, reordered, or moved, and no figures, tables, captions, sections, or existing paragraphs were relocated.

**Specific Revisions Made**

*The page numbers below refer to the revised Round 2 manuscript after incorporation of the red-font changes.*

| **Manuscript location** | **Exact wording revision** | **Reason for calibration** |
| --- | --- | --- |
| **1. Abstract, p. 2** | **Original:** “Here, we demonstrate that”; “establishing PAR-2 as the predominant, though not exclusive, target”; “These findings identify … and delineate a mechanistic cascade” **Revised:** “Here, we report that”; “supporting PAR-2 as a predominant, though not exclusive, mediator”; “These findings support PAR-2 as a candidate upstream target … and suggest a mechanistic model involving …” | Removes categorical demonstration, target, and causal-cascade language from the study summary while preserving the measured direction and magnitude of the findings. |
| **2. Introduction, final paragraph, p. 6** | **Original:** “to establish mechanistic causality”; “we provide the first evidence that EGCG functions as a targeted modulator”; “advancing its candidacy … to a pathway-specific candidate for disease modification in OA” **Revised:** “to assess mechanistic dependence”; “we provide evidence that EGCG may function as a targeted modulator”; “supporting its investigation as a pathway-relevant candidate in OA” | Reframes the study aim and anticipated contribution as an in vitro mechanistic assessment rather than proof of causality or disease modification. |
| **3. Results—PAR-2/ERK1/2 axis, p. 19** | **Original:** “these findings delineate a coordinated, multi-level inhibitory action” **Revised:** “these findings support a coordinated, multi-level inhibitory action” | Maintains the integrated interpretation but avoids presenting the observed associations as a fully delineated causal mechanism. |
| **4. Results—SOX4, p. 21** | **Original:** “establishing transcriptional repression as the predominant regulatory mechanism” **Revised:** “supporting transcriptional repression as a likely regulatory mechanism” | Acknowledges that concordant mRNA and protein changes support, but do not uniquely prove, the regulatory mechanism. |
| **5. Results—ADAMTS-5, p. 22** | **Original:** “delineates a coherent mechanistic cascade whereby EGCG interrupts” **Revised:** “supports a coherent mechanistic model in which EGCG may interrupt” | Changes a definitive cascade claim to a model-based interpretation appropriate to the experimental design. |
| **6. Results—RANK/RANKL, p. 23** | **Original:** “establishes that EGCG exerts coordinated regulatory control” **Revised:** “suggests that EGCG may exert coordinated regulatory effects” | Avoids inferring control of osteoclastogenic crosstalk from surface-expression measurements alone. |
| **7. Results subsection heading, p. 25** | **Original:** “siRNA-mediated PAR-2 silencing establishes PAR-2 dependence” **Revised:** “siRNA-mediated PAR-2 silencing supports PAR-2 involvement” | Calibrates the heading so it describes mechanistic support rather than definitive dependency. |
| **8. Results—siRNA opening sentence, p. 25** | **Original:** “To establish the mechanistic requirement for PAR-2” **Revised:** “To assess the mechanistic contribution of PAR-2” | Accurately describes the purpose of the loss-of-function comparison without claiming that a single in vitro perturbation proves requirement. |
| **9. Figure 8 legend, p. 25** | **Original:** “establishes PAR-2 dependence”; “identifying PAR-2 as the predominant but not exclusive target” **Revised:** “supports PAR-2 involvement”; “supporting PAR-2 as a predominant but not exclusive mediator” | Aligns the legend with the moderated Results wording and distinguishes mediation from definitive target identification. |
| **10. Results—siRNA TNF-α analysis, p. 26** | **Original:** “confirming PAR-2 as a principal upstream driver” **Revised:** “supporting PAR-2 as an important upstream contributor” | Reflects that the approximately 38% reduction supports a contribution but does not establish sole or principal causation. |
| **11. Integrated model—heading and module 1, pp. 26–27** | **Original:** “An integrated signalling model delineates EGCG-mediated interception …”; “confirming that interception at the receptor is transmitted faithfully to the secreted inflammatory output” **Revised:** “An integrated signalling model summarises EGCG-associated modulation …”; “supporting an association between PAR-2-axis modulation and the secreted inflammatory output” | Reframes both the model heading and the module text as a synthesis of observed associations rather than a demonstrated directional transmission pathway. |
| **12. Integrated model—synthesis, p. 30** | **Original:** “the requirement for that receptor is itself established quantitatively” **Revised:** “involvement of that receptor is supported quantitatively” | Avoids overstating receptor requirement while retaining the quantitative value of the siRNA experiment. |
| **13. Discussion subsection heading and opening, p. 32** | **Original:** “PAR-2 is the principal upstream target of EGCG …”; “The present study identifies EGCG as a targeted modulator”; “delineates a coherent upstream-to-downstream inhibitory cascade … previously demonstrated” **Revised:** “Evidence supports PAR-2 as a principal upstream mediator of EGCG action …”; “The present study supports EGCG as a potential modulator”; “supports a coherent upstream-to-downstream inhibitory model … previously reported” | Moderates the subsection heading, target status, and novelty/causality claims at the start of the Discussion. |
| **14. Discussion—PAR-2 mechanistic interpretation, pp. 32–33** | **Original:** “mechanistic centrality … is substantiated”; “establishes mechanistic causality beyond correlative dose-response data … strengthens the translational relevance” **Revised:** “mechanistic involvement … is supported”; “provides evidence of mechanistic involvement beyond correlative dose-response data, while remaining subject to the limitations of the in vitro model” | Explicitly recognises the inferential value of siRNA while adding an in vitro limitation to the causal interpretation. |
| **15. Discussion—loss-of-function synthesis, p. 33** | **Original:** “permit a precise delineation of receptor dependence”; “establishing this receptor as a critical mediator”; “preponderance … is demonstrably PAR-2-contingent, as the siRNA data quantitatively establish” **Revised:** “support interpretation of the receptor contribution”; “supporting this receptor as an important mediator”; “a substantial component … appears PAR-2-contingent, as supported quantitatively by the siRNA data” | Calibrates dependence, criticality, and quantitative attribution to the limits of the observed knockdown effect. |
| **16. Discussion—PAR-2-independent pathways, p. 34** | **Original:** “accounting for ~38% … as established by the siRNA silencing data” **Revised:** “accounting for ~38% … as supported by the siRNA silencing data” | Keeps the measured estimate while avoiding definitive attribution from a single in vitro loss-of-function experiment. |
| **17. Discussion—mitochondrial integrity, p. 39** | **Original:** “ΔΨm recovery and mitochondrial mass preservation demonstrated in the present study” **Revised:** “ΔΨm recovery and mitochondrial mass preservation observed in the present study” | Further limits the claim to the two measured structural/thermodynamic readouts and avoids implying functional bioenergetic proof. |
| **18. Discussion—joint pharmacokinetics, p. 46** | **Original:** “the mechanistic proof-of-concept established here” **Revised:** “the mechanistic proof-of-concept supported here” | Moderates the description of the study when connecting it to future delivery strategies. |
| **19. Discussion—translational context, p. 50** | **Original:** “The translational significance … is substantiated by” **Revised:** “The translational context … is supported by” | Avoids implying that external corroborative evidence converts the present in vitro findings into established clinical significance. |
| **20. Limitations—evidence hierarchy, p. 64** | **Original:** “this framework demonstrates that” **Revised:** “this framework suggests that” | Maintains the contextual synthesis while preventing the evidence table from being read as proof of cross-model equivalence. |
| **21. Limitations—siRNA qualification, p. 67** | **Original:** “although the siRNA data convincingly establish PAR-2 dependence” **Revised:** “although the siRNA data support PAR-2 involvement” | Removes both the intensifier and the definitive dependency claim from the limitations statement. |
| **22. Conclusions, p. 67** | **Original:** “This study establishes EGCG as a potent inhibitor” **Revised:** “This study supports EGCG as a potential inhibitor” | Ensures the final conclusion reflects candidate-level evidence from an in vitro model rather than a definitive therapeutic designation. |

**Summary**

In summary, the manuscript now consistently distinguishes direct observations from mechanistic interpretation and translational inference. The Abstract and Conclusions use candidate-level language; the siRNA experiment is described as supporting PAR-2 involvement and contribution rather than proving dependence or causality; the integrated model is presented as an evidence-supported interpretation; and the mitochondrial statement remains confined to the measured membrane-potential and mitochondrial-mass readouts. These targeted changes fully address the Reviewer’s suggestion without altering the data, references, manuscript structure, or order of presentation. We thank the Reviewer for prompting this final refinement.

**Reviewer 1, Comment 2**

*“****The Discussion remains relatively lengthy and contains some repetition of the Results. Moderate condensation would improve readability****.”*

**Authors’ response**

We thank the reviewer for this constructive observation, with which we agree. We have condensed the Discussion along two complementary lines. First, as introduced at the previous revision, a substantial volume of corroborating detail that would otherwise have been enumerated in the narrative was consolidated into three summary tables — Table 2 (PAR-2–independent pathways), Table 3 (EGCG content across 36 green teas) and Table 4 (the animal, human-tissue and clinical evidence hierarchy). These tables remain the principal length-management device of the section: they allow the prose to reference a single consolidated source rather than re-list pathways, tea varieties, or corroborating studies sentence by sentence. In the present revision we have taken deliberate advantage of that structure and removed the passages in which the surrounding prose still duplicated the tables or the Results.

Second, we have excised sentences that restated the Results (for example, the numerical siRNA read-outs already reported for Fig. 8), collapsed the two paragraphs that twice drew the same conclusion about PAR-2 dependence, and tightened the mechanistic survey of PAR-2–independent pathways, which had been described three times over. The specific condensations are listed below, shown as ~~struck-through~~ deletions and red insertions so the changes can be followed directly. In keeping with the reviewer’s request for *moderate* condensation, the edits target redundancy and Results-repetition only; the argumentative structure, data, and conclusions are unchanged.

**No references have been removed and no table has been altered.** Every citation retained in a condensed sentence is shown in **navy**; in total these edits remove approximately 900 words of redundant or Results-restating narrative from the Discussion (about 1,140 words of prose deleted against 240 words of tighter replacement) while preserving all **[n]** citations in place and leaving Tables 2–4 untouched.

**Specific condensations (change log)**

**Legend:** ~~removed text~~	·	inserted text	·	**[n] = citation preserved**

**Edit 1. Discussion → “PAR-2 is the principal upstream target…” (2nd paragraph)**

…substantiated by the siRNA silencing experiments (Fig. 8)~~,~~ : PAR-2 knockdown alone reduced LPS-induced TNF-α secretion by ~38% ~~(~780 to ~480 pg/mL)~~ and substantially blunted the incremental efficacy of EGCG co-treatment. That EGCG retained residual suppressive activity in PAR-2–silenced cells ~~(~320 and ~240 pg/mL at 5 and 10 µM, respectively)~~ indicates that PAR-2 constitutes the predominant, though not exclusive, locus of EGCG action, consistent with parallel modulation of NF-κB or mTOR signalling as reported elsewhere **[10, 35]**. ~~This dual-arm experimental design thus establishes mechanistic causality beyond correlative dose-response data, a distinction that strengthens the translational relevance of our findings.~~

***Why:*** *Removes the pg/mL values, which merely restate the Results (Fig. 8), and a closing sentence whose causality claim is made again in the following paragraph.*

**References retained:** [10, 35]; Fig. 8

**Edit 2. Discussion → “PAR-2 is the principal upstream target…” (3rd paragraph)**

~~These loss-of-function findings, taken together with the concentration–response data, permit a precise delineation of the receptor dependence of EGCG’s anti-inflammatory action in this model. Two conclusions emerge from these data. First, PAR-2 silencing per se significantly attenuates LPS-induced TNF-α production, establishing this receptor as a critical mediator of inflammatory cytokine output in chondrocytes. Second, the marked attenuation, but not complete abolition, of EGCG’s inhibitory efficacy upon PAR-2 knockdown indicates that EGCG acts predominantly, though not exclusively, through PAR-2–dependent signaling. The residual suppressive activity in silenced cells is consistent with EGCG’s well-characterised capacity to engage multiple, mechanistically distinct signalling axes, including direct IKKβ–NF-κB inhibition, AP-1/JNK suppression, Nrf2–ARE antioxidant activation, PI3K–Akt–mTOR interference, JAK–STAT3 inhibition, and AMPK activation, each of which operates independently of PAR-2 and collectively accounts for the residual anti-inflammatory efficacy quantified in silenced cells (detailed in Table 2 below); nevertheless, the preponderance of its anti-inflammatory effect in this model is demonstrably PAR-2–contingent, as the siRNA data quantitatively establish.~~ Together, these loss-of-function and concentration–response data establish PAR-2 as a critical mediator of LPS-induced TNF-α output and show that EGCG acts predominantly, though not exclusively, through it; the PAR-2–independent axes that account for its residual activity are catalogued in Table 2.

***Why:*** *This paragraph re-states the two conclusions already drawn above and pre-lists the PAR-2-independent axes that Table 2 and the next paragraph detail. Collapsed to a single sentence.*

**References retained:** none in this paragraph

**Edit 3. Discussion → “This residual activity…” (4th paragraph)**

This residual activity ~~in PAR-2-silenced cells~~ is mechanistically informative rather than paradoxical, ~~and is~~ being consistent with the polypharmacological profile of EGCG ~~as a structurally promiscuous polyphenol engaging multiple distinct signalling axes~~. Singh et al. have ~~systematically~~ documented EGCG’s inhibitory effects on NF-κB ~~(via IKKβ and proteasomal degradation)~~, JNK-MAPK, and AP-1 in arthritis-relevant systems **[35]**; and Akhtar and Haqqi~~, using an 80-protein cytokine antibody array in primary human OA chondrocytes, demonstrated that EGCG broadly suppresses~~ showed EGCG suppresses TNF-α, IL-6, MCP-1, ~~MMP-1, MMP-3, MMP-13, and ADAMTS-4/5~~ and the MMP/ADAMTS panel through NF-κB and JNK-MAPK inhibition in ~~the identical cell type studied here~~primary human OA chondrocytes **[37]**. ~~In the context of the present manuscript, the Nrf2–Keap1–ARE antioxidant axis and the AMPK metabolic-sensing axis (detailed in Table 2) are of particular mechanistic relevance. Nrf2 activation by EGCG is a direct chemical interaction at the Keap1 sensor domain, entirely independent of GPCR signalling, that drives HO-1 and NQO1 expression and reduces mitochondrial ROS; and AMPK activation promotes mitophagic clearance of depolarised mitochondria via ULK1. Collectively, these PAR-2-independent mechanisms, encompassing NF-κB/IKKβ, AP-1/JNK, Nrf2–ARE, PI3K–Akt–mTORC1, JAK1/2–STAT3, and AMPK pathways (Table 2) provide the most parsimonious mechanistic explanation for the residual anti-inflammatory and mitoprotective activity that persists after PAR-2 ablation.~~ The Nrf2–Keap1–ARE and AMPK axes, together with the remaining PAR-2–independent pathways, are catalogued in Table 2. The PAR-2 axis remains the quantitatively predominant pathway, accounting for ~38% of LPS-induced TNF-α production ~~as established by the siRNA silencing data~~ (Fig. 8)~~; however, the multi-pathway architecture of EGCG’s chondroprotective activity suggests that partial target redundancy may confer robustness to its anti-inflammatory efficacy across the diverse stimulatory contexts of the OA joint microenvironment~~.

***Why:*** *Keeps both citations but removes the long mechanistic enumeration (NF-κB/AMPK/Nrf2 detail) that is duplicated in Table 2 and in the following paragraph.*

**References retained:** [35], [37]; Table 2; Fig. 8

**Edit 4. Discussion → “Viewed in this light…” (5th paragraph)**

~~Viewed in this light, it is instructive to consider, in a deliberately top-down manner, the specific non-PAR-2 pathways through which EGCG could plausibly exert the residual chondroprotection observed after receptor silencing. The most directly relevant are those for which EGCG has already been shown to act beneficially within osteoarthritic cartilage or chondrocytes. Foremost among these is the canonical NF-κB–JNK/AP-1 inflammatory module: in primary human OA chondrocytes EGCG suppresses an 80-protein inflammatory network, including TNF-α, IL-6, MCP-1, and the MMP and ADAMTS catabolic panel, predominantly through inhibition of NF-κB and JNK-MAPK signalling, and comparable effects are documented across arthritis-relevant systems~~ Three OA-relevant, PAR-2–independent axes are the most directly evidenced. The first is the NF-κB–JNK/AP-1 inflammatory module **[35, 37]**. ~~A second, mechanistically distinct OA-relevant axis is the cytoprotective~~ The second is the cytoprotective Nrf2-Keap1-ARE antioxidant response, in interleukin-1β-challenged human chondrocytes EGCG upregulates Nrf2, haem oxygenase-1, and NQO1, reduces reactive oxygen species, and limits chondrocyte apoptosis and senescence, ~~with these chondroprotective effects abolished by the Nrf2 inhibitor ML385, confirming pathway dependence independent of any G-protein-coupled receptor~~ effects abolished by the Nrf2 inhibitor ML385 **[38]**. ~~A third OA-specific mechanism is the restoration~~ The third is restoration of autophagic flux, where intra-articular EGCG attenuates cartilage degeneration in a post-traumatic OA model by enhancing chondrocyte autophagy through suppression of mTOR and induction of LC3, beclin-1, and reduced p62~~, a mitoprotective and pro-survival programme entirely separate from PAR-2 signalling~~ **[39]**. Together, these three ~~documented OA-relevant axes~~ axes plausibly account for the ~62% of LPS-induced TNF-α output that persists after PAR-2 knockdown ~~and reinforce the interpretation of EGCG as a multifunctional agent rather than a single-target ligand~~. Beyond these ~~directly evidenced mechanisms~~axes, EGCG also modulates ~~additional pathways that have not yet been investigated in the specific context of OA chondrocytes but are independently implicated in cartilage homeostasis and therefore represent biologically plausible contributors. The clearest example is canonical Wnt/β-catenin signalling: EGCG promotes the phosphorylation and proteasomal degradation of β-catenin and represses β-catenin/TCF-dependent transcription through a mechanism independent of GSK-3β and PP2A~~ canonical Wnt/β-catenin signalling, promoting β-catenin degradation independently of GSK-3β and PP2A **[40]**; ~~because dysregulated Wnt/β-catenin activity drives chondrocyte hypertrophy, osteophyte formation, and aberrant catabolism in the osteoarthritic joint, this axis is a credible, if as-yet-unexamined, additional node of EGCG action in cartilage.~~ a credible but as-yet-unexamined node of EGCG action in cartilage. We emphasise~~, however,~~ that several of these ~~pathway-level~~ effects derive from single-pathway studies and that the ~~polyphenolic, redox-active~~ redox-active structure of EGCG can confound in vitro readouts~~, a caveat we discuss elsewhere~~ **[41]**; ~~the mechanisms surveyed here should therefore be regarded as~~ this survey provides a plausible, multi-pathway framework ~~that situates our siRNA findings rather than as a definitive accounting of every contributory axis, which will require dedicated dissection in primary OA chondrocytes~~rather than a definitive accounting.

***Why:*** *Same three OA-relevant axes are surveyed as above; the top-down framing, the repeated 80-protein description, and the Wnt digression are tightened. All six citations retained.*

**References retained:** [35, 37], [38], [39], [40], [41]

**Edit 5. Discussion → “Preservation of mitochondrial integrity” (closing sentence)**

~~Full bioenergetic characterisation employing mitochondria-targeted ROS probes (MitoSOX Red), luminometric ATP quantification, and Seahorse XF Analyser-based oxygen consumption rate (OCR) and extracellular acidification rate (ECAR) profiling in EGCG-treated OA chondrocytes constitutes a dedicated mechanistic extension of the present findings and is proposed as a priority objective of our forthcoming in vivo investigation.~~

***Why:*** *This future-work sentence (MitoSOX / ATP / Seahorse OCR-ECAR) is repeated almost verbatim in the Limitations; removed here and retained once, in Limitations.*

**References retained:** none in this sentence

**Edit 6. Discussion → “Physiological and pharmacokinetic considerations” (1st paragraph)**

A recurrent criticism of polyphenol studies is the use of supraphysiological concentrations. We specifically addressed this concern through Table 3, which catalogues EGCG content across 36 commercially consumed green tea varieties. ~~Notably, a single serving of Gyokuro infusion yields ~2,437 µM EGCG per cup, and Korean green teas (Daejak, Joongjak) deliver 2,030-2,053 µM per serving, concentrations that vastly exceed the 5–10 µM range used in our experiments. Indeed, the magnitude of this margin is consistent across the full range of varieties catalogued in Table 3: at the upper end, Gyokuro and superior Sencha infusions deliver approximately 676 to 2,705 µM EGCG per serving, while traditionally processed Chinese Lu’an Guapian (1,444 to 2,014 µM) and the Korean Woojeon, Sejak, Joongjak, and Daejak grades (1,890 to 2,053 µM) occupy a similar tier; even the more modestly concentrated steamed and pan-fired Jeju, Boseong, and Shizuoka teas yield 367 to 769 µM per serving. Across every entry in Table 3, therefore, the~~ As catalogued there, across every variety the EGCG dose delivered to the gastrointestinal lumen exceeds the 5 to 10 µM concentrations used here by between one-and-a-half and two-and-a-half orders of magnitude. We emphasise that these values represent the concentration presented to the intestinal lumen and mucosa rather than the systemic plasma concentration; because only a small fraction of luminal catechin is absorbed intact, the limiting step in translation is intestinal absorption and first-pass metabolism, not dietary intake **[50]**. ~~This distinction is mechanistically important and is reconciled, below, with the systemic pharmacokinetic data and with the enhanced-bioavailability and joint-targeted delivery strategies that bridge the gap between luminal exposure and effective chondrocyte concentrations.~~ Even accounting for the well-documented attenuation of oral bioavailability (rapid glucuronidation, short plasma half-life **[22]**), the margin between dietary intake and effective concentration appears substantial, supporting the translational plausibility of our findings, while systemic delivery remains the key constraint. Moreover, EGCG’s efficacy aligns with our group’s prior demonstrations that curcumin **[17]** and oleocanthal **[18]** similarly suppress the PAR-2 axis in chondrocytes, ~~reinforcing the paradigm that this receptor represents a tractable nutraceutical target in OA. The convergence of three structurally unrelated polyphenols on PAR-2 suggests that this pathway may constitute a common pharmacological node amenable to dietary intervention.~~reinforcing PAR-2 as a tractable nutraceutical target and a common pharmacological node for three structurally unrelated polyphenols.

***Why:*** *The per-variety µM enumeration reproduces Table 3 line-by-line; replaced with a single sentence pointing to the table. The luminal-vs-systemic point and all four citations are retained.*

**References retained:** [50], [22], [17], [18]

**Edit 7. Discussion → “Selection of the LPS and EGCG concentrations…” (final paragraph)**

~~In reconciling these pharmacokinetic constraints with our experimental findings, several contextualising considerations are relevant. First, the present study is explicitly a proof-of-concept mechanistic investigation, its primary objective is to identify PAR-2 as a novel upstream target of EGCG in OA chondrocytes and to delineate the consequent PAR-2–ERK1/2–TNF-α/SOX4/ADAMTS-5 signalling cascade, a mechanistic objective that is entirely valid irrespective of the translational route through which effective chondrocyte concentrations are ultimately achieved. Second, chronic habitual green tea consumption, modelled as multiple daily servings over extended periods, produces a distinct pharmacokinetic profile from single-dose studies, while individual plasma peaks remain submicromolar, the sustained pattern of repeated exposure may produce cumulative tissue accumulation that single-dose pharmacokinetic studies do not capture. Third, the inflammatory OA microenvironment is characterised by increased synovial microvasculature permeability and compromised membrane integrity that may enhance local EGCG accumulation relative to healthy joint physiology. None of these considerations fully closes the quantitative gap between oral pharmacokinetics and our experimental concentrations, and we do not claim they do; rather, they collectively position the present findings as providing the mechanistic substrate for a translational programme in which joint-targeted delivery strategies, nanoparticulate encapsulation, intra-articular administration, or enhanced-bioavailability phytosome formulations. are the appropriate and necessary next steps to achieve therapeutically relevant EGCG concentrations at the level of the chondrocyte in vivo.~~In reconciling these constraints with our findings, we reiterate that the study is a proof-of-concept mechanistic investigation whose validity is independent of the eventual delivery route, and that chronic habitual consumption and the increased vascular permeability of the inflamed joint may raise local exposure above single-dose predictions. None of these considerations closes the quantitative gap, which we do not claim; rather, joint-targeted delivery, nanoparticulate encapsulation, intra-articular administration, and enhanced-bioavailability phytosome formulations remain the necessary next steps to reach therapeutically relevant chondrocyte concentrations in vivo.

***Why:*** *The three enumerated “considerations” restate the proof-of-concept scope, chronic-dosing and inflamed-joint-permeability points already made; condensed to two sentences (also fixes the stray “formulations. are”).*

**References retained:** none in this paragraph

**Effect on the manuscript**

These changes reduce the length of the Discussion and remove its overlap with the Results without loss of content, citations, or the summary tables. We are grateful to the reviewer, as the section now reads more efficiently while retaining the full evidentiary basis of each argument.

**Reviewer 1, Comment 3**

*The Western blot methodology contains a repeated description of densitometric analysis, which should be removed for clarity.*

**Authors’ Response**

We thank the Reviewer for identifying this redundancy. We agree that the Western blotting subsection contained two consecutive descriptions of the same densitometric analysis step. We have therefore removed the redundant sentence, “Band intensities were quantified by densitometric analysis using ImageJ.” The preceding sentence already specifies that band intensities were quantified by densitometry using ImageJ and normalized to GAPDH. The subsequent sentences have been retained because they provide the analytically important normalization details: target-protein expression was normalized to the corresponding GAPDH band intensity from the same lane, and the resulting normalized values were expressed relative to the LPS-only group, which was set to 1.0. This targeted revision eliminates repetition while preserving the complete information required to reproduce the densitometric analysis. It also maintains consistency with the figure legends for Figures 2, 3, and 5, which report densitometric quantification normalized to GAPDH. No experimental procedure, normalization strategy, underlying data, figure, or statistical result has been altered; the change is exclusively editorial and improves the clarity and flow of the Western blotting methodology.

**Location of revision:** Materials and methods - Western blotting subsection.

**Revised passage:**

*“Chemiluminescent detection was performed using SuperSignal™ ULTRA substrate (Thermo Fisher Scientific), and signals were captured on Kodak Biomax XAR film (GE Healthcare, Mississauga, ON, Canada). Band intensities were quantified by densitometry using ImageJ (NIH, Bethesda, MD, USA) and normalized to GAPDH (catalogue number, ab9482; mouse monoclonal; clone number, mAbcam 9484; Abcam, validated for WB by manufacturer). Target protein expression was normalized to the corresponding GAPDH band intensity from the same lane. The normalized values were then expressed relative to the LPS-only group, which was set to 1.0. All immunoblots were performed in triplicate.”*

**Reviewer 1, Comment 4**

***“If not already included within the supplementary material, providing the representative flow cytometry gating strategy would further improve transparency.”***

**Authors’ response**

We thank the reviewer for this helpful suggestion, which we have adopted in full. A representative flow cytometry gating strategy has now been prepared and added to the manuscript as a new Supporting Information figure, **S1 Fig**. The figure shows the complete, sequential gating applied to every sample: (A) exclusion of debris and selection of viable cells on a forward- versus side-scatter plot (FSC-A vs SSC-A); (B) discrimination of single cells and exclusion of doublets (FSC-A vs FSC-H); and representative fluorescence-intensity histograms for (C) RANK (PE) and (D) RANKL (FITC), each displayed against the isotype/negative control with the positive-gating threshold indicated.

Flow cytometry was used in this study for a single purpose — the measurement of RANK and RANKL surface expression reported in Fig 6; the mitochondrial read-outs were obtained by fluorescence microscopy (Rhodamine 123) and spectrophotometry (Janus Green B), not by flow cytometry. The single gating strategy in **S1 Fig** therefore documents the entirety of the flow-cytometric analysis in the paper.

**Where the referral has been inserted.** To make the new Supporting Information item easy to locate for integration, the cross-references and the Supporting Information caption have been added to the revised manuscript as track change and is shown below in **bold blue**. Three additions were made, shown below.

**Additions to the manuscript (shown in bold blue here, and as Track Changes in the file)**

**1. Methods — “Flow cytometric analysis of RANK and RANKL surface expression”**

… analyzed using FlowJo software (Tree Star Inc., Ashland, OR, USA). **The complete gating strategy — sequential exclusion of debris (FSC-A vs SSC-A), single-cell discrimination (FSC-A vs FSC-H), and the RANK- and RANKL-positive thresholds set against isotype/negative controls — is provided in S1 Fig.** All experiments were performed in triplicate.

*Inserted after the acquisition/analysis sentence, before the closing sentence of the paragraph.*

**2. Results — Fig 6 legend**

… Shaded histograms represent the isotype control; open histograms represent the specific antibody signal. **The representative gating strategy applied to all samples is shown in S1 Fig.** Quantitative analysis of the percentage of RANKL-positive events (E) …

*Inserted within the Fig 6 legend, immediately after the description of the histogram overlays.*

**3. New Supporting Information section (added after the Acknowledgement)**

**Supporting Information** **S1 Fig. Flow cytometry gating strategy for RANK and RANKL surface staining.** Sequential gating (debris exclusion by FSC-A/SSC-A; single-cell selection by FSC-A/FSC-H) and representative RANK (PE) and RANKL (FITC) fluorescence-intensity histograms relative to isotype/negative controls, corresponding to the analyses in Fig 6.

*Provides the required PLOS ONE Supporting Information caption; the heading and label are bold, the descriptive caption in blue.*

**Note on labelling**

The item has been cited and captioned throughout as **S1 Fig**, following the PLOS ONE Supporting Information convention. The accompanying Supporting Information file is currently headed “Supplementary Figure S1 / Fig. S1”; we suggest harmonising that header to “S1 Fig” so that the file and the in-text citations match exactly. No other change to the figure is required.

**Effect on the manuscript**

The representative gating strategy is now available to readers, and the flow-cytometric analysis underlying Fig 6 is fully transparent. We are grateful to the reviewer for a suggestion that strengthens the reproducibility of the work.

**Reviewer - 3**

**Reviewer 3, Comment 1**

***“I'm still noticing several typographical errors. Authors: please do a careful line-by-line review, as I may not be able to notice all the errors. Please spend a lot of time going through the manuscript carefully. Publications with several typos can hurt the reader's confidence in the study's results.”***

**Authors’ response**

We thank the Reviewer for highlighting this important point, and we fully agree that typographical, grammatical, formatting, and notation errors can detract from the clarity of a manuscript and, more importantly, may inadvertently undermine confidence in the presentation of the underlying scientific work. In response, we undertook a further meticulous, line-by-line and manuscript-wide proofing of the complete revised manuscript, extending beyond the specific errors identified by the Reviewer. This audit encompassed the Abstract, Introduction, Materials and Methods, Results, figure legends, Discussion, tables, Supporting Information cross-references, Conclusions, and all accompanying explanatory text. Particular attention was given to duplicated or inadvertently retained text arising from successive rounds of tracked revision; punctuation and spacing; grammatical agreement; capitalization; figure and panel citations; consistency of terminology; statistical notation; scientific symbols; Greek characters; units; and the appropriate use of subscripts and superscripts in biochemical and mathematical expressions. Where an error was identified, the original wording has deliberately been retained in **strikethrough** and the corrected form inserted immediately thereafter in **green font**, so that every editorial intervention remains fully transparent and readily auditable. Importantly, this exercise was restricted to editorial, typographical, grammatical, formatting, and notation corrections: **no experimental result, numerical value, scientific interpretation, figure, table, citation, reference, or reference numbering has been added, removed, reordered, or substantively modified**. We have also prepared the detailed correction matrix below, which maps each identified issue to its manuscript location, reproduces the original and corrected forms, classifies the nature of the correction, and records its effect on the scientific content and references. We appreciate the Reviewer’s vigilance in drawing our attention to this matter and believe that this additional quality-control exercise has substantially improved the precision, consistency, and readability of the manuscript while preserving the scientific record exactly as reported.

**Manuscript-wide typographical, punctuation, cross-reference, and scientific-notation audit**

**Additional authors’ response linked to Reviewer 1, Comment 3.** Beyond removing the duplicated densitometry sentence requested by the Reviewer, we undertook a line-by-line proofread of the complete manuscript up to, but not including, the References section. The audit was intentionally conservative: only typographical, grammatical, punctuation, spacing, figure-cross-reference, capitalization, and scientific-notation defects were corrected. No scientific result, numerical value, experimental design element, interpretive conclusion, table content, figure content, or reference was added, removed, renumbered, reordered, or edited. Existing tracked changes were retained in full. Every new correction in the manuscript is displayed using the same visual convention: the text as found is retained in green strikethrough, immediately followed by the corrected form in green.

| **Audit scope** | **Corrections logged** | **Reference changes** | **Existing tracked changes** | **Notation QA** |
| --- | --- | --- | --- | --- |
| Manuscript text before References | 57 | 0 - untouched | Retained | Sub/superscript and pathway notation checked |

**Legend for the correction matrix:** ~~text as found~~ → corrected text. The “Reference impact” column records the explicit check that no bibliographic reference or citation number was altered by that correction.

**Audit profile by error class**

| **Error class** | **Number corrected** |
| --- | --- |
| Spacing | 14 |
| Punctuation | 13 |
| Scientific notation | 6 |
| Capitalization | 4 |
| Pathway notation | 4 |
| Figure cross-reference | 2 |
| Grammar | 2 |
| Statistical notation | 2 |
| Bracket/punctuation | 1 |
| Citation spacing | 1 |
| Dash/hyphenation | 1 |
| Grammar/article | 1 |
| Grammar/preposition | 1 |
| Hyphenation | 1 |
| Punctuation/citation spacing | 1 |
| Punctuation/spacing | 1 |
| Terminology/compound word | 1 |
| Typographical artefact | 1 |

**Detailed correction matrix**

| **QC ID** | **Manuscript location** | **Error class** | **Text as found** | **Corrected form** | **Why the correction was necessary** | **Reference impact** |
| --- | --- | --- | --- | --- | --- | --- |
| T01 | Abstract, p. 2 | Typographical artefact | ~~4mesenchymal~~ | mesenchymal | Removes an extraneous numeral and duplicated spacing in the Abstract. | None. References/citation numbering unchanged. |
| T02 | Abstract, p. 2 | Spacing | ~~anti-catabolic effects~~ | anti-catabolic effects | Removes duplicated spacing. | None. References/citation numbering unchanged. |
| T03 | Introduction, p. 4 | Grammar/article | ~~In the absence of bona fide pathway-specific candidate~~ | In the absence of a bona fide pathway-specific candidate | Restores the missing indefinite article. | None. References/citation numbering unchanged. |
| T04 | Introduction, p. 6 | Punctuation | ~~mTOR signaling can directly~~ | mTOR signaling, can directly | Closes the parenthetical concessive phrase correctly. | None. References/citation numbering unchanged. |
| T05 | Introduction, p. 6 | Hyphenation | ~~PAR-2 driven inflammatory processes~~ | PAR-2-driven inflammatory processes | Hyphenates the compound modifier. | None. References/citation numbering unchanged. |
| T06 | Materials and methods - Cell culture, pp. 7-8 | Scientific notation | ~~5% CO2~~ | 5% CO₂ | Uses the chemically correct subscript for carbon dioxide. | None. References/citation numbering unchanged. |
| T07 | Materials and methods - Cell culture, pp. 7-8 | Scientific notation | ~~2 × 106 cells~~ | 2 × 10⁶ cells | Restores the intended power-of-ten notation. | None. References/citation numbering unchanged. |
| T08 | Materials and methods - Inflammatory model, p. 9 | Scientific notation | ~~5% CO2~~ | 5% CO₂ | Uses the chemically correct subscript for carbon dioxide. | None. References/citation numbering unchanged. |
| T09 | Materials and methods - Western blotting, pp. 10-11 | Terminology/compound word | ~~electro transferred~~ | electrotransferred | Corrects the standard term for electrophoretic transfer. | None. References/citation numbering unchanged. |
| T10 | Materials and methods - Western blotting, pp. 10-11 | Punctuation/spacing | ~~manufacturer) , rabbit~~ | manufacturer), rabbit | Removes the space before the comma. | None. References/citation numbering unchanged. |
| T11 | Materials and methods - Western blotting, pp. 10-11 | Bracket/punctuation | ~~manufacturer) ], rabbit~~ | manufacturer), rabbit | Removes an unmatched closing bracket and restores the separator. | None. References/citation numbering unchanged. |
| T12 | Materials and methods - Western blotting, pp. 10-11 | Punctuation | ~~manufacturer) rabbit anti-human ERK1/2~~ | manufacturer), rabbit anti-human ERK1/2 | Adds the missing comma between antibody entries. | None. References/citation numbering unchanged. |
| T13 | Materials and methods - Western blotting, pp. 10-11 | Capitalization | ~~Catalogue number, ab184699~~ | catalogue number, ab184699 | Harmonizes catalogue-number capitalization within the same sentence. | None. References/citation numbering unchanged. |
| T14 | Materials and methods - Western blotting, pp. 10-11 | Spacing | ~~Thr202/Tyr204)(catalogue~~ | Thr202/Tyr204) (catalogue | Adds the missing space between parenthetical elements. | None. References/citation numbering unchanged. |
| T15 | Materials and methods - Western blotting, pp. 10-11 | Capitalization | ~~polyclonal; abcam~~ | polyclonal; Abcam | Corrects the manufacturer name capitalization. | None. References/citation numbering unchanged. |
| T16 | Materials and methods - Western blotting, pp. 10-11 | Capitalization | ~~Catalogue number, ab9482~~ | catalogue number, ab9482 | Harmonizes catalogue-number capitalization. | None. References/citation numbering unchanged. |
| T17 | Materials and methods - qPCR, pp. 11-12 | Scientific notation | ~~2−ΔΔCT~~ | 2^⁻ΔΔCt^ | Corrects presentation of the comparative Ct expression notation using a superscript minus and conventional Ct typography. | None. References/citation numbering unchanged. |
| T18 | Materials and methods - Flow cytometry, pp. 12-13 | Scientific notation | ~~1 × 106 cells/mL~~ | 1 × 10⁶ cells/mL | Restores the intended power-of-ten notation. | None. References/citation numbering unchanged. |
| T19 | Materials and methods - Flow cytometry, pp. 12-13 | Spacing | ~~manufacturer) followed~~ | manufacturer) followed | Removes duplicated spacing. | None. References/citation numbering unchanged. |
| T20 | Results - Chondrogenic differentiation, pp. 15-16 | Punctuation | ~~morphological level, the cells~~ | morphological level: the cells | Repairs a comma splice by introducing a colon. | None. References/citation numbering unchanged. |
| T21 | Results - Cytotoxicity profiling, pp. 16-17 | Figure cross-reference | ~~Fig. 1E), validating~~ | Fig. 1F), validating | Corrects the LPS cytotoxicity panel reference to match the figure legend. | None. References/citation numbering unchanged. |
| T22 | Results - Cytotoxicity profiling, pp. 16-17 | Figure cross-reference | ~~20 µM (Fig. 1F).~~ | 20 µM (Fig. 1G). | Corrects the EGCG cytotoxicity panel reference to match the figure legend. | None. References/citation numbering unchanged. |
| T23 | Figure 2 legend, pp. 17-18 | Statistical notation | ~~***< 0.001~~ | ***p < 0.001 | Restores the missing p in the significance key. | None. References/citation numbering unchanged. |
| T24 | Figure 3 legend, pp. 18-19 | Capitalization | ~~supplementary Data~~ | Supplementary Data | Harmonizes Supporting/Supplementary Data capitalization. | None. References/citation numbering unchanged. |
| T25 | Figure 4 legend, p. 20 | Punctuation | ~~(fold change)..~~ | (fold change). | Removes duplicated terminal punctuation. | None. References/citation numbering unchanged. |
| T26 | Results - SOX4/ADAMTS-5, p. 22 | Punctuation | ~~concentrations).).~~ | concentrations). | Removes duplicated closing punctuation. | None. References/citation numbering unchanged. |
| T27 | Results - SOX4/ADAMTS-5, p. 22 | Statistical notation | ~~P < 0.01 and P < 0.001~~ | p < 0.01 and p < 0.001 | Harmonizes p-value notation with the rest of the manuscript. | None. References/citation numbering unchanged. |
| T28 | Results - siRNA analysis, p. 26 | Punctuation | ~~(Fig. 8C, )~~ | (Fig. 8C) | Removes the stray comma and space from the figure citation. | None. References/citation numbering unchanged. |
| T29 | Results - siRNA analysis, p. 26 | Grammar/preposition | ~~contributor of cytokine production~~ | contributor to cytokine production | Corrects the governing preposition. | None. References/citation numbering unchanged. |
| T30 | Discussion - PAR-2 mechanistic interpretation, pp. 32-33 | Punctuation | ~~experiments (Fig. 8), PAR-2 knockdown~~ | experiments (Fig. 8): PAR-2 knockdown | Repairs a comma splice and introduces the explanatory clause correctly. | None. References/citation numbering unchanged. |
| T31 | Discussion - PAR-2-independent pathways, p. 34 | Grammar | ~~and is being consistent~~ | and is consistent | Removes the erroneous progressive construction. | None. References/citation numbering unchanged. |
| T32 | Discussion - PAR-2-independent pathways, p. 34 | Punctuation | ~~Akhtar and Haqqi, showed~~ | Akhtar and Haqqi showed | Removes the incorrect comma between subject and verb. | None. References/citation numbering unchanged. |
| T33 | Discussion - PAR-2-independent pathways, p. 34 | Spacing | ~~panel through~~ | panel through | Removes duplicated spacing. | None. References/citation numbering unchanged. |
| T34 | Discussion - PAR-2-independent pathways, p. 34 | Spacing | ~~in primary~~ | in primary | Removes duplicated spacing. | None. References/citation numbering unchanged. |
| T35 | Discussion - PAR-2-independent pathways, pp. 35-36 | Spacing | ~~module [35, 37]~~ | module [35, 37] | Removes duplicated spacing before the citation. | None. References/citation numbering unchanged. |
| T36 | Discussion - PAR-2-independent pathways, pp. 35-36 | Spacing | ~~cytoprotective Nrf2~~ | cytoprotective Nrf2 | Removes duplicated spacing. | None. References/citation numbering unchanged. |
| T37 | Discussion - PAR-2-independent pathways, pp. 35-36 | Spacing | ~~ML385 [38]~~ | ML385 [38] | Removes duplicated spacing before the citation. | None. References/citation numbering unchanged. |
| T38 | Discussion - PAR-2-independent pathways, pp. 35-36 | Citation spacing | ~~p62[39]~~ | p62 [39] | Adds the missing space before the citation without altering the reference. | None. References/citation numbering unchanged. |
| T39 | Discussion - PAR-2-independent pathways, pp. 35-36 | Spacing | ~~axes, EGCG~~ | axes, EGCG | Removes duplicated spacing. | None. References/citation numbering unchanged. |
| T40 | Discussion - PAR-2-independent pathways, pp. 35-36 | Punctuation/citation spacing | ~~PP2A [40] a credible~~ | PP2A [40], a credible | Adds the missing comma after the preserved citation and removes duplicated spacing. | None. References/citation numbering unchanged. |
| T41 | Discussion - PAR-2-independent pathways, pp. 35-36 | Punctuation | ~~We emphasize, that~~ | We emphasize that | Removes an unnecessary comma after the reporting verb. | None. References/citation numbering unchanged. |
| T42 | Discussion - PAR-2-independent pathways, pp. 35-36 | Grammar | ~~several of these effects from single-pathway studies~~ | several of these effects derive from single-pathway studies | Restores the missing finite verb. | None. References/citation numbering unchanged. |
| T43 | Discussion - PAR-2-independent pathways, pp. 35-36 | Spacing | ~~redox-active structure~~ | redox-active structure | Removes duplicated spacing. | None. References/citation numbering unchanged. |
| T44 | Discussion - PAR-2-independent pathways, pp. 35-36 | Spacing | ~~provides a plausible~~ | provides a plausible | Removes duplicated spacing. | None. References/citation numbering unchanged. |
| T45 | Discussion - PAR-2-independent pathways, pp. 35-36 | Punctuation | ~~definitive accounting. .~~ | definitive accounting. | Removes duplicated terminal punctuation. | None. References/citation numbering unchanged. |
| T46 | Discussion - Mitochondrial integrity, pp. 40-41 | Scientific notation | ~~F1F0-ATP synthase~~ | F₁F₀-ATP synthase | Uses conventional subscripts for the F1 and F0 ATP-synthase sectors. | None. References/citation numbering unchanged. |
| T47 | Discussion - Pharmacokinetics, pp. 45-46 | Punctuation | ~~efficiency, the landmark human pharmacokinetic study~~ | efficiency; the landmark human pharmacokinetic study | Repairs a comma splice between independent clauses. | None. References/citation numbering unchanged. |
| T48 | Discussion - Pharmacokinetics, pp. 45-46 | Punctuation | ~~explicitly acknowledged, that~~ | explicitly acknowledged that | Removes an unnecessary comma before the complement clause. | None. References/citation numbering unchanged. |
| T49 | Discussion - Pharmacokinetics, pp. 45-46 | Spacing | ~~5 to10 µM~~ | 5 to 10 µM | Restores the missing space in the concentration range. | None. References/citation numbering unchanged. |
| T50 | Discussion - Joint pharmacokinetics, pp. 46-47 | Punctuation | ~~including cationic PLGA/Eudragit RL are now established~~ | including cationic PLGA/Eudragit RL, are now established | Closes the parenthetical example phrase with a comma. | None. References/citation numbering unchanged. |
| T51 | Discussion - Human-tissue translational context, pp. 52-53 | Spacing | ~~mRNA in genome-wide~~ | mRNA in genome-wide | Removes duplicated spacing. | None. References/citation numbering unchanged. |
| T52 | Discussion - Human-tissue translational context, pp. 52-53 | Pathway notation | ~~miR-140-3p ADAMTS5~~ | miR-140-3p → ADAMTS5 | Restores the directional pathway arrow. | None. References/citation numbering unchanged. |
| T53 | Discussion - Human-tissue translational context, pp. 52-53 | Pathway notation | ~~SOX4ADAMTS-5~~ | SOX4 → ADAMTS-5 | Restores the directional pathway arrow. | None. References/citation numbering unchanged. |
| T54 | Discussion - Human-tissue translational context, pp. 52-53 | Spacing | ~~architecture,one transcriptional~~ | architecture, one transcriptional | Restores the missing space after the comma. | None. References/citation numbering unchanged. |
| T55 | Discussion - Human patient translational context, p. 53 | Pathway notation | ~~PAR-2 ERK1/2 TNF-α/SOX4/ADAMTS-5~~ | PAR-2 → ERK1/2 → TNF-α/SOX4/ADAMTS-5 | Restores the directional arrows in the signalling cascade. | None. References/citation numbering unchanged. |
| T56 | Limitations, pp. 65-66 | Pathway notation | ~~PAR-2ERK1/2TNF-α/SOX4/ADAMTS-5~~ | PAR-2 → ERK1/2 → TNF-α/SOX4/ADAMTS-5 | Restores missing separators/arrows in the pathway notation. | None. References/citation numbering unchanged. |
| T57 | Limitations, pp. 65-66 | Dash/hyphenation | ~~PAR-2 ERK1/2 axis~~ | PAR-2–ERK1/2 axis | Restores the en dash linking the receptor and kinase axis. | None. References/citation numbering unchanged. |

**Verification statement.** After implementing the corrections, we rechecked the manuscript for punctuation duplication, spacing artefacts, broken pathway arrows, figure-panel references, statistical-significance notation, antibody-list punctuation, and common scientific typography. The References section was deliberately excluded from editorial correction and remains exactly as supplied. The tracked-change history already present in the manuscript was not accepted, rejected, or removed.

**Reviewer 3, Comment 2**

***“Line 6 of the abstract starts with the number 4. Please remove the number.”***

**Authors’ response**

We thank the Reviewer for identifying this typographical artefact in the Abstract. **The correction has been made exactly as requested.** The extraneous numeral “4”, which appeared at the beginning of the phrase describing the cellular source, has been removed so that the sentence now reads continuously and correctly as “human chondrocytes derived from bone marrow mesenchymal stem cells.” **This correction is located in the Abstract on page 2 of the revised manuscript.** For complete transparency, the erroneous numeral has been retained as struck-through text in the tracked manuscript and the corrected wording is shown immediately thereafter in green, consistent with the manuscript-wide correction convention adopted in response to Reviewer 3, Comment 1. The corrected passage has also been rechecked in its surrounding sentence to ensure that removal of the numeral does not introduce any spacing, grammatical, or line-flow artefact. This is a purely typographical correction and does not alter the scientific meaning, experimental description, data, interpretation, citation structure, or reference numbering.

**Location of revision:** Abstract, page 2, sentence describing the BM-MSC-derived chondrocytes.

**Correction implemented:** ~~4~~ **→ removed**

**Corrected Abstract wording: “…human chondrocytes derived from bone marrow mesenchymal stem cells.”**

**Effect on the manuscript**

The stray numeral has been eliminated from the Abstract, restoring the intended wording and uninterrupted sentence flow. No scientific content or reference has been changed.

**Reviewer 3, Comment 3**

*“In the Methods section, at line 4 and line 12 of the subsection ‘Cell culture and chondrogenic differentiation’, the ‘2’ in CO2 should be a subscript, not a superscript. The last sentence of that subsection should read ‘… validate the chondrocytic phenotype following our previously established …’ (rather than ‘follow’).”*

**Authors’ response**

We thank the reviewer for these careful corrections, both of which have been made. The subscript formatting has been applied to the ‘2’ in CO_2_ at both occurrences in the ‘Cell culture and chondrogenic differentiation’ subsection — the mesenchymal stem-cell culture condition (line 4) and the chondrogenic-differentiation condition (line 12) — so that each now reads correctly as CO_2_. In the final sentence of the subsection, ‘follow’ has been changed to ‘following’ as requested.

For ease of integration, the corrected text is shown below in **bold blue and as tracked change** in the revised manuscript. The three corrections are set out below. These are typographic and grammatical only; no reference, citation, or table was affected.

**Corrections made (shown in bold blue in the file)**

**1. Line 4 — mesenchymal stem-cell culture condition**

**Before:** … at 37 °C in a humidified 5% CO^2^ atmosphere …

**After:** … at 37 °C in a humidified **5% CO_2_** atmosphere …

*The ‘2’ changed from superscript to subscript.*

**2. Line 12 — chondrogenic-differentiation condition**

**Before:** … Cultures were maintained at 37 °C with 5% CO^2^; fresh medium …

**After:** … Cultures were maintained at 37 °C with 5% **CO_2_**; fresh medium …

*The ‘2’ changed from superscript to subscript.*

**3. Last sentence of the subsection**

**Before:** … procedures used here to validate the chondrocytic phenotype ~~follow~~ our previously established and published protocols [17–19].

**After:** … procedures used here to validate the chondrocytic phenotype **following** our previously established and published protocols [17–19].

*‘follow’ changed to ‘following’; citation [17–19] retained unchanged.*

**Effect on the manuscript**

The two CO_2_ instances are now correctly subscripted, and the final sentence reads as the reviewer specified. We are grateful for the close reading.

**Reviewer 3, Comment 4**

***“Please make sure to be consistent with the journal’s preference for how to format phrases such as in vivo and in vitro, as sometimes I see them italicised and sometimes not.”***

**Authors’ response**

We thank the reviewer for noting this inconsistency, which we have corrected. We have adopted a single, uniform convention and now set the Latin experimental-context terms *in vitro*, *in vivo*, *ex vivo*, *in situ* and *in silico* in italic throughout the running text of the manuscript. Every previously roman occurrence has been converted to italic, so that these phrases are now formatted identically wherever they appear in the main text. The one term already set in italic (*in silico*) was retained.

**How the change is shown.** For ease of integration, each harmonised instance is marked as tracked changes in the revised manuscript (for example, ***in vitro***); on final integration these are simply set to plain italic. In total, 41 instances across the running text were standardised, as summarised below.

| **Term** | **Change applied** | **Body instances** | **Representative context (as now set)** |
| --- | --- | --- | --- |
| *in vitro* | roman → italic | 18 | “… procedures … were performed exclusively *in vitro* …” |
| *in vivo* | roman → italic | 19 | “*In vivo*, intra-articular EGCG ameliorates cartilage degradation …” |
| *ex vivo* | roman → italic | 3 | “… Animal *in vivo*, human *ex vivo*, and human patient studies …” |
| *in situ* | roman → italic | 1 | “… are established *in situ* over years of … stress …” |
| *in silico* | already italic (retained) | — | Left unchanged; already set in italic. |

**Scope.** The harmonisation covers the running text and the figure/table captions. Within the body of the evidence table (Table 4) and in the reference list, the styling of these terms follows the journal’s table and citation conventions and the wording of the cited article titles, respectively, which we have not overridden manually.

**Verification at proofing**

Because the precise typographic treatment of these Latin terms is ultimately governed by the journal’s house style, we will confirm and finalise their formatting in close consultation with the editorial office during galley proofing, to ensure that the presentation of *in vitro*, *in vivo* and related terms is fully consistent with PLOS ONE conventions throughout the published article.

**Reviewer 3 — Comments 5, 6 and 7 (addressed together)**

Comments 5, 6 and 7 concern a single point — whether the replicates in this study are *technical* or *biological* — and we therefore address them together.

**Comment 5.** *In the Western blotting subsection of the Methods, please be clear in the last sentence if “replicate” refers to technical or biological. In the same subsection, the 4th-to-last sentence ending, there is a space between the period and the last word.*

**Comment 6.** *I see “in triplicate” in other subsections… is this technical or biological? Please specify.*

**Comment 7.** *In the statistics subsection of the methods, I find that the following paragraph to be completely unnecessary. People who perform such experiments regularly already know this information. It’s redundant. I think the authors should just simply state whether the replicates were technical or biological… [the two-sentence n = 3 paragraph is then quoted]*

**Authors’ response**

We are glad to make the basis of replication explicit throughout the Methods. In every assay reported here, **n = 3 denotes three independent biological replicates**, fully separate chondrocyte cultures established, differentiated, treated, and assayed on different occasions. Technical replication, where used, is separately and unambiguously identified (for example, the qPCR reactions, which the Methods already describe as being run in *technical* replicates). All manuscript changes below are marked in **bold** (insertions) and **~~strikethrough~~** (deletions) for ease of integration.

**Comment 5 — Western blotting subsection**

The closing sentence of the subsection now specifies the biological basis of the replicate and reads: “All immunoblots were performed in triplicate **(three independent biological replicates)**.” Separately, the spacing slip the reviewer identifies in the fourth-to-last sentence — “… validated for WB by manufacturer).” is **already corrected** to “… manufacturer).” in the current file (visible as a tracked change), and we have re-read the subsection to confirm that no comparable spacing errors remain.

**Comment 6 — “in triplicate” in other subsections**

We have specified the nature of every remaining instance of “in triplicate” in the Methods. Both further occurrences — “All experiments were performed in triplicate” and, in the Janus Green B assay, “All conditions were performed in triplicate” — now carry the same explicit clarification, **(three independent biological replicates)**. The Methods therefore state the biological basis of replication consistently wherever triplication is mentioned.

**Comment 7 — the n = 3 paragraph in the Statistical analysis section**

We appreciate the reviewer’s preference for concision. We note, for the record, that the paragraph identified here was itself introduced **during the first round of revision, in direct response to Reviewer #1’s Comment 2**, which questioned whether n = 3 biological replicates was sufficient and asked us to justify the sample size and the unit of statistical inference. That paragraph was our response to Reviewer #1, which is the reason the material is present in the current version.

In response to the present comment, we have removed the explanatory justification and the accompanying robustness sentence, retaining only the single plain statement that the reviewer asks for — that the replicates are biological. The passage now reads:

“For each assay, n = 3 denotes three independent biological replicates, defined as fully separate chondrocyte cultures established, differentiated, treated, and assayed on different occasions, rather than technical replicates of a single culture [31–33].”

This condensation removes the redundancy the reviewer identifies and, at the same time, directly answers Comments 5 and 6. The three supporting references — Naegle *et al.*, Lazic *et al.*, and Lord *et al.*, each of which specifically concerns biological replication in cell-culture research — are retained on this single sentence, so no reference is orphaned and the reference list is unchanged.

| **No.** | **Location** | **Change made** |
| --- | --- | --- |
| 5 | Methods — Western blotting (final sentence; 4th-to-last sentence) | “in triplicate” specified as **three independent biological replicates**; the space between “manufacturer)” and the period is already corrected in the file (your tracked change). |
| 6 | Methods — inflammatory-model subsection; Janus Green B assay | both remaining “in triplicate” instances specified as **three independent biological replicates**. |
| 7 | Methods — Statistical analysis | verbose paragraph condensed to a single biological-replicate statement; explanatory justification and robustness sentence deleted; references [31–33] retained. |

**Note on the citation fields**

The two adjacent EndNote fields, [31, 32] and [33], will re-merge to the range **[31–33]** automatically at the next field update; no reference has been added or removed. We are grateful to the reviewer, whose comments have made the description of replication in the Methods both explicit and appropriately concise.

**Reviewer 3, Comment 8**

**Figure 1:**

– *The sizes of the boxes for each panel is inconsistent.*

– *Panel B box line seems thicker than the box for Panel A.*

– *The letter E seems to be a smaller size than the other letters.*

– *Letter G for panel G is on the left but for Panel F, the letter F is on the right? Also, the y-axis for Panel G is part of Panel F. Please make this look neat.*

**Authors’ response**

We have examined Figure 1 side by side in two forms: the composite that appears in the review-build PDF, and the full-resolution TIFF that we uploaded to the submission system. We would ask the Reviewer to weigh an important distinction that emerges from that comparison. **Part of what is described here is a genuine layout matter in the source figure, which we have corrected; part of it, however, is not present in the file we submitted at all, and has been introduced by the down-sampling and panel re-flow that occur when the review PDF is generated from the uploaded artwork.** We deal with the four points in turn and, at the end, make a specific procedural request.

**Points that were genuine, and have been corrected**

**Box sizes.** We have re-set every panel on a single ruled layout grid, so that the two phase-contrast panels (A, B) are identical in size, the three histology panels (C–E) are identical, and the two viability charts (F, G) are matched. The inconsistency the Reviewer noted has been removed.

**Panel B border weight.** The Reviewer is correct: the bounding line of panel B was heavier than that of panel A. A single, uniform border stroke (0.75 pt) has now been applied to every panel, so all boxes match exactly.

**Label “E”.** Also correct: the “E” label was set smaller than its neighbours. Every panel label (A–G) has now been standardised to one typeface, point size and bold weight, and placed in the identical upper-left position within each panel. We are grateful for the close reading that caught these.

**The point on labels F/G and the Y-axis, an artefact of the compiled proof**

On this point we must respectfully, but plainly, note that **the arrangement the Reviewer describes is not the arrangement in the figure we submitted.** In the source TIFF, labels F and G are **both** set at the upper-left of their respective panels, and each viability chart carries its **own** complete Y-axis (“Cell Viability (%)”, 0–100); the Y-axis of panel G is wholly contained within panel G and does not belong to panel F. The appearance the Reviewer reports — “F on the right” and G’s axis “part of” F — is the signature of the panel re-positioning and compression that the submission system applies when it assembles the low-resolution review PDF, and it is not reproducible in the uploaded high-resolution file. To remove any residual ambiguity even under aggressive re-compositing, we have nonetheless widened the spacing between panels F and G in the revised figure.

| **Reviewer’s observation** | **Our assessment** | **Action** |
| --- | --- | --- |
| Panel-box sizes inconsistent | Layout | All panels re-set on a common grid; boxes identical within each row (A–B; C–E; F–G). |
| Panel B border heavier than A | **Correct — present in source** | Uniform 0.75-pt stroke applied to every panel border. |
| Label “E” smaller than the others | **Correct — present in source** | All labels A–G unified in typeface, size, bold weight and position (upper-left). |
| F on the right / G on the left; G’s y-axis “part of” F | **Not present in the source TIFF — compiled-PDF artefact** | Verified: in the source file F and G are both upper-left-labelled, each with its own complete Y-axis. Inter-panel spacing increased; please assess the source TIFF. |

**A procedural request**

Because this is not the first presentational point to turn on the difference between the source artwork and the compiled proof, we would respectfully ask that Figure 1, and, indeed, all figures — be evaluated against the **individually uploaded, full-resolution TIFF files**, which are the figures of record, rather than against the down-sampled composite embedded in the review-build PDF. The review PDF necessarily re-compresses and re-flows each figure, which softens line edges and can displace panels; the source TIFFs do not carry these artefacts. We have re-exported Figure 1 at full publication resolution and are confident that, viewed at source resolution, it now reads cleanly, consistently and neatly. We thank the Reviewer for prompting the genuine improvements above, which we were glad to make.

**Reviewer 3, Comment 9**

***In the text for the “Cytotoxicity profiling of EGCG and LPS” subsection of the Results, you are not referring to the correct panels… please fix.***

**Authors’ response**

The reviewer is correct, and we thank them for catching this. The cause is traceable to an earlier revision: when we inserted the new 28-day post-induction phase-contrast micrograph as **Figure 1B** (in response to the reviewer’s earlier request to show both pre- and post-induction morphology), every subsequent panel was re-lettered by one position. This shifted the two MTT cell-viability panels from their previous positions — LPS viability from panel E to **panel F**, and EGCG viability from panel F to **panel G**. The two figure callouts in the “Cytotoxicity profiling of EGCG and LPS” subsection were inadvertently left pointing to the pre-re-lettering panels (1E and 1F).

We have corrected both callouts so that they now match the re-lettered figure and the Figure 1 legend: the LPS-viability sentence cites **Fig. 1F**, and the EGCG-viability sentence cites **Fig. 1G**. The corrected subsection now reads (panel references shown in blue):

“LPS exhibited no discernible cytotoxicity across the tested concentration range (0–40 µg/mL; **Fig. 1F**), validating its suitability for establishing the pro-inflammatory model. Similarly, EGCG demonstrated no significant reduction in chondrocyte viability at concentrations up to 20 µM (**Fig. 1G**). …”

| **Result callout** | **Previous (incorrect)** | **Corrected** |
| --- | --- | --- |
| MTT viability — LPS (0–40 µg/mL) | Fig. 1E | **Fig. 1F** |
| MTT viability — EGCG (up to 20 µM) | Fig. 1F | **Fig. 1G** |

Both corrections are marked in **green** in the revised manuscript (the superseded references shown struck through, the corrected references in bold). We have additionally re-checked every remaining Figure 1 callout throughout the Results — in the “Validation of chondrogenic differentiation” and adjoining paragraphs — against the re-lettered panel scheme (A, pre-induction; B, post-induction; C, Alcian Blue; D, Toluidine Blue; E, collagen type II; F, LPS viability; G, EGCG viability) and confirm that they are now internally consistent. We are grateful to the reviewer, whose scrutiny has improved the accuracy of the figure–text correspondence.

**Reviewer 3, Comment 10**

*Font sizes are not consistent for Figure 2. Some are not readable. Please fix and make look neater.*

**Authors’ response**

We thank the reviewer, and we have re-typeset Figure 2 so that its typography is uniform and every label is clearly legible. All text throughout the figure is now set in a **single typeface** at **consistent point sizes within each category** — one size for the panel letters, one for the axis titles, one for the tick and condition labels, and one for the statistical annotations — applied identically across the blot (panel A) and the two graphs (panels B and C). The previously small or indistinct labels have been enlarged to a size that reads clearly at publication scale, and the panel spacing and alignment have been tidied so that the figure presents neatly. The specific changes are itemised below.

| **Element** | **Action taken** |
| --- | --- |
| Typeface | A single sans-serif typeface (Arial) is used for every text element in all three panels. |
| Panel letters (A–C) | Set to one uniform size and weight, in the identical upper-left position. |
| Axis titles | Standardised to a single, larger point size across panels B and C for clear legibility. |
| Tick and category (condition) labels | Unified in size and enlarged; the concise labels Ctrl, LPS, LPS + EGCG-5 µM and LPS + EGCG-10 µM sit directly on the axes and blot lanes. |
| Blot annotations | Row identifiers (PAR-2, GAPDH) and molecular weights (44 kDa, 37 kDa) set at one consistent size. |
| Significance markers | “ns”, **, *** rendered at a single uniform size across both graphs. |

The protein-first panel arrangement (panel A, immunoblot; panel B, densitometric protein quantification; panel C, transcript-level quantification) has been retained, for the reasons set out in our earlier response. We would additionally note, as previously, that the review-build PDF down-samples and re-compresses each figure, which can soften small text; we would therefore ask that legibility be assessed against the **individually uploaded, full-resolution TIFF**, which we have re-exported at full publication resolution and against which every label is sharp and readable. We are grateful to the reviewer, whose comment has improved the clarity and presentation of the figure.

**Reviewer 3, Comment 11**

***Figure 2 legend shows: ***< 0.001 . It’s missing the letter p.***

**Authors’ response**

The reviewer is correct, and we have corrected this. The third significance tier in the Figure 2 legend had been typeset without its leading italic *p*, appearing as “***< 0.001” instead of “****p* < 0.001”. The *p* has been restored, so the significance key in the Figure 2 legend now reads:

“**p* < 0.05, ***p* < 0.01, ****p* < 0.001 versus LPS-only group (one-way ANOVA with Dunnett’s post-hoc test).”

To ensure the same omission does not occur elsewhere, we have additionally re-checked the significance keys in every figure legend and harmonised them to the identical three-tier convention — **p* < 0.05, ***p* < 0.01, ****p* < 0.001 — with the italic *p* and consistent spacing applied throughout. We are grateful to the reviewer for catching this typographical omission.

**Reviewer 3 — Comments 12 and 13 (addressed together)**

Both comments concern the presentation and legibility of the figures, and we address them together.

**Comment 12. *Why is Figure 3 so small compared to other figures? The size ratios are off. Not neat. The image is also not good quality and is blurry.***

**Comment 13. *Figures 2, 3, and 5: the sample labels above the lanes in the blots are too small, hard to read.***

**Authors’ response**

We have improved the figures on the specific points raised, and we set out the changes below. We would also, as in our earlier response, ask that the figures be evaluated against the **individually uploaded, full-resolution TIFF files**, rather than the composite embedded in the review-build PDF: the review PDF necessarily down-samples and re-compresses each figure, which softens fine detail and small text and can affect the apparent relative size of a figure on the page. That said, we have addressed the underlying causes directly so that the figures read cleanly at source.

**Figure 3 — proportion, neatness and quality (Comment 12)**

The reviewer is right that Figure 3 sat awkwardly relative to the other figures. Its six panels had been arranged in a wide, landscape format whose proportions differed from those of the other multi-panel figures; when fitted to the column, this caused the figure to render short and comparatively small, with the panels appearing cramped. We have **re-laid out Figure 3 to a proportion consistent with the other figures**, so that it now occupies a comparable footprint and each panel is appropriately sized and clearly separated. We have additionally **re-exported the figure at full publication resolution (≥300 dpi)**; at this resolution the blot and graph elements are sharp, and the softness the reviewer observed — which is accentuated by the review-PDF build — is removed at source.

**Blot lane labels (Comment 13)**

We have increased the point size of the sample (lane) labels positioned above the blots in Figures 2, 3 and 5 (Ctrl, LPS, LPS + EGCG-5 µM, LPS + EGCG-10 µM), so that they are unambiguously legible. Together with the higher export resolution, these labels now read clearly at print size. The same concise labelling scheme is used across all blot figures for consistency.

| **Figure(s)** | **Point raised** | **Action taken** |
| --- | --- | --- |
| Figure 3 | Renders small; size ratios off; not neat | Re-laid out from a wide (landscape) arrangement to a proportion consistent with the other multi-panel figures, so it occupies a comparable footprint and its panels are appropriately sized. |
| Figure 3 (all figures) | Blurry / poor quality | Re-exported at full publication resolution (≥300 dpi), eliminating softening at source. |
| Figures 2, 3, 5 | Sample (lane) labels above the blots too small | Point size of the lane labels increased so they read clearly at print size. |

With these changes — a proportionally consistent, higher-resolution Figure 3 and enlarged blot labels throughout — we believe the figures are now neat, uniformly sized and fully legible. We are grateful to the reviewer for the scrutiny, and we reiterate our request that final legibility be assessed against the uploaded source TIFF files.

**Reviewer 3, Comment 14**

***The text in the Results section dedicated to figure 9 is absolutely unnecessary! It is so verbose! Please find a way to make your point across concisely!***

**Authors' Response**

We thank the Reviewer for this comment and for the sustained attention they have given to the readability of this manuscript, which has improved it materially across both rounds of review. We have considered the point carefully. We respectfully, but firmly, submit that the Results subsection presenting Fig 9 should be retained in its present form, for a reason that extends beyond its individual merit: **this subsection exists, and has the structure and length it has, because it is the literal implementation of a formal instruction issued in Round 1, and because it is the single place in the manuscript where three further undertakings given to the reviewer panel are honoured with respect to the integrated model.** We document that provenance first, because it is the crux of our response, and then address the question of concision on its own terms.

**1. The subsection is the implemented response to Reviewer #3, Comment 21 of Round 1**

In the previous round, Reviewer #3 raised the following comment:

***Comment 21.*** *Figure 9 should have its own brief section in the Results section before discussing in the discussion section.*

We agreed without reservation. In the originally submitted manuscript Fig 9 was first cited only within the Discussion, so the integrated model was effectively interpreted before it had ever been formally presented. Our response stated: *“we have added a dedicated subsection at the end of the Results that introduces Fig 9 in its own right and walks the reader through the model systematically, ahead of any interpretive discussion.”*

**The passage now identified as unnecessary *is* that walkthrough. It was created for no other purpose. Deleting it, or reducing it to a summary sentence, would return the manuscript to precisely the configuration that Comment 21 identified as defective, and would withdraw a revision that has already been made and entered into the record of this review.**

**2. The five-module architecture, and the prose that traces it, were themselves requested**

The elaboration of Fig 9 was not an authorial embellishment. Our Round 1 response records the reason for it explicitly: *“In line with the Reviewer’s broader point that the schematic should make the marker-to-pathway logic transparent, the revised Fig 9 has been elaborated into a five-module architecture, and the new Results subsection traces each module in technical detail.”*

That broader point was not incidental to the review; it was its dominant theme. In Comments 11, 12, 15, 17 and 18 the Reviewer required, in their own words, that *“Figures must be able to stand on their own”*, and every figure in the manuscript was rebuilt to that standard: numeric and alphabetic index keys removed, treatment conditions written out in full on every axis, lane and plot, and separate legend keys eliminated so that no panel depends on an external key. Fig 9 is, by a wide margin, the most complex figure in the paper — five modules, some thirty nodes, two divergent cellular trajectories and a two-colour semantic scheme. The Results prose is the apparatus by which that figure is made to stand on its own. Its length is set by the figure’s complexity, and that complexity is itself a product of the same review round.

**3. The passages that read as verbose are the firewall between what was measured and what is merely depicted**

This is the decisive consideration, and we ask that it be weighed carefully. The subsection performs two functions simultaneously: it presents the model, and it states, node by node, whether each element is an endpoint measured in this study or established pathway architecture included for mechanistic completeness. Every measured node is anchored to the specific figure that reports it — PAR-2 (Fig 2); ERK1/2, DUSP6 and the ERK transcripts (Fig 3); TNF-α, IL-1β and MCP-1 (Fig 4); SOX4 and ADAMTS-5 (Fig 5); RANK and RANKL (Fig 6); ΔΨm and mitochondrial mass (Fig 7); and the receptor requirement itself (Fig 8). Every node that was *not* measured is flagged as such: p38, JNK, the direct NF-κB readout, COX-2/PGE2, MEK1/2, MMP-13, aggrecan and collagen II breakdown, ROS accumulation, apoptotic stress, pre-osteoclast commitment and osteoclastogenesis — eleven depicted elements that the text states plainly were not assayed here.

**That accounting is what generates the sentences the Reviewer identifies as surplus. It is not stylistic padding; it is the mechanism that prevents a figure containing numerous unassayed nodes from reading as a claim to experiments the study did not perform. This commitment to rigorous accuracy was stated explicitly in our Round 1 response and, critically, it is the instrument through which three separate undertakings to the other two Reviewers are discharged:**

**(i) Reviewer #2, Comment 4 — mitochondrial claims.** The Reviewer required that “mitoprotective” claims be refined to structural and thermodynamic markers, because no functional bioenergetic assays (Seahorse OCR, ATP quantification) were performed. We accepted this in full and revised the title, Abstract, Results and Discussion accordingly. Module 4 of Fig 9 depicts ROS accumulation and apoptotic stress. The sentence in the Results that renders these “as the established upstream cause and downstream consequence of membrane-potential collapse rather than as parameters measured here” is the only point in the manuscript at which that accepted restriction is enforced for the schematic. Remove it and the figure re-asserts, unqualified, the very claim Reviewer #2 asked us to withdraw.

**(ii) Reviewer #2, Comment 3 — pathway specificity.** The Reviewer required that EGCG be framed as a multifunctional agent for which PAR-2 is “a predominant node rather than an exclusive target”. The closing paragraph of the Fig 9 subsection carries that qualification into the model, identifying PAR-2 as “the predominant, though not exclusive, node through which EGCG exerts its effect”, on the authority of the siRNA data. Without it, a schematic that routes five effector arms through a single receptor reads unavoidably as a claim of exclusivity.

**(iii) Reviewer #1, Minor Concern 3 — translational overstatement.** The Reviewer asked that the language be moderated to reflect mechanistic plausibility rather than demonstrated effect. The Fig 9 subsection is written throughout in exactly that register — “supports an association”, “the functional consequence inferred from this protease output rather than an endpoint measured here”. Compression would, in practice, restore the confident declarative phrasing that Round 1 was at pains to remove.

**A shorter Fig 9 subsection is therefore not merely a tidier one. Structurally, it is an over-claiming one.**

**4. Comment 20 of Round 1 fixes the register of this subsection, and a descriptive register is necessarily longer**

In Comment 20 the same Reviewer required that the interpretive paragraph beginning *“Two conclusions emerge from these data”* be removed from the Results and relocated to the Discussion. We implemented this in full, and the Results now close on directly measured data. The consequence for Fig 9 is structural: its Results subsection may not compress the model into interpretive summary, because interpretation now belongs elsewhere by the Reviewer’s own instruction. It must *describe*. Interpretation is brief by nature; systematic description of a five-module schematic is not. Comments 20 and 21, read together, determine both the location and the register of this text, and its length follows from that determination rather than from any preference of ours.

**5. Fig 9 also carries the manuscript’s answer to the sample-size objection**

Reviewer #1, Comment 2 challenged the sufficiency of n = 3. Our response did not rest on replicate count; it rested on convergence: *“robustness in a mechanistic study of this nature derives not from replicate count alone, but from the convergence of independent lines of evidence”*, supported by five methodologically orthogonal techniques. Fig 9 is the formalisation of that argument — five independently measured effector arms, interrogated by qPCR, immunoblotting, ELISA, flow cytometry, fluorescence and histochemical staining, and siRNA loss-of-function, all concordantly reversed by a single upstream intervention whose necessity is established quantitatively. It is the only element of the manuscript in which that convergence is made visible as a whole, and the Discussion opens by invoking it as such (“summarized in Fig. 9”). Diminishing its presentation in the Results correspondingly weakens the manuscript’s reply to Reviewer #1’s second Major Concern.

**6. On concision as such**

We do not dismiss the Reviewer’s point, and we have tested it against the text rather than merely asserting a defence. The subsection runs to 1,202 words — approximately 7.8% of a 15,440-word manuscript — for the figure that synthesises the entire study. It comprises seven paragraphs in a strict, non-redundant scheme: one establishing the apical node and the logic of the model, one for each of the five modules, and one integrating the modules into the two divergent trajectories. No module is treated twice. Critically, the subsection restates *none* of the numerical findings reported earlier in the Results: no means, standard deviations, fold-changes, percentages or p-values are repeated, and every cross-reference is internal, in keeping with our Round 1 undertaking that no new citation be introduced there. The writing is dense, but density is not redundancy. We were unable to identify a sentence whose removal would not either sever a node from the figure that evidences it, or erase a measured-versus-depicted distinction that another Reviewer has required us to preserve.

**7. Precedent within this same review**

We note, with respect, that this is the second occasion on which material created at the instance of one reviewer has been challenged as unnecessary by another. In Comments 22 and 23 of Round 1 the same question was raised of the three Discussion tables, and we set out then the principle we believe governs it: content that is the evidentiary basis of an agreed response to another reviewer is not a discretionary stylistic addition, and cannot be removed without simultaneously re-opening a point that the revision has already closed. That principle applies here with greater force still, because the Fig 9 subsection was created not merely to support a response, but in literal compliance with an explicit instruction to create it.

**Manuscript Changes**

*No changes have been made to the manuscript in response to this comment. The Results subsection presenting Fig 9, the five-module architecture of the figure, and the Fig 9 legend in its Results position are all retained unaltered.*

**An offer, should the Editor take a different view**

We recognise that the Editor may nonetheless wish the section shortened, and we would of course comply. We would propose to do so in the manner least destructive of the commitments set out above: retaining the seven-paragraph structure but reducing each module paragraph to its measured endpoints and their source figures, and replacing the distributed measured-versus-depicted statements with (i) a single explicit blanket sentence and (ii) a compact table mapping each node of Fig 9 to its source figure and to its status as measured or depicted. This would reduce the subsection by approximately half while preserving intact the boundary between data and context on which our responses to Reviewer #1 (Minor Concern 3) and Reviewer #2 (Comments 3 and 4) depend. We would be glad to prepare such a version on the Editor’s instruction; we do not propose it as our preferred course, for the reasons given above.

**Summary**

The Results subsection devoted to Fig 9 was created in direct compliance with Reviewer #3’s Comment 21 of Round 1, which required that the integrated model be presented in its own Results section before being interpreted in the Discussion; its five-module structure and node-by-node treatment follow from the same Reviewer’s requirement that the figure be intelligible without an external key; its descriptive rather than interpretive register is fixed by that Reviewer’s Comment 20; and the specific sentences that give it its length are the mechanism by which the manuscript distinguishes its depicted-but-unmeasured nodes from its measured endpoints, thereby honouring the undertakings given to Reviewer #1 (Minor Concern 3) and Reviewer #2 (Comments 3 and 4). Fig 9 is, further, the only place in which the convergence of five orthogonal readouts on a single receptor — the substance of our answer to Reviewer #1’s objection on sample size — is made visible as a whole. We have therefore retained the subsection unchanged and are grateful to the Reviewer for the opportunity to make its role in the manuscript explicit.

**Reviewer Comments 15, 16 and 17**

***Comment 15.*** *The discussion section is extremely long and verbose! The authors should find a more concise way to convey the information.*

***Comment 16.*** *The tables in the discussion section should just be included as part of supplementary information, and not included physically in the discussion.*

***Comment 17.*** *I find that there are not enough references used in the discussion, especially the limitations section. The whole discussion needs to be dramatically cut down and improved. Too many words! Please find a way to get your point across succinctly!*

**Authors' Response**

We thank the Reviewer for returning to the Discussion, and we have examined all three comments closely against the current text. Our response has three parts. First, we document that the condensation requested in Comments 15 and 17 **has already been carried out in this present revision cycle**, at the request of Reviewer #1, and we give the figures. Second, we set out, with the arithmetic, why Comments 16 and 17 cannot both be implemented: the tables in the Discussion ***are*** its referencing apparatus, and relegating them to Supporting Information would strip 49 citation markers and 18 references — eight of which are cited nowhere else in the manuscript — out of the very section the Reviewer finds under-referenced. Third, we accept the one element of Comment 17 that we consider well founded, namely the thin referencing of the Limitations, and we have addressed it by adding citations only, without adding a single word of new text.

**A. The condensation requested in Comments 15 and 17 has already been performed in this revision**

The Discussion has now been shortened twice, in two consecutive rounds, in response to comments from two different reviewers:

| **Round** | **Prompting comment** | **Action taken** |
| --- | --- | --- |
| Round 1 | Reviewer #1, Minor Concern 1 ("overly verbose in several sections"); Reviewer #3, Comments 22 and 23 | Six thematic subheadings introduced to partition the Discussion into signposted units; the two densest passages (mitochondrial membrane potential / ATP synthesis, and the opening of the PAR-2–independent paragraph) rewritten in more economical phrasing. No content or citation removed. |
| Round 2 (present cycle) | Reviewer #1, Comment 2 ("The Discussion remains relatively lengthy and contains some repetition of the Results. Moderate condensation would improve readability.") | Seven itemised condensations, documented in full in the change log earlier in this same response document. Sentences that restated the Results were excised, the two paragraphs that twice drew the same PAR-2-dependence conclusion were collapsed, and the survey of PAR-2–independent pathways was tightened. Approximately 1,140 words of prose deleted against approximately 240 words of replacement — a net reduction of approximately 900 words. No reference removed; no table altered. |
| Round 2 (present cycle) | Reviewer #3's own Comments 5, 6 and 7 | The n = 3 justification paragraph in the Statistical analysis section — itself introduced in Round 1 at Reviewer #1’s request — reduced to the single plain statement the Reviewer asked for. |

**We would respectfully ask that Comments 15 and 17 be weighed against the condensed Discussion rather than the version that prompted them.** The requested action has been taken, within this same revision cycle, and is fully documented above in our response to Reviewer #1, Comment 2; the edits are present in the revised manuscript as tracked changes, comprising approximately 1,134 words of tracked deletion against approximately 255 words of tracked insertion in the Discussion alone. In its present state the Discussion, including the Limitations, runs to approximately 5,050 words of narrative, organised into seven signposted thematic subsections, and carries 63 citation markers drawn from 50 distinct references — approximately one citation for every 80 words of prose.

**B. Comments 16 and 17 are mutually exclusive as drafted, and this is the crux of our response**

Comment 17 states that the Discussion contains too few references. Comment 16 asks that the Discussion tables be moved out of the Discussion. We must point out, with respect, that **the tables are where a substantial part of the Discussion’s referencing physically resides.** This is not incidental to their design; it is their purpose. Table 2 carries a dedicated final column headed "Key References"; Table 3 carries a dedicated column headed "Ref"; and in Table 4 the first column is headed "Reference", each row being an individual published study set out with its model, dose, duration, markers, findings and our critical assessment of it.

The consequence can be quantified precisely:

| **Where the Discussion’s evidence sits** | **Citation load** |
| --- | --- |
| Discussion narrative (including Limitations), ~5,050 words | 63 citation markers · 50 unique references |
| Tables 2, 3 and 4, ~4,031 words of structured matrix | 49 citation markers · 18 unique references |
| **Combined Discussion apparatus** | **112 citation markers · 58 of the manuscript’s 85 references (68% of the entire bibliography)** |
| *References cited* ***only*** *inside Table 3 and nowhere else in the manuscript* | *References [51]–[58] — eight references* |

**Relocating the three tables to Supporting Information would therefore remove 49 citation markers and 18 references from the body of the Discussion, and would move eight references out of the article altogether, since they are cited nowhere else. Executing Comment 16 would make the deficiency identified in Comment 17 substantially worse, in the same section, in the same revision. We do not believe the Reviewer, on reflection, would wish us to do both, and we are unable to select between them without direction.**

**C. Why the three tables belong in the Discussion**

Beyond the contradiction above, four considerations bear on Comment 16, and we ask that they be weighed together.

**1. Each table is the evidentiary basis of an accepted response to another reviewer.** This was set out in our Round 1 response to Comments 22 and 23, and it remains the governing consideration. Table 2 (PAR-2-independent signalling pathways) is the instrument through which we answered Reviewer #2, Comment 3, which required that EGCG be framed as a multifunctional agent for which PAR-2 is a predominant rather than an exclusive node, and it also underpins our response to Reviewer #2, Comment 4. Table 3 (EGCG content across 36 commercially consumed green teas) is the quantitative basis of our answer to Reviewer #1, Major Concern 5, on whether the tested concentrations are physiologically achievable, and of our answer to this Reviewer’s own Round 1 Comment 6 on concentration selection. Table 4 (animal in vivo, human ex vivo and human patient evidence) is the entire basis of our answer to Reviewer #1, Major Concern 1 — "the study is entirely based on an in vitro model, which limits its biological and clinical relevance" — the single most consequential criticism levelled at this manuscript, and it is invoked explicitly in the Limitations. Relegating these tables to Supporting Information would move the answers to three formally resolved concerns out of the article body and into a separate file.

**2. The tables are signposts, not appendices.** Each row pairs a specific mechanism, tea variety or published study with its citation and, in Table 4, with our critical assessment of it, so that a reader can see at a glance *which* published finding corresponds to *which* element of our data, and how far it can be relied upon. Rendered as prose, those same references collapse into an undifferentiated citation string and that correspondence — which is the informational content — is lost. The tables are, in other words, precisely the device by which the Discussion is kept comprehensive without becoming verbose, which was their stated purpose when they were introduced.

**3. Relocation would lengthen the Discussion, not shorten it.** The tabulated material amounts to approximately 4,031 words held in matrix form (Table 2, 7 rows × 5 columns; Table 3, 37 rows × 9 columns; Table 4, 11 rows × 7 columns). If the tables are moved out and the content is not replaced, the Discussion loses its evidentiary basis. If the content is replaced in narrative prose, the Discussion grows substantially — the precise opposite of what Comments 15 and 17 ask for. A table is, by construction, a device for compressing dense, multi-dimensional comparative information into a form apprehensible at a glance; these tables are not the cause of the Discussion’s length but the principal means by which that length is contained.

**4. Relocation would disrupt the flow of the argument.** The Discussion is signposted into labelled thematic subsections, and each table sits within the subsection whose argument it supports, at its point of first relevance: Table 2 within the discussion of PAR-2-independent mechanisms, Table 3 within "Physiological and pharmacokinetic considerations" and "Selection of the LPS and EGCG concentrations", and Table 4 within "Translational context across animal, human-tissue, and clinical evidence". A reader following the translational argument — including in the Limitations, which cross-references Table 4 directly — would have to leave the article, open a separate file, and return, on three separate occasions. We believe this would make the manuscript a materially more difficult read, not an easier one. We would add that the journal’s formatting requirements do not oblige us to relocate them: tables are permitted in the body of the article.

**D. The Limitations references — the element of Comment 17 we accept, and how we have implemented it**

We accept this part of the comment without reservation. The Limitations was indeed the most thinly referenced portion of the Discussion: 894 words carrying only three citation markers, drawn from four references ([82]–[85]). We have remedied this.

**Critically, we have added citations only — not one new sentence, clause or word.** *To add explanatory text in order to accommodate references would lengthen a section that the same Reviewer objects to as too long in Comments 15 and 17. Every reference below has therefore been attached to a claim that the Limitations already makes, at the end of the existing sentence that makes it. The length of the Limitations is unchanged; only its evidentiary support has increased.*

**References added, and the existing sentences to which they attach:**

| **#** | **Existing sentence (unchanged) to which the citation is appended** | **Reference attached** | **Claim now supported** |
| --- | --- | --- | --- |
| 1 | "…in which endogenous PAR-2 agonists (trypsin, tryptase, matriptase) and mechanical load co-drive pathology." | Jackson et al., Arthritis Rheumatol 2014; Xiang et al., Osteoarthritis Cartilage 2006 | That PAR-2 is operative in the OA joint in vivo and is regulated by the endogenous inflammatory milieu. |
| 2 | "…including the NLRP3 inflammasome, and through integrins such as α5β1 and αvβ3, pathways that LPS stimulation does not reproduce." | Zhang et al., J Adv Res 2024 (NLRP3); Forsyth, Pulai & Loeser, Arthritis Rheum 2002 (α5β1) | That the NLRP3 inflammasome and fibronectin-fragment/α5β1 integrin signalling are independently established catabolic routes in chondrocytes. |
| 3 | "…including PIEZO1/2 channels and integrin-mediated signalling, is not modelled by a soluble chemical stimulus." | Lee et al., Proc Natl Acad Sci USA 2021; Gao et al., Front Cell Dev Biol 2022 | That PIEZO-mediated mechanotransduction is a distinct, inflammation-sensitised pathway in articular chondrocytes. |
| 4 | "…the reciprocal crosstalk among chondrocytes, synoviocytes, and macrophages that shapes the inflammatory response in vivo." | Hamasaki et al., Cartilage 2021 | That macrophage–chondrocyte crosstalk demonstrably alters chondrocyte catabolic output in co-culture. |
| 5 | "Continuous in vitro exposure to a fixed EGCG concentration does not reflect the pharmacokinetic reality of intermittent oral dosing…" | Existing reference [22] (Chow et al., Clin Cancer Res 2003) — re-cited, not new | That human EGCG pharmacokinetics under repeated oral dosing are documented and differ from continuous exposure. No new reference required. |
| 6 | "They are validated and informative markers of mitochondrial integrity, but they are not functional bioenergetic assays…" | Perry et al., Biotechniques 2011 | That cationic potentiometric probes report membrane potential with known interpretive limits and are not substitutes for functional assays — the exact distinction Reviewer #2, Comment 4 required us to observe. |
| 7 | "Future in vivo studies employing destabilization of the medial meniscus or similar models…" | Glasson, Blanchet & Morris, Osteoarthritis Cartilage 2007 | The methodological source of the DMM model named in the sentence. |

The Limitations now carries citations at every substantive claim it advances, while remaining exactly the same length. Nine references are added in total; one further citation ([22]) is a re-use of a reference already in the list.

**References added (full details for the reference list)**

**1.** Jackson MT, Moradi B, Zaki S, Smith MM, McCracken S, Smith SM, et al. Depletion of protease-activated receptor 2 but not protease-activated receptor 1 may confer protection against osteoarthritis in mice through extracartilaginous mechanisms. Arthritis Rheumatol. 2014;66(12):3337-48. doi: 10.1002/art.38876. PMID: 25200274.

**2.** Xiang Y, Masuko-Hongo K, Sekine T, Nakamura H, Yudoh K, Nishioka K, et al. Expression of proteinase-activated receptors (PAR)-2 in articular chondrocytes is modulated by IL-1beta, TNF-alpha and TGF-beta. Osteoarthritis Cartilage. 2006;14(11):1163-73. doi: 10.1016/j.joca.2006.04.015. PMID: 16757188.

**3.** Zhang P, Zhai H, Zhang S, Ma X, Gong A, Xu Z, et al. GDF11 protects against mitochondrial-dysfunction-dependent NLRP3 inflammasome activation to attenuate osteoarthritis. J Adv Res. 2024;73:501-15. doi: 10.1016/j.jare.2024.08.001. PMID: 39103049.

**4.** Forsyth CB, Pulai J, Loeser RF. Fibronectin fragments and blocking antibodies to alpha2beta1 and alpha5beta1 integrins stimulate mitogen-activated protein kinase signaling and increase collagenase 3 (matrix metalloproteinase 13) production by human articular chondrocytes. Arthritis Rheum. 2002;46(9):2368-76. doi: 10.1002/art.10502. PMID: 12355484.

**5.** Lee W, Nims RJ, Savadipour A, Zhang Q, Leddy HA, Liu F, et al. Inflammatory signaling sensitizes Piezo1 mechanotransduction in articular chondrocytes as a pathogenic feed-forward mechanism in osteoarthritis. Proc Natl Acad Sci U S A. 2021;118(13):e2001611118. doi: 10.1073/pnas.2001611118. PMID: 33758095.

**6.** Gao W, Hasan H, Anderson DE, Lee W. The role of mechanically-activated ion channels Piezo1, Piezo2, and TRPV4 in chondrocyte mechanotransduction and mechano-therapeutics for osteoarthritis. Front Cell Dev Biol. 2022;10:885224. doi: 10.3389/fcell.2022.885224. PMID: 35602590.

**7.** Hamasaki M, Terkawi MA, Onodera T, Homan K, Iwasaki N. A novel cartilage fragments stimulation model revealed that macrophage inflammatory response causes an upregulation of catabolic factors of chondrocytes. Cartilage. 2021;12(3):354-61. doi: 10.1177/1947603519828426. PMID: 30704288.

**8.** Perry SW, Norman JP, Barbieri J, Brown EB, Gelbard HA. Mitochondrial membrane potential probes and the proton gradient: a practical usage guide. Biotechniques. 2011;50(2):98-115. doi: 10.2144/000113610. PMID: 21486251.

**9.** Glasson SS, Blanchet TJ, Morris EA. The surgical destabilization of the medial meniscus (DMM) model of osteoarthritis in the 129/SvEv mouse. Osteoarthritis Cartilage. 2007;15(9):1061-9. doi: 10.1016/j.joca.2007.03.006. PMID: 17470400.

**Manuscript Changes**

***Comment 15 / Comment 17 (length):*** *no further condensation has been made. The Discussion was condensed by approximately 900 net words earlier in this same revision cycle, in response to Reviewer #1, Comment 2, and that change is documented in full above.*

***Comment 16 (tables):*** *Tables 2, 3 and 4 are retained in their existing positions within the Discussion, unaltered.*

***Comment 17 (references):*** *nine references have been added to the Limitations, and existing reference [22] re-cited, at seven existing sentences. No sentence, clause or word of text has been added, deleted or reordered anywhere in the Discussion or the Limitations.*

**Our position**

**We have therefore not relocated the tables and have made no further reduction to the Discussion. We would not be able to act on Comment 16 without simultaneously deepening the deficiency identified in Comment 17, without withdrawing the accepted answers to Reviewer #1’s Major Concerns 1 and 5 and Reviewer #2’s Comments 3 and 4 from the body of the article, and without either stripping the Discussion of its evidentiary basis or, if that basis were restored as narrative prose, lengthening the very section that Comments 15 and 17 ask us to shorten. Nor can we reduce the Discussion further without undoing the condensation carried out at Reviewer #1’s request earlier in this same cycle. We hope the Reviewer will accept that these are not matters of authorial preference but constraints created by the review record itself, and we have instead directed our effort to the one element of the three comments that could be acted on without such consequences — the referencing of the Limitations, which is now materially stronger at no cost in length.**

**Summary**

The condensation sought in Comments 15 and 17 has already been carried out in this revision cycle at Reviewer #1’s request: approximately 900 net words were removed from the Discussion across seven documented edits, with no reference removed and no table altered, and a further condensation was made in the Methods at this Reviewer’s own Comments 5–7. Comment 16 cannot be implemented alongside Comment 17, because the three Discussion tables carry 49 of the section’s 112 citation markers and 18 of its references, eight of which appear nowhere else in the manuscript; relocating them would strip the referencing out of the section that Comment 17 finds under-referenced, would move the accepted answers to Reviewer #1’s Major Concerns 1 and 5 and Reviewer #2’s Comments 3 and 4 out of the article body, and — if the content were restored as prose — would lengthen rather than shorten the Discussion. We have therefore retained the tables and made no further cuts. We have, however, acted on the element of Comment 17 we consider well founded: the Limitations, previously supported by four references across 894 words, now carries nine additional references attached to seven existing sentences, added without a single new word of text, so that the section is better evidenced without becoming any longer. We are grateful to the Reviewer, whose comment on the Limitations has materially strengthened that section.
