# Supplementary material for: Epigallocatechin-3-gallate attenuates PAR-2–mediated inflammatory signaling and preserves markers of mitochondrial integrity in osteoarthritic chondrocytes
Source: PLoS One. 2026 Nov 2;21(11):e0357766. doi: 10.1371/journal.pone.0357766 (PMC13726950; doi:10.1371/journal.pone.0357766)
Supplement: Peer review history

# Peer review: Decision Letter 0

**DOI:** 10.1371/journal.pone.0357766.r001

**Authors:** UDAYAN BHATTACHARYA

20 May 2026

Dear Dr. Banerjee,

Thank you for submitting your manuscript to PLOS ONE. After careful consideration, we feel that it has merit but does not fully meet PLOS ONE’s publication criteria as it currently stands. Therefore, we invite you to submit a revised version of the manuscript that addresses the points raised during the review process.

Please submit your revised manuscript by Jul 04 2026 11:59PM. If you will need more time than this to complete your revisions, please reply to this message or contact the journal office at plosone@plos.org. When you're ready to submit your revision, log on to https://www.editorialmanager.com/pone/ and select the 'Submissions Needing Revision' folder to locate your manuscript file.

- A letter that responds to each point raised by the academic editor and reviewer(s). You should upload this letter as a separate file labeled 'Response to Reviewers'.
- A marked-up copy of your manuscript that highlights changes made to the original version. You should upload this as a separate file labeled 'Revised Manuscript with Track Changes'.
- An unmarked version of your revised paper without tracked changes. You should upload this as a separate file labeled 'Manuscript'.

If you would like to make changes to your financial disclosure, please include your updated statement in your cover letter. Guidelines for resubmitting your figure files are available below the reviewer comments at the end of this letter.

If applicable, we recommend that you deposit your laboratory protocols in protocols.io to enhance the reproducibility of your results. Protocols.io assigns your protocol its own identifier (DOI) so that it can be cited independently in the future. For instructions see: https://journals.plos.org/plosone/s/submission-guidelines#loc-laboratory-protocols. Additionally, PLOS ONE offers an option for publishing peer-reviewed Lab Protocol articles, which describe protocols hosted on protocols.io. Read more information on sharing protocols at https://plos.org/protocols?utm_medium=editorial-email&utm_source=authorletters&utm_campaign=protocols.

As the corresponding author, your ORCID iD is verified in the submission system and will appear in the published article. PLOS supports the use of ORCID, and we encourage all coauthors to register for an ORCID iD and use it as well. Please encourage your coauthors to verify their ORCID iD within the submission system before final acceptance, as unverified ORCID iDs will not appear in the published article. *Only*  the individual author can complete the verification step; PLOS staff *cannot*  verify ORCID iDs on behalf of authors.

We look forward to receiving your revised manuscript.

Kind regards,

UDAYAN BHATTACHARYA

Academic Editor

PLOS One

Journal Requirements:

When submitting your revision, we need you to address these additional requirements.

1. Please ensure that your manuscript meets PLOS ONE's style requirements, including those for file naming. The PLOS ONE style templates can be found at

https://journals.plos.org/plosone/s/file?id=wjVg/PLOSOne_formatting_sample_main_body.pdf and

https://journals.plos.org/plosone/s/file?id=ba62/PLOSOne_formatting_sample_title_authors_affiliations.pdf

2. Please note that PLOS One has specific guidelines on code sharing for submissions in which author-generated code underpins the findings in the manuscript. In these cases, we expect all author-generated code to be made available without restrictions upon publication of the work. Please review our guidelines at https://journals.plos.org/plosone/s/materials-and-software-sharing#loc-sharing-code and ensure that your code is shared in a way that follows best practice and facilitates reproducibility and reuse.

3. Please update your submission to use the PLOS LaTeX template. The template and more information on our requirements for LaTeX submissions can be found at http://journals.plos.org/plosone/s/latex.

4. Thank you for stating the following in the Acknowledgments Section of your manuscript:

“This work was supported by a research grant (Grant ID: MBRU-CM-RG2023-02) awarded to Dr. Yajnavalka Banerjee, by Mohammed Bin Rashid University of Medicine and Health Sciences – Dubai Health. The funding body had no role in the design of the study, collection, analysis, and interpretation of data, or in writing the manuscript.”

We note that you have provided funding information that is not currently declared in your Funding Statement. However, funding information should not appear in the Acknowledgments section or other areas of your manuscript. We will only publish funding information present in the Funding Statement section of the online submission form.

Please remove any funding-related text from the manuscript and let us know how you would like to update your Funding Statement. Currently, your Funding Statement reads as follows:

“This work was supported by a research grant (Grant ID: MBRU-CM-RG2023-02) awarded to Dr. Yajnavalka Banerjee, by Mohammed Bin Rashid University of Medicine and Health Sciences – Dubai Health.”

Please include your amended statements within your cover letter; we will change the online submission form on your behalf.

5. Thank you for stating the following financial disclosure:

“This work was supported by a research grant (Grant ID: MBRU-CM-RG2023-02) awarded to Dr. Yajnavalka Banerjee, by Mohammed Bin Rashid University of Medicine and Health Sciences – Dubai Health.”

Please state what role the funders took in the study.  If the funders had no role, please state: "The funders had no role in study design, data collection and analysis, decision to publish, or preparation of the manuscript."

If this statement is not correct you must amend it as needed.

Please include this amended Role of Funder statement in your cover letter; we will change the online submission form on your behalf.

6. Thank you for stating the following financial disclosure:

“This work was supported by a research grant (Grant ID: MBRU-CM-RG2023-02) awarded to Dr. Yajnavalka Banerjee, by Mohammed Bin Rashid University of Medicine and Health Sciences – Dubai Health.”

We note that one or more of the authors is affiliated with the funding organization, indicating the funder may have had some role in the design, data collection, analysis or preparation of your manuscript for publication; in other words, the funder played an indirect role through the participation of the co-authors. If the funding organization did not play a role in the study design, data collection and analysis, decision to publish, or preparation of the manuscript and only provided financial support in the form of authors' salaries and/or research materials, please do the following:

1. Review your statements relating to the author contributions, and ensure you have specifically and accurately indicated the role(s) that these authors had in your study. These amendments should be made in the online form.

2. Confirm in your cover letter that you agree with the following statement, and we will change the online submission form on your behalf:

“The funder provided support in the form of salaries for authors [insert relevant initials], but did not have any additional role in the study design, data collection and analysis, decision to publish, or preparation of the manuscript. The specific roles of these authors are articulated in the ‘author contributions’ section.

7. Please expand the acronym “MBRU” (as indicated in your financial disclosure) so that it states the name of your funders in full.

This information should be included in your cover letter; we will change the online submission form on your behalf.

8. PLOS ONE now requires that authors provide the original uncropped and unadjusted images underlying all blot or gel results reported in a submission’s figures or Supporting Information files. This policy and the journal’s other requirements for blot/gel reporting and figure preparation are described in detail at https://journals.plos.org/plosone/s/figures#loc-blot-and-gel-reporting-requirements and https://journals.plos.org/plosone/s/figures#loc-preparing-figures-from-image-files. When you submit your revised manuscript, please ensure that your figures adhere fully to these guidelines and provide the original underlying images for all blot or gel data reported in your submission. See the following link for instructions on providing the original image data: https://journals.plos.org/plosone/s/figures#loc-original-images-for-blots-and-gels.

In your cover letter, please note whether your blot/gel image data are in Supporting Information or posted at a public data repository, provide the repository URL if relevant, and provide specific details as to which raw blot/gel images, if any, are not available. Email us at plosone@plos.org if you have any questions.

9. Please include captions for your Supporting Information files at the end of your manuscript, and update any in-text citations to match accordingly. Please see our Supporting Information guidelines for more information: http://journals.plos.org/plosone/s/supporting-information.

10. If the reviewer comments include a recommendation to cite specific previously published works, please review and evaluate these publications to determine whether they are relevant and should be cited. There is no requirement to cite these works unless the editor has indicated otherwise.

Additional Editor Comments:

Manuscript Number: PONE-D-26-17270

Title: Epigallocatechin-3-gallate Attenuates PAR-2–mediated Inflammatory Signaling and Preserves Mitochondrial Integrity in Osteoarthritic Chondrocytes

Journal: PLOS ONE

Dear Dr. Banerjee,

Thank you for submitting your manuscript to PLOS ONE. Your work has been evaluated by our peer reviewers, and their detailed comments are appended below.

As you will see, while the reviewers find your investigation into the effects of Epigallocatechin-3-gallate on osteoarthritic chondrocytes to be highly interesting and relevant, they have raised significant concerns regarding the data and methodology that must be addressed before the manuscript can be considered for publication. Therefore, we invite you to submit a revised version of the manuscript that thoroughly addresses these points.

In your revision, please ensure you carefully address the major methodological clarifications and any technical concerns raised, particularly those highlighted by Reviewers 1 and 3.

Please submit your revised manuscript along with a detailed, point-by-point response to the reviewers' comments, explicitly outlining the modifications and text changes made in response to each point.

Reviewer Overview:

Reviewer 1 : Major Revision

Reviewer 2 : Minor Revision

Reviewer 3 : Major Revision

(Insert detailed peer review reports here)

We look forward to receiving your revised manuscript.

Sincerely,

Dr. Udayan Bhattacharya

Academic Editor

PLOS ONE

[Note: HTML markup is below. Please do not edit.]

Reviewers' comments:

Reviewer's Responses to Questions

**Comments to the Author**

1. Is the manuscript technically sound, and do the data support the conclusions?

Reviewer #1: Partly

Reviewer #2: Partly

Reviewer #3: Partly

**********

2. Has the statistical analysis been performed appropriately and rigorously?

Reviewer #1: No

Reviewer #2: Yes

Reviewer #3: Yes

**********

3. Have the authors made all data underlying the findings in their manuscript fully available?

The PLOS Data policy

Reviewer #1: Yes

Reviewer #2: Yes

Reviewer #3: No

**********

4. Is the manuscript presented in an intelligible fashion and written in standard English?

Reviewer #1: Yes

Reviewer #2: Yes

Reviewer #3: Yes

**********

Reviewer #1: The manuscript investigates the role of epigallocatechin-3-gallate (EGCG) in modulating PAR-2-mediated inflammatory signaling in osteoarthritic chondrocytes. The study is well structured and addresses a relevant mechanistic question. The use of multiple experimental approaches, including qPCR, Western blot, ELISA, flow cytometry, and siRNA, strengthens the study design.

However, several important issues should be addressed:

Major concerns:

* The study is entirely based on an in vitro model, which limits its biological and clinical relevance.

* The sample size (n=3 biological replicates) is insufficient to ensure robustness and reproducibility.

* The statistical analysis is not appropriate, as multiple t-tests were used instead of one-way ANOVA with proper post hoc correction.

* The use of LPS as a model for osteoarthritis inflammation remains artificial and should be more critically discussed.

* The claim that the tested EGCG concentrations are physiologically achievable requires stronger supporting evidence.

Minor concerns:

* The manuscript is overly verbose in several sections and would benefit from more concise writing.

* Some figure descriptions require clearer explanation.

* The discussion overstates the translational significance of the findings.

Overall, the study provides useful mechanistic insights, but significant revisions are required before it can be considered for publication.

Reviewer #2: The study provides a comprehensive mechanistic look at the PAR-2-ERK1/2-SOX4-ADAMTS-5 signaling axis in a chondrocyte model. The siRNA validation is a particular strength that elevates the work from correlative to causative. To meet the criteria for publication in PLOS ONE, a few minor refinements are required:

1. Sensitivity Analysis: While your pairwise t-test approach is justified, please verify if results maintain significance under a standard one-way ANOVA with Dunnett’s post-hoc test to ensure the robustness of the findings across common statistical frameworks.

2. Cell Model Limitations: Explicitly acknowledge in the limitations that BM-MSC-derived chondrocytes, while phenotypically validated, may not fully capture the epigenetic "drift" characteristic of chronically diseased primary human OA tissue.

3. Pathway Specificity: The siRNA data show that PAR-2 accounts for approximately 38% of the LPS-induced TNF-a output. Because substantial EGCG efficacy remains after silencing, the discussion should more clearly frame EGCG as a multifunctional agent where PAR-2 is a predominant node rather than an exclusive target.

4. Mitochondrial Claims: Since functional bioenergetic assays (e.g., Seahorse OCR or ATP quantification) were not performed, please refine the "mitoprotective" claims to specify that EGCG preserves structural and thermodynamic markers of mitochondrial integrity

Reviewer #3: Although the results of the study are interesting, it is hard at times to determine whether this was a review article or a study. The authors converted the discussion section into a dissertation-like chapter, which is not appropriate for a journal article. There's just too much information that it dilutes the actual study. The figure legends are incorporated within the main text of the manuscript. This should not be the case, and the figure legends in a separate section on their own.

1. In the methods, specifically "Ethics considerations for the present study," there is a lot of repetitive mentioning of not using human subjects and using cell lines. That paragraph could be written in a much more concise manner, as it is a bit confusing when written like that.

2. In the methods, specifically "Cytotoxicity assessment, inflammatory chondrocyte model, and TNF-α quantification," there are two huge paragraphs that go deep into the rationale for using LPS to induce a pro-inflammatory response. I think those 2 big paragraphs should go into the discussion section. You may just use a sentence or so to summarize the rationale within the methods, but all that detailed mechanistic information is more appropriate for the discussion section.

3. There are several instances where words containing dashes are used but the dashes are not supposed to be there. Please proofread the manuscript carefully line-by-line. For example: an-ti-human (the dash after the "n" should not be there), and Di-agnostics (the dash after the "i" should not be there). There are many other examples in the manuscript that need to be looked at.

4. In Figure 1, please include the phase contrast image of the BM-MSCs 28 days post-induction. You only included pre-induction. Readers should be able to look at both in the figure.

5. In the figure legend for Figure 1, you mention the following, but this is more appropriate to be included in the corresponding methods section: "(Note: Chondrogenic differentiation and histochemical/immunocytochemical staining were performed as validation of our previously established protocol, as described in Patnaik et al [17, 18, 25])."

6. Please include why you chose those concentrations of EGCG and LPS, and whether the concentrations are physiological or supraphysiological (i.e., realistic if one drinks a cup of green tea; how much EGCG is actually absorbed? Is this even known?? and the same for LPS...what is the minimum physiological concentration that induces an inflammatory response?)

7. In Figure 2, the fonts are not consistent, as there is both Arial and Times New Roman font. Please just keep one font. The font size is also not consistent on axis labels. The x-axis labels are too verbose. Also, usually mRNA expression is shown first, followed by protein expression, going in chronological order of synthesis. In other words, panel C should actually be A. This should also be fixed in the methods section, as RNA synthesis should be before protein synthesis. Also, Why is there a black bar on the right side labeled PAR-2? It is implied from the x-axis label that this is PAR2 expression. Again, please fix the x-axis labels.

8. Figure 2 would benefit from a different way of labeling the samples. I would just use:

"Ctrl" as a symbol for "Chondrocytes"

"LPS" instead of "Chondrocytes + LPS"

"LPS + EGCG-5uM" instead of "Chondrocytes + LPS + EGCG-5uM"

"LPS + EGCG-10uM" instead of "Chondrocytes + LPS + EGCG-10uM"

9. When comparing the original blot image for PAR2 in Figure 2 to the actual cropped figure, you can see that the image is upside down. The non-specific bands in the original image are below PAR2 bands, but in the cropped manuscript image, the non-specific bands are above PAR2 bands. Please keep the image in the correct position. Also, there is no need to crop GAPDH so close to the bands that the border is almost touching the bands. Please fix this too.

10. In the Figure 2 legend, you mention "2−ΔΔCT". This is incorrect. It should be 2^−ΔΔCT, meaning the "−ΔΔCT" part should be a superscript (exponent). Please also fix this in the methods section.

11. The same sample and x-axis labeling that I pointed out for Figure 2 also exist in Figure 3, 4, 5, 6, 7, and 8. It is not appropriate to label the samples 1, 2, 3, 4, etc. for a publication in a peer-reviewed journal. Please find a way to label the samples as explained in a previous comment.

12. In Figure 3, the fonts are not all the same. The whole figure is actually very confusing and cannot stand on its own as a proper figure. This figure needs significant changes. Since you altered the original blot to exclude unwanted bands, you should add a dashed line between the two bands that were "attached" to each other. In addition, I really don't understand why the figure has lower quality bands in that they are stretched and very thin compared to the original blot. Again, please label the samples correctly as mentioned in a previous comment. And once more, please have mRNA expression panels first, followed by protein expression panels. The x-axis label for Figure 3-D is not the correct label.

13. For Figure 4, again, "1" "2" "3" "4" is not proper labeling of samples. mRNA expression should go first, followed by ELISA. Also, the authors should explain why they used ELISA instead of western blotting? Also, there is no need to use numbers...this should have been a bar graph, and the numbers succinctly shown in the text of the manuscript. Figure 4B is also missing the axes borders...And why did you include EGCG of 1 uM and 20 uM concentrations??? This is not consistent with other figures.

14. For Figure 5, again, "1" "2" "3" "4" is not proper labeling of samples. Please reorder Figure 5 so that mRNA expression is first, followed by protein expression, for each candidate gene/protein. Again, I don't understand why you have the legend for Figure 5 in between the text for SOX4 and ADAMTS5?? This makes it very hard to follow the manuscript! There is also no need to manipulate the bands such that are are so thin, as it makes it difficult to analyze visually. Please use the original bands.

15. Figure 6 labeling is also confusing. Figures must be able to stand on their own. One cannot figure out the difference between the right and left side of the figure in terms of treatment unless you read the figure legend...

17. Figure 7 labeling of A, B, C, D..etc is not consistent with other figures. Please make sure you use proper and consistent labeling of samples.

18. Figure 8...again, again, "1" "2" "3" "4" etc. is not proper labeling of samples. You did not include the original blot for this figure. Also, you did not include band sizes in the figure...Also, there are no panels indicated in this figure...like Figure 8A, 8B, etc. However, it is included in the text...please fix the figure to be consistent with the text! This figure needs to be redone completely. There is no need for a line graph...this is not consistent with how data were presented in other figures...please use bar graphs with the SD shown.

19. Figure 8...TNF alpha mRNA expression should be first, followed by ELISA...also why using ELISA when other figures have western blots???

20. The paragraph that starts with "Two conclusions emerge from these data." belongs in the DISCUSSION section!

21. Figure 9 should have its own brief section in the Results section before discussing in the discussion section.

22. There is no need for all these tables! I don't understand why the authors could not have simply summarized all that information in the discussion!

23. The discussion section is extremely long and difficult to read with unnecessary tables.

**********

what does this mean?). If published, this will include your full peer review and any attached files.

If you choose “no”, your identity will remain anonymous but your review may still be made public.

**Do you want your identity to be public for this peer review?**  For information about this choice, including consent withdrawal, please see our Privacy Policy

Reviewer #1: No

Reviewer #2: No

Reviewer #3: No

**********

[NOTE: If reviewer comments were submitted as an attachment file, they will be attached to this email and accessible via the submission site. Please log into your account, locate the manuscript record, and check for the action link "View Attachments". If this link does not appear, there are no attachment files.]

To ensure your figures meet our technical requirements, please review our figure guidelines: https://journals.plos.org/plosone/s/figures

You may also use PLOS’s free figure tool, NAAS, to help you prepare publication quality figures: https://journals.plos.org/plosone/s/figures#loc-tools-for-figure-preparation.

NAAS will assess whether your figures meet our technical requirements by comparing each figure against our figure specifications.

# Author comment: Author response to Decision Letter 1

**DOI:** 10.1371/journal.pone.0357766.r002

2 Jul 2026

Manuscript PONE-D-26-17270

We thank the Academic Editor and the three reviewers for their time and constructive appraisal. A detailed, point-by-point response to every comment is provided in the uploaded "Response to

Reviewers" file , accompanied by a tracked-changes manuscript and a clean manuscript. The substance is summarised below.

EDITOR

The editorial Journal Requirements are addressed in the cover letter: funding text has been removed from the Acknowledgments; the Financial Disclosure has been updated with the funder named in full and a no-role statement; the original uncropped blots are provided as Supporting Information and on figshare; and the minimal data set is deposited on figshare (DOI: 10.6084/m9.figshare.31960143).

REVIEWER 1 (Major Revision) — all points addressed.

In vitro scope: the in vitro limitation is now stated explicitly and the findings are framed within a structured, multi-level translational evidence hierarchy (Table 4), invoked directly in the Discussion and Limitations.

Sample size: n = 3 is clarified as independent biological replicates (the unit of inference), with supporting methodological references.

Statistics: analysis has been changed from multiple t-tests to one-way ANOVA with Dunnett's post-hoc test throughout the Methods and figure legends, with an added reference and updated significance keys.

LPS model: the OA-specific TLR4/DAMP rationale has been strengthened (including a metabolic-endotoxaemia argument) and developed in a dedicated Discussion subsection, and the Limitations now give a candid account of what the model does not capture.

EGCG concentrations: Table 3 (36 green teas) is used in full to show the delivered dose greatly exceeds the working range, with an explicit luminal-versus-systemic distinction and the bioavailability strategies that address it.

Verbosity, figure legends, and translational tone (minor points): the Discussion now carries six thematic subheadings, the legends of Figures 4, 7 and 8 have been clarified, and the translational language has been moderated.

REVIEWER 2 (Minor Revision) — all points addressed.

Statistics/sensitivity: the ANOVA/Dunnett framework requested is implemented (in line with Reviewer 1); the reported effects remain significant.

Pathway specificity: a new Discussion paragraph frames EGCG as a multifunctional agent for which PAR-2 is the predominant but not exclusive target, complementing Table 2 (PAR-2-independent pathways).

Mitochondrial claims: the ΔΨm/ATP inferences have been softened and qualified in the "Preservation of mitochondrial integrity" subsection.

REVIEWER 3 (Major Revision) — addressed in full, with two reasoned exceptions.

We have implemented the figure-quality, typography, sample-labelling, panel re-lettering, and structural revisions requested (including a new integrated signalling-model figure and relocation of interpretive text to the Discussion). We respectfully retain two points, each with a detailed justification in the response: the protein-before-mRNA panel order (Comment 7, applied consistently across the figures and Methods) and the three Discussion summary tables (Comments 22–23), which are the evidentiary basis of responses already accepted by Reviewers 1 and 2 and whose removal would re-open those concerns.

We hope the revised manuscript is now suitable for publication and look forward to your decision.

Regards

Yajnavalka Banerjee, PhD

Attachment Submitted filename: Cover Letter with Detailed Response_YB.pdf

# Peer review: Decision Letter 1

**DOI:** 10.1371/journal.pone.0357766.r003

**Authors:** UDAYAN BHATTACHARYA

5 Aug 2026

PLOS One

Dear Dr. Banerjee,

Thank you for submitting your manuscript to PLOS ONE. After careful consideration, we feel that it has merit but does not fully meet PLOS ONE’s publication criteria as it currently stands. Therefore, we invite you to submit a revised version of the manuscript that addresses the points raised during the review process.

Please submit your revised manuscript by Sep 19 2026 11:59PM. If you will need more time than this to complete your revisions, please reply to this message or contact the journal office at plosone@plos.org. When you're ready to submit your revision, log on to https://www.editorialmanager.com/pone/ and select the 'Submissions Needing Revision' folder to locate your manuscript file.

Please include the following items when submitting your revised manuscript:

- A letter that responds to each point raised by the academic editor and reviewer(s). You should upload this letter as a separate file labeled 'Response to Reviewers'.
- A marked-up copy of your manuscript that highlights changes made to the original version. You should upload this as a separate file labeled 'Revised Manuscript with Track Changes'.
- An unmarked version of your revised paper without tracked changes. You should upload this as a separate file labeled 'Manuscript'.

If you would like to make changes to your financial disclosure, please include your updated statement in your cover letter. Guidelines for resubmitting your figure files are available below the reviewer comments at the end of this letter.

If applicable, we recommend that you deposit your laboratory protocols in protocols.io to enhance the reproducibility of your results. Protocols.io assigns your protocol its own identifier (DOI) so that it can be cited independently in the future. For instructions see: https://journals.plos.org/plosone/s/submission-guidelines#loc-laboratory-protocols. Additionally, PLOS ONE offers an option for publishing peer-reviewed Lab Protocol articles, which describe protocols hosted on protocols.io. Read more information on sharing protocols at https://plos.org/protocols?utm_medium=editorial-email&utm_source=authorletters&utm_campaign=protocols.

As the corresponding author, your ORCID iD is verified in the submission system and will appear in the published article. PLOS supports the use of ORCID, and we encourage all coauthors to register for an ORCID iD and use it as well. Please encourage your coauthors to verify their ORCID iD within the submission system before final acceptance, as unverified ORCID iDs will not appear in the published article. Only the individual author can complete the verification step; PLOS staff cannot verify ORCID iDs on behalf of authors.

We look forward to receiving your revised manuscript.

Kind regards,

UDAYAN BHATTACHARYA

Academic Editor

PLOS One

Journal Requirements:

If the reviewer comments include a recommendation to cite specific previously published works, please review and evaluate these publications to determine whether they are relevant and should be cited. There is no requirement to cite these works unless the editor has indicated otherwise.

Please review your reference list to ensure that it is complete and correct. If you have cited papers that have been retracted, please include the rationale for doing so in the manuscript text, or remove these references and replace them with relevant current references. Any changes to the reference list should be mentioned in the rebuttal letter that accompanies your revised manuscript. If you need to cite a retracted article, indicate the article’s retracted status in the References list and also include a citation and full reference for the retraction notice.

**Additional Editor Comments:**

Dear Dr. Banerjee and Co-Authors,

Thank you for submitting the revised version of your manuscript to PLOS ONE. We have now received the feedback from the reviewers who evaluated Revision 1 of your manuscript.

As you will see from the detailed comments below, the reviewers appreciate the efforts made in addressing the initial concerns. While one reviewer has recommended acceptance, the other reviewers have identified a few minor remaining points that require your attention before the manuscript can be formally accepted for publication.

Therefore, we invite you to submit a Minor Revision addressing these final comments.

Instructions for Re-submission:

Point-by-Point Response: Please provide a detailed, point-by-point response letter addressing each comment raised by the reviewers.

Tracked Changes: Submit both a clean version of your revised manuscript and a marked-up copy highlighting all modifications made to the text.

Data & Code Availability: Ensure all supplementary data, underlying dataset files, and figure source data comply with PLOS ONE data availability guidelines.

Regards

[Note: HTML markup is below. Please do not edit.]

Reviewers' comments:

Reviewer's Responses to Questions

**Comments to the Author**

Reviewer #1: (No Response)

Reviewer #2: All comments have been addressed

Reviewer #3: All comments have been addressed

**********

2. Is the manuscript technically sound, and do the data support the conclusions?

Reviewer #1: Yes

Reviewer #2: Yes

Reviewer #3: Partly

**********

3. Has the statistical analysis been performed appropriately and rigorously?

Reviewer #1: Yes

Reviewer #2: (No Response)

Reviewer #3: Yes

**********

4. Have the authors made all data underlying the findings in their manuscript fully available?

The PLOS Data policy

Reviewer #1: Yes

Reviewer #2: Yes

Reviewer #3: Yes

**********

5. Is the manuscript presented in an intelligible fashion and written in standard English?

Reviewer #1: Yes

Reviewer #2: Yes

Reviewer #3: (No Response)

**********

Reviewer #1: I appreciate the considerable effort the authors have invested in revising the manuscript. The major concerns raised during the previous round of review have been addressed satisfactorily. The statistical analysis has been strengthened through the implementation of one-way ANOVA with appropriate post hoc testing, the rationale for the inflammatory model has been expanded, the discussion has been substantially improved, and the claims regarding mitochondrial protection have been appropriately moderated.

The manuscript is technically sound, the experiments are well organized, and the conclusions are generally supported by the presented data. The incorporation of PAR-2 silencing experiments further strengthens the mechanistic interpretation of the findings.

I have only a few minor suggestions before publication:

1. Consider replacing a few definitive expressions such as “establishes” or “confirms” with more cautious wording such as “supports” or “suggests”, given that the conclusions are derived from an in vitro model.

2. The Discussion remains relatively lengthy and contains some repetition of the Results. Moderate condensation would improve readability.

3. The Western blot methodology contains a repeated description of densitometric analysis, which should be removed for clarity.

4. If not already included within the supplementary material, providing the representative flow cytometry gating strategy would further improve transparency.

These are minor editorial suggestions only and do not affect the scientific validity of the study.

Reviewer #2: (No Response)

Reviewer #3: The revised manuscript still needs minor improvements (and they are very numerous). In addition, low quality (low dpi) figures were submitted, making it difficult to approve the figures. The authors need to make a serious effort in improving the quality of the figures. They do not look neat.

1. I'm still noticing several typographical errors. Authors: please do a careful line-by-line review, as I may not be able to notice all the errors. Please spend a lot of time going through the manuscript carefully. Publications with several typos can hurt the reader's confidence in the study's results.

2. Line 6 of the abstract starts with the number 4. Please remove the number.

3. In the Methods section, line 4 AND line 12 of the subsection "Cell culture and chondrogenic differentiation": the 2 in CO2 should be a subscript not a superscript. Last sentence of that subsection should state: "validate the chondrocytic phenotype following [NOT FOLLOW] our previously established."

4. Please make sure to be consistent with the journal's preference for how to format phrases as in vivo and in vitro, as sometimes I see them italicized, and sometimes not.

5. In the Western blotting subsection of the Methods, please be clear in the last sentence if "replicate" refers to technical or biological. In the same subsection, the 4th to last sentence ending, there is a space between the period and the last word.

6. I see "in triplicate" in other subsections...is this technical or biological? Please specify.

7. In the statistics subsection of the methods, I find that the following paragraph to be completely unnecessary. People who perform such experiments regularly already know this information. It's redundant. I think the authors should just simply state whether the replicates were technical or biological....

"For each assay, n = 3 denotes three independent biological replicates, defined as fully separate chondrocyte cultures established, differentiated, treated, and assayed on different occasions, rather than technical replicates of a single culture; this constitutes the unit of statistical inference in accordance with established guidance on what validly constitutes “n” in cell-culture experiments [31, 32]. Three independent biological replicates represent the conventional minimum for hypothesis-driven in vitro mechanistic studies, and the robustness of the present conclusions is further reinforced by the convergence of orthogonal, methodologically independent readouts (qPCR, immunoblotting, ELISA, flow cytometry, and siRNA-mediated loss of function), by the reproducible, dose-dependent nature of the responses, and by the large effect sizes obtained, which collectively provide a stronger basis for inference than replicate count considered in isolation [33]. "

8. Figure 1:

-The sizes of the boxes for each panel is inconsistent.

-Panel B box line seems thicker than the box for Panel A.

-The letter E seems to be a smaller size than the other letters.

-Letter G for panel G is on the left but for Panel F, the letter F is on the right? Also, the y-axis for Panel G is part of Panel F. Please make this look neat.

9. In the text for the "Cytotoxicity profiling of EGCG and LPS" subsection of the Results, you are not referring to the correct panels...please fix.

10. Font sizes are not consistent for Figure 2. Some are not readable. Please fix and make look neater.

11. Figure 2 legend shows: ***< 0.001 . It's missing the letter p.

12. Why is Figure 3 so small compared to other figures? The size ratios are off. Not neat. The image is also not good quality and is blurry.

13. Figures 2, 3, and 5: the sample labels above the lanes in the blots are too small, hard to read.

14. The text in the Results section dedicated to figure 9 is absolutely unnecessary! It is so verbose! Please find a way to make your point across concisely!

15. The discussion section is extremely long and verbose! The authors should find a more concise way to convey the information.

16. The tables in the discussion section should just be included as part of supplementary information, and not included physically in the discussion.

17. I find that there are not enough references used in the discussion, especially the limitations section. The whole discussion needs to be dramatically cut down and improved. Too many words! Please find a way to get your point across succinctly!

**********

what does this mean?). If published, this will include your full peer review and any attached files.

If you choose “no”, your identity will remain anonymous but your review may still be made public.

**Do you want your identity to be public for this peer review?**  For information about this choice, including consent withdrawal, please see our Privacy Policy

Reviewer #1: No

Reviewer #2: No

Reviewer #3: No

**********

[NOTE: If reviewer comments were submitted as an attachment file, they will be attached to this email and accessible via the submission site. Please log into your account, locate the manuscript record, and check for the action link "View Attachments". If this link does not appear, there are no attachment files.]

To ensure your figures meet our technical requirements, please review our figure guidelines: https://journals.plos.org/plosone/s/figures

You may also use PLOS’s free figure tool, NAAS, to help you prepare publication quality figures: https://journals.plos.org/plosone/s/figures#loc-tools-for-figure-preparation.

NAAS will assess whether your figures meet our technical requirements by comparing each figure against our figure specifications.

# Author comment: Author response to Decision Letter 2

**DOI:** 10.1371/journal.pone.0357766.r004

16 Aug 2026

16th August 2026

The Academic Editor

PLOS ONE

Dr. Udayan Bhattacharya

Manuscript ID: PONE-D-26-17270R1

Title: Epigallocatechin-3-gallate Attenuates PAR-2–mediated Inflammatory Signaling and Preserves Markers of Mitochondrial Integrity in Osteoarthritic Chondrocytes

Dear Dr Bhattacharya,

We are grateful for the opportunity to submit the second revision of the above manuscript, and we thank the reviewers for the continued care they have given to it. We are particularly pleased that Reviewer #1 considers the major concerns of the first round to have been addressed satisfactorily, and that the statistical analysis, the rationale for the inflammatory model, the expanded Discussion, the moderated mitochondrial claims and the PAR-2 silencing experiment were each recognised in that assessment.

Every comment received in this round has been addressed, and a full point-by-point reply accompanies this letter. All edits are visible as tracked changes in the revised manuscript. Below we summarise, first, the changes we have made; second, the small number of points where we have respectfully retained the present arrangement, with our reasons; and third — offered in a wholly constructive spirit — a few instances in which the guidance we have received points in opposing directions, and on which your steer would be very welcome.

1. Changes made in this revision

Calibration of language and claims (Reviewer #1, Comment 1)

• A systematic audit was performed across the Abstract, the final paragraph of the Introduction, the Results text and figure legends, the integrated mechanistic model, the Discussion, the translational statements and the Conclusions, replacing definitive expressions such as “demonstrate”, “establishes” and “confirms” with “report”, “supports” and “suggests”.

• Definitive terminology was deliberately retained only where it describes a directly observed assay result, a routine methodological validation, or a finding attributed to cited external literature.

Condensation of the Discussion (Reviewer #1, Comment 2)

• Seven itemised condensations were made: sentences that restated the Results were excised, the two paragraphs that twice drew the same conclusion on PAR-2 dependence were collapsed, and the survey of PAR-2–independent pathways was tightened.

• Approximately 1,140 words of prose were deleted against approximately 240 words of replacement — a net reduction of approximately 900 words. No reference was removed and no table was altered.

Methods (Reviewer #1, Comment 3; Reviewer #3, Comments 3, 5, 6 and 7)

• The duplicated description of densitometric analysis in the Western blotting subsection has been removed.

• Every instance of “in triplicate” now specifies “(three independent biological replicates)”, making the basis of replication explicit throughout the Methods.

• The n = 3 explanatory paragraph in the Statistical analysis section — itself introduced in the first round at Reviewer #1’s request — has been reduced to the single plain statement requested; the three supporting methodological references are retained.

• Both occurrences of CO₂ have been correctly subscripted, and the grammatical correction to the closing sentence of the subsection has been made.

New Supporting Information (Reviewer #1, Comment 4)

• A representative flow-cytometry gating strategy has been prepared and added as S1 Fig, showing sequential exclusion of debris (FSC-A vs SSC-A), single-cell discrimination (FSC-A vs FSC-H) and the RANK- and RANKL-positive thresholds. Cross-references have been inserted in the Methods and Results, and a Supporting Information caption added.

Figures (Reviewer #3, Comments 8, 9, 10, 11, 12 and 13)

• Figure 1: all panels re-set on a single ruled layout grid so that panel sizes match within each category; a uniform 0.75 pt border stroke applied throughout; all panel labels (A–G) standardised in typeface, size, weight and position.

• Results: the two Figure 1 viability callouts corrected to Fig. 1F and Fig. 1G following the earlier panel re-lettering, and all remaining Figure 1 callouts re-checked.

• Figure 2: re-typeset in a single typeface with consistent point sizes within each label category, and exported at higher resolution.

• Figure 2 legend: the italic p restored to the third significance tier (“***p < 0.001”), and the significance keys in every figure legend harmonised to the identical three-tier convention.

• Figure 3: re-proportioned from its former wide landscape format to match the other multi-panel figures, and re-exported at higher resolution.

• Blot lane labels (Ctrl, LPS, LPS + EGCG-5 µM, LPS + EGCG-10 µM) enlarged in Figures 2, 3 and 5 for unambiguous legibility at print size.

Typography, punctuation and notation (Reviewer #3, Comments 1, 2 and 4)

• A manuscript-wide, line-by-line audit of typographical, punctuation, cross-reference and scientific-notation errors was undertaken, with each correction shown as a tracked change and itemised in a correction matrix in the response document. No bibliographic reference or citation number was altered by any correction.

• The stray numeral “4” has been removed from the Abstract.

• The Latin experimental-context terms in vitro, in vivo, ex vivo, in situ and in silico have been set uniformly in italic throughout the running text — 41 instances standardised.

References added (Reviewer #3, Comment 17)

• Nine references have been added to the Limitations, attached to seven existing sentences, together with one re-citation of a reference already in the list. No sentence, clause or word of text was added to accommodate them — the citations were appended to claims the Limitations already makes, so that the section is better evidenced without becoming any longer.

2. Points respectfully retained, with reasons

In four instances we have, after careful consideration, retained the present arrangement. In each case our reasoning is set out in full in the accompanying response.

• The Results subsection presenting Fig 9 (Reviewer #3, Comment 14). This subsection exists because Reviewer #3 requested it in the first round (Round 1, Comment 21: “Figure 9 should have its own brief section in the Results section before discussing in the discussion section”). Its module-by-module structure records which nodes of the model were measured in this study and which are canonical architecture — the distinction that Reviewer #2 (Comments 3 and 4) and Reviewer #1 (Minor Concern 3) required us to preserve. Condensing it would restore claims we were asked to withdraw.

• The three Discussion tables (Reviewer #3, Comment 16). Tables 2, 3 and 4 carry 49 of the Discussion’s 112 citation markers and 18 of its references, eight of which are cited nowhere else in the manuscript. Each is also the evidentiary basis of an accepted response to another reviewer: Table 2 to Reviewer #2 (Comments 3 and 4), Table 3 to Reviewer #1 (Major Concern 5), and Table 4 to Reviewer #1 (Major Concern 1). Relocating them would remove the referencing from the section, move those answers out of the article, and — were the content restored as prose — lengthen the Discussion rather than shorten it.

• Further condensation of the Discussion (Reviewer #3, Comments 15 and 17). The Discussion was condensed by approximately 900 net words earlier in this same revision cycle at Reviewer #1’s request, and by six thematic subheadings plus two rewritten passages in the previous round. We would be glad for these comments to be weighed against the condensed text.

• Protein-before-transcript panel order, and the Figure 1 F/G label point. The protein-first arrangement is retained for the four referenced reasons given in the previous round, and is uniform across every multi-panel figure and mirrored in the Methods. On the Figure 1 label positions and Y-axes, the arrangement described does not correspond to the source artwork; we would respectfully ask that the figures be assessed against the individually uploaded full-resolution TIFF files rather than the compiled review-build PDF.

3. A few points on which the guidance received pulls in opposite directions

We raise the following with genuine deference, and with full appreciation that they arise from three reviewers reading the manuscript independently and in good faith, each from a different vantage point. We mention them only because, in each pairing, satisfying one request necessarily undoes the other, and we are reluctant to choose between reviewers without your guidance.

(a) Within the present round — Reviewer #3, Comments 16 and 17

• Comment 17 finds the Discussion insufficiently referenced. Comment 16 asks that the Discussion tables be moved to Supporting Information. The tables are, however, where much of the Discussion’s referencing physically sits: Table 2 carries a “Key References” column, Table 3 a “Ref” column, and in Table 4 the first column is headed “Reference”, each row being an individual study. Acting on Comment 16 would remove 49 citation markers and 18 references from the section that Comment 17 finds under-referenced, and would move eight references out of the article altogether.

(b) Within the present round — Reviewer #3, Comment 17 read against itself

• The same comment asks both for additional references and for the Discussion to be “dramatically cut down”. We have sought to honour both by adding citations without adding a single word of accompanying text; we mention it only to explain why the new references appear without explanatory sentences around them.

(c) Across the two rounds — Reviewer #3, Round 1 Comment 21 and Round 2 Comment 14

• Round 1, Comment 21 asked that Fig 9 be given its own section in the Results, which we created for that purpose. Round 2, Comment 14 describes that same section as unnecessary. We have retained it, since removing it would return the manuscript to the configuration the earlier comment identified as defective.

(d) Across the two rounds — Reviewer #3, Round 1 Comments 2 and 20

• In the previous round this Reviewer asked us to relocate two substantial paragraphs of LPS–TLR4 rationale from the Methods into the Discussion (Comment 2), and to move the interpretive paragraph beginning “Two conclusions emerge from these data” from the Results into the Discussion (Comment 20). Both were implemented in full. The present objection to the length of the Discussion therefore rests in part on material placed there at the Reviewer’s own request.

(e) Across reviewers — Reviewer #1 and Reviewer #3 on the sample size

• Reviewer #1 (Round 1, Comment 2) questioned whether n = 3 was sufficient and asked us to justify it, which we did by adding an explanatory paragraph with three methodological references to the Statistical analysis section. Reviewer #3 (Round 2, Comments 5–7) asks for that paragraph to be reduced to a plain statement. We have followed the more recent request and condensed it, retaining the three references so that Reviewer #1’s concern remains answered.

(f) Across reviewers — Reviewer #2’s requirements and the length of the Discussion

• Much of the Discussion’s present extent is a direct consequence of Reviewer #2’s first-round requirements. Comment 3 asked that EGCG be framed as a multifunctional agent for which PAR-2 is a predominant rather than exclusive node, which we answered with a dedicated Discussion paragraph, three new references and Table 2. Comment 4 asked that the mitochondrial claims be narrowed to structural and thermodynamic markers, which required qualifying language in the Results, the Discussion and the Limitations. Comment 2 asked for an expanded, referenced acknowledgement of the epigenetic limitations of the BM-MSC-derived model, which added a fully referenced paragraph to the Limitations. Each of these additions was necessary and, we believe, improved the manuscript; each also lengthened the Discussion that is now the subject of Reviewer #3’s Comments 15 and 17.

(g) Across reviewers — the tables and Reviewer #1’s Major Concerns

• Table 4 was constructed specifically to answer Reviewer #1’s Major Concern 1 (“the study is entirely based on an in vitro model, which limits its biological and clinical relevance”), and Table 3 to answer Major Concern 5 (“the claim that the tested EGCG concentrations are physiologically achievable requires stronger supporting evidence”). Both are cross-referenced from the Limitations. Moving them to Supporting Information, as Comment 16 proposes, would relocate the manuscript’s answers to two of Reviewer #1’s Major Concerns out of the article body.

(h) Across reviewers — figure legends

• Reviewer #1 (Round 1, Minor Concern 2) asked that several figure descriptions be given clearer explanation, and we accordingly added interpretive statements to the legends of Figures 4, 7 and 8. This runs against the general call for fewer words, and we have retained the added explanation as the more recent and more specific instruction.

4. Guidance respectfully sought

We would be grateful for your direction on a single question: whether Tables 2, 3 and 4 should remain within the Discussion, as we propose and as our responses to Reviewer #1 and Reviewer #2 depend upon, or be relocated to the Supporting Information as Reviewer #3 suggests. We will of course implement whichever arrangement you consider best serves the readership, and we are equally content to be guided by you on any of the other points in Section 3 above.

For completeness, we confirm that no experimental data, figure data point, statistical value or reference has been removed at any stage of this revision; that every textual change is visible as a tracked change in the revised manuscript; and that the full-resolution figure files have been re-uploaded. We remain very grateful to all three reviewers, whose scrutiny across the two rounds has improved the accuracy, the calibration and the presentation of this work considerably.

We hope the revised manuscript is now suitable for publication in PLOS ONE, and we look forward to hearing from you.

Yours sincerely,

Dr. Yajnavalka Banerjee

[Yajnavalka.Banerjee@dubaihealth.ae]

on behalf of all authors

Attachment Submitted filename: Response to Reviewers_YB.docx

# Editor report: Decision Letter 2

**DOI:** 10.1371/journal.pone.0357766.r005

**Authors:** UDAYAN BHATTACHARYA

23 Aug 2026

<p>Epigallocatechin-3-gallate Attenuates PAR-2–mediated Inflammatory Signaling and Preserves Markers of Mitochondrial Integrity in Osteoarthritic Chondrocytes

PONE-D-26-17270R2

Dear Dr. Banerjee,

We’re pleased to inform you that your manuscript has been judged scientifically suitable for publication and will be formally accepted for publication once it meets all outstanding technical requirements.

Within one week, you’ll receive an e-mail detailing the required amendments. When these have been addressed, you’ll receive a formal acceptance letter and your manuscript will be scheduled for publication.

An invoice will be generated when your article is formally accepted. Please note, if your institution has a publishing partnership with PLOS and your article meets the relevant criteria, all or part of your publication costs will be covered. Please make sure your user information is up-to-date by logging into Editorial Manager at Editorial Manager® and clicking the ‘Update My Information' link at the top of the page. For questions related to billing, please contact billing support.

If your institution or institutions have a press office, please notify them about your upcoming paper to help maximize its impact. If they’ll be preparing press materials, please inform our press team as soon as possible -- no later than 48 hours after receiving the formal acceptance. Your manuscript will remain under strict press embargo until 2 pm Eastern Time on the date of publication. For more information, please contact onepress@plos.org.

Kind regards,

UDAYAN BHATTACHARYA

Academic Editor

PLOS One

Additional Editor Comments (optional):

Dear Dr. Banerjee,

Thank you for submitting the second revision of your manuscript, "Epigallocatechin-3-gallate Attenuates PAR-2–mediated Inflammatory Signaling and Preserves Markers of Mitochondrial Integrity in Osteoarthritic Chondrocytes" (PONE-D-26-17270R2).

The reviewers' technical and scientific concerns have been thoroughly and satisfactorily addressed.

Regarding the steer requested in your cover letter concerning Tables 2, 3, and 4:

Please proceed by retaining Tables 2, 3, and 4 within the main Discussion text as you proposed. Their inclusion in the main body is necessary to preserve the evidentiary framework and key citation markers addressing the reviewers' earlier major concerns.

Once you re-upload the final manuscript files confirming this layout, the paper will be formally accepted for publication.

Sincerely,

Dr. Udayan Bhattacharya

Academic Editor, PLOS ONE

Reviewers' comments:

# Editor report: Acceptance letter

**DOI:** 10.1371/journal.pone.0357766.r006

**Authors:** UDAYAN BHATTACHARYA

PONE-D-26-17270R2

PLOS One

Dear Dr. Banerjee,

I'm pleased to inform you that your manuscript has been deemed suitable for publication in PLOS One. Congratulations! Your manuscript is now being handed over to our production team.

At this stage, our production department will prepare your paper for publication. This includes ensuring the following:

* All references, tables, and figures are properly cited

* All relevant supporting information is included in the manuscript submission,

* There are no issues that prevent the paper from being properly typeset

You will receive further instructions from the production team, including instructions on how to review your proof when it is ready. Please keep in mind that we are working through a large volume of accepted articles, so please give us a few days to review your paper and let you know the next and final steps.

Lastly, if your institution or institutions have a press office, please let them know about your upcoming paper now to help maximize its impact. If they'll be preparing press materials, please inform our press team within the next 48 hours. Your manuscript will remain under strict press embargo until 2 pm Eastern Time on the date of publication. For more information, please contact onepress@plos.org.

You will receive an invoice from PLOS for your publication fee after your manuscript has reached the completed accept phase. If you receive an email requesting payment before acceptance or for any other service, this may be a phishing scheme. Learn how to identify phishing emails and protect your accounts at https://explore.plos.org/phishing.

If we can help with anything else, please email us at customercare@plos.org.

Thank you for submitting your work to PLOS One and supporting open access.

Kind regards,

PLOS One Editorial Office Staff

on behalf of

Dr. UDAYAN BHATTACHARYA

Academic Editor

PLOS One
